# The Causal Relationships Between Extrinsic Exposures and Risk of Prostate Cancer: A Phenome-Wide Mendelian Randomization Study

**DOI:** 10.3389/fonc.2022.829248

**Published:** 2022-02-14

**Authors:** Dongqing Gu, Mingshuang Tang, Yutong Wang, Huijie Cui, Min Zhang, Ye Bai, Ziqian Zeng, Yunhua Tan, Xin Wang, Ben Zhang

**Affiliations:** ^1^ Department of Epidemiology and Biostatistics, First Affiliated Hospital, Army Medical University, Chongqing, China; ^2^ Department of Epidemiology and Biostatistics, West China School of Public Health and West China Fourth Hospital, Sichuan University, Chengdu, China; ^3^ School of Public Health and Management, Chongqing Medical University, Chongqing, China

**Keywords:** prostate cancer, Mendelian randomization, risk factor, causal relationship, systematic review

## Abstract

**Background:**

Prostate cancer is the second most common cancer in males worldwide, and multitudes of factors have been reported to be associated with prostate cancer risk.

**Objectives:**

We aim to conduct the phenome-wide exposed-omics analysis of the risk factors for prostate cancer and verify the causal associations between them.

**Methods:**

We comprehensively searched published systematic reviews and meta-analyses of cohort studies and conducted another systematic review and meta-analysis of the Mendelian randomization studies investigating the associations between extrinsic exposures and prostate cancer, thus to find all of the potential risk factors for prostate cancer. Then, we launched a phenome-wide two-sample Mendelian randomization analysis to validate the potentially causal relationships using the PRACTICAL consortium and UK Biobank.

**Results:**

We found a total of 55 extrinsic exposures for prostate cancer risk. The causal effect of 30 potential extrinsic exposures on prostate cancer were assessed, and the results showed docosahexaenoic acid (DHA) [odds ratio (OR)=0.806, 95% confidence interval (CI): 0.661-0.984, *p*=0.034], insulin-like growth factor binding protein 3 (IGFBP-3) (OR=1.0002, 95%CI: 1.00004-1.0004, *p*=0.016), systemic lupus erythematosus (SLE) (OR=0.9993, 95%CI: 0.9986-0.99997, *p*=0.039), and body mass index (BMI) (OR=0.995, 95%CI: 0.990-0.9999, *p*=0.046) were associated with prostate cancer risk. However, no association was found between the other 26 factors and prostate cancer risk.

**Conclusions:**

Our study discovered the phenome-wide exposed-omics risk factors profile of prostate cancer, and verified that the IGFBP-3, DHA, BMI, and SLE were causally related to prostate cancer risk. The results may provide new insight into the study of the pathogenesis of prostate cancer.

## Introduction

Prostate cancer is the second most frequently diagnosed malignancy and the fifth leading cause of cancer-related death among males worldwide (1). In the United States, the estimated new prostate cancer cases reached 191 930, and prostate cancer-related death achieved 33 330 in 2020, making it a malignancy with the highest incidence and second leading cause of mortality in males (2). In 2019, the regions with the most incident cases of prostate cancer were High-income North America, Western Europe, and East Asia. It was reported that the global incident cases were 169.11% higher for prostate cancer during the past 30 year, making it a major global public health challenges (3).

Investigating the risk factors and potential etiological factors for prostate cancer may provide the basis for identifying high-risk populations, developing disease control strategies, and even cognizing the pathogenesis. The burden of prostate cancer was mainly distributed among older men. In addition, epidemiological evidence have established some attributable risk factors for prostate cancer, such as smoking, high body mass index (BMI) and high fasting glucose (3, 4). However, due to the inherent defect of the temporal problem and inadequately controlled confounders in conventional observational studies, the causality between these factors and prostate cancer remains debated.

Mendelian randomization (MR) analysis is a widely used method that uses genetic variants as instrumental variables to inference the causal relationships between potential risk factors and outcomes in observational data in recent years (5). Since the genotypes are presumed to be randomly allocated in gamete formation, Mendelian randomization analysis is not affected by reverse causation. In addition, the inheritance of one exposure predicted by the SNPs is usually independent from the inheritance of another exposure, it is less susceptible to confounding factors (6). Two-sample Mendelian randomization analysis has the additional advantage that access to individual-level data or trait measurements in all samples is not required. Therefore, it can be implemented using summary information for the required genotype-exposure and genotype-outcome associations from separate samples, which significantly increases the scope and efficiency of the approach (7, 8).

Several previous Mendelian randomization studies have identified the etiological factors for prostate cancer, such as serum 25-Hydroxyvitamin D, body mass index (BMI), alcohol consumption, and vitamin B12. However, due to the relatively smaller sample size and lower proportion of variance explained by the instrumental variables, the results are usually inconclusive, and the evidence was insufficient. In this scenario, we aim to first review the published systematic review and meta-analyses of cohort studies and conduct another systematic review and meta-analyses of Mendelian randomization studies to summarize the phenome-wide exposed-omics risk factors for prostate cancer. Next, we conducted two-sample Mendelian randomization analyses to verify the causal relationships using Prostate Cancer Association Group to Investigate Cancer Associated Alterations in the Genome (PRACTICAL) consortium covering 44 825 prostate cancer cases and 27 904 controls, as well as UK Biobank including 6879 prostate cancer cases and 199 891 controls.

## Methods

We obtained summary GWAS statistics from PRACTICAL consortium and UK Biobank (application ID 45973), and all participants included in the consortia were of European ancestry, relevant ethics approval can be found in the original publications (9, 10). Any additional ethical approval was adjudged unnecessary for the present study.

### Potential Risk Factors Identified by the Published Meta-Analysis of Cohort Studies

We searched PubMed, Embase, and Web of Science databases to identify all potential risk factors for prostate cancer reported by the published meta-analysis of cohort studies published in print or online before October 31, 2019. The key terms were as follows: “meta- OR review OR pooled OR consortium OR consortia OR collaboration” AND “Prostate cancer OR prostate adenocarcinoma OR prostate carcinoma OR prostate tumor OR prostate malignancy OR prostate neoplasm”. Inclusion criteria are as follows: (1) meta-analysis of cohort studies; (2) the outcome of interest was prostate cancer; (3) written in the English language. For multiple publications investigating the same factor, the latest publication or publication with the largest sample size was included.

### Systematic Review and Meta-Analysis of Mendelian Randomization Studies

We conducted a systematic review and meta-analysis of published Mendelian randomization studies, and this review was registered in PROSPERO (CRD42021287713). We searched PubMed, Embase, and Web of Science databases to identify all potential risk factors for prostate cancer reported by the Mendelian randomization studies (published in print or online before October 31, 2019) with the following key terms “Prostate cancer OR prostate adenocarcinoma OR prostate carcinoma OR prostate tumor OR prostate malignancy OR prostate neoplasm” AND “Mendelian randomization OR instrumental variable OR causal”. Inclusion criteria are as follows: (1) Mendelian randomization studies to assess the association between exposures and risk of prostate cancer; (2) reported results included odds ratios (ORs) with 95% CIs, which were estimated using an instrumental variable method. When one more study reported data from the same source or databank, only the study with the most participants was included in the analysis. When more than two datasets reported the same factor, the odds ratio (OR) from individual studies were pooled using a random-effects model. Statistical analyses were done using Stata version 15 (Stata, College Station, TX, USA).

### Selection of Factors

Inclusion criteria of factors are as follows: (1) for the same factor, we only included the factor reported to be positive by the largest meta-analysis; (2) dietary factors or internal exposures were excluded. We selected all potential risk factors for prostate cancer identified by the published meta-analysis of cohort studies and Mendelian randomization studies. Then, we searched for each of the risk factors in the GWAS catalog (www.ebi.ac.uk/gwas) to identify the associations between SNPs and the specific risk factor of interest, and any factor without related GWAS or the GWAS with incomplete information was excluded.

### Defining Genetic Instruments

The SNPs for each exposure identified by the largest GWAS in populations of European ancestry were used to conduct instrumental variables. Further details of the exposures and how we defined the genetic instruments are provided in the **Supplementary Methods** and **Supplementary Table S1**. Inclusion criteria of the SNPs as follows: (1) independent loci: defined as r^2^<0.1 based on European ancestry reference data from the 1000 Genomes Project (11, 12), and for a locus in which multiple SNPs in linkage disequilibrium, we selected the SNP with the strongest effect; (2) GWAS *p*-value threshold of <5×10^-08^, and for the SNPs of risk factor less than ten, we set GWAS-significant *p*-value threshold of <5×10^-06^; (3) having the rs numbers (or position information); (4) providing beta-coefficient (β), and standard error (SE) (or sufficient data to calculate them). After selecting the set of SNPs for each risk factor, we extracted the following information for each SNP-risk factor association: rs numbers, effect allele, other alleles, effect allele frequency, β, SE, and *p*-value. Any SNP missing the information was removed.

For the SNP(s) extracted for use in the MR-analysis, we calculated the proportion of variance explained (R^2^) in the risk factor by the SNP(s) and the strength of the instrument (F-statistic) (13). The formulas to calculate R^2^ and F-statistic were:


$${\text{R}}^{2}=[2\times \beta^{2}\times \text{MAF}\times (1-\text{MAF})]/(2\times \beta^{2}\times \text{MAF}\times [1-\text{MAF)+(SE(}\beta )]{}^{2}\times (2\times \text{N})\times \text{MAF}\times (1-\text{MAF),}$$

where β is the effect size (beta coefficient) for a given SNP, MAF is the minor allele frequency, SE(β) is the standard error of the effect size, and N is the sample size of the GWAS for the SNP-risk factor association.

$\text{F}={\text{R}}^{2}\times (\text{N}-1-\text{k})\text{/((1}-{\text{R}}^{2})\times \text{k}$ , where R^2^ is the proportion of variance explained in the risk factor by the genetic instrument, N is the sample size of the GWAS, k is the number of SNPs included in the instrument.

### Outcome Trait

GWAS results for prostate cancer were obtained from fixed-effects meta-analyses based on individuals of European ancestry in the PRACTICAL consortium (44 825 prostate cancer cases and 27 904 controls) (10), and UK Biobank (6879 prostate cancer cases and 199 891 controls) (9). We extracted the following information for each SNP of risk factor: rs numbers, effect allele, other alleles, effect allele frequency, β, SE, and *p-*value. We removed any SNP missing this information, and the one reached a *p*-value threshold of <5×10^-08^.

### Two-Sample Mendelian Randomized Analysis

The inverse variance weighted (IVW) fixed-effect method was used as the main method to estimate the effect of genetically predicted exposure on prostate cancer in our Mendelian randomization analysis. The IVW method estimates the effect of the exposure on the outcome from the slope of the relationship between bXG (SNP-exposure association) and bYG (SNP-outcome association). Casual estimates were presented as an OR and its 95% CI. OR estimates were reported per standard deviation (SD) increment for continuous variable and per log-odds increment for categorical variable in genetically determined risk of the exposures. In addition, other Mendelian randomization methods including MR-Egger, weighted median, and weighted mode method were used to check the consistency of the direction of effect estimates. We assessed horizontal pleiotropy, heterogeneity tests, funnel plots, scatter plots, and leave-one-out plots in sensitivity analyses. In addition, scatter plots of effect estimates of individual SNPs with outcome versus effect estimates of individual SNPs with exposure are provided as a comparative visual assessment of the effect estimates generated from different Mendelian randomization methods. All analyses were conducted using the package TwoSampleMR (version 0.5.6) in R (version 4.1.2).

## Results

### Exposed-Omics Analysis of the Extrinsic Exposures for Prostate Cancer

**Figure 1:**
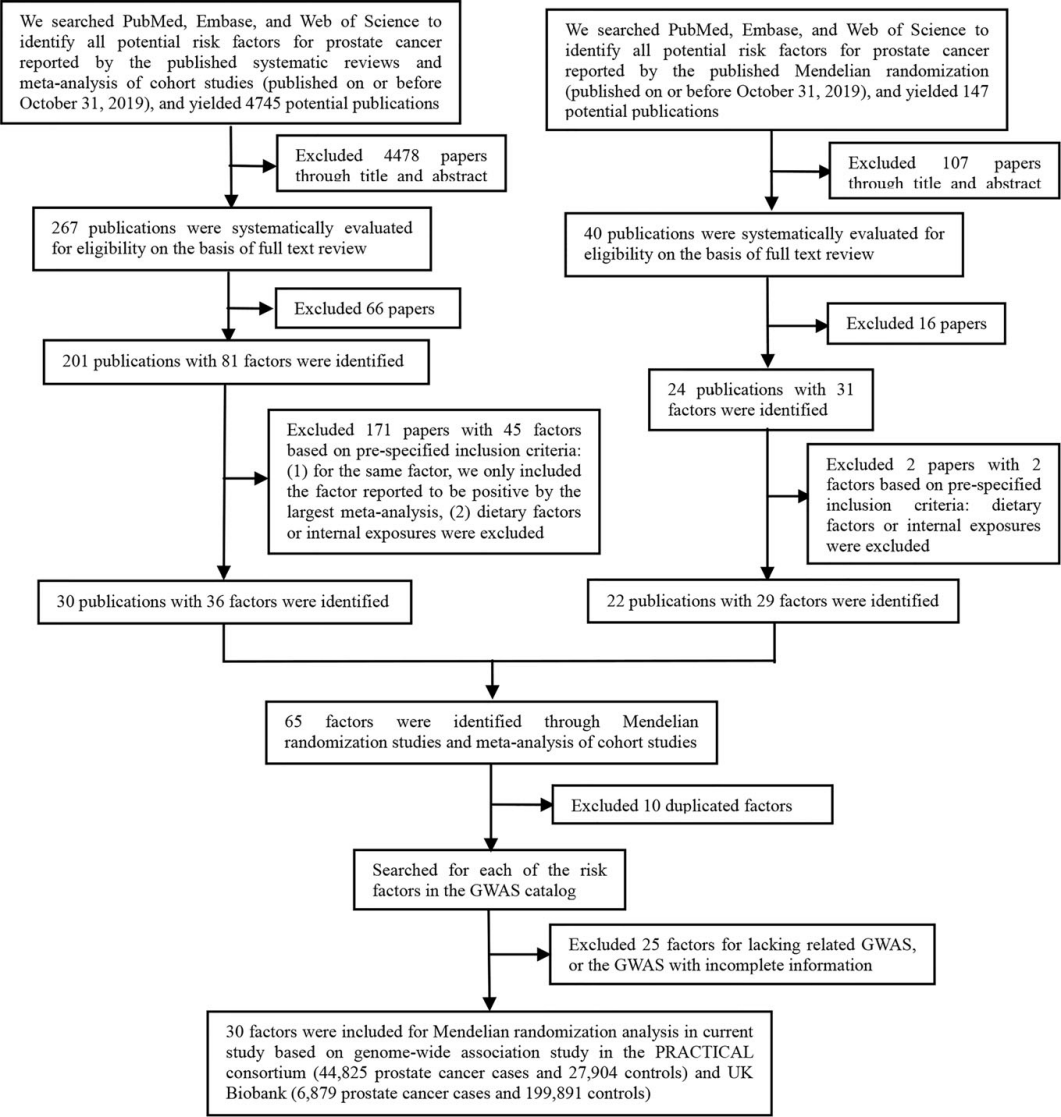
The flowchart of factors selection.

**Figure 2:**
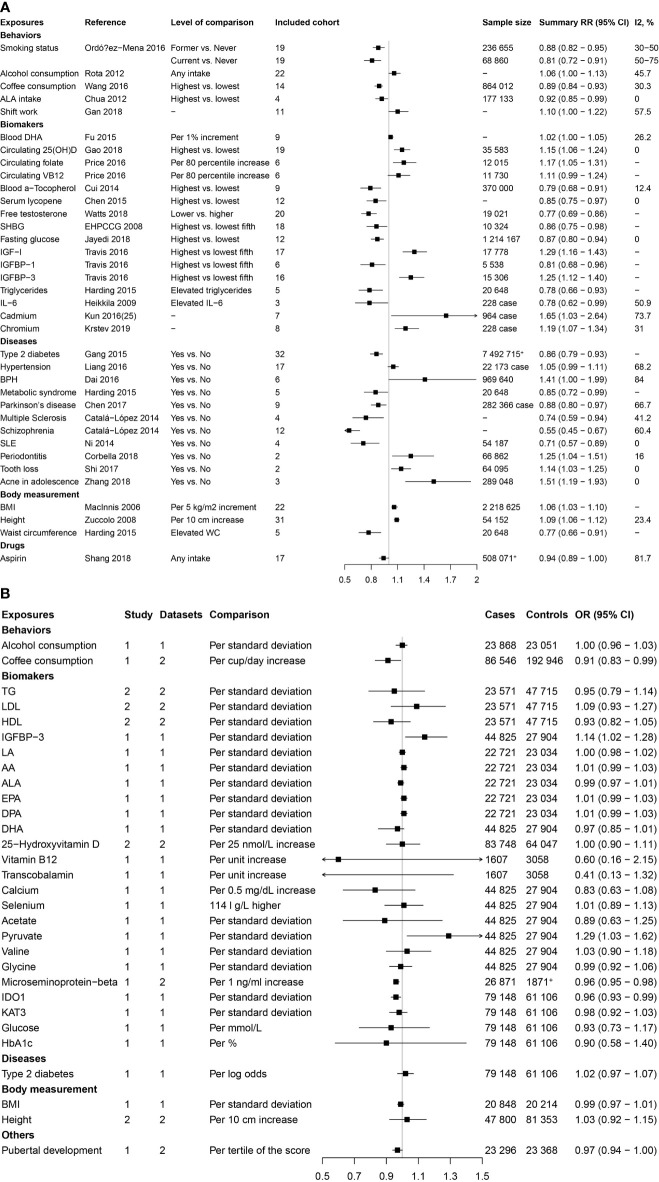
Factors of prostate cancer that were identified by the published systematic reviews and meta-analyses of cohort studies and mendelian randomization studies. **(A)** Factors identified by the published systematic reviews and meta-analyses of cohort studies. **(B)** Factors identified by the published mendelian randomization studies.

As shown in **Figure 1**, the present study conducted two parts of investigation: (1) a total of 4745 published meta-analyses of cohort studies were acquired from the PubMed, Embase, and Web of Science databases. After excluding the 4715 publications through title, abstract, and full-text reading, 30 articles including 36 factors were identified (**Figure 2** **A**). (2) Another systematic review and meta-analysis of the Mendelian randomization study incorporated 24 publications with 31 factors. The characteristics of these studies are shown in **Supplementary Table S2**. Of these studies, 18 studies outcome data source was generated from PRACTICAL consortium, two from UK-based ProtecT study, one from UK Biobank, and six from other sources (among them three studies from two sources). For these studies, eight studies involving eight factors with 140 036 cases and 279 025 controls were eligible for the meta-analysis. Results showed coffee consumption (OR=0.91, 95%CI: 0.83-0.99), microseminoprotein-beta (OR=0.96, 95%CI: 0.95-0.98), and pubertal development (OR=0.97, 95%CI: 0.94-1.00) may be causal protective factors of prostate cancer. However, we found no association of triglycerides (TG), high-density lipoprotein (HDL), low-density lipoprotein (LDL), and height with risk of prostate cancer (**Figure 2** **B**).

These two parts get 65 extrinsic exposures for prostate cancer. After excluding 10 duplicated factors and 25 factors without GWAS, a total of 30 exposures were included for Mendelian randomization analysis in the current study. Of the included 30 factors, 13 were risk factors [including alcohol consumption, blood docosahexaenoic acid (DHA), 25-Hydroxyvitamin D, circulating folate, circulating vitamin B12, Insulin-like growth factor-I (IGF-1), insulin-like growth factor binding protein 3(IGFBP-3), hypertension, benign prostatic hyperplasia (BPH), periodontitis, BMI, height, and LDL] for prostate cancer, while 17 were protective factors [including smoking status, coffee consumption, alpha-linolenic acid (ALA), free testosterone, sex hormone-binding globulin (SHBG), fasting glucose (FG), TG, HDL, Interleukin-6, Type 2 diabetes mellitus (T2DM), metabolic syndrome, Parkinson’s disease, multiple sclerosis, schizophrenia, systemic lupus erythematosus (SLE), waist circumference (WC), and aspirin intake].

### Mendelian Randomization Analysis

The genetic instruments of the selected exposures used in the Mendelian randomization analysis could explain 0.42%-54.81% of variability, and the F-statistic ranged from 8.12 to 286.33 (**Table 1**). The detailed information of variants used to conduct instrumental variables for each exposure was shown in **Supplementary Table S3**. The results of Mendelian randomization analyses are shown in **Table 2** and **Supplementary Figures S1–S4**, and the effect estimates using different MR methods are provided in **Supplementary Table S4**.

**Table 1:** Summary of genetic instruments used in the present Mendelian randomization analysis.

Extrinsic exposures	PMID	Maximum sample size	SNPs in genetic instrument	% explained variability	F statistic
Coffee consumption	31046077	375 833	13	0.55	160.69
Alcohol consumption	23743675	4915	12	4.62	19.80
Smoking status	30643258	518 633	223	1.73	40.99
25-Hydroxy vitamin D	32059762	443 734	138	8.18	286.33
Vitamin B12	19303062	3613	3	23.62	372.11
Folate	19303062	3617	1	0.42	15.26
Fasting blood glucose	20081858	128 613	33	3.3	132.9
IGF-I	21216879	10 280	4	5.93	161.99
IGFBP-3	21216879	10 280	4	1.07	27.86
TG	30275531	617 303	151	2.88	121.33
HDL	30275531	617 303	156	3.71	152.61
LDL	30275531	617 303	119	2.60	138.49
Testosterone	31169883	4291	9	6.29	35.91
SHBG	22829776	29 966	12	5.91	156.79
ALA	21829377	8866	4	3.76	86.46
DHA	21829377	8866	1	0.72	64.42
Interleukin-6	27989323	8189	23	5.99	22.64
Hypertension	31879980	185 565	35	1.14	61.20
Type 2 diabetes	30054458	659 316	149	1.54	68.89
Periodontitis	30218097	15 003	9	1.47	24.81
BPH	30988330	10 419	7	1.59	24.03
SLE	26502338	14 267	69	54.81	249.52
Schizophrenia	29483656	265 304	143	0.44	8.12
Parkinson's disease	31701892	1 474 097	106	0.6	84.38
Multiple sclerosis	31604244	115 804	192	11.48	76.13
Metabolic syndrome	31589552	291 107	93	2.53	81.11
BMI	30108127	793 208	191	1.48	62.20
Height	25282103	333 355	169	5.89	123.48
Waist circumference	28443625	362 932	51	0.69	49.36
Aspirin use	31015401	112 010	10	0.43	48.18

ALA, alpha-linolenic acid; BMI, body mass index; BPH, benign prostatic hyperplasia; SLE, systemic lupus erythematosus; DHA, docosahexaenoic acid; IGF-I, Insulin-like growth factor-I; IGFBP-3, insulin-like growth factor binding protein 3; PCa, Prostate cancer; SHBG, sex hormone binding globulin; SNP, single nucleotide polymorphism.

**Table 2:** Mendelian randomization analyses of the association between extrinsic exposures and prostate cancer risk.

Extrinsic exposures	PRACTICAL consortium			UK Biobank			Combined	
	N SNPs	OR (95%CI)	*p*	N SNPs	OR (95%CI)	*p*	OR (95%CI)	*p*
**Behaviors**								
Coffee consumption	15	0.855 (0.503-1.452)	0.561	15	0.987 (0.972-1,002)	0.081	1.005 (0.985-1.025)	0.078
Alcohol consumption (drinkers vs non-drinkers)	16	0.998 (0.994-1.003)	0.440	15	1.000 (1.000-1.000)	0.726	1.000 (1.000-1.000)	0.700
Smoking status (ever vs never smokers)	214	1.002 (0.872-1.152)	0.973	213	1.003 (0.997-1.009)	0.372	0.999 (0.999-1.000)	0.372
ALA	3	0.655 (0.176-2.442)	0.528	3	0.655 (0.176-2.442)	0.528	0.665 (0.258-1.661)	0.373
DHA	1	**0.806 (0.661-0.984)**	**0.034**	1	1.008 (0.998-1.017)	0.117	0.987 (0.972-1.002)	0.460
**Biomakers**								
Serum 25-Hydroxyvitamin D levels	106	1.012 (0.949-1.080)	0.708	85	0.999 (0.995-1.003)	0.702	0.999 (0.995-1.003)	0.719
Vitamin B12	3	1.000 (1.000-1.001)	0.081	3	1.000 (1.000-1.000)	0.272	1.000 (1.000-1.000)	0.506
Folate	1	0.991 (0.971-1.013)	0.431	1	0.999 (0.998-1.000)	0.223	0.997 (0.989-1.006)	0.210
Fasting blood glucose	32	0.927 (0.730-1.178)	0.536	32	0.997 (0.989-1.006)	0.560	0.923 (0.745-1.142)	0.546
IGF-I	4	1.000 (0.997-1.003)	0.864	4	1.000 (1.000-1.000)	0.423	1.000 (0.997-1.004)	0.416
IGFBP-3	4	**1.000 (1.000-1.000)**	**0.016**	4	1.000 (1.000-1.000)	0.743	1.000 (1.000-1.000)	0.404
Triglycerides	124	0.973 (0.886-1.068)	0.562	121	1.001 (0.997-1.005)	0.634	1.001 (0.997-1.005)	0.653
HDL	131	0.970 (0.891-1.056)	0.488	129	1.001 (0.997-1.006)	0.535	0.999 (0.998-1.000)	0.559
LDL	101	0.981 (0.888-1.084)	0.707	102	1.003 (1.000-1.007)	0.054	0.999 (0.997-1.002)	0.055
Testosterone levels	40	1.000 (0.996-1.004)	0.854	17	1.000 (0.997-1.003)	0.836	1.000 (0.998-1.003)	0.783
Sex hormone-binding globulin levels	23	0.912 (0.791-1.051)	0.202	21	1.001 (0.995-1.008)	0.659	0.970 (0.892-1.054)	0.641
Interleukin-6 levels	21	1.025 (0.951-1.104)	0.520	19	0.999 (0.997-1.002)	0.694	1.000 (1.000-1.000)	0.712
**Diseases**								
Hypertension	41	1.058 (0.994-1.187)	0.332	40	1.000 (0.996-1.004)	0.854	1.000 (0.997-1.003)	0.828
Type 2 diabetes	141	1.029 (0.967-1.095)	0.363	147	1.000 (0.998-1.002)	0.863	1.003 (0.997-1.009)	0.889
Periodontitis	110	1.006 (0.991-1.021)	0.451	105	1.001 (1.000-1.001)	0.078	1.000 (0.999-1.002)	0.073
Benign prostatic hyperplasia	7	1.027 (0.985-1.070)	0.212	5	1.000 (0.997-1.003)	0.898	0.995 (0.990-1.000)	0.647
Systemic lupus erythematosus	66	0.999 (0.984-1.013)	0.860	63	**0.999 (0.999-1.000)**	**0.039**	**0.999 (0.999-1.000)**	**0.039**
Schizophrenia	134	0.916 (0.836-1.004)	0.062	111	1.001 (0.997-1.005)	0.593	1.001 (1.000-1.001)	0.469
Parkinson’s disease	101	0.992 (0.960-1.025)	0.637	101	1.000 (0.999-1.002)	0.733	0.999 (0.997-1.001)	0.750
Multiple sclerosis	310	1.011 (0.967-1.057)	0.623	290	0.999 (0.997-1.001)	0.280	1.000 (0.997-1.003)	0.290
Metabolic syndrome	76	0.999 (0.939-1.062)	0.975	68	1.000 (0.997-1.003)	0.818	1.003 (1.000-1.007)	0.819
**Body measurement**								
Body mass index	195	1.023 (0.906-1.156)	0.711	192	**0.995 (0.990-1.000)**	**0.047**	**0.995 (0.990-1.000)**	**0.048**
Height	164	1.015 (0.950-1.086)	0.655	164	1.000 (0.997-1.003)	0.916	1.001 (0.997-1.006)	0.993
Waist circumference	49	0.913 (0.786-1.060)	0.230	48	1.000 (0.992-1.008)	0.922	0.986 (0.925-1.051)	0.665
**Drugs**								
Aspirin use measurement	9	1.108 (0.970-1.265)	0.130	10	1.006 (0.996-1.016)	0.230	1.031 (0.950-1.119)	0.466

ALA, alpha-linolenic acid; CI, confidence interval; DHA, docosahexaenoic acid; HDL, high density lipoprotein; IGF-I, Insulin-like growth factor-I; IGFBP-3, insulin-like growth factor binding protein 3; LDL, low density lipoprotein; OR, odds ratio; RACTICAL, Prostate Cancer Association Group to Investigate Cancer Associated Alterations in the Genome; SNP, single nucleotide polymorphisms.

The bold means statistical significant.

In the PRACTICAL consortium dataset, of the 30 potential extrinsic exposures examined in our study, DHA was causally associated with a decreased risk of prostate cancer (OR=0.806, 95%CI: 0.661-0.984, *p*=0.034) with the wald ratio method. Consistent with the findings in the previous meta-analysis, the conventional IVW method indicated a causal association between genetically predisposed IGFBP-3 and prostate cancer (OR=1.0002, 95%CI: 1.00004-1.0004, *p*=0.016), and weighted median methods also generated similar effect estimation (OR=1.0002, 95%CI: 1.0001-1.0004, *p*=0.0002). In addition, in the UK Biobank dataset, we found inverse associations for systemic lupus erythematosus (OR=0.9993, 95%CI: 0.9986-0.9999, *p*=0.039) and BMI (OR=0.995, 95%CI: 0.990-0.9999, *p*=0.046) with prostate cancer risk using IVW method, and weighted median methods also supported these associations. Likewise, the MR Egger method indicated a causal association between genetically predisposed SLE and prostate cancer in both PRACTICAL consortium (OR=0.96, 95%CI: 0.93-0.99, *p*=0.003) and UK Biobank (OR=0.999, 95%CI: 0.997-0.999, *p*=0.002). Besides, no causal relationship was found between other exposures and prostate cancer.

## Discussion

In the present study, we summarized the previous meta-analysis of cohort studies and performed a systematic review and meta-analysis of published Mendelian randomization studies, thus finding a total of 55 risk factors for prostate cancer. Besides, we conducted a comprehensive two-sample MR analysis to evaluate the potential causal effect of 30 extrinsic exposures on the risk of prostate cancer based on European-descent individuals in the PRACTICAL consortium and UK Biobank.

The IGF pathway plays a critical role in somatic growth and activates carcinogenic intracellular signaling networks. Published results have shown an association between circulating insulin-like growth factors (IGFs) and their binding proteins (IGFBPs) and the subsequent prostate cancer risk (14–16). Our Mendelian randomization results showed a positive association between IGFBP-3 levels and prostate cancer, as previously reported from observational and Mendelian randomization studies (15–18). IGFBP-3 is the most abundant circulating IGFBP and modulates the bioactivity of IGFs. Independent of IGFs, IGFBP-3 could regulate cell proliferation and apoptosis, leading to the carcinogenesis of certain common cancers (19, 20). Furthermore, experimental pieces of evidence suggested that IGFBP-3 might contribute to the growth and progression of prostate cancer cells (21, 22). Although our Mendelian randomization results were unable to support previous evidence of an association between genetically predicted serum IGF-1 levels and prostate cancer risk, recent published Mendelian randomization studies reported a causal association of IGF-1 levels with prostate cancer (23, 24). The inconsistency might be attributed to the proportions of advanced-stage prostate cancer cases across datasets (23).

Dietary fatty acids, especially omega-3 polyunsaturated fatty acids (ω-3 PUFAs), are one of the most intensively studied dietary factors closely related to prostate cancer risk. ω-3 PUFAs mainly include ALA, EPA, docosapentaenoic acid (DPA), and DHA. Interestingly, our study suggested an inverse association of blood DHA concentration and prostate cancer, whereas no association was observed between the genetically predicted ALA levels and prostate cancer risk. Nevertheless, ω-3 PUFAs were demonstrated to have anti-inflammatory and anti-tumor effects (25). A considerable number of studies, including both animal and *in vitro* cell studies, have indicated that ω-3 PUFAs are the most promising type of nutrients to suppress carcinogenesis and can reduce prostate cancer risk (26–28). Results from observational studies, however, have been inconsistent. Therefore, studies with larger sample sizes and longer follow-up times are warranted to confirm the results.

In the present study, we found that higher BMI was associated with a reduced prostate cancer risk, and the results were consistent with previous Mendelian randomization studies (29, 30). However, no strong evidence was found in a recent Mendelian randomization study of a causal effect of either early or later life BMI on prostate cancer (31). Besides, observational studies also reported inconsistent results since the association between BMI and prostate cancer is complex. This complex relation might be owing to the different effects of obesity on various hormones in men, such as a positive association with estrogen concentrations (32) but an inverse association with prostate-specific antigen (33). Another explanation may be the dual effect of BMI on prostate cancer. A meta-analysis of prospective studies suggested that high BMI may protect against localized prostate cancer, whereas it was a risk factor for advanced prostate cancer (34).

The relationship between SLE and cancer is also intriguing. Epidemiological evidence has suggested an increased risk of some malignancies, such as lung cancer, liver cancer, cervical cancer, and especially some hematologic cancers among patients with SLE. However, several studies found a decreased risk of some hormone-sensitive cancers, such as breast, ovarian, and endometrial cancer, in patients with SLE. Interestingly, as reported by the largest meta-analysis of the cohort study, our Mendelian randomization analysis further supported a protective effect of genetically predicted SLE on prostate cancer risk (35). However, the underlying mechanism remains unclear. Sex hormones might play a putative role in the pathogenesis of prostate cancer in males with SLE (36). As we know, androgens mediate cell proliferation in prostate tissue and are thus important in the development and progression of prostate cancer (37–40). In particular, there is some evidence that males with SLE tend to have low testosterone levels, as compared to males without SLE (41, 42), and men with low circulating free testosterone may carry a lower risk of prostate cancer (43). Further investigations are warranted.

Although our study identified several causal factors for prostate cancer, several limitations should be concerned. A total of 30 factors were included in current study, and a Bonferroni-corrected *p*-value was considered significant to address multiple testing, with a *p*-value <0.0016 being considered suggestive of an association (0.05/30 = 0.0016).

Nevertheless, we found no evidence in support of a relationship between other factors and prostate cancer risk. On the one hand, although we identify all potential risk factors for prostate cancer reported by the most recent and largest published meta-analysis of cohort studies, some meta-analyses were still limited by the small amount of literature or studies with small sample size or large heterogeneity among studies. On the other hand, the results of several exposures, such as DHA and folate, were based on one single genetic variant, which might lead to lower precision. Besides, the F-statistics for all the genetic instruments were large (>10) in our study, except for schizophrenia, indicating strong genetic instruments that are associated with the exposure. However, the percentage of variation explained was low (<3%) for most of the exposures-specific instruments, and future investigations are needed to identify additional variants to further improve the instrument strength. Considering the inconsistent results reported by previous meta-analyses of cohort studies and our research, well-designed cohort studies with larger sample sizes and Mendelian randomization analysis using more genetic variants are needed to verify these associations further.

Our study also has other limitations. First, all GWAS summary statistics used in our study were from European ancestry participants, limiting the inference of findings in other populations. Second, though our study included as many as 30 extrinsic exposures, several other important exposures, such as dietary calcium, physical activity, Cadmium, Chromium, and plasma/serum lycopene, were not included due to unavailable genetic instruments for analyses. Third, due to the lack of individual data, we were unable to test the association of genetic instruments with other confounders such as BMI, smoking, alcohol consumption, and other lifestyle-related factors. Fourth, since the data on advanced-stage prostate cancer were not available, we only investigated the associations between extrinsic exposures and the overall prostate cancer risk. Finally, although our meta-analysis suggested a positive association between IGF-1 and prostate cancer, however, the data generated from one study with three datasets and our Mendelian randomization results were unable to support this association, and therefore, more studies are required to confirm this finding.

In conclusion, we conducted a phenome-wide exposed-omics analysis and found a total of 55 factors for prostate cancer risk. The Mendelian randomization analysis verified the IGFBP-3, DHA, BMI, and SLE were causally related to prostate cancer risk. The results could help the clinicians to tailor individualized prophylactic strategies and may provide new insight into the study of the pathogenesis of prostate cancer. More Mendelian randomization studies with larger sample size and stronger power to explain the variance were needed to confirm the results further.

## Data Availability Statement

The datasets presented in this study can be found in online repositories. The names of the repository/repositories and accession number(s) can be found in the article/**Supplementary Material**.

## Ethics Statement

Our study is a secondary analysis of existing, de-identified article data, or summary-level GWAS data. Specific ethics in this study can be found in the original publications.

## Author Contributions

All authors contributed significantly to this work. BZ and DG designed the research study. DG, MT, and YW collected the data. HC, MZ, YB, ZZ, YT, and XW analyzed the data. DG wrote the first draft of the manuscript. All authors reviewed, edited, and approved the manuscript.

## Funding

This study was supported by the National Natural Science Foundation of China (81903393, and 81903398), Chongqing Natural Science Foundation Program (cstc2020jcyj-msxmX0021), and Chongqing Special Postdoctoral Science Foundation (XmT2018068). The sponsors of this study had no role in study design, data collection, analysis, interpretation, writing of the report, or the decision for submission.

## Conflict of Interest

The authors declare that the research was conducted in the absence of any commercial or financial relationships that could be construed as a potential conflict of interest.

## Publisher’s Note

All claims expressed in this article are solely those of the authors and do not necessarily represent those of their affiliated organizations, or those of the publisher, the editors and the reviewers. Any product that may be evaluated in this article, or claim that may be made by its manufacturer, is not guaranteed or endorsed by the publisher.

## Abbreviations

ALA, alpha-linolenic acid; BMI: body mass index; BPH, Benign prostatic hyperplasia; CI, confidence interval; DHA, docosahexaenoic acid; EPA, eicosapentaenoic acid; FG, fasting glucose; GWAS, genome-wide association study; HDL, high-density lipoprotein; IGF-I, Insulin-like growth factor-I; IGFBP-3, insulin-like growth factor binding protein 3; IVW, inverse variance weighted; LDL, low-density lipoprotein; MR, Mendelian randomization; OR, odds ratio; PACTICAL, Prostate Cancer Association Group to Investigate Cancer Associated Alterations in the Genome; ω-3 PUFAs, omega-3 polyunsaturated fatty acids; SHBG, sex hormone-binding globulin; SLE, systemic lupus erythematosus; SNP, single nucleotide polymorphisms; T2DM, Type 2 diabetes mellitus; WC, waist circumference.
