# Supplementary material for: The Causal Relationships Between Extrinsic Exposures and Risk of Prostate Cancer: A Phenome-Wide Mendelian Randomization Study
Source: Front Oncol. 2022 Feb 14;12:829248. doi: 10.3389/fonc.2022.829248 (PMC8882837; doi:10.3389/fonc.2022.829248)
Supplement: Supplementary file 1 [file DataSheet_1.docx]

**Supplementary Materials to:**

**The causal relationships between extrinsic exposures and risk of prostate cancer: a phenome-wide Mendelian randomization study**

Dongqing Gu^1^, Mingshuang Tang^1^, Yutong Wang^1^, Huijie Cui^2^, Min Zhang^3^, Ye Bai^3^, Ziqian Zeng^1^, Yunhua Tan^1^, Xin Wang^2^, and Ben Zhang^1^*****

**Affiliations:**

^1^ Department of Epidemiology and Biostatistics, First Affiliated Hospital, Army Medical University, Chongqing 400038, China

^2^ Department of Epidemiology and Biostatistics, West China School of Public Health and West China Fourth Hospital, Sichuan University, Chengdu, Sichuan 610041. China

^3^ School of Public Health and Management, Chongqing Medical University, Chongqing 400016, China.

***Corresponding Author**

**Ben Zhang, M.D., Ph.D.**

Department of Epidemiology and Biostatistics

First Affiliated Hospital

Army Medical University

30 Gaotanyan Street, Shapingba District, Chongqing 400038, China

Phone: 86-23-6875-4311, Fax: 86-23-6875-4311

E-mail: benzhang@vip.163.com

## Supplementary methods

## Data sources for exposures

1. **UK Biobank**

The UK Biobank (<https://www.ukbiobank.ac.uk>) is a prospective cohort study of approximately 500,000 participants aged 40 to 69 years with extensive baseline phenotypic measurements, and trait data, as well as biological samples (Sudlow et al., 2015; Galante et al., 2016).

1. **Nurses’ Health Study (NHS)**

The NHS was initiated in 1976, and including 121,700 United States registered nurses between the ages of 30 and 55 (Colditz and Hankinson, 2005). Initial questionnaire reporting medical histories and baseline health-related exposures, including information related to reproductive history, and exposure to exogenous hormones, as well as biennial questionnaires with collection of exposure information on risk factors have been collected prospectively, and outcome data with follow-up of reported disease events are collected.

1. **Framingham Heart Study (FHS)**

The FHS was first established in 1948 with the recruitment of a large cohort of adults from the town on Framingham Massachusetts (Dawber et al., 1951). Since then, three generations of participants have been studied in this ongoing community-based cohort study of cardiovascular risk.

1. **Gothenburg Osteoporosis and Obesity Determinants (GOOD) Study**

The GOOD study was initiated to determine both environmental and genetic factors involved in the regulation of bone and fat mass (Lorentzon et al., 2005). Male study subjects were randomly identified in the greater Gothenburg area in Sweden using national population registers, contacted by telephone, and invited to participate. To be enrolled in the GOOD study, subjects had to be between 18 and 20 years of age. There were no other exclusion criteria, and 49% of the study candidates agreed to participate (n = 1,068).

1. **Health, Aging, and Body Composition (Health ABC) Study**

The cohort of 3,075 healthy European-American and African-American men and women was recruited from Medicare beneficiaries in Memphis, TN and Pittsburgh, PA, USA between 1997 and 1998. At baseline, participants were between 70 and 79 years of age, 46% percent of the women were African-American, as were 37% of the men. Participants at baseline were free of functional limitation, defined as inability to walk up a flight of stairs and/or to walk a quarter of a mile without difficulty. The goal of the Health ABC Study was to understand how change in body composition and weight-related health conditions might influence incident functional limitation.

1. **Invecchiare in Chianti (InCHIANTI)**

The InCHIANTI study is a population-based epidemiological study aimed at evaluating factors that influence mobility in the older population living in the Chianti region of Tuscany, Italy. Details of the study have been previously reported (Ferrucci et al., 2000). Briefly, 1616 residents were selected from the population registry of Greve in Chianti (a rural area; 11,709 residents with 19.3% of the population greater than 65 years of age) and Bagno a Ripoli (Antella village near Florence; 4704 inhabitants, with 20.3% greater than 65 years of age). The participation rate was 90% (n= 1453) and participants ranged between 21–102 years of age. The study protocol was approved by the Italian National Institute of Research and Care of Aging Institutional Review. There were 85 parent-offspring pairs, 6 sib-pairs and 2 half-sibling pairs documented (Melzer et al., 2008). We investigated any further familial relationships using IBD of 10,000 random SNPs using RELPAIR and uncovered 1 parentoffspring, 79 siblings and 13 half-siblings.

1. **Cooperative Health Research in the Region of Augsburg (KORA)**

The KORA study is a series of independent population-based epidemiological surveys and follow-up studies of participants living in the region of Augsburg, Southern Germany. All participants are residents of German nationality identified through the registration and informed consent has been given by all participants (Wichmann et al., 2005).

1. **Multi-Ethnic Study of Atherosclerosis (MESA)**

The MESA is a study of the characteristics of subclinical cardiovascular disease (disease detected non-invasively before it has produced clinical signs and symptoms) and the risk factors that predict progression to clinically overt cardiovascular disease or progression of the subclinical disease. MESA researchers study a diverse, population-based sample of 6,814 asymptomatic men and women aged 45-84. Thirty-eight percent of the recruited participants are white, 28 percent African American, 22 percent Hispanic, and 12 percent Asian, predominantly of Chinese descent. Participants were recruited from six field centers across the United States: Wake Forest University, Columbia University, Johns Hopkins University, University of Minnesota, Northwestern University and University of California – Los Angeles (Bild et al., 2002). The data included in this study are from the European-origin subsample at the baseline examination, which was performed between 2000-2002 in six centers. MESA samples were genotyped as a part of the SNP Health Association Resource (SHARe) Project of the National Heart Lung and Blood Institute.

1. **Northern Finland Birth Cohort 1966 Study (NFBC1966)**

The study population derives from Northern Finland birth cohort 1966 (NFBC1966). In particular, mothers expected to give birth in the two Northern provinces of Oulu and Lapland in 1966 were enrolled in the NFBC1966 study (N=12,058 live births). Primary clinical data collection on parents and the child occurred prenatally and at birth. Data collection on the child continued at ages six months, one year, 14 years (no data from, six months, one year or 14 years are included in this paper), 31 years, with assessment of a wide range of trait measures. Cohort members still living in Northern Finland and those who had moved to the capital area were invited to a clinical examination at age 31 years (N=8,463). The attendees (71% response rate, N=6,007) were adequately representative of the original cohort (Jarvelin et al., 2004). Participants provided fasting blood samples for assessment of SHBG concentration that is the focus of the current study, and DNA was also extracted from the blood samples provided at this time.

1. **Rotterdam Study (RS1)**

Subjects were participants of the RS1, a large prospective population-based cohort study of Caucasian subjects aged 55 years and over, living in the Ommoord district of Rotterdam, the Netherlands. The study was designed to investigate the incidence and determinants of chronic disabling diseases in the elderly. Rationale and design have been described previously (Hofman et al., 2011). All 10,275 inhabitants aged 55 years and over were invited for baseline examination between August 1990 and June 1993. Of those, 7,983 participated. Among the subjects living independently, the overall response rate was 77 percent for home interview and 71 percent for examination in the research centre, where blood samples were taken.

1. **Study of Health in Pomerania (SHIP)**

SHIP is a longitudinal cohort study in West Pomerania, the north-east area of Germany (Völzke et al., 2011). From the entire study population of 212,157 inhabitants living in the area, a sample was selected from the population registration offices, where all German inhabitants are registered. Only individuals with German citizenship and main residency in the study area were included.

1. **TWINS UK**

The TwinsUK cohort consisted of a group of twins ascertained to study the heritability and genetics of age-related diseases (www.twinsUK.ac.uk). These unselected twins were recruited from the general population through national media campaigns in the UK and shown to be comparable to age-matched population singletons in terms of disease-related and lifestyle characteristics (Andrew et al., 2001; Spector and Williams, 2006). TwinsUK consists of twins from the adult twin British registry, also shown to be representative of singleton populations and the United Kingdom population (Andrew et al., 2001).

1. **The Cardiovascular Risk in Young Finns Study (YFS)**

The YFS is a multicenter follow-up study with randomly chosen subjects from the Finnish cities of Helsinki, Kuopio, Oulu, Tampere, and Turku and their rural surroundings (Raitakari et al., 2008). The main aim of the YFS is to determine the contribution made by childhood lifestyle, biological and psychological measures to the risk of cardiovascular diseases in adulthood. The study began in 1980, and over 3,500 children and adolescents all around Finland participated in the baseline study. The follow-up visits have been conducted mainly with 3-year intervals.

1. **Women's Health Initiative (WHI)**

Blood was collected from 27,347 study participants prior to entry into WHI randomized, placebo-controlled clinical trial of hormone therapy in post-menopausal women (Stefanick et al., 2003). The WHI hormone trial enrolled women aged 50-79 years between 1993-1998, primarily by population-based direct mail strategies, at 40 clinical centers in 24 states and the District of Columbia in the U.S.

1. **Million Veteran Program (MVP)**

The MVP is an observational cohort study and mega-biobank in the Department of Veterans Affairs (VA) health care system (Gaziano et al., 2016). Individuals aged 19 to 104 years have been recruited from more than 50 VA Medical Centers nationwide since 2011. As of August 3, 2015, approximately 397,104 veterans have been enrolled from 50 sites nationwide. Data are being collected from participants using questionnaires, the VA electronic health record, and a blood sample for genomic and other testing.

1. **LIFE-Adult**

The population-based cohort LIFE-Adult comprises 10,000 randomly selected participants from Leipzig, Germany. The primary aim of this study is to identify molecular genetics and environmental risk factors of major civilization diseases, including adiposity, vascular disease, dementia, and depression (Loeffler et al., 2015).

1. **LIFE-Heart**

The population-based cohort LIFE-Heart comprises 7000 patients with suspected or confirmed CAD were recruited from the Heart Center of Leipzig, Germany. All patients received diagnostic coronary angiography. CAD was defined as at least one stenosis of ≥50% of any major coronary vessel (Beutner et al., 2011).

1. **Prospect-European Prospective Investigation into Cancer and Nutrition (EPIC)**

The Prospect-EPIC cohort consists of 17357 white women living in Utrecht and surroundings, aged 49-70 years, who were invited to participate in the study through the national breast cancer screening program between 1993 and 1997 (Boker et al., 2001). At recruitment, each participant filled out a general questionnaire on lifestyle factors, reproductive (gynecological and obstetric) history, and past and current morbidity as well as a validated (semi)quantitative food frequency questionnaire (FFQ) aimed at capturing the habitual diet during the year preceding enrolment.

1. **Osteoporotic Fractures in Men (MrOS) Study**

The MrOS study is a multicenter, prospective study including approximately 11,014 elderly men (men aged 69-80 years) were randomly identified using national population registers in Sweden, Hong Kong, and the United States.

1. **Coronary Artery Risk Development in Young Adults (CARDIA) Women’s Study**

The CARDIA study is a multicenter longitudinal cohort study designed to investigate the development of coronary heart disease risk factors in young adults. The initial cohort consisted of 5115 young adult participants from 4 US cities (Birmingham, Alabama; Chicago, Illinois; Minneapolis, Minnesota; and Oakland, California) in 1985-1986 (Friedman et al., 1988).. Follow-up examinations were performed 2, 5, 7, 10, and 15years after baseline with retention of the cohort of 91%, 86%, 81%, 79%, and 74%, respectively. The CARDIA Women’s Study was designed to examine the associations of androgens, polycystic ovaries, and clinical features of the polycystic ovary syndrome with subclinical atherosclerosis.

1. **Genetic Investigation of ANthropometric Traits (GIANT) consortium**

GIANT (<http://www.broadinstitute.org/collaboration/giant/index.php/GIANT_consortium>) performed a meta-analysis of 51 GWAS assessing common variants associated with BMI in over 170,000 individuals of European descent. Genotyping was performed using commercially available Affymetrix or Illumina genotyping arrays or custom Perlegen arrays.

1. **Meta-Analyses of Glucose and Insulin-related traits (MAGIC) Consortium**

MAGIC was a genome wide association study (GWAS) that sought to identify genetic determinants of glycemic and metabolic traits. The association between genetic variants and the change in FG (mmol/L) was assessed in 133,010 and 42,854 non-diabetic European individuals.

1. **Global Lipids Genetic Consortium (GLGC)**

The GLGC performed a meta-analysis of 46 lipid GWAS and examined subjects of European ancestry, including 94,595 individuals from 23 studies genotyped with GWAS arrays and 93,982 individuals from 37 studies genotyped with the Metabochip array.

1. **The Electronic Medical Records and Genomics (eMERGE) Network**

The eMERGE Network is a consortium of several EHR-linked biorepositories formed with the goal of developing approaches for the use of the EHR in genomic research (Gottesman et al., 2013)

1. **Cohorts for Heart and Aging Research in Genomic Epidemiology (CHARGE) Consortium**

The CHARGE Consortium including AGES, Age, Gene/Environment Susceptibility (AGES)-Reykjavik Study; ARIC, The Atherosclerosis Risk in Communities Study; CHS, The Cardiovascular Health Study; FHS, The Framingham Heart Study; RS, the Rotterdam Study (Psaty et al., 2009).

1. **Collaborative Study on the Genetics of Alcoholism (COGA)**

COGA is a multi-site, longitudinal study established to identify vulnerability genes for AD by recruiting multiplex alcohol dependent families as well as representative families from the community (Wang et al., 2013).

1. **Genetics and Environment (SAGE)**

SAGE is funded as part of the Gene Environment Association Studies (GENEVA) initiative supported by the National Human Genome Research Institute (dbGaP study accession phs000092.v1.p1) (Bierut et al., 2010).

1. **Tobacco and Genetics (TAG) Consortium**

TAG Consortium with the largest sample yet of 74,053 individuals identified 130 SNPs (tagging the 15q25 locus) that passed the genome-wide threshold of 5×10^-8^ in the quantity smoked analysis (Tobacco and Genetics, 2010). The samples were collected mainly between 1970 and 2006, and the mean age ranged from 39.6 to 72.3.

1. **Genetic Epidemiology Research on Adult Health and Aging (GERA) Cohort**

The GERA cohort consists of 103,006 adult members of Kaiser Permanente Northern California (KPNC), ranging in age from 18 to 100 years at enrollment (Banda et al., 2015). The cohort was created to enable studies of genetic and environmental influences on many different health conditions and traits by linking high-density genome-wide SNP data with comprehensive longitudinal clinical information from electronic health records (EHR) as well as self-reported data on demographic factors and health behaviors from a structured survey.

**Supplementary Table S1 Data sources and definitions for exposures**

| **Exposures** | **Data sources** | **Definitions** |
| --- | --- | --- |
| Coffee consumption (Zhong et al., 2019) | UK Biobank, NHS, Health Professionals Follow-up Study, Women’s Genome Health Study | Any type (i.e., regular/decaf, instant/ground) |
| Alcohol consumption(Kapoor et al., 2013) | COGA **and** SAGE datasets | The measure for lifetime maximum number of alcoholic drinks consumed in 24 hour period (maxdrinks) |
| Smoking status  (Karlsson Linner et al., 2019) | UK Biobank and TAG Consortium | Ever smoker |
| ALA (Lemaitre et al., 2011) | CHARGE Consortium | Plasma ALA levels |
| DHA (Lemaitre et al., 2011) | CHARGE Consortium | Plasma DHA levels |
| 25-Hydroxy vitamin D (Manousaki et al., 2020) | UK Biobank | Blood 25(OH)D levels |
| Vitamin B12 (Tanaka et al., 2009) | InCHIANTI, SardiNIA, and BLSA studies | Serum vitamin B12 levels |
| Folate (Tanaka et al., 2009) | InCHIANTI, SardiNIA, and BLSA studies | Serum Folate levels |
| Fasting blood glucose (Dupuis et al., 2010) | MAGIC Consortium | Circulating fasting glucose levels |
| IGF-I (Kaplan et al., 2011) | CHS, FHS, KOR, and SHIP | Circulating IGF-I levels |
| IGFBP-3 (Kaplan et al., 2011) | CHS, FHS, KOR, and SHIP | Circulating IGFBP-3 levels |
| TG (Klarin et al., 2018) | GLGC, MVP | Plasma TG level |
| HDL (Klarin et al., 2018) | GLGC, MVP | Plasma HDL level |
| LDL (Klarin et al., 2018) | GLGC, MVP | Plasma LDL level |
| Testosterone31169883 | LIFE-Adult, LIFE-Heart | Testosterone levels |
| SHBG (Coviello et al., 2012) | FHS, GOOD, HABC, KORA, MESA, NFBC1966, RS-I, SHIP, TWINS UK, YFS, Women’s Health Initiative, CARDIA Women’s Study, Prospect-EPIC, MrOS, NHS, InCHIANTI | Circulating SHBG concentrations |
| Interleukin-6 (Ahola-Olli et al., 2017) | YFS, FINRISK | Circulating IL6 levels |
| Hypertension (German et al., 2020) | UK Biobank | Medical records of hypertension |
| Type 2 diabetes (Xue et al., 2018) | DIAGRAM, GERA, UK Biobank | Not reported |
| Periodontitis | ARIC, SHIP and SHIP-TREND | Chronic periodontitis |
| BPH (Hellwege et al., 2019) | eMERGE network | Among men of at least age 40, without prostate or bladder cancers we included all cases of BPH with at least two ICD9 codes indicating a BPH diagnosis (600, 600.0, 600.0*, 600.2, 600.2*, 600.9, 600.9*) |
| SLE (Bentham et al., 2015) | HRS, NIH CGEMS | Systemic lupus erythematosus (SLE; OMIM 152700) |
| Schizophrenia (Pardinas et al., 2018) | CLOZUK, PGC (Schizophrenia Working Group of the Psychiatric Genomics, 2014), CRESTAR | Schizophrenia is characterized by psychosis and negative symptoms such as social and emotional withdrawal. |
| Parkinson's disease (Nalls et al., 2019) | UK Biobank, 23andMe, IPDGC | A family history of PD or self-report of diagnosis PD |
| Multiple sclerosis (International Multiple Sclerosis Genetics, 2019) | International Multiple Sclerosis Genetics Consortium & Wellcome Trust Case Control Consortium 2 (International Multiple Sclerosis Genetics et al., 2011) | The diagnosis depends on  meeting established and well-validated criteria that combine clinical and para-clinical laboratory-based information, introduced in 1983 and revised and updated between 2001 and 2005 (Poser et al., 1983; McDonald et al., 2001; Polman et al., 2005) |
| Metabolic syndrome (Lind, 2019) | UK Biobank | Harmonized NCEP criteria (Alberti et al., 2009) |
| BMI (Hoffmann et al., 2018) | RPGEH, GERA, GIANT consortium, UK Biobank | BMI was calculated by definition: weight (kg)/height (m)2. |
| Height (Wood et al., 2014) | GIANT Consortium | Adult human height |
| Waist circumference (Justice et al., 2017) | GIANT consortium | Waist circumference adjusted for BMI |
| Aspirin use measurement (Wu et al., 2019) | UK Biobank | Self-reported regular medication of Aspirin use |

ALA, alpha-linolenic acid; BMI, body mass index; BPH, benign prostatic hyperplasia; SLE, systemic lupus erythematosus; DHA, docosahexaenoic acid; IGF-I, Insulin-like growth factor-I; IGFBP-3, insulin-like growth factor binding protein 3; SHBG, sex hormone binding globulin

**Supplementary Table S2**. **Characteristics of the published Mendelian randomization studies.**

| **Study** | **Outcome data source** | **Cases (N)** | **Controls (N)** | **Exposures** | **SNPs (N)** | **OR (95%CI)** | **Comment** |
| --- | --- | --- | --- | --- | --- | --- | --- |
| Collin 2012(Collin et al., 2011) | UK-based ProtecT study | 1607 | 3058 | Vitamin B12 | 2 | 0.60 (0.16-2.15) | Confidence intervals around the IV estimates in our study were too wide to allow robust inference |
|  |  |  |  | Transcobalamin | 1 | 0.41 (0.13-1.32) |  |
| Zhang 2015(Zhang et al., 2015) | PRACTICAL | 14 160 | 12 724 | Telomere length | 11 | 1.21 (0.99-1.46) | The weighted TL SNP score was not associated with prostate cancer risk |
| Davies 2015(Davies et al., 2015) | PRACTICAL | 20 848 | 20 214 | Height | 179 | 0.98 (0.96-1.00) | We found little evidence of a substantial effect of genetically elevated height or BMI on prostate cancer risk |
|  |  |  |  | BMI | 32 | 0.99 (0.97-1.01) |  |
| Bull 2016(Bull et al., 2016) | PRACTICAL | 22 249 | 22 133 | LDL | 10 | 1.24 (0.90-1.69) | We found weak evidence that higher LDL and TG levels increase aggressive prostate cancer risk |
|  |  |  |  | HDL | 35 | 0.99 (0.84-1.17) |  |
|  |  |  |  | TG | 14 | 1.09 (0.80-1.50) |  |
| Bonilla 2016(Bonilla et al., 2016a) | UK-based ProtecT study | 1136 | 1791 | Pubertal development | 13 | 0.95 (0.87-1.04) | Older age at sexual maturation is causally linked to a reduced risk of later prostate cancer |
|  | PRACTICAL | 22 160 | 21 577 | Pubertal development | 13 | 0.97 (0.94-1.00) |  |
| Benn 2016(Benn et al., 2016) | Danish general population | 1062 | 44 970 | BMI | 5 | 2.46 (0.34-18.00) | Results do not support causal associations |
| Bonilla 2016(Bonilla et al., 2016b) | PRACTICAL | 22 992 | 22 936 | IGF-II | 1 | 1.14 (1.00-1.31) | These findings can only causally implicate the IGF pathway in general, not any one specific biomarker |
| Khankari 2016(Khankari et al., 2016a) | PRACTICAL | 22 721 | 23 034 | LA | 4 | 1.00 (0.98-1.02) | No overall association was observed between the genetically-predicted PUFAs evaluated in this study and prostate cancer risk |
|  |  |  |  | AA | 2 | 1.01 (0.99-1.03) |  |
|  |  |  |  | ALA | 1 | 0.99 (0.97-1.01) |  |
|  |  |  |  | EPA | 2 | 1.01 (0.99-1.03) |  |
|  |  |  |  | DPA | 3 | 1.01 (0.99-1.03) |  |
|  |  |  |  | DHA | 1 | 1.00 (0.98-1.02) |  |
| Khankari 2016(Khankari et al., 2016b) | GAME-ON | 47 800 | 81 353 | Adult Height | 423 | 1.03 (0.92-1.15) | No association was observed for prostate cancer |
| Brunner 2017(Brunner et al., 2017) | PRACTICAL | 23 868 | 23 051 | Alcohol consumption | 68 | 1.00 (0.96-1.03) | Alcohol consumption is unlikely to affect prostate cancer incidence, but it may influence disease progression. |
| Taylor 2017(Taylor et al., 2017) | PRACTICAL | 22 721 | 23 034 | Coffee consumption | 2 | 1.01 (0.98-1.03 | No clear evidence that coffee consumption was associated with prostate cancer risk |
| Dimitrakopoulou 2017(Dimitrakopoulou et al., 2017) | PRACTICAL | 22 898 | 84 418 | 25(OH)D | 4 | 0.89 (0.77-1.02) | Little evidence for a linear causal association between circulating vitamin D concentration and prostate cancer risk |
| Melander 2017(Orho-Melander et al., 2018) | The Malmo Diet and Cancer Study | 1322 | 25 582 | TG | 26 | 0.89 (0.73-1.09) | There is a causal and negative association between serum TG, LDL, HDL and risk of prostate cancer |
|  |  |  |  | LDL | 32 | 1.04 (0.87-1.24) |  |
|  |  |  |  | HDL | 41 | 0.93 (0.78-1.10) |  |
| Yarmolinsky 2018(Yarmolinsky et al., 2018b) | PRACTICAL | 44 825 | 27 904 | Circulating Selenium | 11 | 1.01 (0.89-1.13) | Do not support a role for selenium supplementation in prostate cancer prevention |
| Tan 2018(Tan et al., 2018) | PRACTICAL | 44 825 | 27 904 | IGFBP-3 | 4 | 1.14 (1.02-1.28) | Members of the IGF pathway, in particular IGFBP-3, has a causal effect on prostate cancer and advanced prostate cancer |
| GoÂmez-Acebo 2018(Gomez-Acebo et al., 2018) | MCC-Spain | 817 | 1006 | Pigmentation phototype | 17 | 1.01 (0.97–1.05) | Phototype is not associated with prostate cancer |
| Yarmolinsky 2018(Yarmolinsky et al., 2018a) | PRACTICAL | 44 825 | 27 904 | Serum calcium | 5 | 0.83 (0.63-1.08) | Ds not support the hypothesis that serum calcium increases risk of overall or advanced prostate cancer |
| Beynon 2019(Beynon et al., 2019) | PRACTICAL | 44 825 | 27 904 | Acetate | 1 | 0.89 (0.63-1.25) | Lycopene lowered levels of pyruvate, which suggests may be causally related to reduced prostate cancer risk |
|  |  |  |  | Pyruvate | 2 | 1.29 (1.03-1.62) |  |
|  |  |  |  | Valine | 4 | 1.03 (0.90-1.18) |  |
|  |  |  |  | DHA | 4 | 0.97 (0.85-1.01) |  |
|  |  |  |  | Glycine | 6 | 0.99 (0.92-1.06) |  |
| Jiang 2019(Jiang et al., 2019) | PRACTICAL | 79 148 | 61 106 | 25(OH)D | 6 | 1.00 (0.93-1.07) | Do not support a causal effect of circulating 25(OH)D concentrations on prostate cancer risk |
| Byrne 2019(Smith Byrne et al., 2019) | EPIC | 1871 | 1871 | MSP | 1 | 0.97 (0.95-0.99) | MSP may play a causally protective role in prostate cancer |
|  | PRACTICAL | 25 000 | - | MSP | 1 | 0.96 (0.95-0.98) |  |
| Li 2019(Li et al., 2019) | PRACTICAL | 79 148 | 61 106 | IDO1 | 4 | 0.96 (0.93-0.99) | IDO1 might be a potential therapeutic target for prostate cancer |
|  |  |  |  | KAT3 | 2 | 0.98 (0.92-1.03) |  |
| Ong 2019(Ong et al., 2019) | UK Biobank | 7352 | 131 834 | Coffee consumption | 35 | 0.86 (0.75-0.98) | Do not support a strong causal relationship between coffee and risk of prostate cancer |
|  | PRACTICAL | 79 194 | 61 112 |  |  | 0.96 (0.84-1.08) |  |
| Zhang 2019(Zhang et al., 2020) | Two sources | 4600 | 2941 | 25(OH)D | 3 | 1.15 (0.87-1.52) | Do not support a causal effect of vitamin D on the development of prostate cancer |
| Yeung 2019(Au Yeung and Schooling, 2019) | PRACTICAL | 79 148 | 61 106 | Glucose | 11 | 0.93 (0.73-1.17) | There was no association of fasting glucose, HbA1c, or type 2 diabetes with prostate cancer |
|  |  |  |  | HbA1c | 19 | 0.90 (0.58-1.40) |  |
|  |  |  |  | Type 2 diabetes | 112 | 1.02 (0.97-1.07) |  |

AA: Arachidonic acid, ALA: a-Linolenic acid, DHA: Docosahexaenoic acid, EPA: Eicosapentaenoic acid, EPIC: European Prospective Investigation into Cancer and Nutrition, LA: Linoleic acid, MSP: Microseminoprotein, PRACTICAL: Prostate Cancer Association Group to Investigate Cancer Associated Alterations in the Genome, 25(OH)D: 25-hydroxyvitamin D

**Supplementary Table S3. The SNPs used to conduct instrumental variable for each exposure**

| **SNP** | **EA** | **OA** | **EAF** | **BETA** | **SE** | ***P*** | **Chr** | **Position** |
| --- | --- | --- | --- | --- | --- | --- | --- | --- |
| **ALA** | | | | | | | | |
| rs16832011 | A | G | 0.9899 | 0.0155 | 0.003 | 3.00E-07 | 2 | 135787729 |
| rs4985167 | T | C | 0.2525 | 0.0059 | 0.001 | 3.00E-07 | 16 | 14989008 |
| rs1692120 | A | G | 0.4242 | -0.0051 | 0.001 | 1.00E-08 | 11 | 61650000 |
| rs174547 | T | C | 0.6414 | -0.0159 | 0.001 | 3.00E-64 | 11 | 61803311 |
| **Alcohol consumption** | | | | | | | | |
| rs1353899 | G | T | 0.22 | -0.09 | 0.02 | 4.00E-06 | 3 | 177511191 |
| rs16985179 | T | C | 0.11 | -0.12 | 0.032 | 6.00E-06 | 22 | 27633571 |
| rs2188561 | G | T | 0.21 | -0.08 | 0.02 | 9.00E-06 | 7 | 107695613 |
| rs36061340 | T | C | 0.05 | -0.15 | 0.03 | 7.00E-06 | 8 | 100795002 |
| rs4543123 | G | A | 0.24 | 0.08 | 0.02 | 9.00E-06 | 4 | 38790903 |
| rs4758317 | A | C | 0.42 | -0.08 | 0.02 | 7.00E-07 | 11 | 8229264 |
| rs59677118 | A | G | 0.06 | 0.14 | 0.03 | 1.00E-06 | 9 | 14441679 |
| rs59972978 | T | A | 0.2 | -0.09 | 0.02 | 5.00E-06 | 20 | 57483299 |
| rs62202398 | A | G | 0.06 | -0.15 | 0.03 | 9.00E-06 | 20 | 48172481 |
| rs66595363 | A | G | 0.09 | -0.13 | 0.03 | 9.00E-06 | 7 | 20089751 |
| rs7144649 | G | A | 0.23 | 0.09 | 0.02 | 4.00E-06 | 14 | 57355498 |
| rs7553212 | G | A | 0.32 | 0.07 | 0.2 | 7.00E-06 | 1 | 216565446 |
| **Aspirin use** | | | | | | | | |
| rs583104 | G | T | 0.22 | -0.08 | 0.009 | 2.00E-16 | 1 | 109278685 |
| rs73015016 | A | G | 0.12 | -0.1 | 0.012 | 6.00E-15 | 19 | 11080624 |
| rs635634 | T | C | 0.18 | 0.07 | 0.01 | 7.00E-11 | 9 | 133279427 |
| rs2523589 | T | G | 0.5 | 0.04 | 0.008 | 2.00E-08 | 6 | 31359557 |
| rs7412 | T | C | 0.08 | -0.11 | 0.015 | 5.00E-14 | 19 | 44908822 |
| rs28601761 | G | C | 0.42 | -0.06 | 0.008 | 1.00E-13 | 8 | 125487789 |
| rs74617384 | T | A | 0.08 | 0.11 | 0.014 | 3.00E-13 | 6 | 160576086 |
| rs140570886 | C | T | 0.02 | 0.22 | 0.032 | 2.00E-12 | 6 | 160591981 |
| rs1831733 | C | T | 0.48 | 0.05 | 0.008 | 9.00E-09 | 9 | 22076072 |
| rs964184 | G | C | 0.13 | 0.06 | 0.012 | 5.00E-08 | 11 | 116778201 |
| **BMI** | | | | | | | | |
| rs10019997 | T | C | 0.4091 | 0.015 | 0.002 | 7.00E-21 | 4 | 136127444 |
| rs10063334 | C | T | 0.1616 | 0.013 | 0.002 | 1.00E-08 | 5 | 113425177 |
| rs10132280 | C | A | 0.6566 | 0.022 | 0.003 | 6.00E-15 | 14 | 25458973 |
| rs10182181 | A | G | 0.5 | -0.031 | 0.003 | 3.00E-35 | 2 | 24927427 |
| rs10460960 | G | A | 0.096 | -0.025 | 0.004 | 1.00E-10 | 3 | 42267243 |
| rs10733682 | A | G | 0.5051 | 0.019 | 0.003 | 8.00E-13 | 9 | 126698635 |
| rs10742752 | C | T | 0.601 | 0.013 | 0.002 | 6.00E-16 | 11 | 45416824 |
| rs1074657 | T | C | 0.2677 | -0.014 | 0.002 | 2.00E-14 | 1 | 243583332 |
| rs10760279 | G | T | 0.5808 | -0.02 | 0.003 | 4.00E-10 | 9 | 123343012 |
| rs10840100 | A | G | 0.3283 | -0.018 | 0.003 | 5.00E-12 | 11 | 8647890 |
| rs10920678 | A | G | 0.3636 | 0.017 | 0.003 | 7.00E-11 | 1 | 190270777 |
| rs10929925 | C | A | 0.5909 | 0.014 | 0.002 | 2.00E-08 | 2 | 6015425 |
| rs10938397 | A | G | 0.5758 | -0.037 | 0.003 | 1.00E-47 | 4 | 45180510 |
| rs10968576 | A | G | 0.6616 | -0.025 | 0.003 | 7.00E-21 | 9 | 28414341 |
| rs11057405 | G | A | 0.9141 | 0.029 | 0.005 | 3.00E-10 | 12 | 122297350 |
| rs11074446 | T | C | 0.8485 | 0.025 | 0.004 | 6.00E-12 | 16 | 20243801 |
| rs11081818 | A | G | 0.4242 | 0.014 | 0.002 | 1.00E-15 | 18 | 33671124 |
| rs11126666 | G | A | 0.7273 | -0.019 | 0.003 | 5.00E-11 | 2 | 26705943 |
| rs11165643 | C | T | 0.4242 | -0.019 | 0.003 | 3.00E-13 | 1 | 96458541 |
| rs11170468 | A | C | 0.8081 | -0.014 | 0.002 | 8.00E-13 | 12 | 39036246 |
| rs11185092 | A | G | 0.7475 | -0.019 | 0.004 | 6.00E-08 | 1 | 107343656 |
| rs11189513 | A | G | 0.7172 | 0.016 | 0.003 | 6.00E-08 | 10 | 98209811 |
| rs11191560 | T | C | 0.9394 | -0.033 | 0.004 | 8.00E-15 | 10 | 103109281 |
| rs11583200 | C | T | 0.3687 | 0.019 | 0.003 | 4.00E-13 | 1 | 50094148 |
| rs11611246 | G | T | 0.8384 | -0.022 | 0.003 | 2.00E-12 | 12 | 830314 |
| rs1167827 | A | G | 0.4091 | -0.019 | 0.003 | 3.00E-12 | 7 | 75533848 |
| rs11679338 | C | T | 0.3283 | -0.02 | 0.003 | 2.00E-11 | 2 | 180742168 |
| rs11688816 | G | A | 0.4646 | 0.014 | 0.002 | 1.00E-08 | 2 | 62825913 |
| rs11727676 | T | C | 0.9394 | 0.029 | 0.005 | 7.00E-09 | 4 | 144737912 |
| rs118067556 | C | T | 0.9697 | -0.035 | 0.005 | 1.00E-13 | 10 | 61376407 |
| rs11847697 | C | T | 0.9747 | -0.042 | 0.006 | 9.00E-11 | 14 | 30045906 |
| rs12042908 | A | G | 0.4848 | 0.022 | 0.003 | 1.00E-17 | 1 | 74532078 |
| rs12286929 | A | G | 0.5404 | -0.021 | 0.003 | 6.00E-16 | 11 | 115151684 |
| rs12325113 | T | C | 0.6364 | -0.029 | 0.003 | 4.00E-29 | 16 | 28837347 |
| rs12352785 | A | C | 0.3434 | -0.013 | 0.002 | 6.00E-13 | 9 | 6956850 |
| rs12429545 | G | A | 0.8788 | -0.032 | 0.004 | 5.00E-18 | 13 | 53528071 |
| rs12446632 | G | A | 0.8434 | 0.034 | 0.004 | 9.00E-20 | 16 | 19924067 |
| rs12602912 | C | T | 0.7727 | -0.017 | 0.003 | 5.00E-08 | 17 | 67873957 |
| rs12770228 | G | A | 0.6768 | -0.018 | 0.003 | 6.00E-09 | 10 | 21494705 |
| rs12885454 | C | A | 0.6515 | 0.02 | 0.003 | 4.00E-14 | 14 | 29267632 |
| rs12899850 | C | T | 0.8131 | -0.014 | 0.002 | 2.00E-10 | 15 | 65758961 |
| rs12939549 | A | G | 0.5505 | 0.017 | 0.003 | 3.00E-11 | 17 | 80637924 |
| rs12961799 | C | T | 0.2929 | 0.011 | 0.002 | 3.00E-09 | 18 | 12904400 |
| rs13021737 | A | G | 0.1667 | -0.058 | 0.003 | 4.00E-69 | 2 | 632348 |
| rs13076366 | C | A | 0.7121 | 0.019 | 0.003 | 2.00E-08 | 3 | 53713347 |
| rs13078960 | T | G | 0.7879 | -0.027 | 0.003 | 4.00E-17 | 3 | 85758440 |
| rs13107325 | C | T | 0.904 | -0.051 | 0.005 | 3.00E-21 | 4 | 102267552 |
| rs1316982 | A | T | 0.8389 | -0.029 | 0.005 | 3.00E-09 | X | 118753835 |
| rs13191362 | A | G | 0.8788 | 0.025 | 0.004 | 2.00E-10 | 6 | 162612318 |
| rs1396141 | T | C | 0.6263 | 0.013 | 0.002 | 7.00E-13 | 2 | 41446605 |
| rs1405552 | G | A | 0.5455 | 0.015 | 0.002 | 1.00E-09 | 12 | 41352871 |
| rs1436351 | T | G | 0.7424 | 0.016 | 0.002 | 2.00E-17 | 3 | 104899129 |
| rs1441264 | G | A | 0.4091 | -0.018 | 0.003 | 2.00E-12 | 13 | 79006784 |
| rs1460676 | T | C | 0.8182 | -0.018 | 0.003 | 4.00E-08 | 2 | 163711179 |
| rs1558902 | T | A | 0.5455 | -0.079 | 0.003 | 2.00E-210 | 16 | 53769662 |
| rs1561589 | G | A | 0.6212 | -0.017 | 0.003 | 7.00E-10 | 10 | 125007104 |
| rs1582931 | G | A | 0.5253 | 0.015 | 0.003 | 9.00E-08 | 5 | 123321505 |
| rs16851483 | G | T | 0.904 | -0.03 | 0.006 | 5.00E-08 | 3 | 141556594 |
| rs16903285 | T | C | 0.8485 | -0.023 | 0.004 | 1.00E-08 | 5 | 88682435 |
| rs16907751 | C | T | 0.899 | 0.029 | 0.005 | 1.00E-09 | 8 | 80463222 |
| rs16996700 | T | C | 0.697 | 0.02 | 0.003 | 2.00E-12 | 20 | 52365406 |
| rs17016673 | C | G | 0.9242 | -0.028 | 0.005 | 5.00E-10 | 2 | 79270653 |
| rs17024393 | T | C | 0.9545 | -0.053 | 0.007 | 6.00E-13 | 1 | 109612066 |
| rs17094222 | T | C | 0.8232 | -0.025 | 0.003 | 6.00E-15 | 10 | 100635683 |
| rs17203016 | A | G | 0.7727 | -0.02 | 0.003 | 1.00E-09 | 2 | 207390794 |
| rs1720825 | A | G | 0.202 | 0.018 | 0.003 | 9.00E-09 | 3 | 138389241 |
| rs17381664 | T | C | 0.6364 | -0.021 | 0.003 | 3.00E-15 | 1 | 77582646 |
| rs17405819 | T | C | 0.7222 | 0.025 | 0.003 | 0.000 | 8 | 75894349 |
| rs17522122 | G | T | 0.5101 | -0.018 | 0.003 | 8.00E-12 | 14 | 32833676 |
| rs17630235 | G | A | 0.6414 | 0.016 | 0.003 | 5.00E-10 | 12 | 112153882 |
| rs17759796 | C | A | 0.8636 | 0.014 | 0.002 | 7.00E-10 | 22 | 21835874 |
| rs1788820 | A | G | 0.2828 | 0.02 | 0.003 | 4.00E-12 | 18 | 23521980 |
| rs1801282 | C | G | 0.904 | -0.023 | 0.004 | 2.00E-09 | 3 | 12351626 |
| rs1808192 | A | G | 0.3586 | 0.016 | 0.003 | 6.00E-09 | 17 | 47717340 |
| rs1816537 | A | C | 0.4545 | -0.018 | 0.003 | 4.00E-10 | 11 | 113097929 |
| rs1819844 | A | G | 0.1717 | -0.016 | 0.002 | 2.00E-14 | 12 | 67811824 |
| rs1928295 | T | C | 0.5606 | 0.016 | 0.002 | 1.00E-10 | 9 | 117616205 |
| rs2033529 | A | G | 0.7172 | -0.018 | 0.003 | 2.00E-11 | 6 | 40380914 |
| rs2044148 | G | A | 0.803 | 0.02 | 0.003 | 3.00E-10 | 2 | 24514964 |
| rs206936 | A | G | 0.8131 | -0.019 | 0.003 | 4.00E-10 | 6 | 34335092 |
| rs2075650 | A | G | 0.8333 | 0.027 | 0.004 | 1.00E-13 | 19 | 44892362 |
| rs2080454 | C | A | 0.3535 | 0.015 | 0.003 | 8.00E-09 | 16 | 49028679 |
| rs2112347 | T | G | 0.6667 | 0.027 | 0.003 | 3.00E-26 | 5 | 75719417 |
| rs2145272 | G | A | 0.4141 | -0.018 | 0.003 | 6.00E-11 | 20 | 6645571 |
| rs215607 | G | A | 0.2071 | 0.024 | 0.004 | 2.00E-10 | 7 | 32298725 |
| rs2163188 | G | C | 0.5051 | -0.015 | 0.003 | 4.00E-08 | 10 | 63554951 |
| rs2185027 | A | C | 0.7222 | -0.015 | 0.003 | 5.00E-08 | 6 | 153060487 |
| rs2243930 | G | A | 0.8232 | -0.017 | 0.003 | 6.00E-08 | 20 | 55572404 |
| rs2245368 | C | T | 0.2424 | 0.025 | 0.004 | 5.00E-09 | 7 | 76978826 |
| rs2270204 | T | G | 0.7222 | -0.018 | 0.003 | 5.00E-09 | 9 | 128280455 |
| rs2287019 | C | T | 0.8384 | 0.031 | 0.003 | 1.00E-19 | 19 | 45698914 |
| rs2321882 | G | C | 0.7475 | -0.024 | 0.004 | 8.00E-12 | 13 | 58877855 |
| rs2357760 | G | A | 0.3232 | -0.015 | 0.003 | 6.00E-08 | 6 | 119892734 |
| rs2365389 | C | T | 0.6667 | 0.02 | 0.003 | 3.00E-15 | 3 | 61250788 |
| rs2371767 | G | C | 0.7424 | -0.016 | 0.003 | 3.00E-08 | 3 | 64732582 |
| rs2372716 | C | T | 0.2323 | -0.015 | 0.002 | 3.00E-15 | 12 | 99179648 |
| rs2481665 | T | C | 0.5253 | 0.018 | 0.003 | 1.00E-12 | 1 | 62129005 |
| rs2494114 | C | G | 0.6364 | -0.027 | 0.003 | 3.00E-20 | 1 | 201831883 |
| rs261967 | A | C | 0.5657 | 0.017 | 0.003 | 1.00E-09 | 5 | 96514546 |
| rs2759315 | C | A | 0.5404 | -0.018 | 0.003 | 5.00E-11 | 15 | 80717305 |
| rs2836754 | T | C | 0.399 | -0.017 | 0.003 | 7.00E-11 | 21 | 38919816 |
| rs28573110 | A | G | 0.601 | 0.019 | 0.003 | 3.00E-12 | 17 | 36558525 |
| rs2890652 | T | C | 0.8485 | -0.024 | 0.004 | 4.00E-10 | 2 | 142202362 |
| rs29941 | A | G | 0.7136 | -0.016 | 0.003 | 3.00E-09 | 19 | 33818627 |
| rs3026101 | T | C | 0.7424 | -0.02 | 0.003 | 3.00E-13 | 17 | 5377145 |
| rs306890 | C | T | 0.2778 | 0.036 | 0.004 | 6.00E-20 | X | 155757485 |
| rs3101336 | T | C | 0.3636 | -0.032 | 0.003 | 7.00E-36 | 1 | 72285502 |
| rs3127553 | G | A | 0.3232 | 0.021 | 0.003 | 3.00E-16 | 1 | 48972333 |
| rs34811474 | G | A | 0.8131 | 0.02 | 0.003 | 2.00E-09 | 4 | 25407216 |
| rs35423729 | C | CG | 0.3691 | -0.025 | 0.004 | 1.00E-10 | X | 53521162 |
| rs3783890 | T | C | 0.8333 | 0.018 | 0.003 | 1.00E-08 | 14 | 93323930 |
| rs3810291 | G | A | 0.3182 | -0.026 | 0.003 | 8.00E-19 | 19 | 47065746 |
| rs3817334 | C | T | 0.596 | -0.023 | 0.003 | 6.00E-20 | 11 | 47629441 |
| rs4072096 | A | C | 0.5202 | -0.015 | 0.003 | 3.00E-09 | 2 | 226171887 |
| rs4130548 | T | C | 0.6566 | -0.022 | 0.003 | 2.00E-16 | 1 | 77998184 |
| rs4238331 | T | G | 0.2222 | -0.011 | 0.002 | 5.00E-09 | 15 | 58719934 |
| rs4671328 | T | G | 0.4394 | 0.024 | 0.003 | 5.00E-18 | 2 | 58708147 |
| rs4715210 | C | T | 0.8333 | -0.04 | 0.003 | 2.00E-34 | 6 | 50929538 |
| rs4755726 | T | G | 0.3131 | 0.018 | 0.003 | 1.00E-10 | 11 | 43620580 |
| rs4776970 | A | T | 0.6364 | 0.026 | 0.003 | 4.00E-24 | 15 | 67788548 |
| rs4820408 | T | G | 0.4293 | 0.016 | 0.003 | 3.00E-10 | 22 | 40208941 |
| **BPH** | | | | | | | | |
| rs141179786 | G | A | 0.02 | 0.916 | 0.187 | 9.00E-07 | 18 | 53662174 |
| rs2710383 | G | C | 0.88 | 0.371 | 0.074 | 5.00E-07 | 22 | 32554983 |
| rs4239633 | C | T | 0.68 | 0.236 | 0.047 | 6.00E-07 | 19 | 17631660 |
| rs11793639 | C | T | 0.77 | 0.211 | 0.044 | 2.00E-06 | 9 | 111968704 |
| rs6078585 | T | C | 0.55 | 0.182 | 0.038 | 2.00E-06 | 20 | 12447612 |
| rs10786938 | T | G | 0.71 | 0.207 | 0.041 | 4.00E-07 | 10 | 106280012 |
| rs534957 | C | G | 0.35 | 0.215 | 0.047 | 5.00E-06 | 6 | 53541553 |
| **Coffee consumption** | | | | | | | | |
| rs574367 | T | G | 0.21 | 0.0104 | 0.002 | 8.06E-09 | 1 | 177873210 |
| rs10865548 | G | A | 0.83 | 0.0153 | 0.002 | 4.46E-15 | 2 | 631606 |
| rs1260326 | C | T | 0.61 | 0.0135 | 0.002 | 2.62E-19 | 2 | 27730940 |
| rs117692895 | C | G | 0.01 | 0.042 | 0.007 | 4.13E-10 | 7 | 172776925 |
| rs4410790 | C | T | 0.63 | 0.0386 | 0.002 | 5.59E-141 | 7 | 17284577 |
| rs73073176 | C | T | 0.87 | 0.0228 | 0.002 | 5.56E-25 | 7 | 17562952 |
| rs34060476 | G | A | 0.13 | 0.0187 | 0.002 | 5.06E-18 | 7 | 73037956 |
| rs1057868 | T | C | 0.29 | 0.0195 | 0.002 | 5.26E-33 | 7 | 75615006 |
| rs597045 | A | T | 0.69 | 0.0106 | 0.002 | 6.62E-11 | 11 | 56272114 |
| rs1956218 | G | A | 0.56 | 0.0082 | 0.002 | 3.62E-08 | 14 | 33075243 |
| rs2472297 | T | C | 0.27 | 0.0444 | 0.002 | 5.19E-155 | 15 | 75027880 |
| rs66723169 | A | C | 0.23 | 0.0146 | 0.002 | 9.88E-17 | 18 | 57808978 |
| rs2330783 | G | T | 0.99 | 0.0443 | 0.006 | 1.57E-12 | 22 | 24747031 |
| **DHA** | | | | | | | | |
| rs2236212 | C | G | 0.3791 | -0.11 | 0.013704 | 1.00E-15 | 6 | 10994782 |
| **FG** | | | | | | | | |
| rs16913693 | T | G | 0.9732 | 0.0430 | 0.0070 | 3.51E-11 | 9 | 110720180 |
| rs6113722 | G | A | 0.9572 | 0.0350 | 0.0050 | 2.49E-11 | 20 | 22505099 |
| rs11195502 | C | T | 0.9129 | 0.0320 | 0.0040 | 1.97E-18 | 10 | 113029657 |
| rs1280 | T | C | 0.8642 | 0.0260 | 0.0030 | 8.56E-18 | 3 | 172195984 |
| rs11603334 | G | A | 0.8331 | 0.0190 | 0.0030 | 1.12E-11 | 11 | 72110633 |
| rs10811661 | T | C | 0.8204 | 0.0240 | 0.0030 | 5.65E-18 | 9 | 22124094 |
| rs11708067 | A | G | 0.7897 | 0.0230 | 0.0030 | 1.30E-18 | 3 | 124548468 |
| rs3783347 | G | T | 0.7890 | 0.0170 | 0.0030 | 1.32E-10 | 14 | 99909014 |
| rs11039182 | T | C | 0.7299 | 0.0230 | 0.0020 | 4.82E-22 | 11 | 47303299 |
| rs3829109 | G | A | 0.7066 | 0.0170 | 0.0030 | 1.13E-10 | 9 | 138376587 |
| rs560887 | C | T | 0.7043 | 0.0710 | 0.0020 | 1.40E-178 | 2 | 169471394 |
| rs4869272 | T | C | 0.6886 | 0.0180 | 0.0020 | 1.02E-15 | 5 | 95565204 |
| rs11558471 | A | G | 0.6809 | 0.0290 | 0.0020 | 7.80E-37 | 8 | 118254914 |
| rs11715915 | C | T | 0.6755 | 0.0120 | 0.0020 | 4.90E-08 | 3 | 49430334 |
| rs10747083 | A | G | 0.6630 | 0.0130 | 0.0020 | 7.57E-09 | 12 | 131551691 |
| rs174576 | C | A | 0.6520 | 0.0200 | 0.0020 | 1.18E-18 | 11 | 61360086 |
| rs780094 | C | T | 0.6095 | 0.0270 | 0.0020 | 2.58E-37 | 2 | 27594741 |
| rs4502156 | T | C | 0.5524 | 0.0220 | 0.0020 | 1.38E-25 | 15 | 60170447 |
| rs2191349 | T | G | 0.5261 | 0.0290 | 0.0020 | 1.28E-42 | 7 | 15030834 |
| rs340874 | C | T | 0.5209 | 0.0130 | 0.0020 | 4.08E-10 | 1 | 212225879 |
| rs10814916 | C | A | 0.5083 | 0.0160 | 0.0020 | 2.26E-13 | 9 | 4283150 |
| rs2302593 | C | G | 0.5033 | 0.0140 | 0.0020 | 9.26E-10 | 19 | 50888474 |
| rs11607883 | G | A | 0.4823 | 0.0210 | 0.0020 | 6.32E-24 | 11 | 45796285 |
| rs6943153 | T | C | 0.3352 | 0.0150 | 0.0020 | 1.63E-12 | 7 | 50759073 |
| rs7651090 | G | A | 0.3063 | 0.0130 | 0.0020 | 1.75E-08 | 3 | 186996086 |
| rs10830963 | G | C | 0.2897 | 0.0780 | 0.0020 | 1.07E-215 | 11 | 92348358 |
| rs9368222 | A | C | 0.2834 | 0.0140 | 0.0020 | 1.00E-09 | 6 | 20794975 |
| rs7903146 | T | C | 0.2797 | 0.0220 | 0.0020 | 2.71E-20 | 10 | 114748339 |
| rs11619319 | G | A | 0.2265 | 0.0200 | 0.0020 | 1.33E-15 | 13 | 27385599 |
| rs6072275 | A | G | 0.1647 | 0.0160 | 0.0030 | 1.66E-08 | 20 | 39177319 |
| rs2908289 | A | G | 0.1628 | 0.0570 | 0.0030 | 3.32E-88 | 7 | 44190467 |
| rs576674 | G | A | 0.1544 | 0.0170 | 0.0030 | 2.26E-08 | 13 | 32452302 |
| rs983309 | T | G | 0.1170 | 0.0260 | 0.0030 | 6.29E-15 | 8 | 9215142 |
| **Folate** | | | | | | | | |
| rs153734 | T | C | 0.84 | 1.237874 | 0.316764 | 7.00E-06 | 3 | 64053049 |
| **HDL** | | | | | | | | |
| rs139271800 | A | G | 0.9992 | -0.243 | 0.037 | 3.00E-11 | 15 | 89671546 |
| rs41278045 | A | G | 0.9986 | 0.156 | 0.028 | 2.00E-08 | 4 | 109717608 |
| rs77960347 | A | G | 0.9878 | -0.233 | 0.013 | 3.00E-73 | 18 | 49583585 |
| rs139097404 | T | C | 0.9746 | 0.099 | 0.009 | 9.00E-26 | 15 | 43641743 |
| rs10507274 | T | C | 0.9391 | -0.023 | 0.004 | 2.00E-08 | 12 | 116723171 |
| rs61743199 | A | G | 0.933 | 0.034 | 0.006 | 2.00E-09 | 19 | 49657834 |
| rs17173637 | T | C | 0.9156 | 0.029 | 0.005 | 9.00E-10 | 7 | 150832361 |
| rs3211938 | T | G | 0.9109 | -0.122 | 0.011 | 3.00E-27 | 7 | 80671133 |
| rs17369400 | A | G | 0.9053 | 0.021 | 0.004 | 7.00E-09 | 4 | 153302896 |
| rs5471 | A | C | 0.9039 | -0.074 | 0.011 | 2.00E-11 | 16 | 72054562 |
| rs79407615 | T | G | 0.9025 | -0.18 | 0.005 | 3.00E-293 | 8 | 19992588 |
| rs686030 | A | C | 0.8696 | 0.043 | 0.004 | 1.00E-27 | 9 | 15304784 |
| rs2044753 | T | C | 0.8682 | 0.021 | 0.004 | 9.00E-09 | 3 | 24251510 |
| rs429358 | T | C | 0.8416 | 0.093 | 0.004 | 1.00E-142 | 19 | 44908684 |
| rs151105710 | D | I | 0.8089 | -0.026 | 0.004 | 2.00E-13 | 3 | 136406837 |
| rs267738 | T | G | 0.8083 | -0.025 | 0.004 | 6.00E-12 | 1 | 150968149 |
| rs380267 | A | G | 0.805 | -0.062 | 0.004 | 4.00E-71 | 19 | 54295230 |
| rs11045171 | A | G | 0.8048 | -0.032 | 0.004 | 2.00E-18 | 12 | 20317265 |
| rs35240997 | A | G | 0.7924 | -0.026 | 0.004 | 1.00E-13 | 3 | 12337852 |
| rs2268840 | T | C | 0.7876 | -0.022 | 0.003 | 2.00E-10 | 3 | 186213385 |
| rs562306828 | D | I | 0.7873 | 0.04 | 0.003 | 1.00E-33 | 20 | 45928577 |
| rs1117816 | A | C | 0.78 | -0.021 | 0.003 | 2.00E-10 | 4 | 68483300 |
| rs3773910 | C | G | 0.7604 | -0.016 | 0.003 | 1.00E-10 | 3 | 152454081 |
| rs689183 | T | G | 0.7461 | -0.018 | 0.003 | 4.00E-09 | 11 | 110141418 |
| rs7730898 | A | G | 0.7431 | -0.018 | 0.002 | 2.00E-14 | 5 | 171032671 |
| rs4749779 | A | G | 0.7382 | -0.016 | 0.003 | 4.00E-09 | 10 | 8534243 |
| rs6120815 | T | C | 0.7246 | -0.015 | 0.003 | 2.00E-08 | 20 | 31597063 |
| rs2792735 | A | G | 0.7206 | -0.031 | 0.003 | 6.00E-21 | 10 | 112162067 |
| rs2281279 | T | C | 0.7201 | -0.018 | 0.003 | 1.00E-12 | 20 | 47661506 |
| rs2954038 | A | C | 0.7182 | 0.041 | 0.003 | 1.00E-41 | 8 | 125495147 |
| rs6971365 | T | C | 0.7147 | 0.026 | 0.003 | 2.00E-18 | 7 | 130747722 |
| rs28499105 | A | G | 0.708 | -0.017 | 0.003 | 5.00E-09 | 5 | 53978637 |
| rs11706108 | T | C | 0.7059 | -0.026 | 0.003 | 2.00E-14 | 3 | 52338350 |
| rs7444 | T | C | 0.7049 | 0.034 | 0.003 | 1.00E-28 | 22 | 21622645 |
| rs2434612 | A | G | 0.7005 | 0.022 | 0.003 | 2.00E-18 | 5 | 158595033 |
| rs61805076 | T | C | 0.6945 | 0.025 | 0.003 | 1.00E-16 | 1 | 182185855 |
| rs821840 | A | G | 0.6902 | -0.221 | 0.003 | 1.00E-300 | 16 | 56959974 |
| rs6710091 | C | G | 0.686 | -0.017 | 0.003 | 5.00E-08 | 2 | 239597 |
| rs2373459 | T | C | 0.6796 | 0.015 | 0.002 | 9.00E-12 | 12 | 101480178 |
| rs10911505 | T | C | 0.673 | 0.013 | 0.002 | 1.00E-08 | 1 | 184080844 |
| rs6720034 | A | G | 0.6594 | 0.015 | 0.002 | 2.00E-09 | 2 | 110907179 |
| rs10842708 | A | G | 0.6564 | 0.018 | 0.002 | 1.00E-14 | 12 | 26321934 |
| rs10773112 | T | C | 0.6429 | 0.039 | 0.003 | 8.00E-43 | 12 | 124853983 |
| rs856404 | A | G | 0.6427 | -0.013 | 0.002 | 3.00E-09 | 20 | 52647247 |
| rs10963012 | C | G | 0.6314 | -0.036 | 0.007 | 3.00E-08 | 9 | 17295543 |
| rs13379043 | T | C | 0.6293 | -0.017 | 0.003 | 1.00E-08 | 14 | 73783423 |
| rs6486121 | T | C | 0.6199 | -0.015 | 0.002 | 1.00E-10 | 11 | 13334223 |
| rs2721954 | T | C | 0.609 | 0.031 | 0.003 | 1.00E-26 | 8 | 115590876 |
| rs12601079 | A | G | 0.6042 | 0.033 | 0.003 | 8.00E-35 | 17 | 78404248 |
| rs1519480 | T | C | 0.5935 | 0.014 | 0.002 | 6.00E-10 | 11 | 27654165 |
| rs2489629 | T | C | 0.5744 | -0.018 | 0.003 | 6.00E-11 | 6 | 127155572 |
| rs9368830 | T | C | 0.574 | 0.028 | 0.003 | 4.00E-21 | 6 | 34700858 |
| rs11434755 | D | I | 0.5737 | 0.015 | 0.003 | 2.00E-08 | 11 | 18045474 |
| rs4551851 | A | G | 0.571 | -0.015 | 0.003 | 3.00E-09 | 12 | 33306619 |
| rs1945391 | A | T | 0.5525 | -0.023 | 0.003 | 2.00E-16 | 11 | 122649583 |
| rs199607859 | T | G | 0.548 | 0.02 | 0.003 | 7.00E-14 | 6 | 139514281 |
| rs2899297 | A | G | 0.5479 | -0.023 | 0.003 | 1.00E-16 | 22 | 38198661 |
| rs2307111 | T | C | 0.5474 | -0.023 | 0.003 | 3.00E-16 | 5 | 75707853 |
| rs5835988 | D | I | 0.5473 | -0.03 | 0.003 | 2.00E-26 | 2 | 164645418 |
| rs4846914 | A | G | 0.5456 | 0.041 | 0.003 | 4.00E-48 | 1 | 230159944 |
| rs2494748 | T | C | 0.5435 | -0.03 | 0.003 | 3.00E-26 | 14 | 104792555 |
| rs4766578 | A | T | 0.5425 | 0.022 | 0.003 | 1.00E-14 | 12 | 111466567 |
| rs34940240 | D | I | 0.5407 | 0.019 | 0.003 | 4.00E-13 | 19 | 33449757 |
| rs6808104 | A | G | 0.534 | 0.016 | 0.003 | 7.00E-09 | 3 | 48730444 |
| rs3173615 | C | G | 0.5291 | 0.012 | 0.002 | 2.00E-09 | 7 | 12229791 |
| rs6925103 | T | C | 0.5094 | -0.012 | 0.002 | 4.00E-10 | 6 | 136754872 |
| rs3809114 | A | G | 0.5061 | 0.018 | 0.003 | 7.00E-11 | 12 | 57454856 |
| rs111439884 | A | C | 0.5043 | 0.022 | 0.003 | 3.00E-16 | 3 | 49986605 |
| rs1788783 | T | C | 0.504 | 0.016 | 0.002 | 8.00E-14 | 18 | 23581170 |
| rs55703462 | A | G | 0.5027 | -0.019 | 0.003 | 3.00E-09 | 15 | 63103229 |
| rs823114 | A | G | 0.4983 | 0.015 | 0.002 | 5.00E-12 | 1 | 205750404 |
| rs235314 | T | C | 0.4952 | -0.017 | 0.003 | 4.00E-10 | 21 | 44851537 |
| rs4077194 | T | G | 0.4801 | -0.019 | 0.003 | 9.00E-12 | 1 | 178564697 |
| rs6777217 | A | G | 0.4746 | -0.013 | 0.002 | 3.00E-12 | 3 | 36937551 |
| rs13133548 | A | G | 0.4745 | -0.022 | 0.003 | 2.00E-16 | 4 | 88818977 |
| rs1917368 | T | G | 0.465 | -0.028 | 0.003 | 2.00E-24 | 7 | 17872129 |
| rs56325564 | A | G | 0.4569 | 0.015 | 0.003 | 4.00E-08 | 17 | 47689405 |
| rs4655268 | C | G | 0.4562 | 0.017 | 0.003 | 2.00E-09 | 1 | 214819637 |
| rs998584 | A | C | 0.4455 | -0.037 | 0.003 | 5.00E-40 | 6 | 43790159 |
| rs71468663 | T | C | 0.4369 | 0.024 | 0.003 | 2.00E-15 | 11 | 64250633 |
| rs10874777 | T | C | 0.4369 | 0.024 | 0.003 | 7.00E-18 | 1 | 93396463 |
| rs1408579 | T | C | 0.4351 | 0.019 | 0.003 | 7.00E-11 | 10 | 100152437 |
| rs13396091 | A | G | 0.4308 | -0.013 | 0.002 | 1.00E-09 | 2 | 145614393 |
| rs7954144 | A | G | 0.4301 | -0.027 | 0.003 | 8.00E-24 | 12 | 109578088 |
| rs34879232 | D | I | 0.4208 | 0.016 | 0.003 | 2.00E-08 | 17 | 28395021 |
| rs4691380 | T | C | 0.4071 | 0.0160 | 0.003 | 1.00E-08 | 4 | 156798972 |
| rs2245477 | A | C | 0.4047 | -0.014 | 0.002 | 1.00E-08 | 15 | 61656236 |
| rs2066152 | A | G | 0.4025 | 0.021 | 0.003 | 9.00E-14 | 1 | 219490688 |
| rs11078917 | A | C | 0.3803 | -0.034 | 0.003 | 3.00E-30 | 17 | 39590106 |
| rs2280334 | T | C | 0.3791 | -0.012 | 0.002 | 2.00E-09 | 2 | 66438014 |
| rs9817452 | T | G | 0.3746 | 0.028 | 0.003 | 7.00E-22 | 3 | 157077625 |
| rs3751812 | T | G | 0.3619 | -0.025 | 0.003 | 2.00E-18 | 16 | 53784548 |
| rs2834707 | T | C | 0.3612 | -0.015 | 0.003 | 8.00E-10 | 21 | 34971255 |
| rs2203452 | A | G | 0.3482 | 0.042 | 0.003 | 9.00E-52 | 2 | 226230042 |
| rs68148663 | D | I | 0.3422 | -0.02 | 0.003 | 9.00E-12 | 1 | 62687529 |
| rs77250403 | D | I | 0.3343 | 0.091 | 0.003 | 6.00E-216 | 15 | 58381251 |
| rs4722593 | A | G | 0.3238 | 0.021 | 0.003 | 4.00E-11 | 7 | 26330570 |
| rs4149307 | T | C | 0.3233 | 0.069 | 0.003 | 3.00E-93 | 9 | 104827463 |
| rs61676547 | C | G | 0.3144 | -0.021 | 0.003 | 3.00E-11 | 17 | 67896391 |
| rs174537 | T | G | 0.3123 | -0.031 | 0.003 | 2.00E-22 | 11 | 61785208 |
| rs2878349 | A | G | 0.3106 | -0.014 | 0.002 | 1.00E-08 | 1 | 107006623 |
| rs2936512 | T | C | 0.3019 | -0.014 | 0.002 | 9.00E-10 | 8 | 6741484 |
| rs12975319 | A | G | 0.3003 | -0.014 | 0.002 | 1.00E-09 | 19 | 3414090 |
| rs459193 | A | G | 0.2967 | 0.029 | 0.003 | 2.00E-23 | 5 | 56510924 |
| rs2925979 | T | C | 0.2955 | -0.031 | 0.003 | 6.00E-27 | 16 | 81501185 |
| rs1045241 | T | C | 0.2937 | 0.014 | 0.002 | 9.00E-09 | 5 | 119393591 |
| rs17615494 | T | G | 0.2881 | -0.017 | 0.003 | 1.00E-10 | 2 | 58723228 |
| rs56149994 | T | C | 0.2862 | -0.023 | 0.003 | 1.00E-13 | 19 | 7242250 |
| rs6728523 | C | G | 0.2803 | 0.024 | 0.003 | 2.00E-15 | 2 | 65055574 |
| rs72926946 | A | C | 0.2784 | -0.023 | 0.003 | 6.00E-14 | 2 | 202613145 |
| rs2642438 | A | G | 0.2715 | -0.022 | 0.003 | 6.00E-13 | 1 | 220796686 |
| rs6881956 | A | G | 0.2605 | -0.016 | 0.003 | 3.00E-09 | 5 | 73630689 |
| rs553682607 | D | I | 0.2484 | 0.027 | 0.003 | 1.00E-15 | 10 | 45564986 |
| rs2294915 | T | C | 0.246 | -0.018 | 0.003 | 3.00E-08 | 22 | 43945024 |
| rs79949326 | T | C | 0.2327 | 0.024 | 0.003 | 3.00E-13 | 7 | 6421679 |
| rs34696509 | D | I | 0.2128 | -0.032 | 0.004 | 2.00E-13 | 11 | 75841535 |
| rs3832016 | D | I | 0.2119 | 0.042 | 0.004 | 4.00E-29 | 1 | 109275537 |
| rs4130023 | T | C | 0.2084 | -0.015 | 0.003 | 2.00E-08 | 6 | 41966776 |
| rs676210 | A | G | 0.2052 | 0.055 | 0.003 | 5.00E-63 | 2 | 21008652 |
| rs10713774 | D | I | 0.188 | -0.022 | 0.004 | 1.00E-10 | 4 | 26048829 |
| rs2306363 | T | G | 0.1834 | 0.023 | 0.004 | 5.00E-10 | 11 | 65638129 |
| rs12938449 | A | C | 0.1814 | -0.018 | 0.003 | 1.00E-08 | 17 | 583581 |
| rs55747707 | A | G | 0.1796 | 0.031 | 0.004 | 6.00E-19 | 7 | 73623036 |
| rs3768321 | T | G | 0.1737 | -0.051 | 0.004 | 1.00E-36 | 1 | 39570256 |
| rs62246406 | A | G | 0.1629 | -0.024 | 0.004 | 5.00E-10 | 3 | 47056495 |
| rs3746428 | A | G | 0.1592 | -0.024 | 0.004 | 3.00E-10 | 20 | 35131380 |
| rs78123380 | A | G | 0.1585 | 0.047 | 0.008 | 3.00E-09 | 8 | 143215145 |
| rs8142788 | A | G | 0.1559 | -0.023 | 0.004 | 1.00E-08 | 22 | 29004527 |
| rs56070533 | A | G | 0.1512 | 0.078 | 0.004 | 9.00E-95 | 16 | 67908417 |
| rs4850047 | T | C | 0.1359 | 0.019 | 0.003 | 5.00E-10 | 2 | 3587163 |
| rs74256604 | A | G | 0.1298 | -0.028 | 0.004 | 3.00E-11 | 19 | 51800816 |
| rs2278426 | T | C | 0.1249 | -0.076 | 0.005 | 1.00E-52 | 19 | 11239812 |
| rs75393320 | C | G | 0.1212 | 0.065 | 0.005 | 8.00E-45 | 11 | 47244920 |
| rs72823013 | A | G | 0.1124 | 0.027 | 0.005 | 4.00E-09 | 10 | 114026474 |
| rs4841132 | A | G | 0.1054 | -0.095 | 0.004 | 1.00E-105 | 8 | 9326086 |
| rs10504474 | A | G | 0.1043 | -0.028 | 0.004 | 2.00E-14 | 8 | 70348225 |
| rs12509976 | T | C | 0.0995 | 0.039 | 0.006 | 8.00E-13 | 4 | 99596167 |
| rs11248051 | T | C | 0.0929 | -0.018 | 0.003 | 2.00E-08 | 4 | 864544 |
| rs4759375 | T | C | 0.0919 | 0.049 | 0.005 | 2.00E-24 | 12 | 123311691 |
| rs11751347 | T | C | 0.0857 | -0.064 | 0.005 | 2.00E-32 | 6 | 160671406 |
| rs13107325 | T | C | 0.0765 | -0.08 | 0.005 | 2.00E-50 | 4 | 102267552 |
| rs10479024 | A | C | 0.0726 | 0.038 | 0.006 | 1.00E-10 | 5 | 133131681 |
| rs10494363 | A | G | 0.0686 | 0.03 | 0.005 | 4.00E-10 | 1 | 149937602 |
| rs200513066 | D | I | 0.0509 | -0.089 | 0.01 | 5.00E-21 | 2 | 218856230 |
| rs16958751 | A | G | 0.0376 | 0.051 | 0.008 | 3.00E-10 | 16 | 69323503 |
| rs1126930 | C | G | 0.0326 | -0.037 | 0.006 | 3.00E-11 | 12 | 49005349 |
| rs1800961 | T | C | 0.0303 | -0.141 | 0.008 | 2.00E-65 | 20 | 44413724 |
| rs72836561 | T | C | 0.029 | -0.199 | 0.008 | 1.00E-123 | 17 | 43848758 |
| rs148149124 | D | I | 0.0281 | -0.102 | 0.01 | 6.00E-26 | 15 | 43382233 |
| rs79598313 | T | C | 0.0216 | -0.09 | 0.01 | 3.00E-19 | 1 | 26958422 |
| rs2901286 | A | C | 0.0199 | 0.044 | 0.008 | 4.00E-09 | 10 | 121141109 |
| rs116843064 | A | G | 0.0191 | 0.258 | 0.01 | 3.00E-137 | 19 | 8364439 |
| rs56121005 | T | C | 0.0129 | -0.105 | 0.017 | 4.00E-10 | 19 | 11304030 |
| rs138326449 | A | G | 0.0033 | 0.729 | 0.03 | 5.00E-134 | 11 | 116830638 |
| rs150090666 | T | C | 8.00E-04 | 0.399 | 0.049 | 3.00E-16 | 11 | 14843853 |
| rs77375493 | T | G | 5.00E-04 | -0.558 | 0.061 | 1.00E-19 | 9 | 5073770 |
| **Height** | | | | | | | | |
| rs9993613 | T | G | 0.473 | 0.03 | 0.003 | 5.00E-24 | 4 | 72610297 |
| rs994533 | C | G | 0.331 | -0.027 | 0.003 | 1.00E-17 | 2 | 217419555 |
| rs991967 | A | C | 0.717 | -0.034 | 0.003 | 2.00E-26 | 1 | 218442109 |
| rs9880211 | A | G | 0.248 | -0.03 | 0.003 | 2.00E-18 | 3 | 136388707 |
| rs9835332 | C | G | 0.46 | -0.028 | 0.003 | 4.00E-22 | 3 | 56633654 |
| rs975210 | A | G | 0.184 | 0.035 | 0.004 | 1.00E-17 | 15 | 70072013 |
| rs9650315 | T | G | 0.133 | -0.061 | 0.005 | 2.00E-41 | 8 | 56243039 |
| rs9434723 | A | G | 0.155 | 0.029 | 0.004 | 9.00E-13 | 1 | 9232223 |
| rs9428104 | A | G | 0.247 | -0.043 | 0.003 | 3.00E-36 | 1 | 118312964 |
| rs9392918 | T | C | 0.525 | -0.038 | 0.003 | 4.00E-38 | 6 | 7708398 |
| rs9292468 | T | C | 0.399 | 0.036 | 0.003 | 2.00E-33 | 5 | 32818967 |
| rs9217 | T | C | 0.63 | -0.028 | 0.003 | 5.00E-20 | 17 | 7459769 |
| rs897080 | T | C | 0.743 | -0.028 | 0.003 | 2.00E-16 | 2 | 44547063 |
| rs891088 | A | G | 0.738 | -0.029 | 0.003 | 7.00E-18 | 19 | 7184751 |
| rs8756 | C | A | 0.508 | -0.059 | 0.003 | 5.00E-90 | 12 | 65965972 |
| rs862034 | A | G | 0.361 | -0.028 | 0.003 | 6.00E-20 | 14 | 74524043 |
| rs822531 | T | C | 0.776 | 0.036 | 0.004 | 2.00E-18 | 7 | 148932667 |
| rs817300 | A | G | 0.071 | -0.085 | 0.007 | 4.00E-34 | 9 | 95617940 |
| rs8102380 | A | G | 0.686 | -0.02 | 0.003 | 8.00E-11 | 19 | 10690509 |
| rs806794 | A | G | 0.71 | 0.06 | 0.003 | 5.00E-74 | 6 | 26200449 |
| rs8067165 | C | G | 0.403 | -0.023 | 0.003 | 7.00E-12 | 17 | 8128618 |
| rs798497 | A | G | 0.698 | 0.057 | 0.003 | 2.00E-71 | 7 | 2756323 |
| rs7980687 | A | G | 0.205 | 0.039 | 0.004 | 1.00E-26 | 12 | 123338164 |
| rs7870753 | A | G | 0.777 | -0.043 | 0.004 | 4.00E-33 | 9 | 96439303 |
| rs7849585 | T | G | 0.331 | 0.036 | 0.003 | 1.00E-29 | 9 | 136220024 |
| rs780094 | T | C | 0.386 | -0.021 | 0.003 | 6.00E-12 | 2 | 27518370 |
| rs7740107 | A | T | 0.737 | -0.042 | 0.003 | 3.00E-36 | 6 | 130053316 |
| rs7731703 | T | C | 0.318 | -0.03 | 0.003 | 4.00E-18 | 5 | 32694836 |
| rs7716219 | T | C | 0.307 | 0.03 | 0.003 | 7.00E-22 | 5 | 55659243 |
| rs7692995 | T | C | 0.848 | 0.074 | 0.004 | 1.00E-71 | 4 | 17935011 |
| rs7652177 | C | G | 0.49 | -0.038 | 0.003 | 3.00E-39 | 3 | 172251287 |
| rs763318 | G | A | 0.468 | -0.021 | 0.003 | 8.00E-13 | 4 | 12961950 |
| rs7568069 | A | G | 0.578 | -0.022 | 0.003 | 3.00E-13 | 2 | 71357355 |
| rs7551732 | A | T | 0.608 | 0.027 | 0.003 | 6.00E-20 | 1 | 88673358 |
| rs749052 | T | C | 0.938 | 0.067 | 0.006 | 2.00E-26 | 2 | 231931900 |
| rs7466269 | A | G | 0.644 | 0.033 | 0.003 | 1.00E-27 | 9 | 130588697 |
| rs7319045 | A | G | 0.392 | 0.024 | 0.003 | 8.00E-15 | 13 | 91372320 |
| rs7273787 | A | G | 0.651 | -0.022 | 0.003 | 3.00E-12 | 20 | 4117920 |
| rs724016 | A | G | 0.555 | -0.078 | 0.003 | 3.00E-158 | 3 | 141386728 |
| rs720390 | A | G | 0.383 | 0.035 | 0.003 | 1.00E-29 | 3 | 185830895 |
| rs7162542 | C | G | 0.447 | -0.046 | 0.003 | 8.00E-55 | 15 | 83845538 |
| rs7154721 | T | C | 0.573 | 0.027 | 0.003 | 5.00E-20 | 14 | 91961004 |
| rs7112925 | T | C | 0.356 | -0.024 | 0.003 | 6.00E-15 | 11 | 67058689 |
| rs711245 | A | G | 0.332 | -0.024 | 0.003 | 4.00E-14 | 2 | 36541732 |
| rs7043114 | T | C | 0.56 | -0.029 | 0.003 | 2.00E-22 | 9 | 92625701 |
| rs6974574 | A | T | 0.313 | -0.03 | 0.003 | 1.00E-18 | 7 | 38070471 |
| rs6920372 | A | G | 0.413 | -0.025 | 0.003 | 2.00E-17 | 6 | 109402736 |
| rs6919534 | A | G | 0.869 | 0.05 | 0.004 | 8.00E-31 | 6 | 35279126 |
| rs6894139 | T | G | 0.562 | 0.03 | 0.003 | 6.00E-24 | 5 | 89031965 |
| rs6887276 | C | G | 0.546 | -0.018 | 0.003 | 1.00E-09 | 5 | 128042602 |
| rs6714546 | A | G | 0.279 | -0.03 | 0.003 | 2.00E-18 | 2 | 33136358 |
| rs6696239 | A | G | 0.187 | -0.038 | 0.004 | 7.00E-24 | 1 | 227562367 |
| rs6694089 | A | G | 0.279 | 0.039 | 0.003 | 4.00E-33 | 1 | 172114741 |
| rs6600365 | T | C | 0.569 | -0.027 | 0.003 | 2.00E-20 | 1 | 41090581 |
| rs6563199 | T | C | 0.352 | 0.018 | 0.003 | 1.00E-08 | 13 | 80976314 |
| rs648831 | T | C | 0.503 | 0.031 | 0.003 | 3.00E-26 | 6 | 80246491 |
| rs6485978 | T | C | 0.544 | -0.023 | 0.003 | 1.00E-15 | 11 | 12656868 |
| rs6457374 | T | C | 0.73 | -0.041 | 0.003 | 8.00E-35 | 6 | 31304484 |
| rs6446315 | A | G | 0.833 | -0.028 | 0.004 | 4.00E-11 | 4 | 5033860 |
| rs6439168 | A | G | 0.212 | -0.037 | 0.004 | 8.00E-25 | 3 | 129332100 |
| rs6137287 | T | C | 0.309 | 0.02 | 0.003 | 4.00E-10 | 20 | 21199620 |
| rs606452 | A | C | 0.142 | 0.043 | 0.004 | 2.00E-23 | 11 | 75565133 |
| rs5742915 | T | C | 0.535 | -0.035 | 0.003 | 2.00E-29 | 15 | 74044292 |
| rs552707 | T | C | 0.305 | 0.046 | 0.003 | 9.00E-46 | 7 | 28165684 |
| rs509035 | A | G | 0.316 | 0.031 | 0.003 | 3.00E-23 | 3 | 172445659 |
| rs4986172 | T | C | 0.346 | -0.034 | 0.003 | 8.00E-27 | 17 | 45138914 |
| rs4896582 | A | G | 0.299 | -0.051 | 0.003 | 3.00E-55 | 6 | 142382740 |
| rs4868126 | T | G | 0.395 | -0.036 | 0.003 | 3.00E-29 | 5 | 171856465 |
| rs4803468 | A | G | 0.419 | 0.03 | 0.003 | 2.00E-21 | 19 | 41416447 |
| rs4735677 | A | T | 0.716 | -0.037 | 0.003 | 6.00E-30 | 8 | 77235955 |
| rs4733724 | A | G | 0.802 | 0.05 | 0.004 | 1.00E-41 | 8 | 129711482 |
| rs4620037 | A | C | 0.796 | 0.032 | 0.004 | 1.00E-18 | 5 | 171448093 |
| rs4448343 | A | G | 0.654 | -0.035 | 0.003 | 5.00E-30 | 9 | 95504088 |
| rs4369779 | T | C | 0.207 | -0.056 | 0.004 | 2.00E-53 | 18 | 23155444 |
| rs4344931 | A | C | 0.29 | -0.02 | 0.003 | 8.00E-10 | 2 | 240879110 |
| rs4332428 | A | G | 0.879 | 0.036 | 0.005 | 2.00E-15 | 10 | 4923242 |
| rs4240326 | A | G | 0.464 | 0.04 | 0.003 | 3.00E-43 | 4 | 144918112 |
| rs422421 | T | C | 0.218 | -0.034 | 0.004 | 2.00E-20 | 5 | 177090325 |
| rs42039 | T | C | 0.266 | 0.068 | 0.003 | 4.00E-88 | 7 | 92615108 |
| rs4141885 | A | T | 0.918 | 0.073 | 0.008 | 8.00E-22 | 6 | 26157253 |
| rs4072910 | C | G | 0.442 | -0.032 | 0.004 | 1.00E-18 | 19 | 8579147 |
| rs3825199 | A | G | 0.773 | -0.051 | 0.004 | 4.00E-49 | 12 | 93583178 |
| rs3814333 | T | C | 0.323 | 0.049 | 0.003 | 5.00E-51 | 1 | 184037985 |
| rs3791679 | A | G | 0.766 | 0.06 | 0.004 | 2.00E-67 | 2 | 55869757 |
| rs3767627 | T | C | 0.844 | 0.036 | 0.004 | 4.00E-19 | 1 | 149966973 |
| rs3760318 | A | G | 0.374 | -0.041 | 0.003 | 3.00E-41 | 17 | 30920697 |
| rs3739707 | A | C | 0.247 | -0.024 | 0.004 | 4.00E-12 | 9 | 111030426 |
| rs318095 | T | C | 0.463 | 0.024 | 0.003 | 2.00E-16 | 17 | 48897372 |
| rs314263 | T | C | 0.676 | -0.043 | 0.003 | 1.00E-42 | 6 | 104944870 |
| rs3118905 | A | G | 0.281 | -0.058 | 0.003 | 1.00E-69 | 13 | 50531198 |
| rs310421 | T | G | 0.537 | 0.032 | 0.003 | 3.00E-27 | 6 | 81082346 |
| rs3020418 | A | G | 0.296 | 0.032 | 0.003 | 8.00E-24 | 6 | 152024027 |
| rs301901 | A | G | 0.565 | 0.024 | 0.003 | 4.00E-16 | 5 | 37046524 |
| rs2974438 | A | G | 0.203 | -0.037 | 0.004 | 4.00E-24 | 5 | 168823898 |
| rs2857693 | T | G | 0.38 | -0.034 | 0.003 | 3.00E-29 | 6 | 31620607 |
| rs2856321 | A | G | 0.639 | -0.031 | 0.003 | 8.00E-24 | 12 | 11702839 |
| rs2854207 | C | G | 0.727 | -0.046 | 0.003 | 1.00E-42 | 17 | 63869747 |
| rs2806561 | A | G | 0.567 | 0.027 | 0.003 | 2.00E-20 | 1 | 23178302 |
| rs2687950 | T | C | 0.254 | 0.036 | 0.003 | 9.00E-27 | 13 | 50144332 |
| rs2573625 | T | C | 0.672 | 0.029 | 0.003 | 6.00E-20 | 15 | 99972953 |
| rs2413143 | T | C | 0.159 | -0.034 | 0.004 | 2.00E-17 | 22 | 32660873 |
| rs2378870 | T | C | 0.372 | 0.02 | 0.003 | 4.00E-10 | 17 | 61561262 |
| rs2326458 | A | C | 0.751 | -0.022 | 0.004 | 5.00E-10 | 16 | 84954073 |
| rs2289195 | A | G | 0.43 | 0.038 | 0.003 | 2.00E-37 | 2 | 25240614 |
| rs2284746 | C | G | 0.475 | -0.04 | 0.003 | 1.00E-40 | 1 | 16980180 |
| rs2280470 | A | G | 0.333 | 0.044 | 0.003 | 3.00E-44 | 15 | 88852395 |
| rs2278483 | T | C | 0.235 | 0.041 | 0.003 | 8.00E-33 | 2 | 24817213 |
| rs2240919 | C | G | 0.658 | 0.027 | 0.003 | 5.00E-17 | 3 | 52797685 |
| rs2238300 | A | G | 0.396 | -0.021 | 0.003 | 2.00E-12 | 15 | 89308349 |
| rs2237886 | T | C | 0.107 | 0.043 | 0.005 | 5.00E-18 | 11 | 2789501 |
| rs2224538 | T | C | 0.646 | 0.017 | 0.003 | 2.00E-08 | 20 | 39923436 |
| rs212524 | T | C | 0.404 | -0.021 | 0.003 | 5.00E-12 | 1 | 21256818 |
| rs2120335 | A | G | 0.406 | -0.019 | 0.003 | 8.00E-10 | 2 | 68267870 |
| rs2093210 | T | C | 0.581 | -0.039 | 0.003 | 3.00E-35 | 14 | 60490561 |
| rs2079795 | T | C | 0.328 | 0.045 | 0.003 | 2.00E-46 | 17 | 61419288 |
| rs2074977 | A | C | 0.637 | -0.029 | 0.003 | 2.00E-20 | 19 | 3434030 |
| rs1950500 | T | C | 0.296 | 0.031 | 0.003 | 3.00E-22 | 14 | 24361644 |
| rs1923367 | G | C | 0.48 | -0.03 | 0.003 | 5.00E-24 | 10 | 79373073 |
| rs1884897 | A | G | 0.364 | 0.044 | 0.003 | 1.00E-48 | 20 | 6632185 |
| rs1832871 | A | G | 0.337 | 0.025 | 0.003 | 2.00E-15 | 6 | 158301002 |
| rs1815314 | A | G | 0.422 | -0.022 | 0.003 | 5.00E-14 | 10 | 79169036 |
| rs1812175 | A | G | 0.163 | -0.079 | 0.004 | 2.00E-86 | 4 | 144653692 |
| rs1809889 | T | C | 0.289 | 0.032 | 0.003 | 4.00E-21 | 12 | 124316680 |
| rs17807185 | A | G | 0.618 | -0.022 | 0.003 | 4.00E-13 | 7 | 77678978 |
| rs17556750 | A | C | 0.314 | 0.046 | 0.003 | 8.00E-48 | 4 | 81234414 |
| rs17450430 | A | T | 0.761 | -0.035 | 0.003 | 2.00E-24 | 20 | 49155727 |
| rs17391694 | T | C | 0.123 | 0.043 | 0.005 | 4.00E-16 | 1 | 78157942 |
| rs17181956 | T | C | 0.129 | -0.028 | 0.005 | 4.00E-09 | 2 | 217281357 |
| rs17081935 | T | C | 0.195 | 0.031 | 0.004 | 7.00E-17 | 4 | 56957310 |
| rs16964211 | A | G | 0.051 | -0.057 | 0.007 | 1.00E-15 | 15 | 51238298 |
| rs16859517 | T | C | 0.039 | 0.067 | 0.008 | 5.00E-17 | 2 | 219084462 |
| rs1681630 | T | C | 0.34 | 0.029 | 0.003 | 2.00E-20 | 11 | 47947600 |
| rs1562975 | A | G | 0.301 | 0.025 | 0.003 | 6.00E-15 | 4 | 108487452 |
| rs143384 | A | G | 0.576 | -0.075 | 0.03 | 1.00E-121 | 20 | 35437976 |
| rs1401795 | A | G | 0.508 | 0.03 | 0.003 | 1.00E-23 | 17 | 56762291 |
| rs13393800 | A | G | 0.295 | 0.027 | 0.003 | 1.00E-17 | 2 | 232577381 |
| rs13177718 | T | C | 0.075 | -0.043 | 0.006 | 3.00E-13 | 5 | 108777643 |
| rs13113518 | T | C | 0.636 | -0.018 | 0.003 | 8.00E-09 | 4 | 55533481 |
| rs13088462 | T | C | 0.941 | -0.059 | 0.007 | 8.00E-18 | 3 | 51034282 |
| rs1265097 | A | C | 0.115 | -0.059 | 0.005 | 2.00E-32 | 6 | 31138682 |
| rs12538407 | A | G | 0.602 | 0.032 | 0.003 | 4.00E-26 | 7 | 23481697 |
| rs12411277 | A | G | 0.368 | -0.022 | 0.003 | 2.00E-13 | 1 | 218802133 |
| rs12330322 | T | C | 0.218 | -0.034 | 0.004 | 3.00E-22 | 3 | 72406204 |
| rs12214804 | T | C | 0.92 | -0.084 | 0.006 | 2.00E-49 | 6 | 34221089 |
| rs12209223 | A | C | 0.116 | 0.051 | 0.005 | 5.00E-25 | 6 | 75454873 |
| rs12144094 | C | G | 0.844 | 0.025 | 0.004 | 2.00E-09 | 1 | 119722200 |
| rs12120956 | A | G | 0.229 | -0.025 | 0.004 | 2.00E-12 | 1 | 112659949 |
| rs11950938 | C | G | 0.971 | -0.061 | 0.009 | 5.00E-11 | 5 | 177149292 |
| rs11880992 | A | G | 0.398 | 0.033 | 0.003 | 7.00E-28 | 19 | 2176404 |
| rs11835818 | T | C | 0.512 | -0.022 | 0.003 | 2.00E-13 | 12 | 122056903 |
| rs11783655 | A | T | 0.395 | -0.018 | 0.003 | 1.00E-09 | 8 | 143963405 |
| rs11750568 | A | G | 0.332 | 0.02 | 0.003 | 6.00E-11 | 5 | 179108712 |
| rs11684404 | T | C | 0.661 | -0.032 | 0.003 | 9.00E-25 | 2 | 88625104 |
| rs11677466 | A | T | 0.914 | -0.064 | 0.007 | 3.00E-23 | 2 | 232117547 |
| rs11612228 | T | C | 0.378 | 0.02 | 0.003 | 7.00E-10 | 12 | 467818 |
| rs1155939 | A | C | 0.499 | 0.042 | 0.003 | 1.00E-45 | 6 | 126544987 |
| rs11152213 | A | C | 0.755 | -0.025 | 0.004 | 7.00E-13 | 18 | 60185715 |
| rs11144688 | A | G | 0.108 | -0.063 | 0.006 | 6.00E-24 | 9 | 75927370 |
| rs11049611 | T | C | 0.304 | -0.038 | 0.003 | 3.00E-32 | 12 | 28447311 |
| rs10997979 | G | A | 0.501 | -0.021 | 0.003 | 4.00E-13 | 10 | 68177435 |
| rs10958476 | T | C | 0.786 | -0.051 | 0.004 | 2.00E-40 | 8 | 56183249 |
| rs10948222 | C | T | 0.418 | -0.031 | 0.003 | 1.00E-20 | 6 | 45276678 |
| rs10859567 | T | G | 0.563 | 0.035 | 0.003 | 5.00E-33 | 12 | 93733149 |
| rs10770705 | A | C | 0.338 | 0.03 | 0.003 | 2.00E-21 | 12 | 20704533 |
| rs10748128 | T | G | 0.352 | 0.038 | 0.003 | 4.00E-29 | 12 | 69433878 |
| rs1074683 | C | G | 0.757 | 0.044 | 0.003 | 8.00E-38 | 20 | 33716847 |
| rs10401193 | A | G | 0.819 | 0.028 | 0.004 | 9.00E-14 | 19 | 19480257 |
| rs1036821 | A | G | 0.3 | -0.037 | 0.003 | 1.00E-30 | 8 | 134638240 |
| rs1007358 | A | G | 0.773 | -0.021 | 0.004 | 9.00E-10 | 7 | 46161757 |
| rs9993613 | T | G | 0.473 | 0.03 | 0.003 | 5.00E-24 | 4 | 72610297 |
| rs994533 | C | G | 0.331 | -0.027 | 0.003 | 1.00E-17 | 2 | 217419555 |
| rs991967 | A | C | 0.717 | -0.034 | 0.003 | 2.00E-26 | 1 | 218442109 |
| **Hypertension** | | | | | | | | |
| rs10849937 | G | A | 0.182 | 0.052 | 0.008 | 7.00E-12 | 12 | 111354623 |
| rs10895001 | A | G | 0.273 | 0.066 | 0.007 | 5.00E-22 | 11 | 100662290 |
| rs11191580 | C | T | 0.061 | 0.105 | 0.011 | 3.00E-20 | 10 | 103146454 |
| rs1173727 | C | T | 0.49 | 0.063 | 0.006 | 4.00E-24 | 5 | 32830415 |
| rs117470167 | A | G | 0.056 | 0.092 | 0.015 | 2.00E-09 | 17 | 48589121 |
| rs11754682 | C | T | 0.429 | -0.049 | 0.006 | 3.00E-15 | 6 | 126856574 |
| rs12258967 | G | C | 0.349 | 0.064 | 0.007 | 1.00E-21 | 10 | 18439030 |
| rs13107325 | T | C | 0.096 | 0.08 | 0.012 | 5.00E-12 | 4 | 102267552 |
| rs1327235 | A | G | 0.48 | -0.043 | 0.006 | 3.00E-12 | 20 | 10988382 |
| rs1530440 | T | C | 0.187 | 0.054 | 0.008 | 3.00E-12 | 10 | 61764833 |
| rs167479 | T | G | 0.429 | 0.049 | 0.006 | 8.00E-16 | 19 | 11416089 |
| rs16982520 | G | A | 0.061 | -0.074 | 0.009 | 6.00E-15 | 20 | 59183665 |
| rs16998073 | T | A | 0.232 | -0.077 | 0.007 | 2.00E-30 | 4 | 80263187 |
| rs17367504 | G | A | 0.157 | 0.101 | 0.008 | 4.00E-34 | 1 | 11802721 |
| rs1872167 | T | C | 0.101 | 0.054 | 0.009 | 9.00E-10 | 11 | 47879717 |
| rs1898841 | C | T | 0.253 | 0.057 | 0.007 | 1.00E-16 | 2 | 164213697 |
| rs2274224 | C | G | 0.455 | 0.047 | 0.006 | 5.00E-14 | 10 | 94279840 |
| rs2293579 | A | G | 0.429 | -0.045 | 0.006 | 5.00E-13 | 11 | 47419207 |
| rs2392929 | G | T | 0.222 | -0.065 | 0.008 | 2.00E-17 | 7 | 106773623 |
| rs2493292 | T | C | 0.167 | -0.054 | 0.009 | 2.00E-09 | 1 | 3412095 |
| rs2586886 | C | T | 0.394 | -0.037 | 0.006 | 4.00E-09 | 2 | 26709163 |
| rs2681492 | C | T | 0.162 | 0.065 | 0.008 | 2.00E-15 | 12 | 89619312 |
| rs34328549 | A | G | 0.091 | 0.073 | 0.009 | 5.00E-15 | 19 | 7253173 |
| rs34550591 | C | A | 0.025 | 0.094 | 0.014 | 2.00E-11 | 1 | 11913466 |
| rs34592089 | A | G | 0.051 | 0.077 | 0.013 | 1.00E-09 | 4 | 102005766 |
| rs35085068 | C | A | 0.359 | -0.036 | 0.006 | 4.00E-09 | 14 | 22940700 |
| rs3744760 | C | T | 0.253 | -0.061 | 0.007 | 1.00E-18 | 17 | 45118614 |
| rs4690974 | C | T | 0.48 | -0.037 | 0.006 | 2.00E-09 | 4 | 155472489 |
| rs4888372 | G | A | 0.414 | 0.036 | 0.006 | 5.00E-09 | 16 | 75279587 |
| rs5016282 | G | A | 0.151 | 0.052 | 0.009 | 3.00E-09 | 11 | 89008492 |
| rs56352102 | T | C | 0.177 | -0.055 | 0.008 | 3.00E-12 | 11 | 10247046 |
| rs6461992 | A | G | 0.056 | 0.068 | 0.012 | 7.00E-09 | 7 | 27181212 |
| rs7497304 | T | G | 0.343 | -0.057 | 0.007 | 2.00E-18 | 15 | 90885946 |
| rs805293 | A | T | 0.47 | -0.043 | 0.006 | 3.00E-12 | 6 | 31720741 |
| rs936226 | C | T | 0.253 | -0.054 | 0.007 | 9.00E-15 | 15 | 74776941 |
| rs10849937 | G | A | 0.182 | 0.052 | 0.008 | 7.00E-12 | 12 | 111354623 |
| rs10895001 | A | G | 0.273 | 0.066 | 0.007 | 5.00E-22 | 11 | 100662290 |
| rs11191580 | C | T | 0.061 | 0.105 | 0.011 | 3.00E-20 | 10 | 103146454 |
| rs1173727 | C | T | 0.49 | 0.063 | 0.006 | 4.00E-24 | 5 | 32830415 |
| rs117470167 | A | G | 0.056 | 0.092 | 0.015 | 2.00E-09 | 17 | 48589121 |
| rs11754682 | C | T | 0.429 | -0.049 | 0.006 | 3.00E-15 | 6 | 126856574 |
| rs12258967 | G | C | 0.349 | 0.064 | 0.007 | 1.00E-21 | 10 | 18439030 |
| rs13107325 | T | C | 0.096 | 0.08 | 0.012 | 5.00E-12 | 4 | 102267552 |
| **IGF1** | | | | | | | | |
| rs2153960 | A | G | 0.69 | 6.6 | 1.31309 | 5.00E-07 | 6 | 108666981 |
| rs1245541 | G | A | 0.61 | 2.8 | 0.557068 | 5.00E-07 | 10 | 72089881 |
| rs700752 | G | C | 0.65 | 3.3 | 0.564375 | 5.00E-09 | 7 | 46713955 |
| rs7780564 | C | A | 0.55 | 2.7 | 0.532654 | 4.00E-07 | 7 | 7843692 |
| **IGFBP3** | | | | | | | | |
| rs1065656 | G | C | 0.68 | 61.56 | 9.044292 | 1.00E-11 | 16 | 1788835 |
| rs11977526 | G | A | 0.41 | 177.505 | 8.30928 | 3.00E-101 | 7 | 45968511 |
| rs4234798 | T | G | 0.39 | 35.7172 | 5.743142 | 5.00E-10 | 4 | 7218206 |
| rs700752 | G | C | 0.65 | 50.4 | 5.343164 | 4.00E-21 | 7 | 46713955 |
| **IL6** | | | | | | | | |
| rs10982213 | G | A | 0.773 | 0.08 | 0.018 | 5.00E-06 | 9 | 114426913 |
| rs11110094 | G | A | 0.92 | 0.082 | 0.018 | 6.00E-06 | 12 | 99894329 |
| rs113207090 | C | T | 0.985 | -0.392 | 0.077 | 4.00E-07 | 14 | 91793465 |
| rs114373846 | C | T | 0.975 | -0.422 | 0.092 | 3.00E-06 | 3 | 152848274 |
| rs117146485 | C | T | 0.025 | 0.283 | 0.064 | 6.00E-06 | 9 | 135932411 |
| rs12079357 | G | A | 0.051 | 0.184 | 0.041 | 7.00E-06 | 1 | 214882179 |
| rs1333040 | C | T | 0.46 | -0.074 | 0.016 | 3.00E-06 | 9 | 22083405 |
| rs13412535 | G | A | 0.783 | 0.116 | 0.022 | 7.00E-08 | 2 | 224010157 |
| rs141644173 | ATAAG | A | 0.939 | 0.154 | 0.034 | 4.00E-06 | 2 | 166880727 |
| rs143858123 | T | A | 0.999 | -0.345 | 0.079 | 8.00E-06 | 22 | 47075298 |
| rs148614378 | C | T | 0.507 | -0.079 | 0.017 | 6.00E-06 | 7 | 77079762 |
| rs182261775 | G | A | 0.995 | -0.564 | 0.122 | 5.00E-06 | 14 | 93277130 |
| rs185628618 | G | A | 0.995 | 0.266 | 0.059 | 3.00E-06 | 6 | 21890181 |
| rs1884910 | G | C | 0.333 | -0.079 | 0.017 | 3.00E-06 | 20 | 56451685 |
| rs189539059 | G | A | 0.001 | 0.566 | 0.13 | 9.00E-06 | 8 | 37445086 |
| rs2004627 | C | T | 0.49 | 0.072 | 0.016 | 7.00E-06 | 9 | 21707493 |
| rs2404476 | G | A | 0.5 | -0.07 | 0.016 | 8.00E-06 | 4 | 154690067 |
| rs4684700 | C | T | 0.47 | 0.072 | 0.016 | 9.00E-06 | 3 | 10507266 |
| rs62486616 | G | C | 0.01 | -0.288 | 0.066 | 9.00E-06 | 7 | 104987457 |
| rs72831623 | G | A | 0.944 | -0.197 | 0.038 | 1.00E-07 | 17 | 47644927 |
| rs73273528 | C | T | 0.98 | -0.267 | 0.056 | 1.00E-06 | 20 | 51814574 |
| rs76856708 | C | T | 0.035 | -0.329 | 0.071 | 3.00E-06 | 16 | 80695146 |
| rs8089344 | G | C | 0.232 | 0.117 | 0.026 | 8.00E-06 | 18 | 6615784 |
| rs10982213 | G | A | 0.773 | 0.08 | 0.018 | 5.00E-06 | 9 | 114426913 |
| rs11110094 | G | A | 0.92 | 0.082 | 0.018 | 6.00E-06 | 12 | 99894329 |
| **LDL** | | | | | | | | |
| rs995000 | T | C | 0.345 | -0.027 | 0.003 | 6.00E-22 | 1 | 62641855 |
| rs976058 | T | C | 0.586 | 0.022 | 0.003 | 2.00E-16 | 4 | 68475769 |
| rs941408 | T | C | 0.279 | 0.015 | 0.002 | 5.00E-10 | 19 | 2814183 |
| rs9297994 | A | G | 0.69 | -0.028 | 0.003 | 8.00E-21 | 8 | 58479765 |
| rs892161 | A | G | 0.378 | 0.012 | 0.002 | 5.00E-08 | 19 | 4493711 |
| rs871841 | T | C | 0.462 | -0.015 | 0.003 | 2.00E-08 | 17 | 8313150 |
| rs870992 | A | G | 0.915 | -0.027 | 0.004 | 5.00E-11 | 5 | 52897406 |
| rs855791 | A | G | 0.407 | 0.012 | 0.002 | 1.00E-10 | 22 | 37066896 |
| rs826682 | A | C | 0.822 | -0.018 | 0.003 | 1.00E-09 | 2 | 108615932 |
| rs8022288 | T | C | 0.864 | 0.026 | 0.004 | 3.00E-11 | 14 | 70380407 |
| rs80215559 | T | C | 0.94 | 0.057 | 0.006 | 3.00E-21 | 6 | 25917997 |
| rs7904973 | T | G | 0.557 | 0.02 | 0.003 | 1.00E-14 | 10 | 122934071 |
| rs78946096 | A | G | 0.95 | 0.041 | 0.007 | 2.00E-10 | 3 | 132469319 |
| rs7873387 | A | C | 0.814 | -0.021 | 0.004 | 4.00E-08 | 9 | 104833321 |
| rs7821092 | C | G | 0.571 | -0.021 | 0.003 | 2.00E-11 | 8 | 10651291 |
| rs780094 | T | C | 0.371 | 0.026 | 0.003 | 3.00E-21 | 2 | 27518370 |
| rs77987196 | D | I | 0.71 | -0.025 | 0.003 | 2.00E-18 | 10 | 112173249 |
| rs77960347 | A | G | 0.988 | -0.075 | 0.013 | 4.00E-09 | 18 | 49583585 |
| rs77542162 | A | G | 0.982 | -0.162 | 0.011 | 4.00E-52 | 17 | 69085137 |
| rs7746081 | A | G | 0.406 | -0.02 | 0.003 | 3.00E-12 | 6 | 16126703 |
| rs77375493 | T | G | 1.00E-03 | -0.451 | 0.061 | 9.00E-14 | 9 | 5073770 |
| rs7567229 | A | C | 0.367 | -0.019 | 0.003 | 4.00E-11 | 2 | 233703893 |
| rs7538216 | T | C | 0.422 | -0.013 | 0.002 | 5.00E-08 | 1 | 16183176 |
| rs73461870 | A | G | 0.891 | -0.025 | 0.005 | 3.00E-08 | 11 | 46279704 |
| rs73339979 | C | G | 0.175 | -0.049 | 0.008 | 4.00E-11 | 17 | 4790607 |
| rs73219351 | T | G | 0.647 | 0.032 | 0.005 | 4.00E-09 | 2 | 17295273 |
| rs73066485 | T | G | 0.813 | -0.03 | 0.004 | 2.00E-16 | 7 | 21572352 |
| rs72951954 | A | C | 0.392 | -0.017 | 0.003 | 3.00E-10 | 6 | 116072564 |
| rs704 | A | G | 0.501 | 0.019 | 0.003 | 1.00E-13 | 17 | 28367840 |
| rs703212 | A | G | 0.18 | -0.028 | 0.004 | 2.00E-14 | 13 | 32466293 |
| rs6907508 | A | G | 0.888 | 0.024 | 0.004 | 3.00E-08 | 6 | 34624313 |
| rs6894249 | A | G | 0.578 | -0.015 | 0.003 | 6.00E-09 | 5 | 132461855 |
| rs68055275 | D | I | 0.865 | -0.037 | 0.004 | 1.00E-20 | 11 | 126369249 |
| rs679574 | C | G | 0.52 | -0.025 | 0.003 | 2.00E-21 | 19 | 48702851 |
| rs66883945 | D | I | 0.819 | -0.021 | 0.004 | 4.00E-09 | 6 | 138996691 |
| rs66650371 | D | I | 0.228 | -0.031 | 0.003 | 1.00E-19 | 6 | 135097495 |
| rs6602911 | T | C | 0.429 | 0.015 | 0.003 | 3.00E-08 | 13 | 113844399 |
| rs6557781 | T | C | 0.184 | -0.019 | 0.003 | 2.00E-10 | 8 | 22080156 |
| rs653178 | T | C | 0.551 | 0.026 | 0.003 | 4.00E-20 | 12 | 111569952 |
| rs648324 | T | G | 0.698 | 0.015 | 0.003 | 3.00E-09 | 1 | 10496390 |
| rs62259757 | A | G | 0.068 | 0.039 | 0.007 | 4.00E-09 | 3 | 51995975 |
| rs62072497 | A | G | 0.188 | 0.02 | 0.003 | 3.00E-09 | 17 | 18222563 |
| rs611917 | A | G | 0.682 | 0.112 | 0.003 | 1.00E-300 | 1 | 109272630 |
| rs6088882 | C | G | 0.201 | -0.025 | 0.003 | 8.00E-14 | 20 | 35578178 |
| rs58466006 | A | G | 0.432 | -0.015 | 0.003 | 2.00E-08 | 5 | 123593271 |
| rs58198139 | T | C | 0.582 | 0.026 | 0.003 | 3.00E-22 | 5 | 156972028 |
| rs577584 | A | T | 0.69 | 0.101 | 0.003 | 1.00E-229 | 2 | 21076995 |
| rs55831924 | T | C | 0.334 | 0.018 | 0.003 | 6.00E-10 | 8 | 143957800 |
| rs5471 | A | C | 0.904 | -0.176 | 0.011 | 5.00E-57 | 16 | 72054562 |
| rs535064984 | T | C | 0.996 | 0.381 | 0.033 | 7.00E-32 | 17 | 7116978 |
| rs506234 | C | G | 0.564 | -0.017 | 0.003 | 8.00E-11 | 11 | 118669395 |
| rs484084 | T | C | 0.418 | -0.033 | 0.003 | 2.00E-31 | 1 | 234721929 |
| rs4773173 | A | G | 0.657 | 0.016 | 0.002 | 5.00E-12 | 13 | 110372771 |
| rs4722551 | T | C | 0.846 | -0.032 | 0.004 | 9.00E-18 | 7 | 25952206 |
| rs4560319 | T | C | 0.082 | -0.027 | 0.005 | 4.00E-08 | 3 | 58400547 |
| rs4418728 | T | G | 0.427 | -0.016 | 0.003 | 2.00E-09 | 10 | 93079967 |
| rs4245791 | T | C | 0.707 | -0.06 | 0.003 | 6.00E-94 | 2 | 43847292 |
| rs41274050 | T | C | 0.009 | 0.092 | 0.015 | 6.00E-10 | 10 | 50814012 |
| rs3812945 | T | C | 0.573 | -0.014 | 0.002 | 8.00E-10 | 15 | 74997381 |
| rs3780181 | A | G | 0.878 | 0.032 | 0.005 | 1.00E-11 | 9 | 2640759 |
| rs3758413 | T | C | 0.61 | -0.018 | 0.003 | 8.00E-11 | 10 | 17226840 |
| rs360801 | A | G | 0.664 | 0.019 | 0.003 | 7.00E-10 | 2 | 62728252 |
| rs34318965 | T | C | 0.713 | 0.017 | 0.003 | 8.00E-09 | 16 | 11537712 |
| rs329007 | A | G | 0.774 | 0.016 | 0.003 | 3.00E-09 | 18 | 9522608 |
| rs3010276 | A | G | 0.197 | -0.015 | 0.003 | 4.00E-08 | 5 | 72718742 |
| rs2992753 | A | C | 0.646 | -0.011 | 0.002 | 5.00E-09 | 1 | 18481798 |
| rs28555129 | A | C | 0.315 | 0.013 | 0.002 | 6.00E-10 | 16 | 83951171 |
| rs2712199 | A | G | 0.701 | -0.015 | 0.003 | 8.00E-09 | 7 | 107499794 |
| rs2642438 | A | G | 0.271 | -0.025 | 0.003 | 7.00E-16 | 1 | 220796686 |
| rs2618566 | T | G | 0.649 | -0.03 | 0.003 | 9.00E-22 | 20 | 17864040 |
| rs2519093 | T | C | 0.181 | 0.074 | 0.003 | 8.00E-102 | 9 | 133266456 |
| rs249756 | A | G | 0.333 | 0.016 | 0.003 | 4.00E-08 | 5 | 142533938 |
| rs2389606 | T | C | 0.579 | -0.015 | 0.003 | 2.00E-08 | 2 | 168973262 |
| rs2218260 | T | C | 0.59 | -0.019 | 0.003 | 4.00E-09 | 15 | 57922012 |
| rs221797 | A | C | 0.099 | 0.02 | 0.004 | 4.00E-08 | 7 | 100688351 |
| rs2183573 | A | G | 0.41 | -0.016 | 0.002 | 2.00E-14 | 21 | 39202379 |
| rs2169387 | A | G | 0.131 | -0.042 | 0.004 | 3.00E-25 | 8 | 9323885 |
| rs2001846 | T | C | 0.448 | 0.054 | 0.003 | 3.00E-82 | 8 | 125466208 |
| rs199987224 | D | I | 0.393 | 0.065 | 0.003 | 2.00E-79 | 5 | 75269375 |
| rs1981405 | T | C | 0.134 | -0.022 | 0.004 | 6.00E-09 | 11 | 78265162 |
| rs1883711 | C | G | 0.027 | 0.109 | 0.012 | 9.00E-21 | 20 | 40551182 |
| rs182472492 | A | G | 0.725 | 0.028 | 0.003 | 6.00E-19 | 2 | 202624734 |
| rs1801689 | A | C | 0.969 | -0.092 | 0.008 | 2.00E-30 | 17 | 66214462 |
| rs17725246 | T | C | 0.793 | -0.033 | 0.003 | 2.00E-22 | 7 | 44542387 |
| rs17660708 | T | C | 0.961 | -0.036 | 0.006 | 1.00E-10 | 21 | 31687518 |
| rs17580 | A | T | 0.041 | 0.062 | 0.007 | 1.00E-18 | 14 | 94380925 |
| rs17561950 | A | G | 0.459 | 0.018 | 0.003 | 2.00E-11 | 17 | 78396063 |
| rs174549 | A | G | 0.277 | -0.03 | 0.003 | 4.00E-20 | 11 | 61803910 |
| rs1730859 | A | G | 0.69 | -0.019 | 0.003 | 4.00E-14 | 1 | 107075085 |
| rs17050272 | A | G | 0.389 | -0.022 | 0.003 | 2.00E-14 | 2 | 120548864 |
| rs17029617 | A | G | 0.161 | -0.033 | 0.004 | 4.00E-16 | 3 | 32500061 |
| rs1521516 | T | C | 0.308 | -0.016 | 0.002 | 3.00E-12 | 12 | 50661925 |
| rs145955280 | D | I | 0.94 | -0.101 | 0.012 | 5.00E-17 | 1 | 92671973 |
| rs145859971 | A | G | 0.061 | -0.074 | 0.012 | 8.00E-10 | 11 | 116817950 |
| rs144787122 | A | G | 0.004 | 0.1 | 0.017 | 1.00E-08 | 7 | 2256917 |
| rs143020224 | C | G | 0.869 | 0.162 | 0.004 | 1.00E-300 | 19 | 11076648 |
| rs14234 | A | G | 0.547 | -0.011 | 0.002 | 2.00E-08 | 2 | 70297010 |
| rs139604259 | D | I | 0.428 | -0.018 | 0.003 | 6.00E-12 | 1 | 25442719 |
| rs138295924 | A | G | 0.955 | 0.098 | 0.007 | 8.00E-42 | 19 | 19283559 |
| rs13288021 | T | C | 0.082 | 0.041 | 0.006 | 2.00E-10 | 9 | 19217423 |
| rs12712955 | A | G | 0.532 | 0.014 | 0.003 | 2.00E-08 | 2 | 45939182 |
| rs12597024 | T | C | 0.638 | 0.016 | 0.003 | 2.00E-08 | 16 | 88484745 |
| rs12464355 | A | G | 0.909 | 0.038 | 0.005 | 2.00E-14 | 2 | 118092274 |
| rs12171249 | A | G | 0.264 | -0.014 | 0.003 | 5.00E-08 | 22 | 50440498 |
| rs118147862 | A | G | 0.037 | -0.398 | 0.008 | 1.00E-300 | 19 | 44816374 |
| rs117492019 | T | G | 0.176 | -0.022 | 0.004 | 1.00E-08 | 19 | 58170494 |
| rs1169288 | A | C | 0.694 | -0.033 | 0.003 | 1.00E-27 | 12 | 120978847 |
| rs11591147 | T | G | 0.015 | -0.395 | 0.012 | 3.00E-257 | 1 | 55039974 |
| rs11456863 | D | I | 0.61 | 0.021 | 0.003 | 1.00E-14 | 3 | 12230419 |
| rs11406672 | D | I | 0.679 | -0.017 | 0.003 | 1.00E-08 | 3 | 122507441 |
| rs112595563 | A | G | 0.735 | 0.02 | 0.003 | 4.00E-09 | 16 | 68020885 |
| rs112259268 | A | C | 0.028 | -0.063 | 0.009 | 1.00E-11 | 17 | 43797377 |
| rs11218721 | A | C | 0.381 | 0.018 | 0.003 | 6.00E-10 | 11 | 122634009 |
| rs10513551 | T | G | 0.53 | -0.014 | 0.002 | 1.00E-10 | 3 | 160368267 |
| rs1047743 | C | G | 0.603 | 0.016 | 0.003 | 2.00E-08 | 17 | 75794012 |
| rs10455872 | A | G | 0.935 | -0.088 | 0.006 | 3.00E-54 | 6 | 160589086 |
| rs10275712 | A | G | 0.198 | -0.028 | 0.003 | 2.00E-16 | 7 | 1043282 |
| rs1014283 | A | C | 0.275 | -0.016 | 0.003 | 7.00E-10 | 7 | 87447271 |
| chr17:45734210 | D | I | 0.502 | -0.022 | 0.003 | 2.00E-16 | 17 | 45734210 |
| rs995000 | T | C | 0.345 | -0.027 | 0.003 | 6.00E-22 | 1 | 62641855 |
| rs976058 | T | C | 0.586 | 0.022 | 0.003 | 2.00E-16 | 4 | 68475769 |
| **Metabolic syndrome** | | | | | | | | |
| rs964184 | G | C | 0.132408 | 0.252628 | 0.009175 | 7.00E-167 | 11 | 116778201 |
| rs247617 | G | C | 0.324218 | -0.19643 | 0.007146 | 2.00E-166 | 16 | 56956804 |
| rs3844510 | C | A | 0.26399 | -0.19113 | 0.007627 | 1.00E-138 | 8 | 20003850 |
| rs72836561 | T | C | 0.032265 | 0.23953 | 0.017414 | 5.00E-43 | 17 | 43848758 |
| rs2980888 | T | C | 0.299847 | 0.095952 | 0.007057 | 4.00E-42 | 8 | 125495066 |
| rs116843064 | A | G | 0.019184 | -0.33748 | 0.026334 | 1.00E-37 | 19 | 8364439 |
| rs2281721 | C | T | 0.38621 | 0.082075 | 0.006679 | 1.00E-34 | 1 | 230161390 |
| rs483082 | T | G | 0.238624 | 0.08993 | 0.007578 | 2.00E-32 | 19 | 44912921 |
| rs673548 | A | G | 0.204049 | -0.09779 | 0.008242 | 2.00E-32 | 2 | 21014672 |
| rs1535 | G | A | 0.347774 | 0.079213 | 0.006808 | 3.00E-31 | 11 | 61830500 |
| rs261290 | T | C | 0.347343 | -0.07796 | 0.006917 | 2.00E-29 | 15 | 58386521 |
| rs56094641 | G | A | 0.402905 | 0.073677 | 0.006639 | 1.00E-28 | 16 | 53772541 |
| rs2138161 | T | C | 0.351852 | -0.07524 | 0.006882 | 8.00E-28 | 2 | 226230443 |
| rs998584 | A | C | 0.481524 | 0.070685 | 0.006551 | 4.00E-27 | 6 | 43790159 |
| rs28597716 | G | A | 0.189687 | -0.15218 | 0.008672 | 1.00E-24 | 8 | 20079176 |
| rs12056034 | G | A | 0.124753 | -0.09684 | 0.010092 | 8.00E-22 | 7 | 73464315 |
| rs66922415 | G | A | 0.234519 | 0.067846 | 0.007625 | 6.00E-19 | 18 | 60181418 |
| rs2925979 | T | C | 0.301767 | 0.06161 | 0.007063 | 3.00E-18 | 16 | 81501185 |
| rs7124681 | A | C | 0.408583 | 0.055786 | 0.006615 | 3.00E-17 | 11 | 47508395 |
| rs11206374 | A | G | 0.225766 | 0.065077 | 0.007736 | 4.00E-17 | 1 | 39582337 |
| rs61057119 | T | TA | 0.187918 | 0.069464 | 0.008297 | 6.00E-17 | 20 | 44553722 |
| rs61789601 | T | C | 0.202909 | -0.06837 | 0.008209 | 8.00E-17 | 3 | 136236137 |
| rs11429307 | GT | G | 0.190881 | 0.066966 | 0.008228 | 4.00E-16 | 5 | 56561199 |
| rs11754773 | G | A | 0.094228 | 0.089294 | 0.011005 | 5.00E-16 | 6 | 34609480 |
| rs1260326 | T | C | 0.392938 | 0.053697 | 0.006656 | 7.00E-16 | 2 | 27508073 |
| rs139974673 | C | T | 0.025125 | 0.160596 | 0.020047 | 1.00E-15 | 15 | 43735687 |
| rs9987289 | A | G | 0.091975 | 0.087388 | 0.011076 | 3.00E-15 | 8 | 9325848 |
| rs1800961 | T | C | 0.031482 | 0.138848 | 0.018036 | 1.00E-14 | 20 | 44413724 |
| rs10822155 | A | C | 0.41581 | -0.05112 | 0.006651 | 2.00E-14 | 10 | 63311455 |
| rs12599637 | C | T | 0.403682 | -0.05097 | 0.006773 | 5.00E-14 | 16 | 69559452 |
| rs638714 | T | G | 0.347785 | -0.0515 | 0.006916 | 1.00E-13 | 1 | 62440818 |
| rs632057 | T | G | 0.372799 | 0.048679 | 0.00673 | 5.00E-13 | 6 | 139512875 |
| rs10260148 | T | C | 0.27901 | 0.052129 | 0.007255 | 7.00E-13 | 7 | 130746210 |
| rs114165349 | C | G | 0.023517 | 0.147253 | 0.020874 | 2.00E-12 | 1 | 26695422 |
| rs10187501 | G | A | 0.346097 | -0.04656 | 0.006907 | 2.00E-11 | 2 | 164675944 |
| rs1532127 | G | A | 0.314222 | -0.04721 | 0.007068 | 2.00E-11 | 19 | 47068681 |
| rs7239575 | C | T | 0.49327 | -0.04323 | 0.006533 | 4.00E-11 | 18 | 23540071 |
| rs10049088 | T | C | 0.386891 | -0.04432 | 0.006743 | 5.00E-11 | 3 | 157079859 |
| rs1009360 | C | T | 0.415241 | -0.04359 | 0.006637 | 5.00E-11 | 2 | 65048915 |
| rs7660883 | G | C | 0.377013 | -0.04439 | 0.006793 | 6.00E-11 | 4 | 87061724 |
| rs11075253 | A | C | 0.297121 | -0.04688 | 0.007193 | 7.00E-11 | 16 | 15054789 |
| rs1534696 | C | A | 0.460436 | 0.042436 | 0.006543 | 9.00E-11 | 7 | 26357619 |
| rs7563362 | A | G | 0.142333 | -0.06136 | 0.009505 | 1.00E-10 | 2 | 620297 |
| rs9378248 | A | G | 0.339668 | 0.044068 | 0.006885 | 2.00E-10 | 6 | 31358512 |
| rs563296 | G | A | 0.44029 | -0.04194 | 0.006593 | 2.00E-10 | 10 | 98012647 |
| rs1023193 | T | G | 0.305574 | -0.0454 | 0.00716 | 2.00E-10 | 15 | 41563538 |
| rs13107325 | T | C | 0.074856 | 0.07609 | 0.012188 | 4.00E-10 | 4 | 102267552 |
| chr12:124503803 | CAA | C | 0.436617 | -0.04149 | 0.006648 | 4.00E-10 | 12 | 124503803 |
| rs779518917 | GTGT | G | 0.403203 | 0.041535 | 0.006683 | 5.00E-10 | 17 | 78402044 |
| rs35661464 | T | C | 0.252033 | 0.04638 | 0.00747 | 5.00E-10 | 11 | 65061370 |
| rs764311894 | CTTT | C | 0.169241 | -0.05698 | 0.00918 | 5.00E-10 | 20 | 52588195 |
| rs748326686 | CT | C | 0.37669 | 0.042439 | 0.006863 | 6.00E-10 | 4 | 66985805 |
| rs577721086 | C | T | 0.05096 | 0.090094 | 0.014708 | 9.00E-10 | 6 | 127118902 |
| rs537069642 | CTT | C | 0.423828 | -0.04185 | 0.006896 | 1.00E-09 | 3 | 50081955 |
| rs76376137 | G | T | 0.050549 | 0.082033 | 0.01461 | 1.00E-09 | 6 | 34205553 |
| rs10913469 | C | T | 0.207314 | 0.048264 | 0.007996 | 2.00E-09 | 1 | 177944384 |
| rs56133711 | A | G | 0.262839 | 0.04433 | 0.007379 | 2.00E-09 | 11 | 27701787 |
| rs3814883 | T | C | 0.48348 | 0.039292 | 0.006551 | 2.00E-09 | 16 | 29983601 |
| rs56282717 | A | G | 0.244425 | -0.0461 | 0.007694 | 2.00E-09 | 7 | 150960007 |
| rs12945575 | T | C | 0.25004 | 0.044779 | 0.007511 | 3.00E-09 | 17 | 42561053 |
| rs2306363 | T | G | 0.206589 | -0.04828 | 0.008137 | 3.00E-09 | 11 | 65638129 |
| rs781006834 | G | GT | 0.344659 | 0.041106 | 0.006936 | 3.00E-09 | 7 | 17902243 |
| rs11751347 | T | C | 0.102714 | 0.062488 | 0.010657 | 5.00E-09 | 6 | 160671406 |
| rs73123462 | T | C | 0.015597 | 0.149549 | 0.025521 | 5.00E-09 | 4 | 36075982 |
| rs62107261 | C | T | 0.048257 | -0.08978 | 0.015663 | 5.00E-09 | 2 | 422144 |
| rs3808439 | A | G | 0.447923 | 0.038346 | 0.006573 | 5.00E-09 | 8 | 115551448 |
| rs56959712 | T | G | 0.210819 | -0.04705 | 0.008075 | 6.00E-09 | 12 | 122703928 |
| rs56001710 | A | T | 0.417154 | 0.038814 | 0.006819 | 7.00E-09 | 7 | 25943780? |
| rs779470261 | C | CTCTAA | 0.26329 | 0.04311 | 0.007444 | 7.00E-09 | 3 | 131927320 |
| rs5789783 | T | TA | 0.402641 | -0.03995 | 0.006915 | 8.00E-09 | 11 | 13326202 |
| rs3949781 | T | A | 0.461633 | -0.03802 | 0.006597 | 8.00E-09 | 9 | 89563557 |
| rs11871285 | T | G | 0.193205 | 0.047341 | 0.008221 | 8.00E-09 | 17 | 67844693 |
| rs6545703 | G | T | 0.415927 | -0.03824 | 0.006648 | 9.00E-09 | 2 | 58805500 |
| rs58175144 | CA | C | 0.301172 | 0.041375 | 0.00722 | 1.00E-08 | 1 | 150867223 |
| rs7188873 | A | G | 0.376011 | -0.03863 | 0.006773 | 1.00E-08 | 16 | 24715743 |
| rs5021727 | G | A | 0.461566 | 0.037134 | 0.00656 | 2.00E-08 | 6 | 32610856 |
| rs1105654 | G | A | 0.345091 | 0.038799 | 0.006893 | 2.00E-08 | 18 | 49621376 |
| rs12752223 | T | C | 0.396475 | -0.03748 | 0.006676 | 2.00E-08 | 1 | 93371576 |
| rs140628616 | A | AAT | 0.16585 | -0.05287 | 0.009455 | 2.00E-08 | 11 | 56434234 |
| rs9971210 | G | C | 0.489597 | 0.03658 | 0.006546 | 2.00E-08 | 10 | 21590155 |
| rs10954772 | T | C | 0.313991 | 0.039198 | 0.007049 | 3.00E-08 | 8 | 31006422 |
| rs12472667 | G | C | 0.371305 | 0.03749 | 0.006763 | 3.00E-08 | 2 | 170772553 |
| rs1143015 | A | G | 0.157971 | 0.04914 | 0.008881 | 3.00E-08 | 17 | 7581888 |
| rs11789603 | T | C | 0.107873 | -0.05889 | 0.010691 | 4.00E-08 | 9 | 104884738 |
| rs9332817 | C | G | 0.026501 | -0.1158 | 0.021029 | 4.00E-08 | 11 | 118494495 |
| rs535286942 | AT | A | 0.374794 | -0.03768 | 0.006847 | 4.00E-08 | 10 | 93037445 |
| rs8121509 | C | T | 0.451827 | -0.03607 | 0.006565 | 4.00E-08 | 20 | 64080700 |
| rs143321598 | CT | C | 0.488259 | -0.03829 | 0.006983 | 4.00E-08 | 3 | 39075189 |
| rs10945840 | C | G | 0.332614 | -0.03829 | 0.006991 | 4.00E-08 | 6 | 162553872 |
| rs7138803 | A | G | 0.368675 | 0.036957 | 0.006747 | 4.00E-08 | 12 | 49853685 |
| rs11655056 | C | T | 0.46087 | 0.035993 | 0.006573 | 4.00E-08 | 17 | 49286745 |
| rs4921913 | C | T | 0.221605 | 0.042644 | 0.007797 | 5.00E-08 | 8 | 18414867 |
| rs2307111 | C | T | 0.393101 | -0.03667 | 0.006708 | 5.00E-08 | 5 | 75003678 |
| **Multiple sclerosis** | | | | | | | | |
| rs10063294 | G | A | 0.424 | 0.052 | 0.005 | 2.00E-28 | 5 | 35877403 |
| rs1014486 | C | T | 0.434 | 0.045 | 0.004 | 3.00E-28 | 3 | 159973324 |
| rs10191360 | T | C | 0.394 | 0.035 | 0.006 | 4.00E-10 | 2 | 136127109 |
| rs10230723 | A | T | 0.818 | 0.036 | 0.006 | 8.00E-11 | 7 | 50200284 |
| rs10245867 | T | G | 0.328 | 0.029 | 0.004 | 1.00E-11 | 7 | 28102567 |
| rs1026916 | A | G | 0.374 | 0.047 | 0.004 | 2.00E-28 | 17 | 42377817 |
| rs10271373 | A | C | 0.54 | 0.024 | 0.004 | 3.00E-09 | 7 | 139045049 |
| rs1076928 | T | C | 0.414 | 0.053 | 0.006 | 3.00E-19 | 6 | 36380912 |
| rs1077667 | C | T | 0.783 | 0.062 | 0.005 | 8.00E-33 | 19 | 6668961 |
| rs10801908 | C | T | 0.859 | 0.114 | 0.006 | 5.00E-70 | 1 | 116547871 |
| rs1087056 | A | G | 0.424 | 0.037 | 0.004 | 3.00E-19 | 10 | 31106832 |
| rs10936602 | T | C | 0.732 | 0.032 | 0.005 | 2.00E-11 | 3 | 169818849 |
| rs10951042 | C | T | 0.399 | 0.042 | 0.005 | 1.00E-18 | 7 | 3099783 |
| rs10951154 | C | T | 0.157 | 0.033 | 0.005 | 3.00E-10 | 7 | 27095695 |
| rs11079784 | C | T | 0.515 | 0.044 | 0.004 | 2.00E-27 | 17 | 47624914 |
| rs11083862 | A | T | 0.455 | 0.032 | 0.005 | 2.00E-09 | 19 | 47135282 |
| rs11125803 | C | T | 0.293 | 0.030 | 0.005 | 5.00E-10 | 2 | 24829308 |
| rs1112718 | A | G | 0.606 | 0.036 | 0.004 | 2.00E-17 | 10 | 92719350 |
| rs111430408 | C | T | 0.934 | 0.063 | 0.010 | 6.00E-11 | 3 | 100848597 |
| rs111635774 | C | T | 0.914 | 0.046 | 0.005 | 1.00E-17 | 6 | 14691215 |
| rs11231749 | C | T | 0.318 | 0.029 | 0.004 | 1.00E-11 | 11 | 64327706 |
| rs112344141 | G | T | 0.025 | 0.073 | 0.013 | 2.00E-08 | 1 | 154983036 |
| rs11256593 | T | C | 0.561 | 0.081 | 0.005 | 3.00E-65 | 10 | 6075359 |
| rs112741635 | G | A | 0.581 | 0.029 | 0.005 | 4.00E-08 | 1 | 24207504 |
| rs11542663 | A | C | 0.647 | 0.032 | 0.005 | 3.00E-10 | 6 | 119215402 |
| rs11578655 | G | T | 0.131 | 0.036 | 0.007 | 3.00E-08 | 1 | 100947346 |
| rs116877451 | A | G | 0.96 | 0.038 | 0.005 | 3.00E-16 | 7 | 50328339 |
| rs116899835 | C | T | 0.955 | 0.124 | 0.011 | 2.00E-30 | 14 | 88523488 |
| rs11749040 | A | G | 0.157 | 0.061 | 0.006 | 5.00E-25 | 5 | 40396323 |
| rs1177228 | G | A | 0.758 | 0.040 | 0.004 | 2.00E-19 | 2 | 61015275 |
| rs11852059 | C | A | 0.157 | 0.039 | 0.006 | 1.00E-09 | 14 | 51839373 |
| rs11899404 | C | T | 0.51 | 0.023 | 0.004 | 2.00E-08 | 2 | 12467767 |
| rs11919880 | A | G | 0.525 | 0.025 | 0.004 | 8.00E-10 | 3 | 32920559 |
| rs12133753 | C | T | 0.828 | 0.053 | 0.007 | 2.00E-16 | 1 | 91756532 |
| rs12147246 | A | G | 0.379 | 0.039 | 0.005 | 2.00E-15 | 14 | 102799507 |
| rs12211604 | G | A | 0.359 | 0.028 | 0.005 | 2.00E-08 | 6 | 7099796 |
| rs12365699 | G | A | 0.823 | 0.051 | 0.006 | 2.00E-19 | 11 | 118872577 |
| rs12434551 | A | T | 0.535 | 0.034 | 0.004 | 4.00E-17 | 14 | 68786647 |
| rs12478539 | G | C | 0.722 | 0.048 | 0.005 | 2.00E-24 | 2 | 43128185 |
| rs1250551 | T | G | 0.384 | 0.042 | 0.004 | 2.00E-23 | 10 | 79299578 |
| rs12588969 | G | C | 0.394 | 0.032 | 0.005 | 2.00E-09 | 14 | 102764421 |
| rs12609500 | C | T | 0.753 | 0.025 | 0.004 | 5.00E-09 | 19 | 11063252 |
| rs12614091 | A | T | 0.788 | 0.032 | 0.005 | 6.00E-12 | 2 | 203768138 |
| rs12622670 | T | C | 0.515 | 0.037 | 0.005 | 5.00E-16 | 2 | 68419404 |
| rs12832171 | C | G | 0.992 | 0.067 | 0.011 | 4.00E-10 | 12 | 6332456 |
| rs12925972 | C | T | 0.53 | 0.044 | 0.005 | 1.00E-19 | 16 | 79077400 |
| rs12971909 | A | G | 0.384 | 0.030 | 0.005 | 6.00E-09 | 19 | 4466469 |
| rs13066789 | C | T | 0.444 | 0.028 | 0.005 | 1.00E-08 | 3 | 188269836 |
| rs13136820 | C | T | 0.308 | 0.032 | 0.005 | 8.00E-10 | 4 | 40305547 |
| rs1323292 | A | G | 0.803 | 0.065 | 0.005 | 4.00E-33 | 1 | 192571891 |
| rs13327021 | C | T | 0.611 | 0.035 | 0.004 | 2.00E-16 | 3 | 27741524 |
| rs13385171 | C | T | 0.546 | 0.029 | 0.004 | 3.00E-11 | 2 | 65434709 |
| rs13414105 | C | A | 0.495 | 0.038 | 0.006 | 7.00E-10 | 2 | 30249576 |
| rs1365120 | C | T | 0.121 | 0.040 | 0.007 | 4.00E-08 | 11 | 36416525 |
| rs137955 | T | C | 0.374 | 0.024 | 0.004 | 1.00E-08 | 22 | 39895803 |
| rs138433213 | T | G | 0.99 | 0.134 | 0.023 | 3.00E-09 | 3 | 112693983 |
| rs1399180 | C | T | 0.818 | 0.042 | 0.006 | 7.00E-11 | 10 | 8056756 |
| rs140522 | T | C | 0.318 | 0.048 | 0.005 | 1.00E-21 | 22 | 50532837 |
| rs1415069 | G | C | 0.232 | 0.031 | 0.006 | 3.00E-08 | 1 | 92961312 |
| rs146566517 | T | C | 0.025 | 0.038 | 0.005 | 5.00E-16 | 16 | 11353879 |
| rs1465697 | T | C | 0.263 | 0.040 | 0.005 | 3.00E-18 | 19 | 49333989 |
| rs149114341 | G | A | 0.97 | 0.102 | 0.016 | 9.00E-11 | 11 | 118783424 |
| rs17051321 | T | C | 0.227 | 0.035 | 0.005 | 1.00E-10 | 4 | 121198294 |
| rs1738074 | C | T | 0.525 | 0.054 | 0.004 | 3.00E-35 | 6 | 159044945 |
| rs17724508 | T | C | 0.96 | 0.058 | 0.008 | 6.00E-14 | 16 | 79316307 |
| rs17741873 | G | T | 0.818 | 0.028 | 0.005 | 5.00E-08 | 10 | 73894042 |
| rs17780048 | C | T | 0.944 | 0.042 | 0.006 | 5.00E-12 | 6 | 137858009 |
| rs1800693 | C | T | 0.49 | 0.060 | 0.004 | 2.00E-47 | 12 | 6330843 |
| rs1801133 | G | A | 0.702 | 0.044 | 0.006 | 2.00E-15 | 1 | 11796321 |
| rs200866143 | C | G | 0.522 | 0.056 | 0.004 | 7.00E-43 | 3 | 121765368 |
| rs2084007 | C | T | 0.53 | 0.035 | 0.005 | 2.00E-13 | 5 | 134555592 |
| rs2150879 | G | A | 0.439 | 0.048 | 0.004 | 4.00E-31 | 17 | 59781849 |
| rs2248137 | C | G | 0.601 | 0.038 | 0.004 | 2.00E-19 | 20 | 54173204 |
| rs2269434 | C | T | 0.333 | 0.038 | 0.005 | 1.00E-13 | 11 | 47338861 |
| rs2289746 | C | T | 0.606 | 0.035 | 0.005 | 5.00E-12 | 3 | 105737111 |
| rs2317231 | G | T | 0.525 | 0.034 | 0.004 | 3.00E-16 | 1 | 157716547 |
| rs2327586 | T | C | 0.737 | 0.043 | 0.005 | 9.00E-20 | 6 | 135174088 |
| rs2331964 | C | T | 0.657 | 0.039 | 0.004 | 5.00E-20 | 3 | 121824051 |
| rs2364485 | A | C | 0.172 | 0.048 | 0.005 | 2.00E-20 | 12 | 6405797 |
| rs244656 | A | T | 0.899 | 0.046 | 0.006 | 3.00E-14 | 5 | 134114136 |
| rs2469434 | C | T | 0.364 | 0.023 | 0.004 | 3.00E-08 | 18 | 69876810 |
| rs249677 | A | C | 0.641 | 0.031 | 0.004 | 6.00E-13 | 5 | 142159774 |
| rs2546890 | A | G | 0.571 | 0.037 | 0.004 | 5.00E-19 | 5 | 159332892 |
| rs2585447 | C | T | 0.182 | 0.037 | 0.006 | 3.00E-10 | 20 | 54127898 |
| rs2590438 | G | T | 0.313 | 0.029 | 0.005 | 5.00E-09 | 3 | 187848180 |
| rs2705616 | C | G | 0.414 | 0.032 | 0.005 | 1.00E-11 | 4 | 86941244 |
| rs2726479 | C | T | 0.606 | 0.028 | 0.004 | 4.00E-11 | 4 | 105334432 |
| rs2836438 | A | G | 0.136 | 0.044 | 0.007 | 3.00E-10 | 21 | 38492803 |
| rs28703878 | G | A | 0.298 | 0.045 | 0.005 | 5.00E-22 | 8 | 78504987 |
| rs28834106 | T | C | 0.748 | 0.048 | 0.005 | 4.00E-19 | 19 | 10481468 |
| rs2986736 | C | T | 0.217 | 0.049 | 0.006 | 9.00E-17 | 1 | 6452487 |
| rs3184504 | T | C | 0.45 | 0.027 | 0.004 | 4.00E-11 | 12 | 111446804 |
| rs32658 | T | G | 0.379 | 0.027 | 0.005 | 3.00E-08 | 5 | 119367967 |
| rs34026809 | G | C | 0.929 | 0.038 | 0.005 | 8.00E-17 | 11 | 118609980 |
| rs34536443 | G | C | 0.96 | 0.081 | 0.012 | 3.00E-11 | 19 | 10352442 |
| rs34681760 | C | T | 0.682 | 0.034 | 0.005 | 2.00E-11 | 5 | 6712721 |
| rs34695601 | T | C | 0.727 | 0.035 | 0.005 | 6.00E-13 | 14 | 75547955 |
| rs34947566 | C | A | 0.823 | 0.059 | 0.006 | 1.00E-23 | 16 | 11319069 |
| rs35218683 | T | C | 0.513 | 0.033 | 0.006 | 1.00E-08 | 11 | 321138 |
| rs354033 | G | A | 0.758 | 0.034 | 0.006 | 8.00E-10 | 7 | 149592373 |
| rs35486093 | G | A | 0.121 | 0.082 | 0.007 | 2.00E-31 | 1 | 85264137 |
| rs35540610 | C | T | 0.283 | 0.058 | 0.005 | 3.00E-33 | 2 | 230257114 |
| rs35703946 | G | A | 0.869 | 0.054 | 0.006 | 3.00E-17 | 16 | 85987899 |
| rs3737798 | A | G | 0.53 | 0.031 | 0.004 | 4.00E-14 | 1 | 160420194 |
| rs3809627 | C | A | 0.586 | 0.039 | 0.004 | 1.00E-21 | 16 | 30091839 |
| rs3923387 | T | C | 0.45 | 0.027 | 0.005 | 2.00E-08 | 8 | 143912625 |
| rs405343 | T | G | 0.177 | 0.042 | 0.005 | 3.00E-15 | 16 | 1017832 |
| rs4262739 | G | A | 0.581 | 0.028 | 0.004 | 4.00E-12 | 11 | 128551280 |
| rs4325907 | C | T | 0.379 | 0.031 | 0.005 | 2.00E-09 | 3 | 102030178 |
| rs438613 | C | T | 0.485 | 0.061 | 0.004 | 2.00E-49 | 3 | 28030595 |
| rs4409785 | C | T | 0.141 | 0.036 | 0.005 | 7.00E-12 | 11 | 95578258 |
| rs4728142 | A | G | 0.394 | 0.025 | 0.004 | 3.00E-09 | 7 | 128933913 |
| rs4796224 | G | A | 0.546 | 0.038 | 0.005 | 1.00E-15 | 17 | 36486677 |
| rs4808760 | C | G | 0.717 | 0.049 | 0.005 | 6.00E-25 | 19 | 18191169 |
| rs4812772 | C | T | 0.707 | 0.031 | 0.005 | 6.00E-09 | 20 | 43950411 |
| rs4820955 | A | T | 0.742 | 0.027 | 0.005 | 1.00E-08 | 22 | 31226553 |
| rs483180 | C | G | 0.657 | 0.036 | 0.004 | 1.00E-17 | 1 | 119724882 |
| rs4896153 | T | A | 0.399 | 0.047 | 0.004 | 3.00E-29 | 6 | 135512325 |
| rs4939490 | G | C | 0.374 | 0.051 | 0.004 | 2.00E-29 | 11 | 61026179 |
| rs4940730 | A | G | 0.505 | 0.026 | 0.004 | 4.00E-10 | 18 | 58602505 |
| rs531612 | T | C | 0.47 | 0.029 | 0.005 | 1.00E-09 | 11 | 65937961 |
| rs55858457 | T | G | 0.338 | 0.028 | 0.005 | 3.00E-08 | 7 | 2403667 |
| rs56095240 | A | T | 0.172 | 0.045 | 0.007 | 1.00E-09 | 11 | 95688666 |
| rs570429157 | G | A | 0.02 | 0.109 | 0.016 | 9.00E-12 | 11 | 14868316 |
| rs57116599 | G | A | 0.773 | 0.039 | 0.006 | 1.00E-11 | 2 | 112013222 |
| rs5756405 | A | G | 0.535 | 0.027 | 0.004 | 5.00E-11 | 22 | 36914912 |
| rs58166386 | G | A | 0.333 | 0.045 | 0.004 | 4.00E-24 | 19 | 16448610 |
| rs58394161 | C | T | 0.157 | 0.054 | 0.007 | 1.00E-14 | 1 | 92474402 |
| rs59655222 | T | C | 0.692 | 0.043 | 0.005 | 6.00E-21 | 1 | 200906769 |
| rs6020055 | A | G | 0.995 | 0.025 | 0.005 | 5.00E-08 | 20 | 49805558 |
| rs6032662 | C | T | 0.227 | 0.041 | 0.005 | 5.00E-19 | 20 | 46105671 |
| rs60600003 | G | T | 0.081 | 0.059 | 0.007 | 5.00E-19 | 7 | 37342861 |
| rs6072343 | A | G | 0.141 | 0.034 | 0.006 | 3.00E-09 | 20 | 41339548 |
| rs61708525 | G | A | 0.349 | 0.029 | 0.005 | 2.00E-08 | 12 | 94267677 |
| rs61863928 | G | T | 0.641 | 0.037 | 0.005 | 3.00E-16 | 10 | 62689789 |
| rs61884005 | C | G | 0.874 | 0.040 | 0.007 | 6.00E-09 | 11 | 14381384 |
| rs62013236 | C | T | 0.833 | 0.044 | 0.005 | 9.00E-17 | 15 | 78955140 |
| rs62420820 | A | G | 0.167 | 0.059 | 0.005 | 9.00E-36 | 6 | 137116920 |
| rs631204 | A | C | 0.439 | 0.046 | 0.004 | 5.00E-25 | 6 | 137638318 |
| rs6427540 | C | T | 0.854 | 0.044 | 0.007 | 1.00E-09 | 1 | 160664798 |
| rs6496663 | C | A | 0.328 | 0.037 | 0.005 | 1.00E-12 | 15 | 90344352 |
| rs6498163 | C | T | 0.414 | 0.077 | 0.004 | 4.00E-71 | 16 | 11213951 |
| rs6533052 | A | G | 0.475 | 0.039 | 0.005 | 7.00E-16 | 4 | 102990624 |
| rs6564681 | C | T | 0.308 | 0.034 | 0.004 | 4.00E-14 | 16 | 79618823 |
| rs6589706 | A | G | 0.47 | 0.045 | 0.004 | 5.00E-26 | 11 | 118877104 |
| rs6589939 | G | A | 0.434 | 0.035 | 0.005 | 2.00E-12 | 11 | 122647817 |
| rs6670198 | T | C | 0.672 | 0.056 | 0.004 | 2.00E-36 | 1 | 2589088 |
| rs6672420 | A | T | 0.546 | 0.027 | 0.004 | 1.00E-09 | 1 | 24964519 |
| rs67111717 | G | A | 0.359 | 0.043 | 0.005 | 3.00E-21 | 5 | 177363161 |
| rs6738544 | C | A | 0.631 | 0.031 | 0.004 | 2.00E-13 | 2 | 191124630 |
| rs6742 | C | T | 0.768 | 0.060 | 0.008 | 4.00E-14 | 20 | 63743088 |
| rs6789653 | G | A | 0.697 | 0.025 | 0.004 | 1.00E-09 | 3 | 141432148 |
| rs6837324 | G | A | 0.419 | 0.028 | 0.004 | 1.00E-11 | 4 | 48125245 |
| rs6880809 | T | A | 0.47 | 0.030 | 0.004 | 1.00E-11 | 5 | 40429250 |
| rs6911131 | G | A | 0.061 | 0.050 | 0.008 | 1.00E-10 | 6 | 143544084 |
| rs6990534 | G | A | 0.712 | 0.042 | 0.005 | 6.00E-20 | 8 | 127801845 |
| rs701006 | G | A | 0.591 | 0.051 | 0.004 | 1.00E-30 | 12 | 57713053 |
| rs719316 | T | C | 0.52 | 0.030 | 0.004 | 2.00E-13 | 6 | 16672529 |
| rs7222450 | A | G | 0.495 | 0.026 | 0.004 | 2.00E-10 | 17 | 45330304 |
| rs7260482 | C | A | 0.232 | 0.033 | 0.005 | 1.00E-09 | 19 | 44640642 |
| rs72922276 | G | A | 0.864 | 0.066 | 0.008 | 1.00E-15 | 1 | 64963636 |
| rs72928038 | A | G | 0.182 | 0.062 | 0.006 | 8.00E-29 | 6 | 90267049 |
| rs72989863 | G | A | 0.601 | 0.028 | 0.005 | 6.00E-09 | 4 | 163572655 |
| rs73414214 | C | A | 0.939 | 0.052 | 0.008 | 6.00E-10 | 7 | 106066016 |
| rs735542 | A | G | 0.687 | 0.031 | 0.004 | 6.00E-13 | 8 | 127163451 |
| rs75937181 | T | G | 0.914 | 0.049 | 0.008 | 8.00E-10 | 3 | 121783015 |
| rs760517 | C | T | 0.631 | 0.029 | 0.004 | 5.00E-11 | 22 | 36862944 |
| rs7731626 | G | A | 0.631 | 0.039 | 0.005 | 4.00E-15 | 5 | 56148856 |
| rs77654077 | A | C | 0.965 | 0.045 | 0.005 | 9.00E-17 | 13 | 100026952 |
| rs7819665 | C | T | 0.242 | 0.051 | 0.005 | 3.00E-25 | 8 | 129177769 |
| rs7855251 | T | C | 0.697 | 0.032 | 0.005 | 1.00E-10 | 9 | 98105907 |
| rs78727559 | G | T | 0.025 | 0.062 | 0.010 | 3.00E-10 | 8 | 95851818 |
| rs7975763 | T | C | 0.232 | 0.036 | 0.005 | 3.00E-13 | 12 | 123119506 |
| rs7977720 | T | C | 0.414 | 0.041 | 0.004 | 5.00E-24 | 12 | 9713753 |
| rs79979643 | A | G | 0.172 | 0.054 | 0.007 | 1.00E-15 | 1 | 32738415 |
| rs802730 | T | C | 0.732 | 0.027 | 0.004 | 1.00E-09 | 6 | 127958959 |
| rs8062446 | T | C | 0.343 | 0.037 | 0.006 | 4.00E-09 | 16 | 57043182 |
| rs883871 | A | G | 0.172 | 0.052 | 0.007 | 9.00E-14 | 17 | 40096407 |
| rs9308424 | G | A | 0.687 | 0.039 | 0.005 | 9.00E-14 | 1 | 212704434 |
| rs9568402 | T | A | 0.126 | 0.037 | 0.006 | 5.00E-09 | 13 | 50387821 |
| rs9591325 | T | C | 0.939 | 0.089 | 0.010 | 1.00E-19 | 13 | 50237084 |
| rs9610458 | T | C | 0.571 | 0.037 | 0.004 | 1.00E-19 | 22 | 21851064 |
| rs962052 | C | T | 0.268 | 0.026 | 0.005 | 8.00E-09 | 2 | 150787689 |
| rs9808753 | G | A | 0.141 | 0.034 | 0.006 | 2.00E-09 | 21 | 33415005 |
| rs983494 | G | A | 0.742 | 0.036 | 0.005 | 6.00E-16 | 1 | 160734175 |
| rs9843355 | G | A | 0.849 | 0.062 | 0.005 | 4.00E-30 | 3 | 119509661 |
| rs9863496 | C | T | 0.258 | 0.032 | 0.004 | 3.00E-14 | 3 | 18757356 |
| rs9878602 | T | G | 0.515 | 0.041 | 0.005 | 2.00E-18 | 3 | 71486187 |
| rs9900529 | C | G | 0.187 | 0.027 | 0.005 | 5.00E-08 | 17 | 75339695 |
| rs9909593 | G | A | 0.475 | 0.034 | 0.004 | 9.00E-17 | 17 | 39813896 |
| rs9955954 | A | G | 0.783 | 0.037 | 0.006 | 5.00E-11 | 18 | 58680812 |
| rs9992763 | G | T | 0.389 | 0.028 | 0.004 | 2.00E-11 | 4 | 108137562 |
| rs10063294 | G | A | 0.424 | 0.052 | 0.005 | 2.00E-28 | 5 | 35877403 |
| rs1014486 | C | T | 0.434 | 0.045 | 0.004 | 3.00E-28 | 3 | 159973324 |
| rs10191360 | T | C | 0.394 | 0.035 | 0.006 | 4.00E-10 | 2 | 136127109 |
| rs10230723 | A | T | 0.818 | 0.036 | 0.006 | 8.00E-11 | 7 | 50200284 |
| rs10245867 | T | G | 0.328 | 0.029 | 0.004 | 1.00E-11 | 7 | 28102567 |
| rs1026916 | A | G | 0.374 | 0.047 | 0.004 | 2.00E-28 | 17 | 42377817 |
| rs10271373 | A | C | 0.54 | 0.024 | 0.004 | 3.00E-09 | 7 | 139045049 |
| rs1076928 | T | C | 0.414 | 0.053 | 0.006 | 3.00E-19 | 6 | 36380912 |
| rs1077667 | C | T | 0.783 | 0.062 | 0.005 | 8.00E-33 | 19 | 6668961 |
| rs10801908 | C | T | 0.859 | 0.114 | 0.006 | 5.00E-70 | 1 | 116547871 |
| rs1087056 | A | G | 0.424 | 0.037 | 0.004 | 3.00E-19 | 10 | 31106832 |
| rs10936602 | T | C | 0.732 | 0.032 | 0.005 | 2.00E-11 | 3 | 169818849 |
| rs10951042 | C | T | 0.399 | 0.042 | 0.005 | 1.00E-18 | 7 | 3099783 |
| rs10951154 | C | T | 0.157 | 0.033 | 0.005 | 3.00E-10 | 7 | 27095695 |
| rs11079784 | C | T | 0.515 | 0.044 | 0.004 | 2.00E-27 | 17 | 47624914 |
| rs11083862 | A | T | 0.455 | 0.032 | 0.005 | 2.00E-09 | 19 | 47135282 |
| rs11125803 | C | T | 0.293 | 0.030 | 0.005 | 5.00E-10 | 2 | 24829308 |
| rs1112718 | A | G | 0.606 | 0.036 | 0.004 | 2.00E-17 | 10 | 92719350 |
| rs111430408 | C | T | 0.934 | 0.063 | 0.010 | 6.00E-11 | 3 | 100848597 |
| rs111635774 | C | T | 0.914 | 0.046 | 0.005 | 1.00E-17 | 6 | 14691215 |
| rs11231749 | C | T | 0.318 | 0.029 | 0.004 | 1.00E-11 | 11 | 64327706 |
| rs112344141 | G | T | 0.025 | 0.073 | 0.013 | 2.00E-08 | 1 | 154983036 |
| rs11256593 | T | C | 0.561 | 0.081 | 0.005 | 3.00E-65 | 10 | 6075359 |
| rs112741635 | G | A | 0.581 | 0.029 | 0.005 | 4.00E-08 | 1 | 24207504 |
| rs11542663 | A | C | 0.647 | 0.032 | 0.005 | 3.00E-10 | 6 | 119215402 |
| rs11578655 | G | T | 0.131 | 0.036 | 0.007 | 3.00E-08 | 1 | 100947346 |
| rs116877451 | A | G | 0.96 | 0.038 | 0.005 | 3.00E-16 | 7 | 50328339 |
| rs116899835 | C | T | 0.955 | 0.124 | 0.011 | 2.00E-30 | 14 | 88523488 |
| rs11749040 | A | G | 0.157 | 0.061 | 0.006 | 5.00E-25 | 5 | 40396323 |
| rs1177228 | G | A | 0.758 | 0.040 | 0.004 | 2.00E-19 | 2 | 61015275 |
| rs11852059 | C | A | 0.157 | 0.039 | 0.006 | 1.00E-09 | 14 | 51839373 |
| rs11899404 | C | T | 0.51 | 0.023 | 0.004 | 2.00E-08 | 2 | 12467767 |
| rs11919880 | A | G | 0.525 | 0.025 | 0.004 | 8.00E-10 | 3 | 32920559 |
| rs12133753 | C | T | 0.828 | 0.053 | 0.007 | 2.00E-16 | 1 | 91756532 |
| rs12147246 | A | G | 0.379 | 0.039 | 0.005 | 2.00E-15 | 14 | 102799507 |
| rs12211604 | G | A | 0.359 | 0.028 | 0.005 | 2.00E-08 | 6 | 7099796 |
| rs12365699 | G | A | 0.823 | 0.051 | 0.006 | 2.00E-19 | 11 | 118872577 |
| rs12434551 | A | T | 0.535 | 0.034 | 0.004 | 4.00E-17 | 14 | 68786647 |
| rs12478539 | G | C | 0.722 | 0.048 | 0.005 | 2.00E-24 | 2 | 43128185 |
| rs1250551 | T | G | 0.384 | 0.042 | 0.004 | 2.00E-23 | 10 | 79299578 |
| rs12588969 | G | C | 0.394 | 0.032 | 0.005 | 2.00E-09 | 14 | 102764421 |
| rs12609500 | C | T | 0.753 | 0.025 | 0.004 | 5.00E-09 | 19 | 11063252 |
| rs12614091 | A | T | 0.788 | 0.032 | 0.005 | 6.00E-12 | 2 | 203768138 |
| rs12622670 | T | C | 0.515 | 0.037 | 0.005 | 5.00E-16 | 2 | 68419404 |
| rs12832171 | C | G | 0.992 | 0.067 | 0.011 | 4.00E-10 | 12 | 6332456 |
| rs12925972 | C | T | 0.53 | 0.044 | 0.005 | 1.00E-19 | 16 | 79077400 |
| rs12971909 | A | G | 0.384 | 0.030 | 0.005 | 6.00E-09 | 19 | 4466469 |
| rs13066789 | C | T | 0.444 | 0.028 | 0.005 | 1.00E-08 | 3 | 188269836 |
| rs13136820 | C | T | 0.308 | 0.032 | 0.005 | 8.00E-10 | 4 | 40305547 |
| rs1323292 | A | G | 0.803 | 0.065 | 0.005 | 4.00E-33 | 1 | 192571891 |
| rs13327021 | C | T | 0.611 | 0.035 | 0.004 | 2.00E-16 | 3 | 27741524 |
| rs13385171 | C | T | 0.546 | 0.029 | 0.004 | 3.00E-11 | 2 | 65434709 |
| rs13414105 | C | A | 0.495 | 0.038 | 0.006 | 7.00E-10 | 2 | 30249576 |
| rs1365120 | C | T | 0.121 | 0.040 | 0.007 | 4.00E-08 | 11 | 36416525 |
| rs137955 | T | C | 0.374 | 0.024 | 0.004 | 1.00E-08 | 22 | 39895803 |
| rs138433213 | T | G | 0.99 | 0.134 | 0.023 | 3.00E-09 | 3 | 112693983 |
| rs1399180 | C | T | 0.818 | 0.042 | 0.006 | 7.00E-11 | 10 | 8056756 |
| rs140522 | T | C | 0.318 | 0.048 | 0.005 | 1.00E-21 | 22 | 50532837 |
| rs1415069 | G | C | 0.232 | 0.031 | 0.006 | 3.00E-08 | 1 | 92961312 |
| rs146566517 | T | C | 0.025 | 0.038 | 0.005 | 5.00E-16 | 16 | 11353879 |
| rs1465697 | T | C | 0.263 | 0.040 | 0.005 | 3.00E-18 | 19 | 49333989 |
| rs149114341 | G | A | 0.97 | 0.102 | 0.016 | 9.00E-11 | 11 | 118783424 |
| rs17051321 | T | C | 0.227 | 0.035 | 0.005 | 1.00E-10 | 4 | 121198294 |
| rs1738074 | C | T | 0.525 | 0.054 | 0.004 | 3.00E-35 | 6 | 159044945 |
| rs17724508 | T | C | 0.96 | 0.058 | 0.008 | 6.00E-14 | 16 | 79316307 |
| rs17741873 | G | T | 0.818 | 0.028 | 0.005 | 5.00E-08 | 10 | 73894042 |
| rs17780048 | C | T | 0.944 | 0.042 | 0.006 | 5.00E-12 | 6 | 137858009 |
| rs1800693 | C | T | 0.49 | 0.060 | 0.004 | 2.00E-47 | 12 | 6330843 |
| rs1801133 | G | A | 0.702 | 0.044 | 0.006 | 2.00E-15 | 1 | 11796321 |
| rs200866143 | C | G | 0.522 | 0.056 | 0.004 | 7.00E-43 | 3 | 121765368 |
| rs2084007 | C | T | 0.53 | 0.035 | 0.005 | 2.00E-13 | 5 | 134555592 |
| rs2150879 | G | A | 0.439 | 0.048 | 0.004 | 4.00E-31 | 17 | 59781849 |
| rs2248137 | C | G | 0.601 | 0.038 | 0.004 | 2.00E-19 | 20 | 54173204 |
| rs2269434 | C | T | 0.333 | 0.038 | 0.005 | 1.00E-13 | 11 | 47338861 |
| rs2289746 | C | T | 0.606 | 0.035 | 0.005 | 5.00E-12 | 3 | 105737111 |
| rs2317231 | G | T | 0.525 | 0.034 | 0.004 | 3.00E-16 | 1 | 157716547 |
| rs2327586 | T | C | 0.737 | 0.043 | 0.005 | 9.00E-20 | 6 | 135174088 |
| rs2331964 | C | T | 0.657 | 0.039 | 0.004 | 5.00E-20 | 3 | 121824051 |
| rs2364485 | A | C | 0.172 | 0.048 | 0.005 | 2.00E-20 | 12 | 6405797 |
| rs244656 | A | T | 0.899 | 0.046 | 0.006 | 3.00E-14 | 5 | 134114136 |
| rs2469434 | C | T | 0.364 | 0.023 | 0.004 | 3.00E-08 | 18 | 69876810 |
| rs249677 | A | C | 0.641 | 0.031 | 0.004 | 6.00E-13 | 5 | 142159774 |
| rs2546890 | A | G | 0.571 | 0.037 | 0.004 | 5.00E-19 | 5 | 159332892 |
| rs2585447 | C | T | 0.182 | 0.037 | 0.006 | 3.00E-10 | 20 | 54127898 |
| rs2590438 | G | T | 0.313 | 0.029 | 0.005 | 5.00E-09 | 3 | 187848180 |
| rs2705616 | C | G | 0.414 | 0.032 | 0.005 | 1.00E-11 | 4 | 86941244 |
| rs2726479 | C | T | 0.606 | 0.028 | 0.004 | 4.00E-11 | 4 | 105334432 |
| rs2836438 | A | G | 0.136 | 0.044 | 0.007 | 3.00E-10 | 21 | 38492803 |
| rs28703878 | G | A | 0.298 | 0.045 | 0.005 | 5.00E-22 | 8 | 78504987 |
| rs28834106 | T | C | 0.748 | 0.048 | 0.005 | 4.00E-19 | 19 | 10481468 |
| rs2986736 | C | T | 0.217 | 0.049 | 0.006 | 9.00E-17 | 1 | 6452487 |
| rs3184504 | T | C | 0.45 | 0.027 | 0.004 | 4.00E-11 | 12 | 111446804 |
| rs32658 | T | G | 0.379 | 0.027 | 0.005 | 3.00E-08 | 5 | 119367967 |
| rs34026809 | G | C | 0.929 | 0.038 | 0.005 | 8.00E-17 | 11 | 118609980 |
| rs34536443 | G | C | 0.96 | 0.081 | 0.012 | 3.00E-11 | 19 | 10352442 |
| rs34681760 | C | T | 0.682 | 0.034 | 0.005 | 2.00E-11 | 5 | 6712721 |
| rs34695601 | T | C | 0.727 | 0.035 | 0.005 | 6.00E-13 | 14 | 75547955 |
| rs34947566 | C | A | 0.823 | 0.059 | 0.006 | 1.00E-23 | 16 | 11319069 |
| rs35218683 | T | C | 0.513 | 0.033 | 0.006 | 1.00E-08 | 11 | 321138 |
| rs354033 | G | A | 0.758 | 0.034 | 0.006 | 8.00E-10 | 7 | 149592373 |
| rs35486093 | G | A | 0.121 | 0.082 | 0.007 | 2.00E-31 | 1 | 85264137 |
| rs35540610 | C | T | 0.283 | 0.058 | 0.005 | 3.00E-33 | 2 | 230257114 |
| rs35703946 | G | A | 0.869 | 0.054 | 0.006 | 3.00E-17 | 16 | 85987899 |
| rs3737798 | A | G | 0.53 | 0.031 | 0.004 | 4.00E-14 | 1 | 160420194 |
| rs3809627 | C | A | 0.586 | 0.039 | 0.004 | 1.00E-21 | 16 | 30091839 |
| rs3923387 | T | C | 0.45 | 0.027 | 0.005 | 2.00E-08 | 8 | 143912625 |
| rs405343 | T | G | 0.177 | 0.042 | 0.005 | 3.00E-15 | 16 | 1017832 |
| rs4262739 | G | A | 0.581 | 0.028 | 0.004 | 4.00E-12 | 11 | 128551280 |
| rs4325907 | C | T | 0.379 | 0.031 | 0.005 | 2.00E-09 | 3 | 102030178 |
| rs438613 | C | T | 0.485 | 0.061 | 0.004 | 2.00E-49 | 3 | 28030595 |
| rs4409785 | C | T | 0.141 | 0.036 | 0.005 | 7.00E-12 | 11 | 95578258 |
| rs4728142 | A | G | 0.394 | 0.025 | 0.004 | 3.00E-09 | 7 | 128933913 |
| rs4796224 | G | A | 0.546 | 0.038 | 0.005 | 1.00E-15 | 17 | 36486677 |
| rs4808760 | C | G | 0.717 | 0.049 | 0.005 | 6.00E-25 | 19 | 18191169 |
| rs4812772 | C | T | 0.707 | 0.031 | 0.005 | 6.00E-09 | 20 | 43950411 |
| rs4820955 | A | T | 0.742 | 0.027 | 0.005 | 1.00E-08 | 22 | 31226553 |
| rs483180 | C | G | 0.657 | 0.036 | 0.004 | 1.00E-17 | 1 | 119724882 |
| rs4896153 | T | A | 0.399 | 0.047 | 0.004 | 3.00E-29 | 6 | 135512325 |
| rs4939490 | G | C | 0.374 | 0.051 | 0.004 | 2.00E-29 | 11 | 61026179 |
| rs4940730 | A | G | 0.505 | 0.026 | 0.004 | 4.00E-10 | 18 | 58602505 |
| rs531612 | T | C | 0.47 | 0.029 | 0.005 | 1.00E-09 | 11 | 65937961 |
| rs55858457 | T | G | 0.338 | 0.028 | 0.005 | 3.00E-08 | 7 | 2403667 |
| rs56095240 | A | T | 0.172 | 0.045 | 0.007 | 1.00E-09 | 11 | 95688666 |
| rs570429157 | G | A | 0.02 | 0.109 | 0.016 | 9.00E-12 | 11 | 14868316 |
| rs57116599 | G | A | 0.773 | 0.039 | 0.006 | 1.00E-11 | 2 | 112013222 |
| rs5756405 | A | G | 0.535 | 0.027 | 0.004 | 5.00E-11 | 22 | 36914912 |
| rs58166386 | G | A | 0.333 | 0.045 | 0.004 | 4.00E-24 | 19 | 16448610 |
| rs58394161 | C | T | 0.157 | 0.054 | 0.007 | 1.00E-14 | 1 | 92474402 |
| rs59655222 | T | C | 0.692 | 0.043 | 0.005 | 6.00E-21 | 1 | 200906769 |
| rs6020055 | A | G | 0.995 | 0.025 | 0.005 | 5.00E-08 | 20 | 49805558 |
| rs6032662 | C | T | 0.227 | 0.041 | 0.005 | 5.00E-19 | 20 | 46105671 |
| rs60600003 | G | T | 0.081 | 0.059 | 0.007 | 5.00E-19 | 7 | 37342861 |
| rs6072343 | A | G | 0.141 | 0.034 | 0.006 | 3.00E-09 | 20 | 41339548 |
| rs61708525 | G | A | 0.349 | 0.029 | 0.005 | 2.00E-08 | 12 | 94267677 |
| rs61863928 | G | T | 0.641 | 0.037 | 0.005 | 3.00E-16 | 10 | 62689789 |
| rs61884005 | C | G | 0.874 | 0.040 | 0.007 | 6.00E-09 | 11 | 14381384 |
| rs62013236 | C | T | 0.833 | 0.044 | 0.005 | 9.00E-17 | 15 | 78955140 |
| **Parkinson's disease** | | | | | | | | |
| rs10221156 | A | G | 0.0932 | -0.1156 | 0.017857 | 1.00E-10 | 16 | 52935514 |
| rs10513789 | T | G | 0.8112 | 0.1485 | 0.012755 | 1.00E-34 | 3 | 183042285 |
| rs10748818 | A | G | 0.8514 | -0.079 | 0.012755 | 1.00E-09 | 10 | 102255522 |
| rs10756907 | A | G | 0.7666 | -0.0926 | 0.010969 | 5.00E-17 | 9 | 17727067 |
| rs10797576 | T | C | 0.1403 | 0.1114 | 0.013265 | 7.00E-17 | 1 | 232528865 |
| rs10847864 | T | G | 0.364 | 0.1478 | 0.010204 | 1.00E-37 | 12 | 122842051 |
| rs11150601 | A | G | 0.6442 | 0.0907 | 0.009949 | 5.00E-20 | 16 | 30966478 |
| rs11158026 | T | C | 0.3245 | -0.0842 | 0.010204 | 2.00E-16 | 14 | 54882151 |
| rs504594 | A | C | 0.163 | -0.1676 | 0.015306 | 7.00E-28 | 6 | 32610995 |
| rs114138760 | C | G | 0.0112 | 0.2812 | 0.045918 | 4.00E-09 | 1 | 154925709 |
| rs11557080 | A | G | 0.1389 | 0.1315 | 0.012755 | 3.00E-22 | 1 | 205768611 |
| rs11578699 | T | C | 0.1949 | -0.0704 | 0.01199 | 4.00E-09 | 1 | 171750629 |
| rs11610045 | A | G | 0.4896 | 0.0601 | 0.009439 | 2.00E-10 | 12 | 132487182 |
| rs11658976 | A | G | 0.5802 | -0.0624 | 0.01148 | 4.00E-08 | 17 | 46789439 |
| rs11683001 | A | T | 0.337 | 0.0705 | 0.009949 | 8.00E-13 | 2 | 101780501 |
| rs117073808 | T | C | 0.9809 | -0.3955 | 0.043367 | 2.00E-20 | 12 | 40528770 |
| rs11707416 | A | T | 0.3672 | -0.0627 | 0.009694 | 1.00E-10 | 3 | 151391177 |
| rs117615688 | A | G | 0.067 | -0.2324 | 0.028061 | 7.00E-16 | 17 | 45720942 |
| rs117896735 | A | G | 0.0166 | 0.4354 | 0.038265 | 2.00E-28 | 10 | 119776815 |
| rs11950533 | A | C | 0.102 | -0.0916 | 0.015816 | 7.00E-09 | 5 | 134863415 |
| rs12147950 | T | C | 0.4376 | -0.0529 | 0.009694 | 4.00E-08 | 14 | 37520065 |
| rs12283611 | A | C | 0.4148 | -0.0645 | 0.009949 | 3.00E-10 | 11 | 83776234 |
| rs12456492 | A | G | 0.6816 | -0.0983 | 0.009949 | 4.00E-23 | 18 | 43093415 |
| rs12497850 | T | G | 0.6476 | 0.0636 | 0.009949 | 1.00E-10 | 3 | 48711556 |
| rs12528068 | T | C | 0.2844 | 0.0657 | 0.010204 | 2.00E-10 | 6 | 71778059 |
| rs12600861 | A | C | 0.6484 | -0.0565 | 0.009949 | 1.00E-08 | 17 | 7452302 |
| rs1293298 | A | C | 0.7444 | 0.093 | 0.011224 | 4.00E-16 | 8 | 11854934 |
| rs12951632 | T | C | 0.7349 | 0.0642 | 0.010714 | 1.00E-09 | 17 | 42588995 |
| rs13117519 | T | C | 0.1744 | 0.0875 | 0.0125 | 1.00E-12 | 4 | 113447909 |
| rs13294100 | T | G | 0.3422 | -0.0859 | 0.010204 | 9.00E-18 | 9 | 17579692 |
| rs138017112 | C | G | 0.0142 | 0.413 | 0.061224 | 7.00E-12 | 12 | 41901977 |
| rs141128804 | T | C | 0.0173 | 0.3468 | 0.045918 | 6.00E-14 | 12 | 40542837 |
| rs144755950 | T | C | 0.9856 | -0.3993 | 0.061224 | 2.00E-10 | 12 | 42141601 |
| rs1450522 | A | G | 0.6742 | -0.0616 | 0.009949 | 5.00E-10 | 3 | 161359842 |
| rs1474055 | T | C | 0.1312 | 0.1796 | 0.015306 | 3.00E-39 | 2 | 168253884 |
| rs17686238 | T | G | 0.1071 | -0.1896 | 0.017857 | 8.00E-27 | 17 | 45339907 |
| rs181609621 | A | C | 0.9882 | -0.4232 | 0.063776 | 8.00E-11 | 12 | 32657825 |
| rs1867598 | A | G | 0.9019 | -0.1554 | 0.017857 | 3.00E-23 | 5 | 60842132 |
| rs1941685 | T | G | 0.4983 | 0.0531 | 0.009439 | 2.00E-08 | 18 | 33724354 |
| rs199351 | A | C | 0.5939 | 0.1016 | 0.009439 | 5.00E-26 | 7 | 23260430 |
| rs199453 | T | C | 0.231 | -0.2045 | 0.012755 | 9.00E-67 | 17 | 46723580 |
| rs2042477 | A | T | 0.2422 | -0.0657 | 0.01148 | 1.00E-08 | 2 | 95335195 |
| rs2086641 | T | C | 0.7225 | -0.0605 | 0.010459 | 2.00E-08 | 8 | 129889663 |
| rs2248244 | A | G | 0.2828 | 0.0714 | 0.010714 | 3.00E-11 | 21 | 37480059 |
| rs2251086 | T | C | 0.1417 | -0.1186 | 0.01352 | 6.00E-18 | 15 | 61705186 |
| rs2269906 | A | C | 0.6531 | 0.0631 | 0.010204 | 6.00E-10 | 17 | 44216969 |
| rs2280104 | T | C | 0.3604 | 0.0556 | 0.009949 | 1.00E-08 | 8 | 22668467 |
| rs2295545 | T | C | 0.6105 | 0.0622 | 0.009694 | 8.00E-11 | 20 | 3184040 |
| rs26431 | C | G | 0.7025 | 0.0621 | 0.010204 | 2.00E-09 | 5 | 103030090 |
| rs2904880 | C | G | 0.3094 | -0.065 | 0.010714 | 8.00E-10 | 16 | 28933075 |
| rs3104783 | A | C | 0.4343 | 0.0668 | 0.009439 | 1.00E-12 | 16 | 52602330 |
| rs34025766 | A | T | 0.1589 | -0.0839 | 0.013265 | 3.00E-10 | 4 | 17967188 |
| rs34311866 | T | C | 0.8065 | -0.2126 | 0.012755 | 1.00E-69 | 4 | 958159 |
| rs356182 | A | G | 0.6278 | -0.2774 | 0.010204 | 4.00E-154 | 4 | 89704960 |
| rs356203 | T | C | 0.6149 | -0.2504 | 0.010204 | 5.00E-149 | 4 | 89744890 |
| rs356228 | C | G | 0.4589 | 0.1503 | 0.010204 | 1.00E-48 | 4 | 89685975 |
| rs35643925 | A | G | 0.0195 | 0.389 | 0.040816 | 8.00E-22 | 1 | 156185069 |
| rs35749011 | A | G | 0.0169 | 0.6068 | 0.033163 | 2.00E-70 | 1 | 155162560 |
| rs3742785 | A | C | 0.7866 | 0.0707 | 0.011735 | 2.00E-09 | 14 | 74906331 |
| rs3802920 | T | G | 0.2054 | 0.1073 | 0.011735 | 6.00E-20 | 11 | 133917106 |
| rs4101061 | A | G | 0.7107 | -0.0912 | 0.010204 | 5.00E-19 | 4 | 76226816 |
| rs4140646 | A | G | 0.2081 | 0.0833 | 0.01199 | 6.00E-12 | 6 | 27771022 |
| rs4653767 | T | C | 0.7196 | 0.0833 | 0.010459 | 1.00E-15 | 1 | 226728377 |
| rs4698412 | A | G | 0.5529 | 0.1035 | 0.009439 | 2.00E-28 | 4 | 15735725 |
| rs4771268 | T | C | 0.2295 | 0.0675 | 0.010969 | 1.00E-09 | 13 | 97212767 |
| rs4954162 | A | G | 0.1642 | -0.0746 | 0.01301 | 2.00E-08 | 2 | 134681219 |
| rs5019538 | A | G | 0.6792 | -0.1565 | 0.012755 | 1.00E-36 | 4 | 89715479 |
| rs55818311 | T | C | 0.6937 | -0.0696 | 0.010969 | 4.00E-10 | 19 | 2341049 |
| rs55961674 | T | C | 0.1722 | 0.0861 | 0.012755 | 1.00E-11 | 3 | 122478045 |
| rs57891859 | A | G | 0.7185 | 0.0807 | 0.010714 | 5.00E-14 | 2 | 134707046 |
| rs61169879 | T | C | 0.1641 | 0.082 | 0.013265 | 9.00E-10 | 17 | 61840005 |
| rs620513 | T | G | 0.2682 | -0.0856 | 0.010969 | 3.00E-15 | 8 | 16840084 |
| rs62053943 | T | C | 0.1552 | -0.27 | 0.015306 | 4.00E-68 | 17 | 45666837 |
| rs62333164 | A | G | 0.3264 | -0.0638 | 0.009949 | 2.00E-10 | 4 | 169662006 |
| rs6476434 | T | C | 0.7336 | -0.0615 | 0.010459 | 7.00E-09 | 9 | 34046393 |
| rs6497339 | A | T | 0.4536 | 0.063 | 0.009694 | 3.00E-11 | 16 | 19266171 |
| rs6500328 | A | G | 0.5985 | 0.0586 | 0.009694 | 2.00E-09 | 16 | 50702745 |
| rs6658353 | C | G | 0.5011 | 0.065 | 0.009184 | 6.00E-12 | 1 | 161499264 |
| rs666463 | A | T | 0.8328 | 0.076 | 0.012755 | 3.00E-09 | 17 | 78429399 |
| rs6808178 | T | C | 0.3794 | 0.0658 | 0.009694 | 8.00E-12 | 3 | 28664199 |
| rs6825004 | C | G | 0.6912 | 0.0622 | 0.010204 | 1.00E-09 | 4 | 76189212 |
| rs6854006 | T | C | 0.3632 | -0.0912 | 0.009694 | 6.00E-21 | 4 | 76276901 |
| rs6875262 | T | C | 0.0986 | 0.1141 | 0.019898 | 1.00E-08 | 5 | 124774580 |
| rs7134559 | T | C | 0.404 | -0.0539 | 0.009694 | 4.00E-08 | 12 | 46025303 |
| rs7221167 | T | C | 0.581 | 0.1443 | 0.012755 | 1.00E-37 | 17 | 45855941 |
| rs7225002 | A | G | 0.6094 | 0.1351 | 0.007653 | 2.00E-40 | 17 | 46111701 |
| rs72840788 | G | A | 0.7845 | 0.0763 | 0.011224 | 2.00E-11 | 10 | 119656173 |
| rs73038319 | A | C | 0.9592 | -0.1693 | 0.02551 | 6.00E-13 | 3 | 18320267 |
| rs75859381 | T | C | 0.9673 | -0.2207 | 0.035714 | 1.00E-10 | 6 | 132889222 |
| rs76116224 | A | T | 0.9042 | 0.1104 | 0.019388 | 1.00E-08 | 2 | 17966582 |
| rs76763715 | T | C | 0.9953 | -0.7467 | 0.076531 | 2.00E-22 | 1 | 155235843 |
| rs76904798 | T | C | 0.1444 | 0.1439 | 0.012755 | 2.00E-28 | 12 | 40220632 |
| rs76949143 | A | T | 0.0507 | -0.1432 | 0.025255 | 1.00E-08 | 7 | 66544864 |
| rs77351827 | T | C | 0.1275 | 0.0802 | 0.013776 | 9.00E-09 | 20 | 6025395 |
| rs7938782 | A | G | 0.8776 | 0.087 | 0.014286 | 2.00E-09 | 11 | 10537230 |
| rs8087969 | T | G | 0.5496 | -0.0578 | 0.010204 | 1.00E-08 | 18 | 51157219 |
| rs823118 | T | C | 0.566 | 0.1066 | 0.009439 | 1.00E-29 | 1 | 205754444 |
| rs850738 | A | G | 0.6056 | -0.071 | 0.010714 | 1.00E-11 | 17 | 44357262 |
| rs873786 | T | C | 0.0988 | -0.1731 | 0.017857 | 2.00E-21 | 4 | 931588 |
| rs896435 | T | C | 0.6892 | 0.0735 | 0.009949 | 3.00E-13 | 10 | 15515407 |
| rs9261484 | T | C | 0.2451 | -0.0635 | 0.010969 | 2.00E-08 | 6 | 30140906 |
| rs9267659 | A | G | 0.2004 | 0.079 | 0.011735 | 4.00E-11 | 6 | 31878457 |
| rs9568188 | T | C | 0.7397 | 0.0617 | 0.010714 | 1.00E-08 | 13 | 49353596 |
| rs979812 | T | G | 0.4421 | 0.061 | 0.009184 | 6.00E-11 | 14 | 87997920 |
| rs9912362 | T | C | 0.3594 | 0.077 | 0.010204 | 2.00E-13 | 17 | 45706862 |
| rs997368 | A | G | 0.8049 | 0.0714 | 0.01199 | 2.00E-09 | 6 | 111922088 |
| **Periodontitis** | | | | | | | | |
| rs729876 | T | C | 0.8 | 0.21 | 0.04 | 1.00E-08 | 16 | 16p13.12 |
| rs11084095 | A | G | 0.41 | 0.16 | 0.03 | 5.00E-08 | 19 | 51623777 |
| rs9982623 | C | T | 0.86 | 0.20 | 0.04 | 9.00E-07 | 21 | 21q22.3 |
| rs9984417 | T | A | 0.61 | 0.15 | 0.03 | 9.00E-07 | 21 | 22474790 |
| rs6725179 | A | G | 0.313 | 0.14 | 0.03 | 3.00E-06 | 2 | 126759652 |
| rs1247559 | C | T | 0.818 | 0.17 | 0.04 | 4.00E-06 | 6 | 160782724 |
| rs7086701 | T | G | 0.49 | 0.14 | 0.03 | 1.00E-06 | 10 | 10298081 |
| rs886431 | G | A | 0.177 | 0.17 | 0.04 | 4.00E-06 | 12 | 47733232 |
| rs2833579 | G | A | 0.485 | 0.14 | 0.03 | 3.00E-06 | 21 | 31956217 |
| rs729876 | T | C | 0.8 | 0.21 | 0.04 | 1.00E-08 | 16 | 16p13.12 |
| rs11084095 | A | G | 0.41 | 0.16 | 0.03 | 5.00E-08 | 19 | 51623777 |
| rs9982623 | C | T | 0.86 | 0.20 | 0.04 | 9.00E-07 | 21 | 21q22.3 |
| rs9984417 | T | A | 0.61 | 0.15 | 0.03 | 9.00E-07 | 21 | 22474790 |
| rs6725179 | A | G | 0.313 | 0.14 | 0.03 | 3.00E-06 | 2 | 126759652 |
| rs1247559 | C | T | 0.818 | 0.17 | 0.04 | 4.00E-06 | 6 | 160782724 |
| rs7086701 | T | G | 0.49 | 0.14 | 0.03 | 1.00E-06 | 10 | 10298081 |
| rs886431 | G | A | 0.177 | 0.17 | 0.04 | 4.00E-06 | 12 | 47733232 |
| rs2833579 | G | A | 0.485 | 0.14 | 0.03 | 3.00E-06 | 21 | 31956217 |
| rs729876 | T | C | 0.8 | 0.21 | 0.04 | 1.00E-08 | 16 | 16p13.12 |
| rs11084095 | A | G | 0.41 | 0.16 | 0.03 | 5.00E-08 | 19 | 51623777 |
| rs9982623 | C | T | 0.86 | 0.20 | 0.04 | 9.00E-07 | 21 | 21q22.3 |
| rs9984417 | T | A | 0.61 | 0.15 | 0.03 | 9.00E-07 | 21 | 22474790 |
| rs6725179 | A | G | 0.313 | 0.14 | 0.03 | 3.00E-06 | 2 | 126759652 |
| rs1247559 | C | T | 0.818 | 0.17 | 0.04 | 4.00E-06 | 6 | 160782724 |
| rs7086701 | T | G | 0.49 | 0.14 | 0.03 | 1.00E-06 | 10 | 10298081 |
| rs886431 | G | A | 0.177 | 0.17 | 0.04 | 4.00E-06 | 12 | 47733232 |
| rs2833579 | G | A | 0.485 | 0.14 | 0.03 | 3.00E-06 | 21 | 31956217 |
| rs729876 | T | C | 0.8 | 0.21 | 0.04 | 1.00E-08 | 16 | 16p13.12 |
| rs11084095 | A | G | 0.41 | 0.16 | 0.03 | 5.00E-08 | 19 | 51623777 |
| rs9982623 | C | T | 0.86 | 0.20 | 0.04 | 9.00E-07 | 21 | 21q22.3 |
| rs9984417 | T | A | 0.61 | 0.15 | 0.03 | 9.00E-07 | 21 | 22474790 |
| rs6725179 | A | G | 0.313 | 0.14 | 0.03 | 3.00E-06 | 2 | 126759652 |
| rs1247559 | C | T | 0.818 | 0.17 | 0.04 | 4.00E-06 | 6 | 160782724 |
| rs7086701 | T | G | 0.49 | 0.14 | 0.03 | 1.00E-06 | 10 | 10298081 |
| rs886431 | G | A | 0.177 | 0.17 | 0.04 | 4.00E-06 | 12 | 47733232 |
| rs2833579 | G | A | 0.485 | 0.14 | 0.03 | 3.00E-06 | 21 | 31956217 |
| rs729876 | T | C | 0.8 | 0.21 | 0.04 | 1.00E-08 | 16 | 16p13.12 |
| rs11084095 | A | G | 0.41 | 0.16 | 0.03 | 5.00E-08 | 19 | 51623777 |
| rs9982623 | C | T | 0.86 | 0.20 | 0.04 | 9.00E-07 | 21 | 21q22.3 |
| rs9984417 | T | A | 0.61 | 0.15 | 0.03 | 9.00E-07 | 21 | 22474790 |
| rs6725179 | A | G | 0.313 | 0.14 | 0.03 | 3.00E-06 | 2 | 126759652 |
| rs1247559 | C | T | 0.818 | 0.17 | 0.04 | 4.00E-06 | 6 | 160782724 |
| rs7086701 | T | G | 0.49 | 0.14 | 0.03 | 1.00E-06 | 10 | 10298081 |
| rs886431 | G | A | 0.177 | 0.17 | 0.04 | 4.00E-06 | 12 | 47733232 |
| rs2833579 | G | A | 0.485 | 0.14 | 0.03 | 3.00E-06 | 21 | 31956217 |
| rs729876 | T | C | 0.8 | 0.21 | 0.04 | 1.00E-08 | 16 | 16p13.12 |
| rs11084095 | A | G | 0.41 | 0.16 | 0.03 | 5.00E-08 | 19 | 51623777 |
| rs9982623 | C | T | 0.86 | 0.20 | 0.04 | 9.00E-07 | 21 | 21q22.3 |
| rs9984417 | T | A | 0.61 | 0.15 | 0.03 | 9.00E-07 | 21 | 22474790 |
| rs6725179 | A | G | 0.313 | 0.14 | 0.03 | 3.00E-06 | 2 | 126759652 |
| rs1247559 | C | T | 0.818 | 0.17 | 0.04 | 4.00E-06 | 6 | 160782724 |
| rs7086701 | T | G | 0.49 | 0.14 | 0.03 | 1.00E-06 | 10 | 10298081 |
| rs886431 | G | A | 0.177 | 0.17 | 0.04 | 4.00E-06 | 12 | 47733232 |
| rs2833579 | G | A | 0.485 | 0.14 | 0.03 | 3.00E-06 | 21 | 31956217 |
| rs729876 | T | C | 0.8 | 0.21 | 0.04 | 1.00E-08 | 16 | 16p13.12 |
| rs11084095 | A | G | 0.41 | 0.16 | 0.03 | 5.00E-08 | 19 | 51623777 |
| rs9982623 | C | T | 0.86 | 0.20 | 0.04 | 9.00E-07 | 21 | 21q22.3 |
| rs9984417 | T | A | 0.61 | 0.15 | 0.03 | 9.00E-07 | 21 | 22474790 |
| rs6725179 | A | G | 0.313 | 0.14 | 0.03 | 3.00E-06 | 2 | 126759652 |
| rs1247559 | C | T | 0.818 | 0.17 | 0.04 | 4.00E-06 | 6 | 160782724 |
| rs7086701 | T | G | 0.49 | 0.14 | 0.03 | 1.00E-06 | 10 | 10298081 |
| rs886431 | G | A | 0.177 | 0.17 | 0.04 | 4.00E-06 | 12 | 47733232 |
| rs2833579 | G | A | 0.485 | 0.14 | 0.03 | 3.00E-06 | 21 | 31956217 |
| rs729876 | T | C | 0.8 | 0.21 | 0.04 | 1.00E-08 | 16 | 16p13.12 |
| rs11084095 | A | G | 0.41 | 0.16 | 0.03 | 5.00E-08 | 19 | 51623777 |
| rs9982623 | C | T | 0.86 | 0.20 | 0.04 | 9.00E-07 | 21 | 21q22.3 |
| rs9984417 | T | A | 0.61 | 0.15 | 0.03 | 9.00E-07 | 21 | 22474790 |
| rs6725179 | A | G | 0.313 | 0.14 | 0.03 | 3.00E-06 | 2 | 126759652 |
| rs1247559 | C | T | 0.818 | 0.17 | 0.04 | 4.00E-06 | 6 | 160782724 |
| rs7086701 | T | G | 0.49 | 0.14 | 0.03 | 1.00E-06 | 10 | 10298081 |
| rs886431 | G | A | 0.177 | 0.17 | 0.04 | 4.00E-06 | 12 | 47733232 |
| rs2833579 | G | A | 0.485 | 0.14 | 0.03 | 3.00E-06 | 21 | 31956217 |
| **Schizophrenia** | | | | | | | | |
| rs10083370 | G | A | 0.4091 | 0.032619 | 0.00991 | 3.44E-14 | 12 | 110285440 |
| rs10148671 | T | C | 0.3384 | -0.02751 | 0.010208 | 5.46E-10 | 14 | 29000167 |
| rs10156310 | A | T | 0.7475 | 0.03161 | 0.01173 | 5.56E-10 | 8 | 38351611 |
| rs10196799 | A | T | 0.5606 | 0.024363 | 0.009563 | 4.51E-09 | 2 | 184776001 |
| rs1042992 | C | T | 0.8333 | -0.03184 | 0.012431 | 3.67E-09 | 8 | 26411675 |
| rs10520163 | T | C | 0.5152 | -0.02342 | 0.009711 | 2.81E-08 | 4 | 169705401 |
| rs10650434 | A | ACT | 0.3889 | -0.03709 | 0.0097 | 1.10E-18 | 7 | 1985462 |
| rs10783624 | C | A | 0.3384 | 0.02592 | 0.01023 | 5.44E-09 | 12 | 39129105 |
| rs1080500 | G | A | 0.7525 | 0.031408 | 0.01034 | 2.71E-12 | 3 | 53141001 |
| rs10985817 | T | C | 0.8485 | -0.03478 | 0.013116 | 1.02E-09 | 9 | 98308808 |
| rs111294930 | A | G | 0.7172 | 0.03575 | 0.012069 | 9.04E-12 | 5 | 152797561 |
| rs11165867 | C | T | 0.8636 | -0.03003 | 0.012578 | 3.87E-08 | 1 | 97412512 |
| rs1120004 | T | G | 0.2929 | 0.026778 | 0.010877 | 1.42E-08 | 12 | 23480498 |
| rs11409090 | A | AT | 0.5051 | -0.02601 | 0.01 | 2.11E-09 | 3 | 17846144 |
| rs11646127 | G | C | 0.5354 | 0.030721 | 0.009804 | 5.52E-13 | 10 | 18456176 |
| rs11685299 | C | A | 0.6667 | 0.026206 | 0.010248 | 3.86E-09 | 2 | 224526579 |
| rs11783093 | C | T | 0.8636 | 0.040444 | 0.013607 | 7.64E-12 | 8 | 27567832 |
| rs1191551 | T | G | 0.197 | 0.031651 | 0.011657 | 4.12E-10 | 14 | 29531199 |
| rs11993663 | C | A | 0.697 | -0.02399 | 0.010008 | 3.4E-08 | 8 | 10175384 |
| rs12009217 | A | G | 0.8121 | -0.02827 | 0.010198 | 1.78E-10 | X | 6002841 |
| rs12129573 | C | A | 0.6111 | -0.0332 | 0.00986 | 8.94E-15 | 12 | 110285440 |
| rs12129719 | G | A | 0.4646 | -0.02354 | 0.009819 | 3.35E-08 | 1 | 65858829 |
| rs12148337 | C | T | 0.5051 | -0.03575 | 0.014429 | 1.16E-08 | 15 | 70296933 |
| rs12293670 | A | G | 0.6313 | 0.035069 | 0.010137 | 1.70E-15 | 22 | 42207808 |
| rs12416331 | T | A | 0.9394 | 0.063183 | 0.016888 | 7.09E-18 | 10 | 103169157 |
| rs12447542 | G | A | 0.8838 | -0.03707 | 0.015058 | 1.44E-08 | 16 | 7694178 |
| rs12704290 | G | A | 0.8434 | 0.049489 | 0.015044 | 3.57E-14 | 14 | 103847845 |
| rs12705761 | G | C | 0.6212 | 0.028449 | 0.009971 | 5.11E-11 | 7 | 111336208 |
| rs12712510 | T | C | 0.4848 | 0.025019 | 0.010002 | 8.18E-09 | 2 | 22526854 |
| rs12898315 | G | A | 0.4747 | -0.02471 | 0.009546 | 2.51E-09 | 15 | 61561804 |
| rs12908161 | A | G | 0.7222 | 0.028937 | 0.010896 | 9.41E-10 | 15 | 84664594 |
| rs12991836 | A | C | 0.596 | -0.02651 | 0.009877 | 6.46E-10 | 2 | 144383974 |
| rs13107325 | C | T | 0.904 | -0.06932 | 0.019267 | 1.19E-16 | 12 | 123180566 |
| rs13121251 | T | C | 0.6717 | 0.024937 | 0.01047 | 4.06E-08 | 4 | 142908606 |
| rs13169274 | T | C | 0.5152 | -0.0259 | 0.009675 | 7.06E-10 | 5 | 138519616 |
| rs1319017 | G | A | 0.6869 | -0.02884 | 0.01021 | 7.82E-11 | 9 | 82121388 |
| rs1339227 | C | T | 0.6465 | 0.027227 | 0.010008 | 3.76E-10 | 6 | 72445999 |
| rs1353545 | G | C | 0.6566 | -0.02565 | 0.010137 | 5.67E-09 | 3 | 60302116 |
| rs140505938 | C | T | 0.803 | 0.036349 | 0.013541 | 6.50E-10 | 1 | 150059494 |
| rs14403 | C | T | 0.7525 | 0.032216 | 0.011617 | 1.71E-10 | 1 | 243500591 |
| rs1451488 | A | G | 0.4444 | -0.02916 | 0.009712 | 4.75E-12 | 2 | 199125384 |
| rs146678232 | CA | C | 0.8434 | -0.03079 | 0.0126 | 1.63E-08 | 7 | 24737471 |
| rs1473594 | T | C | 0.3939 | 0.027798 | 0.009656 | 3.33E-11 | 8 | 59783967 |
| rs150437760 | A | C | 0.9495 | 0.052694 | 0.022196 | 4.58E-08 | 14 | 59515050? |
| rs16867576 | A | G | 0.8939 | 0.043873 | 0.014996 | 1.65E-11 | 5 | 89450514 |
| rs16902086 | A | G | 0.6465 | -0.02865 | 0.010063 | 5.55E-11 | 5 | 45285650 |
| rs17465671 | C | A | 0.4444 | 0.024978 | 0.009784 | 4.14E-09 | 16 | 63678815 |
| rs17514846 | A | C | 0.4949 | -0.02989 | 0.009831 | 2.55E-12 | 15 | 90873320 |
| rs1765142 | C | A | 0.399 | -0.025 | 0.010078 | 1.13E-08 | 11 | 30357012 |
| rs1899543 | A | T | 0.4899 | -0.02504 | 0.00949 | 1.23E-09 | 11 | 24384873 |
| rs1975802 | A | G | 0.8687 | -0.03016 | 0.012598 | 3.56E-08 | 16 | 68251944 |
| rs198160 | G | T | 0.5051 | 0.024321 | 0.010258 | 4.88E-08 | 16 | 24229404 |
| rs2007044 | A | G | 0.5707 | -0.03852 | 0.009691 | 5.63E-20 | 12 | 2235794 |
| rs2053079 | A | G | 0.697 | -0.03071 | 0.011092 | 1.82E-10 | 19 | 30496516 |
| rs2077586 | A | G | 0.702 | 0.026697 | 0.011083 | 2.96E-08 | 2 | 72934422 |
| rs211829 | T | C | 0.601 | 0.025634 | 0.009877 | 2.29E-09 | 7 | 110408836 |
| rs215411 | T | A | 0.6919 | -0.02511 | 0.010191 | 1.4E-08 | 4 | 23421980 |
| rs2161711 | A | G | 0.8283 | 0.030357 | 0.012752 | 4.22E-08 | 16 | 71325163? |
| rs217287 | C | T | 0.5808 | 0.029992 | 0.009675 | 9.53E-13 | 22 | 41191552 |
| rs2332700 | C | G | 0.2778 | 0.030397 | 0.010931 | 1.52E-10 | 14 | 71950609 |
| rs2410572 | G | A | 0.5303 | 0.023705 | 0.009546 | 1.07E-08 | 8 | 18563964 |
| rs2514218 | C | T | 0.6313 | -0.03776 | 0.012407 | 2.42E-12 | 11 | 113522272 |
| rs254782 | A | G | 0.0303 | -0.05462 | 0.022975 | 4.4E-08 | 5 | 88704813 |
| rs2660304 | G | T | 0.1768 | -0.04702 | 0.012376 | 2.18E-18 | 1 | 98046571 |
| rs281299 | C | T | 0.4192 | -0.02396 | 0.009858 | 2.19E-08 | 15 | 47393884 |
| rs28374258 | T | A | 0.8283 | -0.03187 | 0.011873 | 6.35E-10 | 1 | 190980421 |
| rs2851447 | G | C | 0.2778 | 0.037865 | 0.010765 | 5.55E-16 | 1 | 73302683 |
| rs2905432 | G | A | 0.3687 | 0.029789 | 0.009989 | 6.62E-12 | 19 | 19373486 |
| rs2917569 | T | C | 0.4495 | 0.026492 | 0.009691 | 3.11E-10 | 11 | 132698360 |
| rs2949006 | T | G | 0.2121 | 0.04348 | 0.011892 | 3.69E-17 | 4 | 102267552 |
| rs2970610 | T | C | 0.3434 | 0.029465 | 0.010045 | 1.39E-11 | 1 | 43631859 |
| rs312477 | G | A | 0.7828 | 0.028327 | 0.011499 | 1.38E-08 | 3 | 53481109 |
| rs3130820 | T | A | 0.904 | 0.107413 | 0.017695 | 2.12E-44 | 6 | 29238906 |
| rs34179565 | CA | C | 0.5253 | -0.02423 | 0.0097 | 8.88E-09 | 14 | 32829526 |
| rs34269918 | G | GA | 0.702 | -0.02627 | 0.0102 | 3.27E-09 | 1 | 8364925 |
| rs34796896 | G | A | 0.803 | 0.03615 | 0.01195 | 3.19E-12 | 3 | 180905467 |
| rs35346733 | G | A | 0.8283 | 0.031489 | 0.010352 | 2.42E-12 | 3 | 2479638 |
| rs35604463 | G | A | 0.6212 | 0.024363 | 0.009945 | 1.66E-08 | 14 | 99245695 |
| rs35736453 | T | TC | 0.2727 | -0.02632 | 0.0109 | 2.95E-08 | 6 | 128008349 |
| rs35774874 | T | C | 0.5404 | 0.029952 | 0.010276 | 1.97E-11 | 11 | 130941461 |
| rs36043959 | G | A | 0.8232 | 0.029465 | 0.009784 | 4.07E-12 | 8 | 110459785 |
| rs36104021 | C | G | 0.8384 | 0.039216 | 0.015612 | 7.31E-09 | 12 | 102967334 |
| rs3735025 | T | C | 0.6515 | 0.028042 | 0.009897 | 7.02E-11 | 7 | 137390098 |
| rs3743078 | C | G | 0.2121 | -0.03395 | 0.01121 | 3.11E-12 | 15 | 78602417 |
| rs4144797 | T | C | 0.3788 | 0.03551 | 0.010059 | 4.33E-16 | 11 | 124743036 |
| rs4240748 | C | G | 0.3687 | -0.0242 | 0.009951 | 2.15E-08 | 12 | 91853010 |
| rs42945 | A | G | 0.4242 | -0.02742 | 0.009953 | 2.25E-10 | 16 | 58511522 |
| rs4470825 | G | A | 0.5707 | 0.024157 | 0.009675 | 8.94E-09 | 6 | 83080079? |
| rs4648845 | C | T | 0.5303 | -0.03317 | 0.01113 | 6.74E-12 | 1 | 2455662 |
| rs4650963 | T | C | 0.1515 | 0.023541 | 0.009509 | 1.16E-08 | 1 | 177340354 |
| rs4766428 | C | T | 0.5404 | -0.03308 | 0.010005 | 2.68E-14 | 7 | 86798310 |
| rs489939 | G | A | 0.6263 | 0.024773 | 0.010025 | 1.24E-08 | 3 | 161752804 |
| rs4925114 | A | G | 0.303 | 0.024568 | 0.010172 | 2.64E-08 | 17 | 17807956 |
| rs4936215 | A | G | 0.803 | 0.039652 | 0.012135 | 5.32E-14 | 18 | 55081986 |
| rs55669358 | T | C | 0.904 | -0.04122 | 0.016715 | 1.37E-08 | 8 | 34454894 |
| rs56145559 | C | T | 0.7576 | -0.03112 | 0.01173 | 1.01E-09 | 2 | 73396311 |
| rs56282503 | T | C | 0.6465 | -0.02707 | 0.011152 | 2.3E-08 | 15 | 40274558 |
| rs56775891 | C | T | 0.7424 | -0.02893 | 0.011111 | 2.03E-09 | 18 | 79815613 |
| rs56807175 | T | TC | 0.8333 | -0.03804 | 0.0129 | 1.36E-11 | 2 | 145678655 |
| rs5757730 | A | G | 0.4798 | -0.03093 | 0.010101 | 1.76E-12 | 22 | 39571425 |
| rs58033671 | A | ACCCCTGCACGCCCG | 0.399 | 0.036349 | 0.0097 | 8.65E-18 | 8 | 142238758 |
| rs5825114 | G | GA | 0.399 | -0.03166 | 0.0097 | 5.03E-14 | 5 | 61325153 |
| rs58950470 | G | T | 0.6465 | -0.02495 | 0.010245 | 2.07E-08 | 11 | 65616284 |
| rs6002655 | C | T | 0.5657 | -0.0326 | 0.009823 | 2.15E-14 | 14 | 103847845 |
| rs6035706 | A | G | 0.7323 | -0.02593 | 0.010319 | 7.24E-09 | 20 | 20840362 |
| rs6065094 | A | G | 0.3535 | -0.03695 | 0.010211 | 7.91E-17 | 2 | 232697487 |
| rs61937595 | C | T | 0.8939 | 0.054268 | 0.018836 | 3.28E-11 | 12 | 57289173 |
| rs62334820 | C | T | 0.8081 | -0.03523 | 0.011906 | 9.60E-12 | 4 | 175934070 |
| rs62606711 | A | G | 0.2416 | 0.031311 | 0.010198 | 1.26E-12 | X | 69157361 |
| rs634940 | G | T | 0.7424 | -0.0275 | 0.011134 | 1.3E-08 | 6 | 92367782 |
| rs6434928 | G | A | 0.3131 | 0.031691 | 0.010044 | 3.62E-13 | 11 | 46351761 |
| rs6678676 | T | C | 0.2525 | -0.02839 | 0.011821 | 3.22E-08 | 1 | 200297375 |
| rs6680011 | A | C | 0.8535 | -0.03193 | 0.013245 | 2.83E-08 | 1 | 95375310 |
| rs2098497 | G | C | 0.7081 | 0.037944 | 0.015428 | 1.47E-08 | 17 | 19111518 |
| rs6694545 | A | G | 0.2576 | 0.033343 | 0.011172 | 6.20E-12 | 1 | 29964421 |
| rs6701877 | G | T | 0.8384 | 0.031812 | 0.013116 | 2.37E-08 | 1 | 174046121 |
| rs6800435 | C | A | 0.8889 | -0.03575 | 0.014669 | 2E-08 | 3 | 10762866 |
| rs7010876 | T | A | 0.2273 | 0.026982 | 0.01071 | 6.51E-09 | 8 | 88252522 |
| rs704373 | A | G | 0.3737 | 0.028042 | 0.010063 | 1.39E-10 | 3 | 63881679 |
| rs7129727 | G | A | 0.6717 | -0.02716 | 0.010341 | 1.47E-09 | 11 | 57717188 |
| rs71534391 | G | GA | 0.71 | -0.02892 | 0.0116 | 8.48E-09 | 8 | 4323090 |
| rs7191183 | T | C | 0.6566 | -0.02585 | 0.010248 | 6.31E-09 | 16 | 9806200 |
| rs7216638 | T | A | 0.6616 | 0.028042 | 0.010358 | 4.59E-10 | 17 | 2253159 |
| rs7225476 | G | A | 0.4293 | -0.02258 | 0.009527 | 4.86E-08 | 17 | 80587803? |
| rs72769124 | C | A | 0.8687 | -0.04725 | 0.017468 | 4.73E-10 | 1 | 239046758 |
| rs72986630 | C | T | 0.9545 | -0.06083 | 0.0228 | 8.09E-10 | 19 | 11738921 |
| rs7432375 | G | A | 0.5404 | -0.03489 | 0.011584 | 4.07E-12 | 3 | 136569563 |
| rs7499750 | A | C | 0.2424 | 0.03064 | 0.011303 | 4.24E-10 | 16 | 13655408 |
| rs7508148 | T | C | 0.7879 | 0.033504 | 0.011683 | 4.06E-11 | 19 | 49648885 |
| rs7596038 | C | T | 0.5303 | 0.029181 | 0.009582 | 2.37E-12 | 2 | 58156685 |
| rs75968099 | C | T | 0.5707 | -0.02855 | 0.010152 | 9.41E-11 | 3 | 36817092 |
| rs760608 | G | A | 0.2626 | 0.02637 | 0.010803 | 1.9E-08 | 6 | 114398283 |
| rs7632921 | G | T | 0.5859 | 0.024568 | 0.009851 | 9.52E-09 | 3 | 71494607 |
| rs7701440 | T | C | 0.5 | -0.03144 | 0.009563 | 3.72E-14 | 7 | 86798310 |
| rs7789569 | T | C | 0.3232 | 0.028327 | 0.010005 | 7.00E-11 | 7 | 105287139 |
| rs7801375 | A | G | 0.1364 | -0.03359 | 0.013314 | 6.27E-09 | 7 | 131882504 |
| rs783540 | A | G | 0.5909 | -0.02564 | 0.00962 | 8.45E-10 | 15 | 82585958 |
| rs7893279 | T | G | 0.9192 | 0.048325 | 0.015387 | 4.80E-13 | 2 | 197439853 |
| rs7951870 | T | C | 0.8434 | -0.04053 | 0.012793 | 2.99E-13 | 11 | 133982789 |
| rs893949 | C | T | 0.4899 | 0.02317 | 0.009632 | 2.98E-08 | 11 | 134426490 |
| rs9545047 | A | C | 0.596 | 0.024321 | 0.009804 | 1.15E-08 | 13 | 79285321 |
| rs9607782 | T | A | 0.7071 | -0.03523 | 0.01125 | 5.54E-13 | 16 | 29954956 |
| rs9881798 | A | C | 0.5909 | 0.023005 | 0.009544 | 2.81E-08 | 3 | 16805459 |
| **SHBG** | | | | | | | | |
| rs4149056 | T | C | 0.82 | 0.029 | 0.005 | 1.90E-08 | 12 | 21178615 |
| rs440837 | A | G | 0.78 | -0.028 | 0.005 | 3.00E-09 | 8 | 80549739 |
| rs8023580 | T | C | 0.72 | -0.025 | 0.006 | 5.00E-06 | 15 | 96165062 |
| rs10454142 | T | C | 0.69 | 0.023 | 0.004 | 1.30E-07 | 2 | 48419260 |
| rs293428 | A | G | 0.69 | -0.029 | 0.005 | 3.00E-08 | 4 | 68726064 |
| rs17496332 | A | G | 0.67 | -0.027 | 0.005 | 2.00E-07 | 1 | 107003753 |
| rs7910927 | T | G | 0.51 | -0.05 | 0.005 | 1.00E-25 | 10 | 63379150 |
| rs780093 | T | C | 0.4 | -0.026 | 0.005 | 7.00E-08 | 2 | 27519736 |
| rs1573036 | T | C | 0.39 | 0.028 | 0.004 | 3.00E-16 | X | 110576840 |
| rs2411984 | A | G | 0.28 | 0.034 | 0.006 | 2.00E-10 | 17 | 49368389 |
| rs12150660 | T | G | 0.24 | 0.11 | 0.006 | 4.00E-80 | 17 | 7618597 |
| rs3779195 | A | T | 0.17 | -0.028 | 0.005 | 3.00E-08 | 7 | 98364050 |
| **SLE** | | | | | | | | |
| rs10028805 | G | A | 0.6566 | 0.182 | 0.022 | 4.00E-17 | 4 | 101816093 |
| rs10036748 | T | C | 0.2121 | 0.322 | 0.024 | 1.00E-45 | 5 | 151078585 |
| rs10048743 | G | T | 0.0909 | 0.223 | 0.035 | 2.00E-10 | 2 | 213025508 |
| rs10488631 | C | T | 0.1111 | 0.652 | 0.029 | 9.00E-110 | 7 | 128954129 |
| rs1059312 | G | A | 0.399 | 0.157 | 0.021 | 1.00E-13 | 12 | 128794319 |
| rs10753074 | T | C | 0.6212 | 0.191 | 0.028 | 6.00E-12 | 1 | 173377204 |
| rs10774625 | A | G | 0.4697 | 0.122 | 0.021 | 4.00E-09 | 12 | 111472415 |
| rs10912578 | A | G | 0.3081 | 0.239 | 0.027 | 4.00E-19 | 1 | 173282717 |
| rs11059919 | G | A | 0.3889 | 0.148 | 0.026 | 8.00E-09 | 12 | 128804645 |
| rs11085727 | C | T | 0.697 | 0.215 | 0.029 | 1.00E-13 | 19 | 10355447 |
| rs114090659 | C | T | 0.1566 | 0.688 | 0.034 | 6.00E-92 | 6 | 30973212 |
| rs1150757 | A | G | 0.0859 | 0.846 | 0.039 | 6.00E-107 | 6 | 32061428 |
| rs11644034 | G | A | 0.798 | 0.223 | 0.026 | 1.00E-17 | 16 | 85939006 |
| rs11889341 | T | C | 0.2172 | 0.548 | 0.024 | 6.00E-122 | 2 | 191079016 |
| rs12531540 | C | T | 0.5051 | 0.14 | 0.025 | 3.00E-08 | 7 | 28123055 |
| rs1270942 | G | A | 0.0808 | 0.824 | 0.03 | 2.00E-165 | 6 | 31951083 |
| rs12802200 | C | A | 0.8333 | 0.207 | 0.034 | 9.00E-10 | 11 | 566936 |
| rs13332649 | A | G | 0.803 | 0.293 | 0.033 | 2.00E-18 | 16 | 85933077 |
| rs143123127 | A | G | 0.0253 | 0.412 | 0.071 | 6.00E-09 | 17 | 39850937 |
| rs1734787 | C | A | 0.1745 | 0.27 | 0.034 | 2.00E-15 | X | 154059995 |
| rs17849501 | T | G | 0.7172 | 0.742 | 0.038 | 3.00E-88 | 1 | 183573188 |
| rs2111485 | G | A | 0.6111 | 0.14 | 0.021 | 1.00E-11 | 2 | 162254026 |
| rs2286672 | T | C | 0.0758 | 0.223 | 0.038 | 3.00E-09 | 17 | 4809322 |
| rs2289583 | A | C | 0.2778 | 0.174 | 0.022 | 6.00E-15 | 15 | 75018695 |
| rs2304256 | C | A | 0.7121 | 0.215 | 0.03 | 4.00E-13 | 19 | 10364976 |
| rs2431098 | G | A | 0.4493 | 0.223 | 0.026 | 4.00E-18 | 5 | 160460329 |
| rs2431697 | T | C | 0.5404 | 0.231 | 0.021 | 8.00E-28 | 5 | 160452971 |
| rs2476601 | A | G | 0.1162 | 0.358 | 0.032 | 1.00E-28 | 1 | 113834946 |
| rs268134 | G | A | 0.7525 | 0.191 | 0.029 | 1.00E-10 | 2 | 65381229 |
| rs2732549 | A | G | 0.6061 | 0.215 | 0.021 | 1.00E-23 | 11 | 35066852 |
| rs2736332 | C | G | 0.2576 | 0.27 | 0.028 | 2.00E-22 | 8 | 11482456 |
| rs2736340 | T | C | 0.2374 | 0.255 | 0.028 | 6.00E-20 | 8 | 11486464 |
| rs2941509 | T | C | 0.0303 | 0.3 | 0.052 | 8.00E-09 | 17 | 39764941 |
| rs3024505 | A | G | 0.1919 | 0.157 | 0.027 | 5.00E-09 | 1 | 206766559 |
| rs34572943 | A | G | 0.101 | 0.536 | 0.03 | 3.00E-76 | 16 | 31261032 |
| rs35000415 | T | C | 0.1111 | 0.604 | 0.037 | 1.00E-60 | 7 | 128945562 |
| rs35472514 | G | C | 0.1162 | 0.531 | 0.035 | 4.00E-53 | 16 | 31272002 |
| rs3747093 | A | G | 0.1869 | 0.231 | 0.032 | 3.00E-13 | 22 | 21630090 |
| rs3757387 | C | T | 0.4192 | 0.372 | 0.025 | 1.00E-48 | 7 | 128936032 |
| rs3768792 | G | A | 0.0909 | 0.215 | 0.029 | 1.00E-13 | 2 | 213006985 |
| rs3794060 | C | T | 0.298 | 0.207 | 0.022 | 1.00E-20 | 11 | 71476633 |
| rs3957147 | T | C | 0.1263 | 0.693 | 0.034 | 3.00E-93 | 6 | 32714358 |
| rs4388254 | T | C | 0.0303 | 0.336 | 0.055 | 7.00E-10 | 5 | 134092910 |
| rs4902562 | A | G | 0.4141 | 0.131 | 0.021 | 6.00E-10 | 14 | 68264741 |
| rs4917014 | T | G | 0.6768 | 0.166 | 0.022 | 6.00E-14 | 7 | 50266267 |
| rs4948496 | C | T | 0.4697 | 0.131 | 0.02 | 1.00E-10 | 10 | 62045858 |
| rs564799 | C | T | 0.6111 | 0.131 | 0.022 | 2.00E-09 | 3 | 160011200 |
| rs58688157 | A | G | 0.7323 | 0.215 | 0.03 | 5.00E-13 | 11 | 625085 |
| rs58721818 | T | C | 0.0303 | 0.599 | 0.067 | 3.00E-19 | 6 | 137922602 |
| rs597808 | A | G | 0.4545 | 0.166 | 0.027 | 6.00E-10 | 12 | 111535554 |
| rs6568431 | A | C | 0.4596 | 0.191 | 0.025 | 5.00E-14 | 6 | 106140931 |
| rs6671847 | A | G | 0.4747 | 0.182 | 0.026 | 1.00E-12 | 1 | 161509020 |
| rs6736175 | C | T | 0.4192 | 0.215 | 0.026 | 9.00E-17 | 2 | 191081596 |
| rs6932056 | C | T | 0.0202 | 0.604 | 0.052 | 2.00E-31 | 6 | 137921300 |
| rs704840 | G | T | 0.2828 | 0.199 | 0.022 | 3.00E-19 | 1 | 173257056 |
| rs7097397 | G | A | 0.6566 | 0.182 | 0.027 | 9.00E-12 | 10 | 48817351 |
| rs74290525 | G | A | 0.9798 | 0.723 | 0.101 | 1.00E-12 | 6 | 31867385 |
| rs7444 | C | T | 0.1768 | 0.239 | 0.025 | 2.00E-22 | 22 | 21622645 |
| rs7726414 | T | C | 0.0303 | 0.372 | 0.046 | 4.00E-16 | 5 | 134096143 |
| rs77583790 | A | G | 0.0051 | 0.765 | 0.117 | 6.00E-11 | 3 | 159976265 |
| rs7941765 | C | T | 0.5152 | 0.131 | 0.02 | 1.00E-10 | 11 | 128629105 |
| rs820077 | G | A | 0.1768 | 0.174 | 0.03 | 1.00E-08 | 6 | 35066077 |
| rs849142 | T | C | 0.5051 | 0.131 | 0.02 | 9.00E-11 | 7 | 28146272 |
| rs887369 | C | A | 0.7584 | 0.14 | 0.022 | 5.00E-10 | X | 30559729 |
| rs9273076 | T | C | 0.197 | 0.262 | 0.037 | 8.00E-13 | 6 | 32644524 |
| rs9311676 | C | T | 0.596 | 0.157 | 0.021 | 3.00E-14 | 3 | 58484624 |
| rs9462027 | A | G | 0.2576 | 0.131 | 0.023 | 8.00E-09 | 6 | 34829464 |
| rs9652601 | G | A | 0.7071 | 0.191 | 0.023 | 7.00E-17 | 16 | 11080508 |
| rs9782955 | C | T | 0.7475 | 0.148 | 0.024 | 1.00E-09 | 1 | 235876577 |
| **Smoking status (ever vs never smokers)** | | | | | | | | |
| rs7938812 | T | G | 0.61 | -0.0299 | 0.0021 | 7.00E-48 | 11 | 113040282 |
| rs961414 | T | G | 0.533 | -0.022 | 0.002 | 7.00E-28 | 2 | 145356165 |
| rs240955 | A | G | 0.813 | -0.0255 | 0.0026 | 4.00E-23 | 6 | 111339440 |
| rs7092200 | T | C | 0.585 | -0.0197 | 0.002 | 4.00E-22 | 10 | 103085115 |
| rs11783093 | T | C | 0.166 | -0.0259 | 0.0027 | 7.00E-22 | 8 | 27567832 |
| rs1368550 | T | C | 0.519 | 0.0192 | 0.002 | 1.00E-21 | 2 | 103462197 |
| rs34495106 | A | G | 0.372 | 0.0208 | 0.0022 | 2.00E-20 | 3 | 85601986 |
| rs3911063 | T | C | 0.674 | 0.0191 | 0.0021 | 5.00E-19 | 3 | 85857778 |
| rs7921378 | C | G | 0.481 | -0.0175 | 0.002 | 3.00E-18 | 10 | 61915126 |
| rs6265 | T | C | 0.192 | -0.0222 | 0.0026 | 4.00E-18 | 11 | 27658369 |
| rs1909590 | A | T | 0.148 | -0.0249 | 0.0031 | 4.00E-16 | 3 | 118096996 |
| rs1004787 | A | G | 0.53 | 0.0177 | 0.0022 | 4.00E-16 | 2 | 44931952 |
| rs4664442 | A | G | 0.463 | 0.016 | 0.002 | 2.00E-15 | 2 | 161971491 |
| rs1435741 | A | G | 0.432 | 0.0158 | 0.002 | 6.00E-15 | 15 | 47643646 |
| rs13237637 | C | G | 0.497 | -0.0163 | 0.0022 | 6.00E-14 | 7 | 3463575 |
| rs4869058 | A | T | 0.641 | -0.0156 | 0.0021 | 8.00E-14 | 5 | 167565242 |
| rs17417989 | T | G | 0.3 | 0.0176 | 0.0024 | 1.00E-13 | 11 | 112843134 |
| rs7668995 | A | T | 0.292 | -0.0163 | 0.0022 | 1.00E-13 | 4 | 146998126 |
| rs2310752 | A | G | 0.43 | -0.0149 | 0.002 | 2.00E-13 | 1 | 65926722 |
| rs13258512 | A | G | 0.576 | 0.0149 | 0.002 | 3.00E-13 | 8 | 91765205 |
| rs11210892 | A | G | 0.673 | -0.0156 | 0.0021 | 4.00E-13 | 1 | 43634413 |
| rs13396935 | A | G | 0.177 | -0.0191 | 0.0026 | 4.00E-13 | 2 | 653195 |
| rs2585817 | A | G | 0.618 | 0.0149 | 0.0021 | 5.00E-13 | 11 | 28580674 |
| rs4984916 | A | G | 0.221 | -0.0174 | 0.0024 | 7.00E-13 | 16 | 699057 |
| rs10914684 | A | G | 0.328 | -0.0152 | 0.0021 | 1.00E-12 | 1 | 33329971 |
| rs3099769 | A | G | 0.409 | 0.0144 | 0.002 | 2.00E-12 | 11 | 132318528 |
| rs2419903 | T | C | 0.416 | -0.0143 | 0.002 | 2.00E-12 | 2 | 60300917 |
| rs6119897 | A | G | 0.243 | 0.0164 | 0.0023 | 3.00E-12 | 20 | 32557613 |
| rs74697736 | A | G | 0.288 | 0.0167 | 0.0024 | 3.00E-12 | 2 | 144654704 |
| rs7585579 | C | G | 0.506 | -0.014 | 0.002 | 3.00E-12 | 2 | 59797722 |
| rs1919621 | A | G | 0.528 | 0.014 | 0.002 | 4.00E-12 | 3 | 34380253 |
| rs12030183 | T | C | 0.679 | 0.0149 | 0.0022 | 4.00E-12 | 1 | 41295548 |
| rs6965740 | T | G | 0.454 | -0.0139 | 0.002 | 5.00E-12 | 7 | 117874786 |
| rs4650277 | A | G | 0.434 | 0.0151 | 0.0022 | 6.00E-12 | 1 | 74528037 |
| rs1469908 | T | C | 0.588 | -0.0141 | 0.002 | 6.00E-12 | 16 | 69730509 |
| rs6852117 | C | G | 0.58 | 0.014 | 0.002 | 6.00E-12 | 4 | 172155737 |
| rs12764388 | A | G | 0.113 | 0.0218 | 0.0032 | 6.00E-12 | 10 | 102653628 |
| rs2697329 | A | G | 0.71 | -0.0151 | 0.0022 | 9.00E-12 | 2 | 145134860 |
| rs10994943 | T | G | 0.581 | 0.015 | 0.0022 | 1.00E-11 | 10 | 61831655 |
| rs9423279 | C | G | 0.343 | 0.0155 | 0.0023 | 1.00E-11 | 10 | 123920903 |
| rs7754741 | T | C | 0.728 | -0.0153 | 0.0023 | 1.00E-11 | 6 | 98301453 |
| rs2340403 | T | C | 0.626 | -0.014 | 0.0021 | 2.00E-11 | 1 | 73370094 |
| rs597808 | A | G | 0.484 | 0.0146 | 0.0022 | 2.00E-11 | 12 | 111535554 |
| rs17151637 | T | C | 0.284 | -0.0149 | 0.0022 | 2.00E-11 | 8 | 10295572 |
| rs42417 | T | C | 0.69 | 0.0157 | 0.0023 | 3.00E-11 | 5 | 94862585 |
| rs1221976 | A | C | 0.663 | -0.0142 | 0.0021 | 3.00E-11 | 18 | 52469766 |
| rs77878475 | A | T | 0.086 | -0.0258 | 0.0039 | 3.00E-11 | 16 | 17964691 |
| rs35761479 | A | G | 0.121 | -0.0221 | 0.0033 | 3.00E-11 | 1 | 154181718 |
| rs1246265 | T | C | 0.305 | -0.0156 | 0.0024 | 3.00E-11 | 9 | 84146830 |
| rs4737525 | A | G | 0.511 | -0.0133 | 0.002 | 4.00E-11 | 8 | 58887222 |
| rs12042107 | T | C | 0.451 | 0.0143 | 0.0022 | 5.00E-11 | 1 | 90730619 |
| rs2202237 | T | C | 0.531 | -0.0132 | 0.002 | 6.00E-11 | 17 | 52131424 |
| rs76608582 | A | C | 0.048 | -0.0334 | 0.0051 | 6.00E-11 | 19 | 4474728 |
| rs1492546 | C | G | 0.449 | -0.0142 | 0.0022 | 9.00E-11 | 2 | 80786611 |
| rs12022778 | A | C | 0.788 | -0.0159 | 0.0025 | 1.00E-10 | 1 | 50138323 |
| rs26251 | T | G | 0.629 | 0.0134 | 0.0021 | 1.00E-10 | 5 | 107501749 |
| rs5003492 | A | G | 0.425 | 0.0131 | 0.002 | 1.00E-10 | 8 | 92183301 |
| rs17669415 | C | G | 0.684 | 0.0139 | 0.0022 | 1.00E-10 | 18 | 52358627 |
| rs17733784 | T | C | 0.62 | 0.0132 | 0.0021 | 2.00E-10 | 18 | 56052694 |
| rs11255908 | T | G | 0.744 | -0.0146 | 0.0023 | 2.00E-10 | 10 | 8760949 |
| rs1430065 | T | C | 0.27 | 0.0143 | 0.0023 | 2.00E-10 | 2 | 103240616 |
| rs4543592 | T | C | 0.521 | -0.0127 | 0.002 | 3.00E-10 | 9 | 3014254 |
| rs7870475 | T | C | 0.525 | -0.0127 | 0.002 | 3.00E-10 | 9 | 125371755 |
| rs3818987 | T | C | 0.473 | -0.0137 | 0.0022 | 3.00E-10 | 6 | 37516830 |
| rs4790870 | A | C | 0.61 | 0.013 | 0.0021 | 3.00E-10 | 17 | 2066907 |
| rs1373129 | A | C | 0.472 | -0.0127 | 0.002 | 3.00E-10 | 9 | 99391416 |
| rs7679162 | T | G | 0.66 | 0.0144 | 0.0023 | 3.00E-10 | 4 | 31181708 |
| rs11134672 | A | G | 0.687 | 0.0136 | 0.0022 | 3.00E-10 | 5 | 170968816 |
| rs2240294 | A | T | 0.448 | -0.0127 | 0.002 | 3.00E-10 | 7 | 96994945 |
| rs883403 | T | C | 0.843 | 0.0173 | 0.0028 | 3.00E-10 | 7 | 99450355 |
| rs72678864 | A | G | 0.173 | -0.018 | 0.0029 | 3.00E-10 | 4 | 111500989 |
| rs568599 | C | G | 0.54 | 0.0127 | 0.002 | 3.00E-10 | 11 | 121665302 |
| rs1267068 | T | G | 0.697 | 0.0137 | 0.0022 | 4.00E-10 | 2 | 161155624 |
| rs4949465 | T | C | 0.865 | -0.0183 | 0.0029 | 4.00E-10 | 1 | 31712888 |
| rs28647734 | A | G | 0.211 | 0.0166 | 0.0027 | 4.00E-10 | 9 | 135085187 |
| rs1108879 | A | G | 0.555 | -0.0126 | 0.002 | 5.00E-10 | 7 | 3516500 |
| rs17049905 | A | G | 0.895 | -0.0204 | 0.0033 | 5.00E-10 | 2 | 59074437 |
| rs12600773 | T | C | 0.354 | 0.013 | 0.0021 | 6.00E-10 | 17 | 52258612 |
| rs4814884 | T | C | 0.539 | 0.0125 | 0.002 | 6.00E-10 | 20 | 19682649 |
| rs10952199 | T | C | 0.428 | -0.0136 | 0.0022 | 6.00E-10 | 7 | 1625703 |
| rs1931386 | C | G | 0.495 | 0.0124 | 0.002 | 6.00E-10 | 9 | 11177422 |
| rs13187930 | T | C | 0.26 | 0.0142 | 0.0023 | 6.00E-10 | 5 | 61088344 |
| rs4856463 | T | C | 0.218 | -0.0162 | 0.0026 | 7.00E-10 | 3 | 83589417 |
| rs479971 | A | G | 0.274 | 0.015 | 0.0024 | 7.00E-10 | 12 | 69278691 |
| rs9864951 | A | C | 0.647 | 0.014 | 0.0023 | 7.00E-10 | 3 | 118221997 |
| rs6714110 | A | G | 0.033 | 0.0344 | 0.0056 | 8.00E-10 | 2 | 145060608 |
| rs1891196 | A | G | 0.565 | -0.0124 | 0.002 | 9.00E-10 | 1 | 208545327 |
| rs1109480 | A | G | 0.389 | -0.0136 | 0.0022 | 9.00E-10 | 12 | 120645476 |
| rs1136998 | A | T | 0.105 | -0.02 | 0.0033 | 1.00E-09 | 2 | 43888044 |
| rs4748779 | T | C | 0.724 | -0.0137 | 0.0022 | 1.00E-09 | 10 | 21999203 |
| rs13065007 | A | T | 0.265 | 0.0139 | 0.0023 | 1.00E-09 | 3 | 117803865 |
| rs12211126 | T | C | 0.593 | -0.0124 | 0.002 | 1.00E-09 | 6 | 66839558 |
| rs1628768 | T | C | 0.765 | 0.0155 | 0.0026 | 1.00E-09 | 10 | 103253237 |
| rs12690535 | A | G | 0.811 | 0.0155 | 0.0026 | 1.00E-09 | 2 | 225394657 |
| rs13357015 | A | G | 0.636 | 0.0136 | 0.0023 | 2.00E-09 | 5 | 80967584 |
| rs3783177 | T | G | 0.758 | 0.0141 | 0.0023 | 2.00E-09 | 13 | 100499106 |
| rs11872397 | A | G | 0.26 | -0.0149 | 0.0025 | 2.00E-09 | 18 | 74823326 |
| rs2324999 | T | C | 0.2 | 0.0151 | 0.0025 | 2.00E-09 | 3 | 86109735 |
| rs12787182 | A | G | 0.709 | 0.0133 | 0.0022 | 2.00E-09 | 11 | 59423338 |
| rs10970595 | T | C | 0.117 | -0.0188 | 0.0031 | 2.00E-09 | 9 | 31910766 |
| rs17373738 | C | G | 0.618 | 0.0124 | 0.0021 | 2.00E-09 | 4 | 140014110 |
| rs12535004 | T | C | 0.752 | -0.015 | 0.0025 | 2.00E-09 | 7 | 115307058 |
| rs10780649 | T | G | 0.479 | -0.013 | 0.0022 | 2.00E-09 | 9 | 84088646 |
| rs1825734 | T | C | 0.839 | 0.0163 | 0.0027 | 2.00E-09 | 17 | 52255197 |
| rs1863161 | A | G | 0.559 | 0.0121 | 0.002 | 2.00E-09 | 2 | 59912389 |
| rs1775370 | T | C | 0.248 | -0.0139 | 0.0023 | 2.00E-09 | 1 | 72527173 |
| rs10928235 | A | T | 0.748 | 0.0138 | 0.0023 | 3.00E-09 | 2 | 144920547 |
| rs2866724 | A | G | 0.732 | -0.0135 | 0.0023 | 3.00E-09 | 16 | 13666295 |
| rs2291256 | T | C | 0.091 | 0.0208 | 0.0035 | 3.00E-09 | 12 | 132816737 |
| rs2734971 | A | G | 0.465 | 0.013 | 0.0022 | 3.00E-09 | 6 | 29866672 |
| rs61902492 | A | G | 0.776 | -0.0155 | 0.0026 | 3.00E-09 | 11 | 113121199 |
| rs1486900 | T | G | 0.175 | 0.0158 | 0.0026 | 3.00E-09 | 15 | 47350689 |
| rs176644 | T | G | 0.408 | -0.0122 | 0.002 | 3.00E-09 | 15 | 89370401 |
| rs4947121 | T | C | 0.228 | -0.0142 | 0.0024 | 3.00E-09 | 6 | 111513751 |
| rs2939756 | A | G | 0.481 | -0.0119 | 0.002 | 3.00E-09 | 11 | 41414747 |
| rs6432686 | T | C | 0.712 | 0.0131 | 0.0022 | 3.00E-09 | 2 | 161468689 |
| rs16951001 | T | G | 0.418 | 0.0121 | 0.002 | 3.00E-09 | 15 | 67561903 |
| rs11165528 | T | C | 0.482 | -0.0128 | 0.0022 | 3.00E-09 | 1 | 95967238 |
| rs7333559 | A | G | 0.79 | -0.0157 | 0.0027 | 3.00E-09 | 13 | 99894196 |
| rs4680392 | T | C | 0.328 | -0.0136 | 0.0023 | 4.00E-09 | 3 | 157686540 |
| rs7160389 | T | C | 0.536 | -0.0119 | 0.002 | 4.00E-09 | 14 | 98184651 |
| rs772921 | T | C | 0.342 | -0.0125 | 0.0021 | 4.00E-09 | 12 | 56009793 |
| rs13234909 | A | G | 0.397 | 0.0121 | 0.0021 | 4.00E-09 | 7 | 1827295 |
| rs78058594 | A | G | 0.944 | 0.0278 | 0.0047 | 4.00E-09 | 11 | 132410392 |
| rs17348216 | T | C | 0.173 | -0.0156 | 0.0027 | 4.00E-09 | 5 | 104588673 |
| rs4354968 | A | T | 0.538 | 0.0118 | 0.002 | 4.00E-09 | 16 | 76434236 |
| rs10030552 | A | C | 0.26 | 0.0145 | 0.0025 | 4.00E-09 | 4 | 140051110 |
| rs12825376 | T | C | 0.116 | 0.0184 | 0.0031 | 4.00E-09 | 12 | 120742136 |
| rs6598539 | T | C | 0.488 | -0.0127 | 0.0022 | 4.00E-09 | 15 | 98661254 |
| rs7115027 | T | C | 0.848 | -0.0164 | 0.0028 | 4.00E-09 | 11 | 112896664 |
| rs11166986 | A | G | 0.49 | 0.0118 | 0.002 | 4.00E-09 | 8 | 140646056 |
| rs1981919 | A | G | 0.105 | 0.0192 | 0.0033 | 4.00E-09 | 15 | 47463885 |
| rs71367545 | A | G | 0.211 | 0.0156 | 0.0027 | 4.00E-09 | 18 | 79816337 |
| rs11771982 | T | C | 0.154 | -0.0163 | 0.0028 | 5.00E-09 | 7 | 132617489 |
| rs3800227 | A | G | 0.259 | -0.0134 | 0.0023 | 5.00E-09 | 6 | 108672958 |
| rs6452791 | A | C | 0.543 | 0.0118 | 0.002 | 5.00E-09 | 5 | 88475276 |
| rs56133711 | A | G | 0.262 | 0.0144 | 0.0025 | 5.00E-09 | 11 | 27701787 |
| rs72733235 | T | C | 0.838 | -0.0172 | 0.0029 | 5.00E-09 | 9 | 38275775 |
| rs1164598 | A | T | 0.621 | -0.012 | 0.0021 | 6.00E-09 | 13 | 96449317 |
| rs144528474 | A | C | 0.998 | 0.129 | 0.0222 | 6.00E-09 | 2 | 63650871 |
| rs11066971 | T | C | 0.145 | -0.0166 | 0.0028 | 6.00E-09 | 12 | 114165055 |
| rs11808210 | A | G | 0.078 | 0.0218 | 0.0038 | 6.00E-09 | 1 | 227327348 |
| rs3851186 | T | C | 0.212 | -0.0154 | 0.0027 | 7.00E-09 | 11 | 86220283 |
| rs35891966 | A | G | 0.072 | -0.0243 | 0.0042 | 7.00E-09 | 11 | 20107765 |
| rs12930834 | A | C | 0.763 | 0.0137 | 0.0024 | 7.00E-09 | 16 | 72917728 |
| rs4837631 | T | C | 0.452 | -0.0126 | 0.0022 | 7.00E-09 | 9 | 119299670 |
| rs6720647 | A | G | 0.76 | -0.0136 | 0.0024 | 7.00E-09 | 2 | 136858118 |
| rs6474609 | A | T | 0.588 | -0.0128 | 0.0022 | 7.00E-09 | 9 | 10981069 |
| rs11876432 | C | G | 0.751 | 0.0134 | 0.0023 | 7.00E-09 | 18 | 45089460 |
| rs962472 | T | C | 0.492 | -0.0116 | 0.002 | 8.00E-09 | 10 | 54940837 |
| rs78175438 | T | C | 0.871 | -0.0187 | 0.0032 | 8.00E-09 | 21 | 39291329 |
| rs2236941 | T | C | 0.334 | 0.0123 | 0.0021 | 8.00E-09 | 3 | 50210391 |
| rs55921136 | T | C | 0.797 | 0.0155 | 0.0027 | 8.00E-09 | 1 | 210185988 |
| rs7124348 | A | G | 0.766 | -0.0136 | 0.0024 | 9.00E-09 | 11 | 132515544 |
| rs288175 | A | G | 0.33 | -0.0122 | 0.0021 | 1.00E-08 | 5 | 108016378 |
| rs4479577 | T | C | 0.481 | 0.0124 | 0.0022 | 1.00E-08 | 3 | 5682131 |
| rs1174864 | A | G | 0.549 | 0.0116 | 0.002 | 1.00E-08 | 7 | 53059866 |
| rs12662631 | T | C | 0.319 | 0.0123 | 0.0022 | 1.00E-08 | 6 | 93132728 |
| rs9395644 | A | G | 0.898 | 0.0205 | 0.0036 | 1.00E-08 | 6 | 50955924 |
| rs6061162 | T | C | 0.907 | 0.0197 | 0.0035 | 1.00E-08 | 20 | 31305698 |
| rs9571576 | T | C | 0.506 | -0.0115 | 0.002 | 1.00E-08 | 13 | 66362001 |
| rs2222016 | C | G | 0.732 | 0.0129 | 0.0023 | 1.00E-08 | 2 | 136385737 |
| rs11779675 | A | G | 0.366 | -0.0128 | 0.0023 | 1.00E-08 | 8 | 10980951 |
| rs7130826 | T | G | 0.726 | 0.0128 | 0.0023 | 1.00E-08 | 11 | 17016246 |
| rs525444 | T | C | 0.79 | 0.0151 | 0.0027 | 1.00E-08 | 12 | 113673156 |
| rs745570 | A | G | 0.487 | 0.0114 | 0.002 | 1.00E-08 | 17 | 79807926 |
| rs10160101 | C | G | 0.402 | -0.0116 | 0.002 | 1.00E-08 | 10 | 13507462 |
| rs8047834 | A | G | 0.702 | 0.0125 | 0.0022 | 1.00E-08 | 16 | 25360395 |
| rs2185913 | T | C | 0.699 | 0.0124 | 0.0022 | 1.00E-08 | 10 | 113650475 |
| rs2783130 | A | G | 0.523 | 0.0114 | 0.002 | 1.00E-08 | 13 | 79596025 |
| rs4888444 | A | G | 0.948 | 0.0256 | 0.0045 | 2.00E-08 | 16 | 75656381 |
| rs10461104 | A | G | 0.634 | 0.0127 | 0.0023 | 2.00E-08 | 4 | 27488957 |
| rs11844846 | A | G | 0.346 | -0.0129 | 0.0023 | 2.00E-08 | 14 | 29018462 |
| rs117898875 | T | C | 0.962 | -0.0319 | 0.0056 | 2.00E-08 | 3 | 84223836 |
| rs553785 | A | G | 0.505 | 0.0122 | 0.0022 | 2.00E-08 | 1 | 96429769 |
| rs1449435 | A | G | 0.423 | 0.0123 | 0.0022 | 2.00E-08 | 11 | 132219023 |
| rs10020574 | C | G | 0.26 | 0.0128 | 0.0023 | 2.00E-08 | 4 | 28061344 |
| rs13160204 | A | G | 0.301 | 0.0123 | 0.0022 | 2.00E-08 | 5 | 158307754 |
| rs74873827 | A | T | 0.905 | 0.0208 | 0.0037 | 2.00E-08 | 2 | 145545337 |
| rs969650 | T | C | 0.643 | 0.0117 | 0.0021 | 2.00E-08 | 9 | 106615799 |
| rs16972552 | C | G | 0.884 | -0.0175 | 0.0031 | 2.00E-08 | 16 | 73776555 |
| rs239245 | A | C | 0.533 | -0.0112 | 0.002 | 2.00E-08 | 6 | 100642501 |
| rs12053870 | T | G | 0.458 | -0.0113 | 0.002 | 2.00E-08 | 3 | 118583668 |
| rs12757183 | T | C | 0.802 | 0.0141 | 0.0025 | 2.00E-08 | 1 | 216747031 |
| rs56081685 | T | G | 0.686 | 0.013 | 0.0023 | 2.00E-08 | 13 | 58880006 |
| rs7889 | C | G | 0.361 | 0.0126 | 0.0023 | 3.00E-08 | 6 | 31637671 |
| rs7645872 | T | C | 0.483 | 0.0121 | 0.0022 | 3.00E-08 | 3 | 25168310 |
| rs2652434 | T | C | 0.467 | -0.0112 | 0.002 | 3.00E-08 | 2 | 154886382 |
| rs10751226 | T | C | 0.826 | -0.0147 | 0.0026 | 3.00E-08 | 11 | 73601207 |
| rs905871 | A | G | 0.658 | 0.0117 | 0.0021 | 3.00E-08 | 11 | 4640055 |
| rs1271272 | A | G | 0.315 | -0.012 | 0.0022 | 3.00E-08 | 2 | 207090753 |
| rs55646170 | T | C | 0.92 | -0.0221 | 0.004 | 3.00E-08 | 1 | 98990375 |
| rs2358443 | T | C | 0.425 | -0.0112 | 0.002 | 3.00E-08 | 2 | 174065288 |
| rs3026629 | A | G | 0.348 | -0.0117 | 0.0021 | 3.00E-08 | 22 | 37933278 |
| rs6058078 | C | G | 0.485 | -0.012 | 0.0022 | 3.00E-08 | 20 | 34555019 |
| rs10252114 | T | C | 0.618 | -0.0123 | 0.0022 | 3.00E-08 | 7 | 122315414 |
| rs1150668 | T | G | 0.567 | 0.0112 | 0.002 | 3.00E-08 | 6 | 28162011 |
| rs3980078 | A | G | 0.179 | -0.0156 | 0.0028 | 3.00E-08 | 2 | 112570466 |
| rs6971782 | T | C | 0.32 | -0.0119 | 0.0022 | 3.00E-08 | 7 | 71061905 |
| rs28459916 | A | C | 0.138 | -0.0173 | 0.0031 | 4.00E-08 | 4 | 79409589 |
| rs12476173 | A | G | 0.638 | 0.0115 | 0.0021 | 4.00E-08 | 2 | 117490138 |
| rs7295765 | A | C | 0.797 | -0.0149 | 0.0027 | 4.00E-08 | 12 | 86065262 |
| rs4312833 | A | T | 0.089 | -0.0194 | 0.0035 | 4.00E-08 | 5 | 51686320 |
| rs143427404 | A | G | 0.011 | 0.0569 | 0.0104 | 4.00E-08 | 3 | 52826860 |
| rs301805 | T | G | 0.423 | -0.0112 | 0.002 | 4.00E-08 | 1 | 8420956 |
| rs12456711 | A | G | 0.735 | -0.0135 | 0.0025 | 4.00E-08 | 18 | 280897 |
| rs2633725 | T | C | 0.153 | 0.0153 | 0.0028 | 4.00E-08 | 3 | 53717715 |
| rs1944688 | A | G | 0.779 | 0.0133 | 0.0024 | 4.00E-08 | 11 | 121764755 |
| rs12997449 | A | G | 0.874 | -0.0179 | 0.0033 | 4.00E-08 | 2 | 178174845 |
| rs9388686 | A | C | 0.177 | -0.0144 | 0.0026 | 4.00E-08 | 6 | 129104419 |
| rs7901883 | A | G | 0.231 | -0.0131 | 0.0024 | 4.00E-08 | 10 | 101427081 |
| rs1022261 | A | C | 0.506 | -0.011 | 0.002 | 4.00E-08 | 4 | 35474678 |
| rs58695218 | A | G | 0.81 | 0.0151 | 0.0028 | 4.00E-08 | 7 | 39392731 |
| rs12517438 | T | G | 0.459 | -0.011 | 0.002 | 4.00E-08 | 5 | 30841947 |
| rs6438208 | A | G | 0.254 | -0.0126 | 0.0023 | 4.00E-08 | 3 | 114451425 |
| rs550677 | A | G | 0.428 | 0.0111 | 0.002 | 4.00E-08 | 12 | 125326510 |
| rs146127221 | T | C | 0.02 | 0.0422 | 0.0077 | 4.00E-08 | 10 | 85705377 |
| rs138045331 | A | C | 0.994 | -0.0751 | 0.0137 | 4.00E-08 | 2 | 122082314 |
| rs10279261 | A | G | 0.617 | -0.0113 | 0.0021 | 5.00E-08 | 7 | 133905093 |
| rs550612 | A | C | 0.727 | 0.0123 | 0.0023 | 5.00E-08 | 2 | 136893930 |
| rs71471272 | A | G | 0.075 | 0.0225 | 0.0041 | 5.00E-08 | 10 | 103245744 |
| rs12895462 | T | C | 0.804 | 0.0138 | 0.0025 | 5.00E-08 | 14 | 77149098 |
| rs7575189 | A | G | 0.587 | -0.0111 | 0.002 | 5.00E-08 | 2 | 173150440 |
| **T2DM** | | | | | | | | |
| rs10077431 | A | C | 0.2147 | -0.0487 | 0.0089 | 5.00E-08 | 5 | 113591989 |
| rs10087241 | G | A | 0.4051 | 0.0475 | 0.008 | 3.00E-09 | 8 | 31006206 |
| rs1009358 | C | T | 0.3773 | -0.0545 | 0.008 | 1.00E-11 | 2 | 65049318 |
| rs10100265 | A | C | 0.3895 | 0.0491 | 0.0079 | 6.00E-10 | 8 | 10775649 |
| rs10114341 | C | T | 0.4408 | -0.0409 | 0.0072 | 1.00E-08 | 9 | 94156900 |
| rs10169613 | T | C | 0.4721 | -0.0429 | 0.0078 | 4.00E-08 | 2 | 111177400 |
| rs10401969 | C | T | 0.0766 | 0.0921 | 0.0133 | 4.00E-12 | 19 | 19296909 |
| rs1050226 | G | A | 0.4068 | -0.0491 | 0.0074 | 3.00E-11 | 6 | 7281421 |
| rs1061813 | G | A | 0.4629 | 0.0429 | 0.0073 | 3.00E-09 | 5 | 14847222 |
| rs1063192 | G | A | 0.4403 | -0.0634 | 0.0073 | 3.00E-18 | 9 | 22003368 |
| rs1063355 | T | G | 0.3976 | -0.0709 | 0.0079 | 4.00E-19 | 6 | 32659937 |
| rs10811661 | C | T | 0.1736 | -0.1569 | 0.0098 | 4.00E-58 | 9 | 22134095 |
| rs10830963 | G | C | 0.2758 | 0.0909 | 0.008 | 6.00E-30 | 11 | 92975544 |
| rs10842994 | T | C | 0.197 | -0.0755 | 0.0091 | 1.00E-16 | 12 | 27812217 |
| rs10974438 | C | A | 0.3512 | 0.0591 | 0.0075 | 3.00E-15 | 9 | 4291928 |
| rs11048456 | C | T | 0.2438 | 0.0488 | 0.0082 | 3.00E-09 | 12 | 26310149 |
| rs11098676 | T | C | 0.2124 | -0.054 | 0.0096 | 2.00E-08 | 4 | 122911999 |
| rs11107116 | T | G | 0.2197 | 0.0467 | 0.0085 | 4.00E-08 | 12 | 93584728 |
| rs11257655 | T | C | 0.2068 | 0.0737 | 0.0087 | 2.00E-17 | 10 | 12265895 |
| rs1127655 | C | T | 0.4709 | 0.0438 | 0.0079 | 2.00E-08 | 1 | 116987885 |
| rs11591741 | C | G | 0.4404 | -0.0475 | 0.0078 | 1.00E-09 | 10 | 100216744 |
| rs11651755 | C | T | 0.4847 | 0.0741 | 0.0077 | 9.00E-22 | 17 | 37739849 |
| rs11708067 | G | A | 0.239 | -0.0965 | 0.0086 | 6.00E-29 | 3 | 123346931 |
| rs117229942 | C | T | 0.99182 | 0.357 | 0.054 | 4.00E-11 | 10 | 112978018 |
| rs11774915 | T | C | 0.3371 | 0.0495 | 0.0086 | 9.00E-09 | 8 | 9331252 |
| rs11925227 | A | G | 0.1834 | -0.0534 | 0.0095 | 2.00E-08 | 3 | 171048829 |
| rs11926707 | T | C | 0.3744 | -0.0463 | 0.0082 | 2.00E-08 | 3 | 46884049 |
| rs12088739 | G | A | 0.0898 | -0.0884 | 0.013 | 1.00E-11 | 1 | 51041214 |
| rs12299509 | G | A | 0.4786 | 0.0467 | 0.0073 | 2.00E-10 | 12 | 4297115 |
| rs12617659 | T | C | 0.1472 | -0.0685 | 0.0103 | 3.00E-11 | 2 | 120552183 |
| rs12681990 | C | T | 0.1637 | 0.0634 | 0.0096 | 4.00E-11 | 8 | 37001668 |
| rs12910825 | G | A | 0.3604 | 0.0517 | 0.0074 | 2.00E-12 | 15 | 90968030 |
| rs12945601 | T | C | 0.3864 | 0.048 | 0.008 | 2.00E-09 | 17 | 17750097 |
| rs12970134 | A | G | 0.2651 | 0.0555 | 0.008 | 5.00E-12 | 18 | 60217517 |
| rs13234269 | A | T | 0.4925 | -0.0583 | 0.0078 | 7.00E-14 | 7 | 130744427 |
| rs13239186 | T | C | 0.302 | 0.0539 | 0.0085 | 3.00E-10 | 7 | 117870567 |
| rs13389219 | T | C | 0.3944 | -0.0722 | 0.0074 | 2.00E-22 | 2 | 164672366 |
| rs1359790 | A | G | 0.2867 | -0.0796 | 0.008 | 3.00E-23 | 13 | 80143021 |
| rs146886108 | C | T | 0.99286 | 0.342 | 0.058 | 4.00E-09 | 5 | 14751196 |
| rs1480474 | G | A | 0.416 | 0.0411 | 0.0073 | 2.00E-08 | 12 | 65933163 |
| rs1496653 | G | A | 0.2048 | -0.0769 | 0.0088 | 3.00E-18 | 3 | 23413299 |
| rs1552224 | C | A | 0.1542 | -0.1034 | 0.0101 | 9.00E-25 | 11 | 72722053 |
| rs16988333 | G | A | 0.0904 | -0.0745 | 0.013 | 9.00E-09 | 22 | 30156824 |
| rs17086692 | T | G | 0.3134 | -0.0467 | 0.0084 | 2.00E-08 | 4 | 52268127 |
| rs17168486 | T | C | 0.1736 | 0.0742 | 0.0094 | 2.00E-15 | 7 | 14858657 |
| rs17334919 | T | C | 0.1002 | -0.1398 | 0.0128 | 7.00E-28 | 2 | 43480246 |
| rs17405722 | A | G | 0.0742 | 0.087 | 0.0146 | 2.00E-09 | 17 | 42390483 |
| rs17411031 | G | C | 0.2617 | -0.045 | 0.0081 | 3.00E-08 | 8 | 19994799 |
| rs1758632 | C | G | 0.3766 | -0.0491 | 0.0081 | 1.00E-09 | 9 | 34025642 |
| rs17631783 | T | C | 0.2635 | -0.0487 | 0.0089 | 4.00E-08 | 17 | 63610240 |
| rs17791483 | G | A | 0.0626 | -0.102 | 0.0147 | 3.00E-12 | 9 | 79284065 |
| rs1801214 | C | T | 0.4004 | -0.0903 | 0.0074 | 6.00E-34 | 4 | 6301295 |
| rs184847416 | T | C | 1.20E-04 | 1.264 | 0.221 | 1.00E-08 | 2 | 136287660 |
| rs1899951 | T | C | 0.1233 | -0.1118 | 0.0109 | 2.00E-24 | 3 | 12353341 |
| rs2071479 | T | C | 0.0274 | 0.1473 | 0.0226 | 7.00E-11 | 6 | 32813335 |
| rs2191348 | G | T | 0.4531 | -0.0652 | 0.0073 | 3.00E-19 | 7 | 15024630 |
| rs2237892 | T | C | 0.0625 | -0.096 | 0.0157 | 9.00E-10 | 11 | 2818521 |
| rs2246012 | C | T | 0.1648 | 0.0527 | 0.0094 | 2.00E-08 | 6 | 131577068 |
| rs2261181 | T | C | 0.0965 | 0.0985 | 0.0118 | 9.00E-17 | 12 | 65818538 |
| rs2292662 | T | C | 0.1513 | -0.0629 | 0.0111 | 1.00E-08 | 3 | 63911539 |
| rs2294120 | G | A | 0.4559 | -0.0443 | 0.0079 | 2.00E-08 | 8 | 144778182 |
| rs2296173 | G | A | 0.212 | 0.065 | 0.0087 | 8.00E-14 | 1 | 39447679 |
| rs2299383 | T | C | 0.4235 | 0.0412 | 0.0073 | 1.00E-08 | 7 | 103778399 |
| rs2307111 | C | T | 0.3973 | -0.0407 | 0.0074 | 3.00E-08 | 5 | 75707853 |
| rs2421016 | T | C | 0.4738 | -0.0458 | 0.0071 | 1.00E-10 | 10 | 122407996 |
| rs243019 | C | T | 0.4558 | 0.0566 | 0.0071 | 2.00E-15 | 2 | 60358671 |
| rs244415 | A | G | 0.4129 | -0.0467 | 0.0079 | 4.00E-09 | 16 | 69632780 |
| rs2493394 | G | A | 0.1073 | 0.073 | 0.0113 | 1.00E-10 | 1 | 119928601 |
| rs2616132 | A | G | 0.4743 | 0.0455 | 0.0078 | 7.00E-09 | 10 | 69709758 |
| rs2633310 | T | G | 0.4351 | -0.0443 | 0.0079 | 2.00E-08 | 10 | 73834292 |
| rs2796441 | A | G | 0.4165 | -0.0715 | 0.0073 | 2.00E-22 | 9 | 81694033 |
| rs2820426 | A | G | 0.3899 | -0.0521 | 0.0073 | 1.00E-12 | 1 | 219487193 |
| rs2857605 | C | T | 0.2106 | -0.0672 | 0.009 | 6.00E-14 | 6 | 31557074 |
| rs2867125 | T | C | 0.1722 | -0.0601 | 0.0096 | 4.00E-10 | 2 | 622827 |
| rs2908282 | A | G | 0.1774 | 0.0552 | 0.0094 | 4.00E-09 | 7 | 44209229 |
| rs2925979 | T | C | 0.2991 | 0.0534 | 0.0078 | 9.00E-12 | 16 | 81501185 |
| rs2972144 | A | G | 0.3546 | -0.0913 | 0.0075 | 3.00E-34 | 2 | 226236695 |
| rs302864 | A | G | 0.087 | 0.071 | 0.0127 | 2.00E-08 | 17 | 58680223 |
| rs340874 | T | C | 0.4361 | -0.0626 | 0.0073 | 8.00E-18 | 1 | 213985913 |
| rs348330 | G | A | 0.3665 | 0.0487 | 0.0081 | 2.00E-09 | 1 | 229537208 |
| rs3802177 | A | G | 0.3113 | -0.1217 | 0.008 | 2.00E-52 | 8 | 117172786 |
| rs3887925 | C | T | 0.4468 | -0.0474 | 0.0079 | 2.00E-09 | 3 | 186947857 |
| rs3900856 | A | G | 0.0392 | 0.1136 | 0.0185 | 7.00E-10 | 5 | 56538065 |
| rs4472028 | T | C | 0.4442 | 0.0453 | 0.0071 | 2.00E-10 | 3 | 152335461 |
| rs4502156 | C | T | 0.4359 | -0.0411 | 0.0073 | 2.00E-08 | 15 | 62090956 |
| rs459193 | A | G | 0.2547 | -0.0711 | 0.0083 | 9.00E-18 | 5 | 56510924 |
| rs4810426 | T | C | 0.0968 | 0.0726 | 0.013 | 2.00E-08 | 20 | 44373081 |
| rs4823182 | G | A | 0.3357 | 0.0482 | 0.0077 | 3.00E-10 | 22 | 43981562 |
| rs4865796 | G | A | 0.3069 | -0.053 | 0.0078 | 1.00E-11 | 5 | 53976834 |
| rs4918796 | C | T | 0.2226 | 0.0623 | 0.0086 | 4.00E-13 | 10 | 113120583 |
| rs4932143 | G | C | 0.2791 | 0.0568 | 0.0087 | 6.00E-11 | 15 | 89828835 |
| rs516946 | T | C | 0.2394 | -0.0824 | 0.0085 | 3.00E-22 | 8 | 41661730 |
| rs5215 | C | T | 0.3601 | 0.0678 | 0.0073 | 2.00E-20 | 11 | 17387083 |
| rs527320094 | C | A | 2.30E-04 | 1.019 | 0.174 | 5.00E-09 | 1 | 72387303 |
| rs551513405 | A | G | 1.60E-04 | 1.115 | 0.199 | 2.00E-08 | 7 | 56003690 |
| rs551640889 | G | C | 1.20E-04 | 1.284 | 0.22 | 5.00E-09 | 16 | 17166334 |
| rs559651557 | C | T | 2.30E-04 | 0.982 | 0.177 | 3.00E-08 | 14 | 36430441 |
| rs576083050 | T | C | 3.50E-04 | 0.85 | 0.153 | 3.00E-08 | 12 | 450654 |
| rs576674 | G | A | 0.1675 | 0.0654 | 0.0097 | 2.00E-11 | 13 | 32980164 |
| rs6059662 | A | G | 0.3368 | -0.0446 | 0.0079 | 2.00E-08 | 20 | 34087921 |
| rs6066138 | A | G | 0.2784 | -0.049 | 0.0082 | 2.00E-09 | 20 | 46966072 |
| rs61953351 | T | G | 0.2499 | -0.07 | 0.0091 | 2.00E-14 | 12 | 121018813 |
| rs622217 | C | T | 0.4839 | -0.0485 | 0.0077 | 3.00E-10 | 6 | 160345738 |
| rs6515236 | C | A | 0.2493 | -0.0504 | 0.0091 | 3.00E-08 | 20 | 22455111 |
| rs67232546 | T | C | 0.2092 | 0.0596 | 0.0096 | 5.00E-10 | 11 | 128529043 |
| rs6795735 | T | C | 0.4109 | -0.0558 | 0.0073 | 2.00E-14 | 3 | 64719689 |
| rs6808574 | T | C | 0.3904 | -0.0552 | 0.0076 | 4.00E-13 | 3 | 188022735 |
| rs687621 | G | A | 0.3248 | 0.0433 | 0.0076 | 1.00E-08 |  |  |
| rs6878122 | G | A | 0.3182 | 0.0564 | 0.0079 | 1.00E-12 | 5 | 77131486 |
| rs7138300 | C | T | 0.4432 | 0.0443 | 0.0072 | 6.00E-10 | 12 | 71045809 |
| rs7144011 | T | G | 0.2211 | 0.0482 | 0.0085 | 2.00E-08 | 14 | 79474040 |
| rs7177055 | G | A | 0.2817 | -0.0647 | 0.0079 | 3.00E-16 | 15 | 77540420 |
| rs7185735 | G | A | 0.3973 | 0.1056 | 0.0073 | 2.00E-47 | 16 | 53788739 |
| rs7240767 | C | T | 0.3837 | 0.0451 | 0.0081 | 2.00E-08 | 18 | 7070643 |
| rs72892910 | T | G | 0.1724 | 0.0648 | 0.0099 | 6.00E-11 | 6 | 50849174 |
| rs735949 | C | T | 0.1411 | -0.0711 | 0.0106 | 2.00E-11 | 4 | 184795078 |
| rs753270 | T | C | 0.4165 | -0.0528 | 0.0079 | 3.00E-11 | 10 | 79205218 |
| rs7561798 | G | A | 0.4822 | 0.04 | 0.0072 | 3.00E-08 | 2 | 228108944 |
| rs7572970 | A | G | 0.278 | -0.059 | 0.0087 | 1.00E-11 | 2 | 160280145 |
| rs7651090 | G | A | 0.3134 | 0.1204 | 0.0076 | 4.00E-57 | 3 | 185795604 |
| rs7674212 | T | G | 0.4089 | -0.0465 | 0.0075 | 6.00E-10 | 4 | 103067742 |
| rs7685296 | T | C | 0.2794 | -0.0511 | 0.0081 | 2.00E-10 | 4 | 152332969 |
| rs77258096 | A | C | 0.1014 | -0.1171 | 0.0134 | 2.00E-18 | 16 | 75209874 |
| rs7729395 | T | C | 0.0509 | 0.1373 | 0.016 | 1.00E-17 | 5 | 102764872 |
| rs7756992 | G | A | 0.2669 | 0.1297 | 0.0078 | 6.00E-62 | 6 | 20679478 |
| rs7786095 | G | A | 0.1039 | -0.0743 | 0.0129 | 1.00E-08 | 7 | 157191153 |
| rs780094 | T | C | 0.3872 | -0.0692 | 0.0074 | 5.00E-21 | 2 | 27518370 |
| rs78408340 | G | C | 0.0096 | 0.293 | 0.039 | 4.00E-14 | 5 | 103003035 |
| rs7841082 | T | C | 0.4383 | -0.042 | 0.0077 | 5.00E-08 | 8 | 8311465 |
| rs7845219 | C | T | 0.4928 | -0.0422 | 0.0072 | 5.00E-09 | 8 | 94925274 |
| rs7903146 | T | C | 0.2916 | 0.3059 | 0.0077 | 1E-347 | 10 | 112998590 |
| rs7923866 | T | C | 0.3788 | -0.0972 | 0.0074 | 9.00E-40 | 10 | 92722319 |
| rs7929543 | C | A | 0.0832 | 0.0828 | 0.0138 | 2.00E-09 | 11 | 49329474 |
| rs7931302 | C | A | 0.2769 | 0.0461 | 0.008 | 8.00E-09 | 11 | 128366163 |
| rs79768058 | G | T | 2.00E-04 | 1.054 | 0.184 | 1.00E-08 | 9 | 132436281 |
| rs79890196 | C | G | 2.60E-04 | 0.956 | 0.17 | 2.00E-08 | 15 | 95945146 |
| rs8068804 | A | G | 0.3251 | 0.0587 | 0.0078 | 4.00E-14 | 17 | 4082570 |
| rs8108269 | G | T | 0.281 | 0.0644 | 0.0079 | 3.00E-16 | 19 | 45655255 |
| rs825476 | C | T | 0.4195 | -0.0524 | 0.0073 | 7.00E-13 | 12 | 124083909 |
| rs849135 | G | A | 0.5009 | 0.0999 | 0.0072 | 1.00E-43 | 7 | 28156794 |
| rs853974 | T | C | 0.2624 | 0.0601 | 0.0088 | 8.00E-12 | 6 | 126747838 |
| rs9369425 | G | A | 0.2918 | 0.0546 | 0.0085 | 1.00E-10 | 6 | 43843237 |
| rs940904 | G | A | 0.2566 | -0.0499 | 0.0083 | 2.00E-09 | 12 | 123007025 |
| rs963740 | T | A | 0.2943 | -0.0479 | 0.0086 | 2.00E-08 | 13 | 50521959 |
| rs982077 | A | G | 0.4345 | 0.0453 | 0.0072 | 3.00E-10 | 15 | 63531102 |
| rs9844972 | C | G | 0.0697 | 0.0956 | 0.0148 | 1.00E-10 | 3 | 150379848 |
| rs9894220 | G | A | 0.4337 | -0.0585 | 0.0079 | 2.00E-13 | 17 | 48911792 |
| rs9911983 | C | T | 0.4334 | -0.0397 | 0.0073 | 5.00E-08 | 17 | 47808390 |
| rs993380 | A | G | 0.3344 | 0.0507 | 0.0081 | 5.00E-10 | 4 | 82663343 |
| rs9940149 | A | G | 0.1786 | -0.058 | 0.0095 | 9.00E-10 | 16 | 250642 |
| **Testosterone levels** | | | | | | | | |
| rs9692804 | A | G | 0.426 | 0.048 | 0.01 | 4.00E-06 | 8 | 142977208 |
| rs9989479 | G | A | 0.964 | 0.149 | 0.028 | 2.00E-07 | 17 | 7514792 |
| rs11078696 | T | G | 0.805 | 0.083 | 0.014 | 4.00E-09 | 17 | 7555982 |
| rs858516 | T | C | 0.584 | 0.06 | 0.011 | 2.00E-08 | 17 | 7633780 |
| rs35894069 | A | G | 0.347 | 0.069 | 0.011 | 5.00E-10 | 17 | 7491857 |
| rs62059803 | T | C | 0.22 | 0.085 | 0.013 | 5.00E-11 | 17 | 7548527 |
| rs34289079 | C | T | 0.096 | -0.106 | 0.018 | 4.00E-09 | 17 | 7690001 |
| [rs17551157](https://www.ncbi.nlm.nih.gov/SNP/snp_ref.cgi?rs=17551157) | CGGGG | C | 0.075 | -0.111 | 0.02 | 4.00E-08 | 17 | 7591511 |
| rs3867595 | C | G | 0.017 | -0.231 | 0.041 | 1.00E-08 | 17 | 7476457 |
| **TG** | | | | | | | | |
| rs114139997 | A | G | 0.0193 | -0.2399 | 0.0216 | 1.00E-300 | 21 | 45455861 |
| rs7350481 | T | C | 0.0708 | 0.2272 | 0.0055 | 1.00E-09 | 11 | 116715567 |
| rs38246 | T | C | 0.2494 | 0.0164 | 0.0027 | 1.00E-10 | 7 | 15901658 |
| rs13266634 | T | C | 0.2822 | -0.0134 | 0.0021 | 1.00E-09 | 8 | 117172544 |
| rs2603151 | A | G | 0.3612 | 0.0168 | 0.0028 | 1.00E-300 | 4 | 68502154 |
| rs1260326 | T | C | 0.379 | 0.1118 | 0.0028 | 1.00E-14 | 2 | 27508073 |
| rs6602911 | T | C | 0.4294 | 0.0216 | 0.0028 | 1.00E-08 | 13 | 113844399 |
| rs1788783 | T | C | 0.504 | -0.012 | 0.0021 | 1.00E-19 | 18 | 23581170 |
| rs111524356 | D | I | 0.5634 | 0.025 | 0.0027 | 1.00E-22 | 2 | 65057023 |
| rs7120118 | T | C | 0.6572 | 0.0275 | 0.0028 | 1.00E-08 | 11 | 47264739 |
| rs11745702 | T | C | 0.7608 | 0.0193 | 0.0034 | 1.00E-300 | 5 | 133108609 |
| rs1569209 | T | G | 0.9206 | 0.2112 | 0.0053 | 1.00E-300 | 8 | 19972659 |
| rs11720145 | A | G | 0.2337 | 0.0223 | 0.003 | 1.00E-13 | 3 | 171021874 |
| rs11638671 | T | C | 0.6684 | -0.0272 | 0.003 | 1.00E-19 | 15 | 63503429 |
| rs9657541 | T | C | 0.1885 | 0.0305 | 0.0034 | 1.00E-19 | 8 | 10785654 |
| rs202032561 | T | C | 0.1315 | -0.0331 | 0.0051 | 1.00E-10 | 2 | 43520715 |
| rs540747651 | A | G | 8.00E-04 | 0.8716 | 0.1526 | 1.00E-08 | 2 | 117986228 |
| rs56271783 | C | G | 0.0448 | 0.0572 | 0.0069 | 1.00E-16 | 11 | 64237251 |
| rs150090666 | T | C | 9.00E-04 | -0.2779 | 0.0487 | 1.00E-08 | 11 | 14843853 |
| rs148149124 | D | I | 0.0281 | 0.1011 | 0.0097 | 1.00E-25 | 15 | 43382233 |
| rs6532795 | T | C | 0.2812 | -0.0168 | 0.003 | 1.00E-08 | 4 | 99121070 |
| rs13108218 | A | G | 0.4233 | 0.0234 | 0.0028 | 1.00E-16 | 4 | 3442204 |
| rs1884507 | A | C | 0.8759 | 0.0248 | 0.0038 | 1.00E-10 | 20 | 52395625 |
| rs1564282 | T | C | 0.0934 | 0.0224 | 0.0033 | 1.00E-11 | 4 | 858525 |
| rs10853981 | A | G | 0.3705 | 0.015 | 0.0022 | 1.00E-11 | 19 | 4965053 |
| rs7176058 | A | G | 0.7559 | 0.0152 | 0.0027 | 1.00E-08 | 15 | 39171966 |
| rs41278045 | A | G | 0.9987 | -0.2008 | 0.0283 | 1.00E-12 | 4 | 109717608 |
| rs727428 | T | C | 0.4508 | 0.0187 | 0.0028 | 1.00E-11 | 17 | 7634474 |
| rs5785580 | D | I | 0.4678 | -0.0291 | 0.0027 | 1.00E-26 | 10 | 63530495 |
| rs12257692 | T | C | 0.799 | -0.0283 | 0.0047 | 1.00E-09 | 10 | 72944091 |
| rs200513066 | D | I | 0.0508 | 0.0883 | 0.0095 | 1.00E-20 | 2 | 218856230 |
| rs4738141 | A | G | 0.6374 | -0.0149 | 0.0025 | 1.00E-09 | 8 | 71557507 |
| rs1332263671 | D | I | 0.2349 | -0.0283 | 0.0038 | 1.00E-13 | 17 | 46232429 |
| rs34078567 | D | I | 0.6105 | 0.065 | 0.0029 | 1.00E-112 | 19 | 44909968 |
| rs1051424 | A | G | 0.8238 | -0.015 | 0.0025 | 1.00E-09 | 17 | 59946963 |
| rs2980755 | A | G | 0.5437 | 0.0209 | 0.0033 | 1.00E-10 | 8 | 8506173 |
| rs2281721 | T | C | 0.5522 | -0.0377 | 0.0028 | 1.00E-40 | 1 | 230161390 |
| rs174537 | T | G | 0.312 | 0.0617 | 0.0031 | 2.00E-86 | 11 | 61785208 |
| rs2018519 | T | C | 0.8433 | -0.0212 | 0.003 | 2.00E-12 | 19 | 35068883 |
| rs2298117 | T | C | 0.4895 | 0.0111 | 0.0018 | 2.00E-09 | 10 | 68586983 |
| rs12460070 | A | T | 0.6562 | -0.0184 | 0.0029 | 2.00E-10 | 19 | 33442547 |
| rs55738118 | T | C | 0.1089 | -0.0208 | 0.0037 | 2.00E-08 | 1 | 11778394 |
| rs11296991 | D | I | 0.307 | 0.0243 | 0.003 | 2.00E-15 | 5 | 54012825 |
| rs7502307 | C | G | 0.7958 | -0.0244 | 0.0034 | 2.00E-12 | 17 | 67993845 |
| rs139441335 | A | G | 0.969 | 0.075 | 0.0091 | 2.00E-16 | 7 | 73748480 |
| rs4149056 | T | C | 0.8516 | -0.0335 | 0.0039 | 2.00E-17 | 12 | 21178615 |
| rs11780610 | T | C | 0.7089 | -0.0362 | 0.0029 | 2.00E-35 | 8 | 18402366 |
| rs17600346 | T | C | 0.9621 | -0.0472 | 0.0067 | 2.00E-12 | 3 | 172506192 |
| rs1079596 | T | C | 0.1767 | 0.0141 | 0.0025 | 2.00E-08 | 11 | 113425897 |
| rs746011 | T | C | 0.3037 | 0.0173 | 0.0027 | 2.00E-10 | 8 | 22600291 |
| rs1982151 | A | G | 0.2953 | -0.0115 | 0.002 | 2.00E-08 | 9 | 84002350 |
| rs538276778 | D | I | 0.7875 | -0.0357 | 0.0033 | 2.00E-27 | 20 | 44557215 |
| rs382534 | T | C | 0.2379 | 0.0149 | 0.0025 | 2.00E-09 | 3 | 155829485 |
| rs2914228 | T | C | 0.3265 | 0.024 | 0.0027 | 2.00E-19 | 5 | 158580157 |
| rs73081656 | A | C | 0.3342 | -0.0186 | 0.0031 | 2.00E-09 | 7 | 25896127 |
| rs2354167 | T | G | 0.4443 | 0.0144 | 0.0024 | 2.00E-09 | 3 | 142938363 |
| rs58253018 | A | T | 0.3738 | 0.0984 | 0.0034 | 2.00E-182 | 8 | 125478256 |
| rs2207132 | A | G | 0.0275 | 0.0833 | 0.0118 | 2.00E-12 | 20 | 40513876 |
| rs1519104 | A | G | 0.6821 | -0.016 | 0.0025 | 2.00E-10 | 2 | 66453760 |
| rs12454712 | T | C | 0.6503 | 0.0128 | 0.0023 | 2.00E-08 | 18 | 63178651 |
| rs998584 | A | C | 0.4455 | 0.0431 | 0.0028 | 2.00E-54 | 6 | 43790159 |
| rs573454216 | A | G | 0.5604 | -0.0262 | 0.0028 | 2.00E-21 | 6 | 139516292 |
| rs576907609 | A | C | 0.003 | -0.3111 | 0.0407 | 2.00E-14 | 11 | 117628414 |
| rs75601653 | C | G | 0.6799 | -0.0171 | 0.003 | 2.00E-08 | 11 | 65715899 |
| rs73165526 | T | C | 0.9169 | -0.0306 | 0.0051 | 2.00E-09 | 7 | 150834187 |
| rs11340914 | D | I | 0.6579 | 0.02 | 0.003 | 2.00E-11 | 1 | 23421504 |
| rs6544366 | T | G | 0.2243 | -0.0549 | 0.0032 | 2.00E-67 | 2 | 20981153 |
| rs1133400 | A | G | 0.7981 | -0.0131 | 0.0023 | 2.00E-08 | 10 | 132645884 |
| rs1913657 | T | C | 0.6215 | 0.0433 | 0.0028 | 2.00E-55 | 2 | 226242785 |
| rs55951234 | D | I | 0.4087 | -0.0268 | 0.0028 | 2.00E-22 | 22 | 38205424 |
| rs12206516 | A | G | 0.8218 | 0.0154 | 0.0028 | 2.00E-08 | 6 | 20506584 |
| rs28567725 | T | C | 0.6227 | -0.0211 | 0.0028 | 2.00E-14 | 16 | 53792116 |
| rs871290 | C | G | 0.323 | 0.0139 | 0.0025 | 2.00E-08 | 16 | 85673761 |
| rs2613503 | A | C | 0.8334 | 0.0178 | 0.0028 | 2.00E-10 | 1 | 72374091 |
| rs6430090 | A | G | 0.3706 | 0.0161 | 0.0024 | 2.00E-11 | 2 | 145589891 |
| rs12893623 | A | G | 0.6853 | -0.0172 | 0.0029 | 3.00E-09 | 14 | 103824476 |
| rs2298058 | T | C | 0.3135 | 0.0245 | 0.0026 | 3.00E-20 | 13 | 94596312 |
| rs2971672 | A | C | 0.5854 | -0.0178 | 0.0028 | 3.00E-10 | 7 | 44166307 |
| rs3118359 | T | G | 0.0838 | -0.0378 | 0.0052 | 3.00E-13 | 6 | 28826127 |
| rs10184004 | T | C | 0.453 | -0.0309 | 0.0028 | 3.00E-29 | 2 | 164651879 |
| rs10846744 | C | G | 0.3084 | 0.0252 | 0.0033 | 3.00E-14 | 12 | 124827879 |
| rs11247287 | T | C | 0.6649 | -0.0127 | 0.0023 | 3.00E-08 | 15 | 101365328 |
| rs2068888 | A | G | 0.4268 | -0.0296 | 0.0027 | 3.00E-28 | 10 | 93079885 |
| rs4709741 | A | C | 0.1249 | -0.022 | 0.004 | 3.00E-08 | 6 | 163671259 |
| rs730180 | A | G | 0.7356 | 0.015 | 0.0025 | 3.00E-09 | 15 | 72715552 |
| rs2925979 | T | C | 0.2951 | 0.0181 | 0.0029 | 3.00E-10 | 16 | 81501185 |
| rs17779355 | A | G | 0.0578 | -0.0292 | 0.0046 | 3.00E-10 | 10 | 102168617 |
| rs74563318 | A | C | 0.0311 | -0.0495 | 0.0089 | 3.00E-08 | 10 | 112365219 |
| rs193220 | T | C | 0.2956 | -0.0189 | 0.003 | 3.00E-10 | 17 | 76272538 |
| rs72663520 | T | C | 0.2135 | 0.0225 | 0.0036 | 3.00E-10 | 1 | 39537738 |
| rs59168178 | A | G | 0.0818 | 0.0767 | 0.0101 | 3.00E-14 | 19 | 11239650 |
| rs13214992 | A | G | 0.0562 | 0.1055 | 0.0094 | 3.00E-29 | 6 | 32632808 |
| rs6088793 | T | C | 0.7225 | 0.0162 | 0.0029 | 3.00E-08 | 20 | 31623794 |
| rs247616 | T | C | 0.3071 | -0.0255 | 0.0028 | 3.00E-19 | 16 | 56955678 |
| rs62112763 | C | G | 0.6009 | -0.0221 | 0.0029 | 3.00E-14 | 19 | 7220002 |
| rs10793310 | T | G | 0.8332 | 0.0193 | 0.0029 | 3.00E-11 | 11 | 78387968 |
| rs149793040 | A | G | 0.9987 | 0.2148 | 0.0389 | 3.00E-08 | 12 | 109223867 |
| rs1077835 | A | G | 0.6836 | -0.021 | 0.003 | 3.00E-12 | 15 | 58431227 |
| rs2821231 | T | C | 0.4417 | 0.0146 | 0.0025 | 3.00E-09 | 1 | 203549254 |
| rs41265930 | T | C | 0.9445 | -0.0488 | 0.0067 | 4.00E-13 | 6 | 160543103 |
| rs10199914 | A | G | 0.6285 | -0.0112 | 0.0019 | 4.00E-09 | 2 | 238975165 |
| rs140868651 | D | I | 0.0608 | -0.0924 | 0.006 | 4.00E-53 | 19 | 19321482 |
| rs72735627 | T | C | 0.0672 | -0.0362 | 0.0066 | 4.00E-08 | 15 | 40765309 |
| rs41302559 | A | G | 0.0027 | -0.152 | 0.0276 | 4.00E-08 | 20 | 57565383 |
| rs13234131 | A | G | 0.8853 | 0.1275 | 0.0043 | 4.00E-191 | 7 | 73611645 |
| rs12208493 | T | C | 0.707 | -0.0134 | 0.0023 | 4.00E-09 | 6 | 85952549 |
| rs9817452 | T | G | 0.3753 | -0.0292 | 0.0029 | 4.00E-23 | 3 | 157077625 |
| rs1861410 | T | C | 0.5536 | -0.0146 | 0.0023 | 4.00E-10 | 2 | 58706456 |
| rs963059 | T | C | 0.7967 | -0.0167 | 0.003 | 4.00E-08 | 10 | 110337315 |
| rs13198641 | A | T | 0.6356 | 0.0208 | 0.003 | 4.00E-12 | 6 | 126863842 |
| rs3851294 | A | G | 0.0913 | -0.0201 | 0.0034 | 4.00E-09 | 1 | 205161285 |
| rs41274050 | T | C | 0.0089 | 0.0881 | 0.015 | 4.00E-09 | 10 | 50814012 |
| rs9948087 | A | C | 0.2461 | -0.0151 | 0.0028 | 5.00E-08 | 18 | 268992 |
| rs709822 | C | G | 0.7117 | -0.0212 | 0.0029 | 5.00E-13 | 8 | 11844804 |
| rs2245586 | A | G | 0.3175 | 0.0131 | 0.0022 | 5.00E-09 | 15 | 66586562 |
| rs372826270 | D | I | 0.0011 | 0.3676 | 0.0673 | 5.00E-08 | 14 | 94325616 |
| rs6777217 | A | G | 0.4742 | 0.0101 | 0.0018 | 5.00E-08 | 3 | 36937551 |
| rs12928099 | A | C | 0.2698 | -0.0253 | 0.0032 | 5.00E-15 | 16 | 15056648 |
| rs55707100 | T | C | 0.0279 | 0.0939 | 0.0086 | 5.00E-28 | 15 | 43528519 |
| rs9715911 | A | G | 0.6039 | 0.035 | 0.0027 | 5.00E-38 | 5 | 156967430 |
| rs1801689 | A | C | 0.969 | 0.0801 | 0.0081 | 6.00E-23 | 17 | 66214462 |
| rs3843935 | T | C | 0.3916 | 0.0155 | 0.0024 | 6.00E-11 | 9 | 33787873 |
| rs7316454 | T | G | 0.1789 | 0.019 | 0.0029 | 6.00E-11 | 12 | 45806613 |
| rs1045241 | T | C | 0.2937 | -0.0154 | 0.0024 | 6.00E-11 | 5 | 119393591 |
| rs200911786 | A | C | 0.1523 | -0.0251 | 0.0043 | 6.00E-09 | 4 | 103253686 |
| rs34483103 | D | I | 0.3573 | -0.0756 | 0.0027 | 6.00E-168 | 1 | 63070538 |
| rs116843064 | A | G | 0.0193 | -0.2524 | 0.0102 | 6.00E-134 | 19 | 8364439 |
| rs3173615 | C | G | 0.5296 | -0.0113 | 0.0019 | 7.00E-09 | 7 | 12229791 |
| rs1799777 | D | I | 0.8768 | 0.0265 | 0.0046 | 7.00E-09 | 9 | 104903755 |
| rs1519480 | T | C | 0.5941 | -0.016 | 0.0022 | 7.00E-13 | 11 | 27654165 |
| rs2395655 | A | G | 0.5474 | 0.0147 | 0.0021 | 7.00E-12 | 6 | 36677919 |
| rs34518086 | D | I | 0.5138 | 0.0181 | 0.0028 | 7.00E-11 | 7 | 130757845 |
| rs34831171 | T | C | 0.7997 | 0.0246 | 0.0038 | 7.00E-11 | 3 | 12132155 |
| rs2723509 | A | G | 0.4843 | 0.0163 | 0.0028 | 7.00E-09 | 7 | 17777413 |
| rs2937124 | T | C | 0.3417 | -0.0202 | 0.0031 | 7.00E-11 | 16 | 69828206 |
| rs4969145 | T | C | 0.4022 | 0.023 | 0.0028 | 7.00E-17 | 17 | 78410089 |
| rs34248373 | D | I | 0.5179 | 0.0178 | 0.0027 | 7.00E-11 | 12 | 124019257 |
| rs375519927 | D | I | 0.1456 | 0.0237 | 0.0041 | 7.00E-09 | 13 | 50433390 |
| rs72836561 | T | C | 0.0288 | 0.1636 | 0.0084 | 8.00E-84 | 17 | 43848758 |
| rs11656215 | T | C | 0.4756 | -0.0201 | 0.0028 | 8.00E-13 | 17 | 17549753 |
| rs2820441 | A | C | 0.707 | 0.0256 | 0.0031 | 8.00E-17 | 1 | 219561618 |
| rs9963938 | C | G | 0.4413 | 0.0162 | 0.0028 | 8.00E-09 | 18 | 22330221 |
| rs2836878 | A | G | 0.256 | 0.0123 | 0.0021 | 8.00E-09 | 21 | 39093608 |
| rs1278530 | A | G | 0.5078 | -0.016 | 0.0023 | 8.00E-12 | 1 | 50423583 |
| rs9297994 | A | G | 0.6906 | -0.0227 | 0.003 | 8.00E-14 | 8 | 58479765 |
| rs838133 | A | G | 0.4204 | 0.0231 | 0.0032 | 8.00E-13 | 19 | 48756272 |
| rs1408 | A | G | 0.6123 | 0.0305 | 0.0028 | 8.00E-28 | 4 | 87136201 |
| rs718314 | A | G | 0.7408 | -0.0177 | 0.0023 | 9.00E-15 | 12 | 26300350 |
| rs540730 | T | C | 0.2889 | -0.0212 | 0.003 | 9.00E-13 | 12 | 57413331 |
| rs573022400 | D | I | 0.8932 | 0.0296 | 0.0046 | 9.00E-11 | 6 | 26441498 |
| rs459193 | A | G | 0.2968 | -0.0324 | 0.0029 | 9.00E-29 | 5 | 56510924 |
| **VB12** | | | | | | | | |
| rs602662 | A | G | 0.53 | 49.77 | 6.47 | 3.00E-20 | 19 | 53898797 |
| rs11254363 | A | G | 0.7 | 21.49 | 7.03 | 1.00E-06 | 10 | 17170699 |
| rs526934 | A | G | 0.67 | 27.62 | 8.15 | 2.00E-06 | 11 | 59390069 |
| **VD** | | | | | | | | |
| rs6698680 | G | A | 0.464195 | -0.012 | 0.002 | 8.99E-10 | 1 | 2329661 |
| rs3750296 | C | G | 0.341423 | -0.021 | 0.002 | 2.09E-24 | 1 | 17559656 |
| rs7519574 | A | G | 0.181694 | 0.017 | 0.003 | 2.09E-11 | 1 | 34726552 |
| rs56044892 | T | C | 0.2107 | 0.015 | 0.002 | 2.85E-10 | 1 | 41830086 |
| rs2934744 | A | C | 0.643546 | -0.022 | 0.002 | 3.96E-26 | 1 | 63048045 |
| rs7528419 | G | A | 0.224958 | 0.019 | 0.002 | 2.41E-16 | 1 | 109817192 |
| rs3768013 | A | G | 0.369605 | -0.015 | 0.002 | 1.37E-13 | 1 | 150815411 |
| rs115045402 | A | G | 0.026334 | 0.107 | 0.007 | 3.05E-55 | 1 | 152029548 |
| rs12123821 | T | C | 0.04793 | 0.074 | 0.005 | 2.25E-59 | 1 | 152179152 |
| rs201561609 | T | C | 0.986941 | -0.129 | 0.012 | 6.99E-28 | 1 | 152187902 |
| rs185433896 | A | C | 0.993156 | -0.246 | 0.019 | 1.50E-38 | 1 | 152249021 |
| rs189918701 | G | A | 0.997322 | -0.238 | 0.029 | 2.47E-16 | 1 | 152254152 |
| rs375984409 | G | A | 0.991891 | -0.229 | 0.018 | 3.22E-38 | 1 | 152255772 |
| rs144613541 | G | A | 0.290985 | 0.015 | 0.002 | 6.49E-12 | 1 | 152270875 |
| rs150597413 | T | G | 0.004002 | 0.105 | 0.016 | 6.18E-11 | 1 | 152277622 |
| rs138726443 | A | G | 0.00482 | 0.112 | 0.014 | 8.81E-15 | 1 | 152280023 |
| rs61816761 | A | G | 0.023096 | 0.125 | 0.007 | 8.57E-74 | 1 | 152285861 |
| rs576242124 | A | G | 0.011317 | 0.114 | 0.015 | 3.08E-15 | 1 | 152390763 |
| rs184958517 | T | A | 0.993064 | -0.135 | 0.017 | 5.55E-15 | 1 | 153111312 |
| rs558560635 | G | A | 0.99774 | -0.272 | 0.034 | 5.83E-16 | 1 | 153147997 |
| rs11264360 | A | T | 0.24292 | 0.018 | 0.002 | 3.34E-15 | 1 | 155284586 |
| rs867772 | G | A | 0.681808 | -0.014 | 0.002 | 3.64E-11 | 1 | 220972343 |
| rs10127775 | T | A | 0.604643 | 0.012 | 0.002 | 3.43E-09 | 1 | 230295789 |
| rs12997242 | A | G | 0.437687 | -0.013 | 0.002 | 2.23E-10 | 2 | 21381177 |
| rs11127048 | A | G | 0.616569 | 0.018 | 0.002 | 6.41E-19 | 2 | 27752463 |
| rs6724965 | G | A | 0.171608 | -0.017 | 0.003 | 1.29E-10 | 2 | 101440151 |
| rs7569755 | A | G | 0.292374 | 0.014 | 0.002 | 8.03E-11 | 2 | 118648261 |
| rs1047891 | A | C | 0.316449 | -0.014 | 0.002 | 1.16E-11 | 2 | 211540507 |
| rs2011425 | G | T | 0.07939 | -0.046 | 0.004 | 9.66E-38 | 2 | 234627608 |
| rs7650253 | A | T | 0.689684 | 0.015 | 0.002 | 1.76E-10 | 3 | 49431160 |
| rs1972994 | T | A | 0.64702 | -0.018 | 0.002 | 7.99E-18 | 3 | 85631142 |
| rs6438900 | G | C | 0.260706 | 0.014 | 0.002 | 9.59E-10 | 3 | 125148287 |
| rs6773343 | T | C | 0.720232 | 0.013 | 0.002 | 5.20E-09 | 3 | 141825598 |
| rs78649910 | A | T | 0.110004 | -0.018 | 0.003 | 4.32E-09 | 4 | 3482213 |
| rs7699711 | T | G | 0.454848 | -0.029 | 0.002 | 6.97E-49 | 4 | 69947596 |
| rs529640451 | C | G | 0.997488 | 0.233 | 0.027 | 2.25E-17 | 4 | 72177044 |
| rs528776789 | A | G | 0.993104 | 0.178 | 0.015 | 3.67E-31 | 4 | 72486140 |
| rs113938679 | A | G | 0.005873 | -0.184 | 0.015 | 5.88E-36 | 4 | 72488025 |
| rs564377207 | G | C | 0.995541 | -0.199 | 0.021 | 1.05E-21 | 4 | 72488525 |
| rs186897112 | G | A | 0.998019 | 0.247 | 0.034 | 3.79E-13 | 4 | 72528565 |
| rs557657187 | G | A | 0.99894 | 0.365 | 0.045 | 6.18E-16 | 4 | 72539857 |
| rs145432346 | C | T | 0.825873 | 0.109 | 0.003 | 6.78E-286 | 4 | 72575017 |
| rs705117 | T | C | 0.849453 | -0.034 | 0.003 | 1.71E-36 | 4 | 72608115 |
| rs11723621 | G | A | 0.291123 | -0.187 | 0.002 | 2.90E-1689 | 4 | 72615362 |
| rs560384646 | C | A | 0.023835 | -0.193 | 0.009 | 6.91E-112 | 4 | 72616618 |
| rs200641845 | T | A | 0.545188 | 0.018 | 0.002 | 6.92E-14 | 4 | 72620895 |
| rs565277381 | T | G | 0.998975 | 0.308 | 0.047 | 6.62E-11 | 4 | 72625772 |
| rs3775150 | C | T | 0.262221 | -0.091 | 0.002 | 3.90E-295 | 4 | 72640750 |
| rs222026 | T | A | 0.870776 | -0.052 | 0.003 | 6.98E-68 | 4 | 72643760 |
| rs190688847 | C | T | 0.997836 | 0.291 | 0.033 | 1.02E-18 | 4 | 72705716 |
| rs184291421 | C | T | 0.994155 | 0.17 | 0.015 | 1.25E-28 | 4 | 72752846 |
| rs188838036 | A | G | 0.995469 | 0.179 | 0.018 | 3.07E-24 | 4 | 72783385 |
| rs186881826 | A | T | 0.223069 | 0.046 | 0.002 | 3.64E-77 | 4 | 72785743 |
| rs186441690 | G | A | 0.997189 | -0.267 | 0.03 | 1.96E-18 | 4 | 72820969 |
| rs546541682 | T | G | 0.994255 | -0.157 | 0.018 | 2.06E-18 | 4 | 72864566 |
| rs143106299 | T | A | 0.005747 | -0.169 | 0.015 | 1.50E-28 | 4 | 72920085 |
| rs192785674 | A | G | 0.996588 | 0.169 | 0.026 | 8.14E-11 | 4 | 73505826 |
| rs58073039 | G | A | 0.298368 | -0.014 | 0.002 | 2.16E-11 | 4 | 88287363 |
| rs28364331 | G | A | 0.018845 | 0.061 | 0.007 | 1.31E-17 | 4 | 100201295 |
| rs1229984 | C | T | 0.973099 | -0.047 | 0.006 | 4.85E-13 | 4 | 100239319 |
| rs7718395 | G | C | 0.319522 | 0.013 | 0.002 | 1.67E-09 | 5 | 118652574 |
| rs3822868 | G | A | 0.835029 | 0.022 | 0.003 | 1.41E-15 | 6 | 131934986 |
| rs111529171 | C | G | 0.216376 | -0.015 | 0.002 | 6.24E-11 | 7 | 21571932 |
| rs1011468 | A | G | 0.475748 | -0.014 | 0.002 | 1.35E-12 | 7 | 104613791 |
| rs1858889 | C | A | 0.50084 | 0.013 | 0.002 | 3.85E-11 | 7 | 107117447 |
| rs804280 | A | C | 0.581959 | 0.013 | 0.002 | 4.43E-11 | 8 | 11612698 |
| rs34726834 | T | C | 0.253615 | 0.014 | 0.002 | 6.65E-10 | 8 | 25889606 |
| rs7828742 | G | A | 0.596853 | -0.022 | 0.002 | 3.06E-28 | 8 | 116960729 |
| rs10818769 | G | C | 0.856966 | -0.017 | 0.003 | 3.35E-09 | 9 | 125719923 |
| rs532436 | A | G | 0.184388 | -0.015 | 0.003 | 2.17E-09 | 9 | 136149830 |
| rs10887718 | T | C | 0.527362 | -0.012 | 0.002 | 1.44E-10 | 10 | 82042624 |
| rs538325438 | C | A | 0.998583 | 0.227 | 0.032 | 6.07E-13 | 11 | 13414030 |
| rs373514022 | C | T | 0.998234 | 0.203 | 0.029 | 4.77E-12 | 11 | 13955649 |
| rs571618690 | A | C | 0.998605 | 0.366 | 0.031 | 1.90E-31 | 11 | 13996822 |
| rs191379475 | G | A | 0.98893 | -0.103 | 0.013 | 1.70E-15 | 11 | 14075712 |
| rs561089663 | G | C | 0.998499 | 0.409 | 0.03 | 4.79E-43 | 11 | 14100539 |
| rs10832218 | C | T | 0.19774 | -0.034 | 0.003 | 7.09E-32 | 11 | 14181174 |
| rs117206369 | T | C | 0.998354 | 0.468 | 0.032 | 1.07E-48 | 11 | 14335876 |
| rs567876843 | G | T | 0.995283 | 0.542 | 0.019 | 1.83E-180 | 11 | 14414139 |
| rs148514005 | T | C | 0.005501 | -0.447 | 0.015 | 1.37E-184 | 11 | 14464878 |
| rs571484036 | A | G | 0.998325 | -0.217 | 0.027 | 4.13E-16 | 11 | 14512559 |
| rs577185477 | C | T | 0.01469 | -0.379 | 0.01 | 1.624E-342 | 11 | 14612563 |
| rs554808052 | C | A | 0.998315 | 0.349 | 0.026 | 5.41E-40 | 11 | 14636390 |
| rs10832289 | T | A | 0.410173 | -0.069 | 0.002 | 2.03E-266 | 11 | 14669496 |
| rs187443664 | T | G | 0.98867 | -0.106 | 0.013 | 3.49E-16 | 11 | 14768892 |
| rs188480917 | G | C | 0.010804 | -0.343 | 0.01 | 5.00E-275 | 11 | 14785870 |
| rs534042887 | G | T | 0.996899 | 0.392 | 0.02 | 2.82E-82 | 11 | 14818258 |
| rs532836473 | G | A | 0.998425 | 0.436 | 0.031 | 4.90E-44 | 11 | 14822853 |
| rs201501563 | T | C | 0.122197 | -0.066 | 0.004 | 9.17E-67 | 11 | 14882470 |
| rs117913124 | A | G | 0.027583 | -0.354 | 0.006 | 1.653E-775 | 11 | 14900931 |
| rs117576073 | T | G | 0.012385 | -0.115 | 0.009 | 1.22E-38 | 11 | 14912573 |
| rs150585703 | G | C | 0.996054 | 0.482 | 0.02 | 7.16E-125 | 11 | 14951216 |
| rs574992951 | C | T | 0.991488 | 0.087 | 0.015 | 4.04E-09 | 11 | 16580958 |
| rs567415847 | G | A | 0.997973 | 0.283 | 0.037 | 1.03E-14 | 11 | 16854631 |
| rs523583 | C | A | 0.469219 | 0.012 | 0.002 | 5.58E-10 | 11 | 66070146 |
| rs12803256 | G | A | 0.770616 | 0.1 | 0.002 | 8.599E-407 | 11 | 71132868 |
| rs536006581 | G | G | 0.008644 | -0.174 | 0.014 | 8.87E-35 | 11 | 71135151 |
| rs574615332 | A | C | 0.99727 | -0.287 | 0.026 | 1.38E-28 | 11 | 71144427 |
| rs549940584 | T | C | 0.013335 | 0.183 | 0.01 | 2.31E-72 | 11 | 71222408 |
| rs200454003 | T | C | 0.264587 | -0.087 | 0.003 | 3.68E-256 | 11 | 71228990 |
| rs10793129 | A | G | 0.090078 | 0.024 | 0.003 | 1.64E-12 | 11 | 75459865 |
| rs1149605 | C | T | 0.171453 | 0.019 | 0.003 | 7.34E-14 | 11 | 76485216 |
| rs964184 | C | G | 0.863547 | 0.04 | 0.003 | 5.11E-44 | 11 | 116648917 |
| rs2847500 | A | G | 0.124391 | -0.021 | 0.003 | 7.79E-13 | 11 | 120114421 |
| rs12317268 | G | A | 0.151589 | -0.019 | 0.003 | 9.15E-12 | 12 | 21352541 |
| rs9668081 | T | C | 0.47058 | 0.012 | 0.002 | 5.38E-09 | 12 | 38602911 |
| rs61937878 | T | C | 0.006418 | 0.119 | 0.012 | 4.43E-22 | 12 | 96371731 |
| rs10859995 | C | T | 0.581322 | -0.039 | 0.002 | 7.03E-89 | 12 | 96375682 |
| rs8018720 | C | G | 0.820235 | -0.032 | 0.003 | 4.04E-36 | 14 | 39556185 |
| rs261291 | C | T | 0.35583 | -0.022 | 0.002 | 2.89E-28 | 15 | 58680178 |
| rs1800588 | T | C | 0.21461 | -0.03 | 0.002 | 2.65E-36 | 15 | 58723675 |
| rs17765311 | C | A | 0.344611 | -0.015 | 0.002 | 1.35E-13 | 15 | 63789952 |
| rs62007299 | A | G | 0.709377 | -0.014 | 0.002 | 1.69E-11 | 15 | 77711719 |
| rs8063706 | T | A | 0.272828 | 0.013 | 0.002 | 3.64E-09 | 16 | 11909552 |
| rs77924615 | A | G | 0.197773 | -0.016 | 0.002 | 1.46E-10 | 16 | 20392332 |
| rs71383766 | T | C | 0.419574 | 0.013 | 0.002 | 1.15E-09 | 16 | 30930233 |
| rs1800775 | A | C | 0.486317 | -0.017 | 0.002 | 1.56E-17 | 16 | 56995236 |
| rs2909218 | T | C | 0.792744 | 0.017 | 0.002 | 2.81E-12 | 17 | 66464546 |
| rs8091117 | A | C | 0.065396 | -0.024 | 0.004 | 1.03E-09 | 18 | 28919794 |
| rs2037511 | A | G | 0.165391 | 0.016 | 0.003 | 9.29E-10 | 18 | 61366207 |
| rs57631352 | G | A | 0.297255 | -0.013 | 0.002 | 1.48E-09 | 19 | 4338173 |
| rs73015021 | G | A | 0.121031 | 0.023 | 0.003 | 1.15E-14 | 19 | 11192915 |
| rs10500209 | C | T | 0.282129 | -0.013 | 0.002 | 6.18E-10 | 19 | 11979164 |
| rs58542926 | T | C | 0.075847 | 0.032 | 0.004 | 8.57E-19 | 19 | 19379549 |
| rs3814995 | T | C | 0.31224 | -0.015 | 0.002 | 2.83E-12 | 19 | 36342212 |
| rs1065853 | T | G | 0.082083 | 0.027 | 0.004 | 8.32E-14 | 19 | 45413233 |
| rs157595 | G | A | 0.614127 | -0.016 | 0.002 | 2.95E-14 | 19 | 45425460 |
| rs112285002 | T | C | 0.159529 | 0.06 | 0.003 | 1.77E-110 | 19 | 48374320 |
| rs62130059 | C | A | 0.335823 | -0.027 | 0.002 | 9.25E-34 | 19 | 48461240 |
| rs10426 | A | G | 0.213371 | 0.025 | 0.002 | 3.31E-26 | 19 | 51517798 |
| rs8103262 | C | T | 0.305004 | 0.013 | 0.002 | 3.18E-09 | 19 | 53065814 |
| rs6123359 | G | A | 0.105192 | 0.032 | 0.003 | 7.74E-24 | 20 | 52714706 |
| rs6127099 | T | A | 0.279034 | -0.037 | 0.002 | 9.30E-62 | 20 | 52731402 |
| rs2585442 | G | C | 0.246306 | 0.034 | 0.002 | 6.87E-49 | 20 | 52737123 |
| rs2762942 | A | G | 0.942083 | 0.053 | 0.004 | 7.99E-35 | 20 | 52788925 |
| rs2229742 | C | G | 0.103928 | -0.026 | 0.003 | 7.13E-16 | 21 | 16339172 |
| rs2074735 | C | G | 0.064197 | 0.027 | 0.004 | 6.55E-12 | 22 | 31535872 |
| rs960596 | T | C | 0.339588 | 0.012 | 0.002 | 2.23E-09 | 22 | 41393520 |
| **WC** | | | | | | | | |
| rs16957304 | A | G | 0.9298 | 0.0599 | 0.009184 | 8.00E-11 | 16 | 67301066 |
| rs12207675 | T | C | 0.8857 | -0.0333 | 0.005357 | 7.00E-10 | 6 | 75528025 |
| rs1776897 | T | G | 0.8414 | -0.0536 | 0.006378 | 7.00E-17 | 6 | 34227234 |
| rs4141278 | T | C | 0.7935 | -0.0315 | 0.004337 | 1.00E-12 | 7 | 25817905 |
| rs6470765 | A | C | 0.777 | 0.024 | 0.003061 | 3.00E-12 | 8 | 129724451 |
| rs7166081 | A | G | 0.7599 | 0.0266 | 0.004337 | 2.00E-10 | 15 | 67199963 |
| rs7830933 | A | G | 0.7571 | 0.0332 | 0.005102 | 1.00E-10 | 8 | 23745811 |
| rs3791679 | A | G | 0.7555 | 0.0354 | 0.004337 | 5.00E-16 | 2 | 55869757 |
| rs9408815 | C | G | 0.75 | 0.023 | 0.003061 | 1.00E-11 | 9 | 106128240 |
| rs7536458 | T | G | 0.748 | 0.0333 | 0.004337 | 2.00E-15 | 1 | 118321979 |
| rs2197271 | C | G | 0.7215 | -0.0248 | 0.004082 | 1.00E-09 | 4 | 81239194 |
| rs12679556 | T | G | 0.7067 | -0.0221 | 0.004082 | 1.00E-08 | 8 | 71601993 |
| rs12991495 | T | C | 0.6973 | 0.0253 | 0.004082 | 3.00E-10 | 2 | 25263901 |
| rs12048049 | C | G | 0.6885 | -0.0239 | 0.003571 | 1.00E-10 | 1 | 218423955 |
| rs6772896 | T | C | 0.6799 | 0.026 | 0.003571 | 1.00E-12 | 3 | 134484505 |
| rs2638953 | C | G | 0.6764 | 0.0258 | 0.003571 | 2.00E-12 | 12 | 28381482 |
| rs882367 | T | C | 0.6556 | -0.0227 | 0.003571 | 4.00E-10 | 17 | 61417213 |
| rs9860730 | A | G | 0.6495 | 0.0344 | 0.004847 | 5.00E-13 | 3 | 64715470 |
| rs12317176 | T | C | 0.649 | 0.0211 | 0.003571 | 4.00E-09 | 12 | 123920171 |
| rs17451107 | T | C | 0.6145 | 0.0263 | 0.003827 | 6.00E-13 | 3 | 157079820 |
| rs10923712 | A | G | 0.6025 | 0.0366 | 0.003316 | 2.00E-25 | 1 | 118962811 |
| rs11205277 | A | G | 0.596 | -0.0311 | 0.004082 | 3.00E-14 | 1 | 149920979 |
| rs10195252 | T | C | 0.5727 | 0.038 | 0.005102 | 9.00E-14 | 2 | 164656581 |
| rs1344674 | A | G | 0.5451 | -0.0268 | 0.003316 | 8.00E-15 | 3 | 141406344 |
| rs4542783 | T | C | 0.543 | 0.0231 | 0.004082 | 1.00E-08 | 19 | 8577276 |
| rs6743226 | C | T | 0.543 | 0.018 | 0.003061 | 7.00E-12 | 2 | 241297557 |
| rs2214442 | A | G | 0.5223 | -0.0234 | 0.004337 | 2.00E-08 | 7 | 20353164 |
| rs2745353 | T | C | 0.5202 | 0.0215 | 0.003316 | 6.00E-10 | 6 | 127131790 |
| rs7773004 | A | G | 0.4885 | 0.0225 | 0.003316 | 5.00E-11 | 6 | 26267527 |
| rs7697556 | T | C | 0.486 | 0.024 | 0.003061 | 5.00E-19 | 4 | 72649596 |
| rs12493901 | A | G | 0.4712 | -0.0239 | 0.003571 | 1.00E-11 | 3 | 172204265 |
| rs2160077 | A | G | 0.4322 | -0.0196 | 0.003316 | 2.00E-08 | 14 | 91962066 |
| rs7162542 | C | G | 0.4275 | -0.0342 | 0.003316 | 2.00E-23 | 15 | 83845538 |
| rs849140 | T | C | 0.4168 | 0.0253 | 0.003571 | 3.00E-12 | 7 | 28144083 |
| rs6012558 | A | G | 0.412 | 0.016 | 0.003061 | 2.00E-09 | 20 | 48914749 |
| rs272869 | A | G | 0.4005 | -0.0194 | 0.003316 | 2.00E-08 | 5 | 132342304 |
| rs1884897 | A | G | 0.3739 | 0.0229 | 0.003571 | 7.00E-10 | 20 | 6632185 |
| rs4886782 | A | G | 0.3642 | -0.0228 | 0.003827 | 1.00E-09 | 15 | 73936469 |
| rs2071449 | A | C | 0.3614 | 0.0292 | 0.003827 | 4.00E-14 | 12 | 54034227 |
| rs6556301 | T | G | 0.3605 | 0.0314 | 0.004082 | 4.00E-14 | 5 | 177100576 |
| rs12608504 | A | G | 0.3589 | 0.0208 | 0.003571 | 3.00E-08 | 19 | 18278325 |
| rs2274432 | A | G | 0.3486 | 0.0283 | 0.003827 | 3.00E-14 | 1 | 184051811 |
| rs10269774 | A | G | 0.333 | 0.019 | 0.003061 | 3.00E-10 | 7 | 92624658 |
| rs6570507 | A | G | 0.3186 | -0.0256 | 0.003827 | 2.00E-10 | 6 | 142358435 |
| rs395962 | T | G | 0.3169 | 0.0281 | 0.003571 | 3.00E-14 | 6 | 104949543 |
| rs9435732 | T | C | 0.2658 | -0.0318 | 0.003827 | 3.00E-16 | 1 | 16981663 |
| rs4239436 | A | G | 0.2027 | -0.0418 | 0.004337 | 2.00E-22 | 18 | 23151966 |
| rs7684221 | A | G | 0.1931 | -0.0266 | 0.004592 | 9.00E-09 | 4 | 17955731 |
| rs17396340 | A | G | 0.132 | 0.027 | 0.004082 | 1.00E-11 | 1 | 10226118 |
| rs4378999 | T | A | 0.119 | 0.03 | 0.005102 | 8.00E-11 | 3 | 51171215 |

EA: Effect allele, OA: Other allele, EAF: Effect allele frequency

**Supplementary Table S4. Other Mendelian randomization estimates between extrinsic exposures and prostate cancer risk.**

| **Exposures** | **Method** | **PRACTICAL consortium** | | | | | **UK Biobank** | | | | |
| --- | --- | --- | --- | --- | --- | --- | --- | --- | --- | --- | --- |
|  |  | **SNPs** | **OR** | **LCI** | **UCI** | ***P*** | **SNPs** | **OR** | **LCI** | **UCI** | ***P*** |
| ALA | MR Egger | 3 | 0.489 | 0.011 | 22.412 | 0.714 | 3 | 0.489 | 0.011 | 22.412 | 0.714 |
|  | Weighted median | 3 | 0.655 | 0.167 | 2.574 | 0.545 | 3 | 0.655 | 0.162 | 2.644 | 0.553 |
|  | Simple mode | 3 | 1.089 | 0.107 | 11.039 | 0.942 | 3 | 1.089 | 0.120 | 9.919 | 0.940 |
|  | Weighted mode | 3 | 0.595 | 0.109 | 3.241 | 0.549 | 3 | 0.595 | 0.103 | 3.433 | 0.562 |
| Alcohol | MR Egger | 11 | 1.505 | 0.990 | 2.288 | 0.347 | 11 | 0.489 | 0.011 | 22.412 | 0.972 |
|  | Weighted median | 11 | 1.056 | 0.929 | 1.200 | 0.468 | 11 | 0.655 | 2.653 | 0.162 | 0.777 |
|  | Simple mode | 11 | 0.992 | 0.785 | 1.254 | 0.955 | 11 | 1.089 | 0.104 | 11.387 | 0.484 |
|  | Weighted mode | 11 | 0.966 | 0.771 | 1.211 | 0.314 | 11 | 0.595 | 3.358 | 0.106 | 0.690 |
| Aspirin | MR Egger | 9 | 0.869 | 0.600 | 1.257 | 0.455 | 10 | 0.984 | 0.959 | 1.009 | 0.204 |
|  | Weighted median | 9 | 1.037 | 0.870 | 1.236 | 0.684 | 10 | 1.012 | 1.003 | 1.020 | 0.008 |
|  | Simple mode | 9 | 0.993 | 0.750 | 1.314 | 0.960 | 10 | 1.017 | 1.001 | 1.033 | 0.035 |
|  | Weighted mode | 9 | 1.000 | 0.783 | 1.277 | 0.999 | 10 | 1.015 | 1.001 | 1.029 | 0.041 |
| BMI | MR Egger | 186 | 1.313 | 0.897 | 1.920 | 0.146 | 183 | 0.992 | 1.008 | 0.977 | 0.248 |
|  | Weighted median | 186 | 1.008 | 0.881 | 1.153 | 0.979 | 183 | 0.991 | 0.998 | 0.984 | 0.006 |
|  | Simple mode | 186 | 1.082 | 0.740 | 1.582 | 0.808 | 183 | 0.982 | 0.964 | 1.001 | 0.143 |
|  | Weighted mode | 186 | 0.984 | 0.696 | 1.392 | 0.849 | 183 | 0.987 | 0.974 | 1.001 | 0.092 |
| BPH | MR Egger | 7 | 1.018 | 0.914 | 1.133 | 0.748 | 5 | 1.003 | 0.996 | 1.010 | 0.427 |
|  | Weighted median | 7 | 1.031 | 0.976 | 1.088 | 0.273 | 5 | 1.000 | 0.997 | 1.003 | 0.957 |
|  | Simple mode | 7 | 1.064 | 0.972 | 1.163 | 0.179 | 5 | 0.997 | 0.991 | 1.003 | 0.333 |
|  | Weighted mode | 7 | 1.056 | 0.965 | 1.154 | 0.234 | 5 | 1.002 | 0.997 | 1.006 | 0.441 |
| Coffee | MR Egger | 13 | 1.543 | 0.539 | 4.424 | 0.607 | 13 | 0.997 | 0.967 | 1.029 | 0.923 |
|  | Weighted median | 13 | 0.954 | 0.564 | 1.613 | 0.791 | 13 | 0.993 | 0.973 | 1.013 | 0.427 |
|  | Simple mode | 13 | 0.192 | 0.038 | 0.964 | 0.080 | 13 | 0.993 | 0.959 | 1.029 | 0.072 |
|  | Weighted mode | 13 | 1.218 | 0.744 | 1.995 | 0.593 | 13 | 0.993 | 0.974 | 1.013 | 0.517 |
| DHA | Wald ratio | 1 | 0.806 | 0.661 | 0.984 | 0.034 | 1 | 1.008 | 0.998 | 1.017 | 0.117 |
| FG | MR Egger | 32 | 1.216 | 0.781 | 1.891 | 0.387 | 32 | 0.996 | 0.980 | 1.013 | 0.662 |
|  | Weighted median | 32 | 0.988 | 0.782 | 1.250 | 0.922 | 32 | 0.994 | 0.983 | 1.004 | 0.248 |
|  | Simple mode | 32 | 1.239 | 0.764 | 2.008 | 0.385 | 32 | 1.008 | 0.987 | 1.028 | 0.465 |
|  | Weighted mode | 32 | 1.023 | 0.825 | 1.268 | 0.839 | 32 | 0.996 | 0.985 | 1.007 | 0.452 |
| Folate | Wald | 1 | 0.991 | 0.971 | 1.013 | 0.431 | 1 | 0.999 | 0.998 | 1.000 | 0.223 |
| HDL | MR Egger | 130 | 0.949 | 0.842 | 1.070 | 0.391 | 128 | 1.002 | 0.996 | 1.009 | 0.485 |
|  | Weighted median | 130 | 0.975 | 0.882 | 1.077 | 0.618 | 128 | 1.003 | 0.998 | 1.008 | 0.265 |
|  | Simple mode | 130 | 0.976 | 0.805 | 1.183 | 0.804 | 128 | 1.008 | 0.998 | 1.019 | 0.118 |
|  | Weighted mode | 130 | 0.976 | 0.903 | 1.055 | 0.536 | 128 | 1.001 | 0.995 | 1.008 | 0.749 |
| Height | MR Egger | 161 | 0.999 | 0.827 | 1.206 | 0.990 | 161 | 1.002 | 0.993 | 1.011 | 0.655 |
|  | Weighted median | 161 | 1.019 | 0.935 | 1.111 | 0.670 | 161 | 1.000 | 0.996 | 1.004 | 0.935 |
|  | Simple mode | 161 | 1.108 | 0.855 | 1.435 | 0.438 | 161 | 0.995 | 0.981 | 1.009 | 0.480 |
|  | Weighted mode | 161 | 1.100 | 0.872 | 1.388 | 0.422 | 161 | 0.997 | 0.984 | 1.010 | 0.663 |
| Hypertension | MR Egger | 33 | 1.164 | 0.722 | 1.878 | 0.533 | 32 | 0.994 | 0.977 | 1.011 | 0.662 |
|  | Weighted median | 33 | 1.031 | 0.910 | 1.168 | 0.631 | 32 | 1.000 | 0.995 | 1.006 | 0.274 |
|  | Simple mode | 33 | 0.959 | 0.734 | 1.254 | 0.760 | 32 | 1.000 | 0.987 | 1.013 | 0.454 |
|  | Weighted mode | 33 | 1.000 | 1.299 | 0.769 | 0.998 | 32 | 1.002 | 0.991 | 1.013 | 0.454 |
| IGF1 | MR Egger | 4 | 1.003 | 0.994 | 1.012 | 0.493 | 4 | 1.000 | 1.000 | 1.001 | 0.316 |
|  | Weighted median | 4 | 1.000 | 0.997 | 1.004 | 0.862 | 4 | 1.000 | 1.000 | 1.000 | 0.328 |
|  | Simple mode | 4 | 0.999 | 0.994 | 1.004 | 0.797 | 4 | 1.000 | 1.000 | 1.000 | 0.484 |
|  | Weighted mode | 4 | 1.001 | 0.997 | 1.005 | 0.620 | 4 | 1.000 | 1.000 | 1.000 | 0.256 |
| IGFBP3 | MR Egger | 4 | 1.000 | 1.000 | 1.001 | 0.220 | 4 | 1.000 | 1.000 | 1.000 | 0.938 |
|  | Weighted median | 4 | 1.000 | 1.000 | 1.000 | 0.0002 | 4 | 1.000 | 1.000 | 1.000 | 0.752 |
|  | Simple mode | 4 | 1.000 | 1.000 | 1.000 | 0.387 | 4 | 1.000 | 1.000 | 1.000 | 0.444 |
|  | Weighted mode | 4 | 1.000 | 1.000 | 1.000 | 0.036 | 4 | 1.000 | 1.000 | 1.000 | 0.830 |
| IL-6 | MR Egger | 19 | 0.954 | 0.820 | 1.111 | 0.547 | 17 | 0.999 | 0.994 | 1.005 | 0.821 |
|  | Weighted median | 19 | 0.938 | 0.853 | 1.030 | 0.181 | 17 | 0.999 | 0.995 | 1.003 | 0.703 |
|  | Simple mode | 19 | 0.937 | 0.806 | 1.090 | 0.401 | 17 | 1.004 | 0.996 | 1.013 | 0.297 |
|  | Weighted mode | 19 | 0.94 | 0.824 | 1.072 | 0.355 | 17 | 0.998 | 0.990 | 1.005 | 0.545 |
| LDL | MR Egger | 100 | 0.991 | 0.866 | 1.135 | 0.883 | 101 | 1.000 | 0.996 | 1.005 | 0.903 |
|  | Weighted median | 100 | 0.961 | 0.864 | 1.070 | 0.468 | 101 | 1.005 | 1.000 | 1.011 | 0.045 |
|  | Simple mode | 100 | 0.919 | 0.738 | 1.145 | 0.303 | 101 | 1.009 | 0.997 | 1.021 | 0.135 |
|  | Weighted mode | 100 | 0.984 | 0.907 | 1.067 | 0.657 | 101 | 1.004 | 0.999 | 1.009 | 0.083 |
| Metabolic syndrome | MR Egger | 76 | 1.017 | 0.900 | 1.148 | 0.790 | 68 | 1.002 | 0.996 | 1.007 | 0.594 |
|  | Weighted median | 76 | 0.966 | 0.896 | 1.041 | 0.364 | 68 | 1.001 | 0.997 | 1.004 | 0.666 |
|  | Simple mode | 76 | 1.191 | 0.985 | 1.441 | 0.071 | 68 | 0.994 | 0.987 | 1.000 | 0.066 |
|  | Weighted mode | 76 | 0.981 | 0.898 | 1.072 | 0.673 | 68 | 1.001 | 0.997 | 1.004 | 0.714 |
| MS | MR Egger | 183 | 1.006 | 0.847 | 1.196 | 0.943 | 172 | 1.000 | 0.991 | 1.009 | 0.966 |
|  | Weighted median | 183 | 1.001 | 0.934 | 1.074 | 0.970 | 172 | 0.999 | 0.996 | 1.003 | 0.746 |
|  | Simple mode | 183 | 0.977 | 0.810 | 1.179 | 0.811 | 172 | 1.000 | 0.990 | 1.010 | 0.97 |
|  | Weighted mode | 183 | 0.985 | 0.853 | 1.137 | 0.837 | 172 | 0.999 | 0.992 | 1.006 | 0.705 |
| PD | MR Egger | 98 | 1.024 | 0.959 | 1.095 | 0.471 | 98 | 1.000 | 0.997 | 1.003 | 0.972 |
|  | Weighted median | 98 | 1.001 | 0.960 | 1.043 | 0.826 | 98 | 1.000 | 0.998 | 1.002 | 0.746 |
|  | Simple mode | 98 | 0.994 | 0.920 | 1.073 | 0.801 | 98 | 0.999 | 0.996 | 1.003 | 0.895 |
|  | Weighted mode | 98 | 1.001 | 0.954 | 1.050 | 0.793 | 98 | 1.000 | 0.998 | 1.002 | 0.814 |
| Periodontitis | MR Egger | 9 | 0.940 | 0.879 | 1.006 | 0.871 | 9 | 0.994 | 0.973 | 1.017 | 0.62 |
|  | Weighted median | 9 | 0.956 | 0.887 | 1.030 | 0.239 | 9 | 1.001 | 0.998 | 1.005 | 0.533 |
|  | Simple mode | 9 | 0.916 | 0.786 | 1.068 | 0.263 | 9 | 1.001 | 0.995 | 1.007 | 0.723 |
|  | Weighted mode | 9 | 0.965 | 0.852 | 1.092 | 0.568 | 9 | 1.001 | 0.996 | 1.006 | 0.696 |
| Schizophrenia | MR Egger | 134 | 0.839 | 0.570 | 1.235 | 0.374 | 111 | 1.003 | 0.983 | 1.024 | 0.752 |
|  | Weighted median | 134 | 0.956 | 0.857 | 1.067 | 0.421 | 111 | 1.000 | 0.995 | 1.005 | 0.999 |
|  | Simple mode | 134 | 1.125 | 0.830 | 1.524 | 0.448 | 111 | 0.992 | 0.977 | 1.008 | 0.320 |
|  | Weighted mode | 134 | 1.096 | 0.821 | 1.464 | 0.533 | 111 | 0.997 | 0.983 | 1.011 | 0.670 |
| SHBG | MR Egger | 12 | 0.992 | 0.519 | 1.897 | 0.982 | 10 | 0.990 | 0.959 | 1.023 | 0.549 |
|  | Weighted median | 12 | 0.908 | 0.742 | 1.112 | 0.350 | 10 | 0.994 | 0.983 | 1.004 | 0.25 |
|  | Simple mode | 12 | 0.795 | 0.510 | 1.240 | 0.312 | 10 | 0.995 | 0.972 | 1.018 | 0.662 |
|  | Weighted mode | 12 | 0.885 | 0.709 | 1.104 | 0.278 | 10 | 0.993 | 0.981 | 1.005 | 0.271 |
| SLE | MR Egger | 66 | 0.960 | 0.934 | 0.987 | 0.003 | 63 | 0.998 | 0.997 | 0.999 | 0.002 |
|  | Weighted median | 66 | 1.011 | 0.990 | 1.031 | 0.305 | 63 | 0.999 | 0.998 | 1.000 | 0.021 |
|  | Simple mode | 66 | 1.030 | 0.980 | 1.083 | 0.249 | 63 | 1.000 | 1.002 | 0.998 | 0.898 |
|  | Weighted mode | 66 | 1.031 | 0.980 | 1.085 | 0.233 | 63 | 0.998 | 0.997 | 0.999 | 0.000 |
| Smoking | MR Egger | 214 | 0.761 | 0.410 | 1.413 | 0.387 | 213 | 1.005 | 0.979 | 1.032 | 0.704 |
|  | Weighted median | 214 | 0.922 | 0.774 | 1.098 | 0.362 | 213 | 1.001 | 0.993 | 1.010 | 0.772 |
|  | Simple mode | 214 | 0.842 | 0.451 | 1.572 | 0.589 | 213 | 0.983 | 0.955 | 1.012 | 0.246 |
|  | Weighted mode | 214 | 0.773 | 0.405 | 1.476 | 0.436 | 213 | 0.994 | 0.971 | 1.019 | 0.651 |
| T2DM | MR Egger | 141 | 1.004 | 0.872 | 1.156 | 0.956 | 147 | 1.001 | 0.996 | 1.006 | 0.748 |
|  | Weighted median | 141 | 0.998 | 0.942 | 1.057 | 0.934 | 147 | 1.002 | 0.999 | 1.005 | 0.120 |
|  | Simple mode | 141 | 0.924 | 0.820 | 1.040 | 0.189 | 147 | 1.003 | 0.997 | 1.009 | 0.331 |
|  | Weighted mode | 141 | 0.981 | 0.921 | 1.046 | 0.560 | 147 | 1.002 | 0.999 | 1.005 | 0.151 |
| Testosterone | MR Egger | 9 | 1.044 | 0.620 | 1.759 | 0.871 | 7 | 1.003 | 0.986 | 1.021 | 0.704 |
|  | Weighted median | 9 | 0.956 | 0.886 | 1.032 | 0.250 | 7 | 1.000 | 0.991 | 1.008 | 0.908 |
|  | Simple mode | 9 | 0.916 | 0.788 | 1.065 | 0.253 | 7 | 1.001 | 0.988 | 1.014 | 0.925 |
|  | Weighted mode | 9 | 0.965 | 0.853 | 1.090 | 0.563 | 7 | 1.000 | 0.988 | 1.012 | 0.998 |
| TG | MR Egger | 124 | 0.964 | 0.843 | 1.103 | 0.595 | 121 | 0.999 | 0.994 | 1.005 | 0.843 |
|  | Weighted median | 124 | 1.017 | 0.905 | 1.143 | 0.777 | 121 | 1.002 | 0.997 | 1.008 | 0.445 |
|  | Simple mode | 124 | 1.051 | 0.827 | 1.335 | 0.687 | 121 | 1.006 | 0.994 | 1.019 | 0.314 |
|  | Weighted mode | 124 | 0.952 | 0.864 | 1.048 | 0.314 | 121 | 1.002 | 0.997 | 1.007 | 0.422 |
| VB12 | MR Egger | 3 | 1.001 | 0.999 | 1.003 | 0.393 | 3 | 1.000 | 1.000 | 1.000 | 0.430 |
|  | Weighted median | 3 | 1.000 | 1.000 | 1.001 | 0.070 | 3 | 1.000 | 1.000 | 1.000 | 0.411 |
|  | Simple mode | 3 | 1.000 | 1.000 | 1.001 | 0.359 | 3 | 1.000 | 1.000 | 1.000 | 0.632 |
|  | Weighted mode | 3 | 1.000 | 1.000 | 1.001 | 0.206 | 3 | 1.000 | 1.000 | 1.000 | 0.292 |
| VD | MR Egger | 106 | 1.014 | 0.931 | 1.104 | 0.752 | 85 | 0.997 | 0.992 | 1.002 | 0.254 |
|  | Weighted median | 106 | 1.024 | 0.932 | 1.126 | 0.618 | 85 | 0.998 | 0.993 | 1.004 | 0.571 |
|  | Simple mode | 106 | 1.003 | 0.826 | 1.219 | 0.973 | 85 | 0.995 | 0.982 | 1.008 | 0.471 |
|  | Weighted mode | 106 | 1.003 | 0.921 | 1.093 | 0.939 | 85 | 0.997 | 0.992 | 1.001 | 0.159 |
| WC | MR Egger | 49 | 0.859 | 0.449 | 1.645 | 0.647 | 48 | 1.038 | 1.004 | 1.073 | 0.026 |
|  | Weighted median | 49 | 0.934 | 0.780 | 1.119 | 0.459 | 48 | 0.999 | 0.990 | 1.008 | 0.856 |
|  | Simple mode | 49 | 0.944 | 0.643 | 1.387 | 0.770 | 48 | 0.998 | 0.978 | 1.019 | 0.869 |
|  | Weighted mode | 49 | 0.944 | 0.657 | 1.356 | 0.757 | 48 | 0.999 | 0.983 | 1.016 | 0.917 |

ALA: alpha-linolenic acid measurement, BPH: Benign Prostatic Hyperplasia, BMI: Body mass index, DHA: Docosahexaenoic acid, HDL: High density lipoprotein, FG: Fasting blood glucose, LCI = Lower 95% confidence interval, LDL: Low density lipoprotein, MS: Multiple Sclerosis, OR = odds ratio, PD: Parkinson’s disease, SHBG: Sex hormone binding globulin, TG: Triglycerides, SLE: Systemic lupus erythematosus, UCI = Upper 95% confidence interval, WC: Waist circumference, VB12: Vitamin B12, VD: Vitamin D.

**Supplementary Figure S1. Forest plots of MR estimate concerning relationship between exposures and prostate cancer. a. IGFBP-3 levels and prostate cancer in PRACTICAL consortium, b. BMI and prostate cancer in UK Biobank, c. SLE and prostate cancer in UK Biobank.** The causal effect of exposure on outcome is estimated using each SNP singly using the Wald ratio.

a.


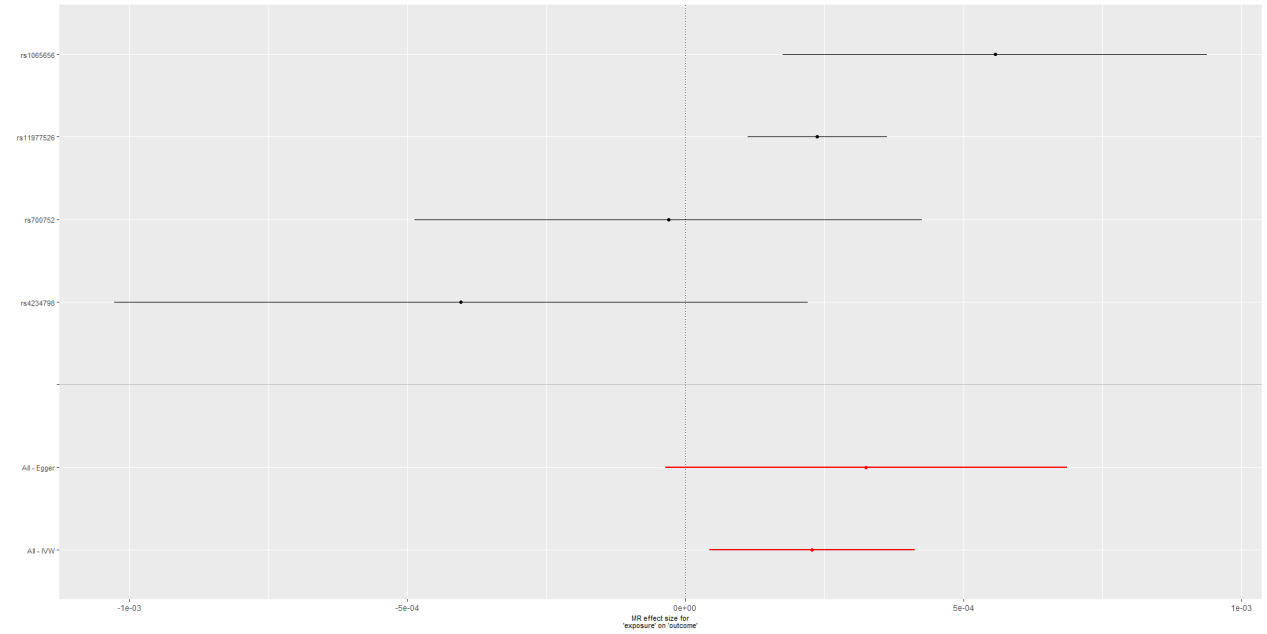


b.


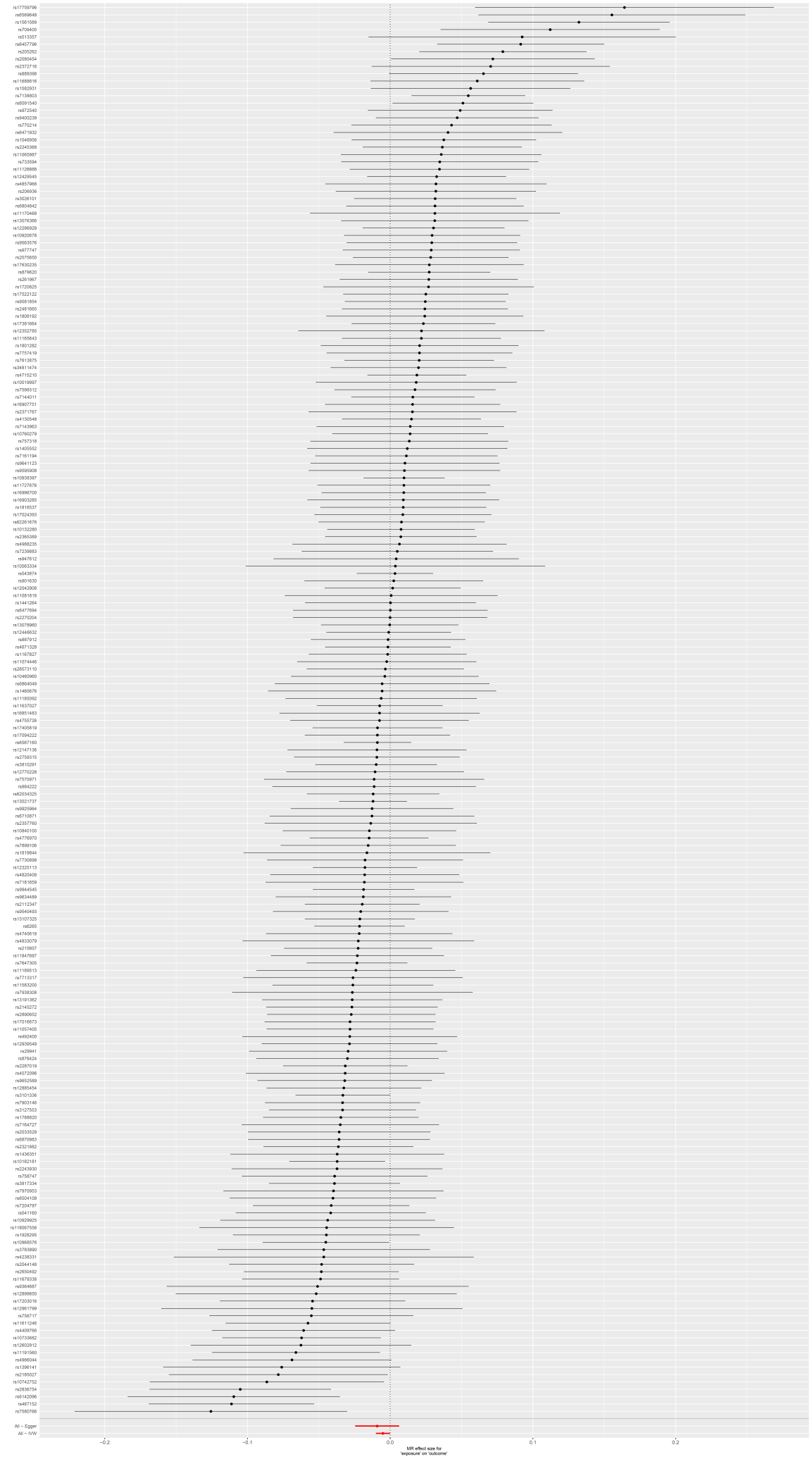


**c.**


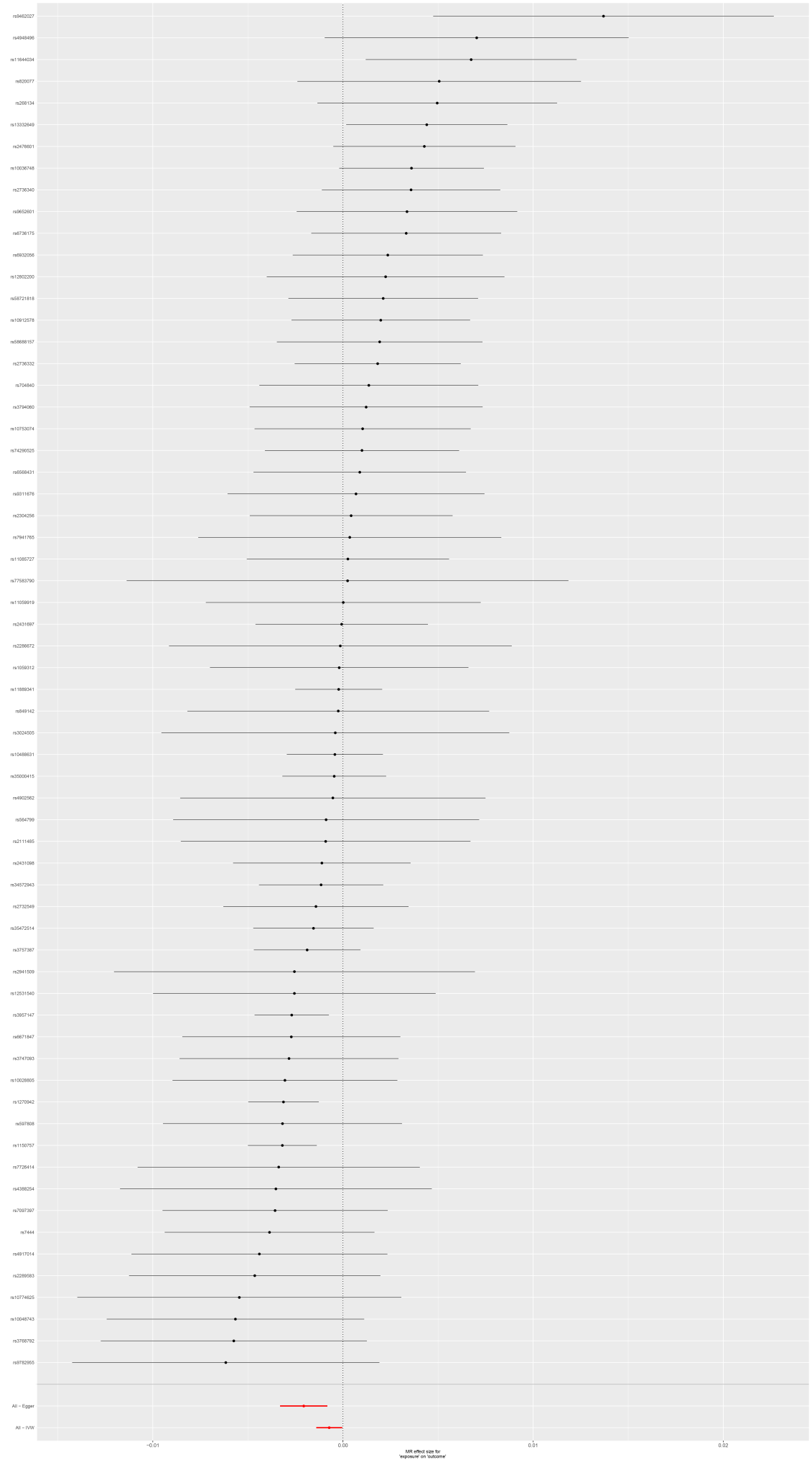


**Supplementary Figure S2. Funnel plots of MR estimate concerning relationship between exposures and prostate cancer. a. BMI and prostate cancer in UK Biobank, b. SLE and prostate cancer in UK Biobank.** The causal effect of exposure on outcome is estimated using each SNP singly using the Wald ratio.

a.


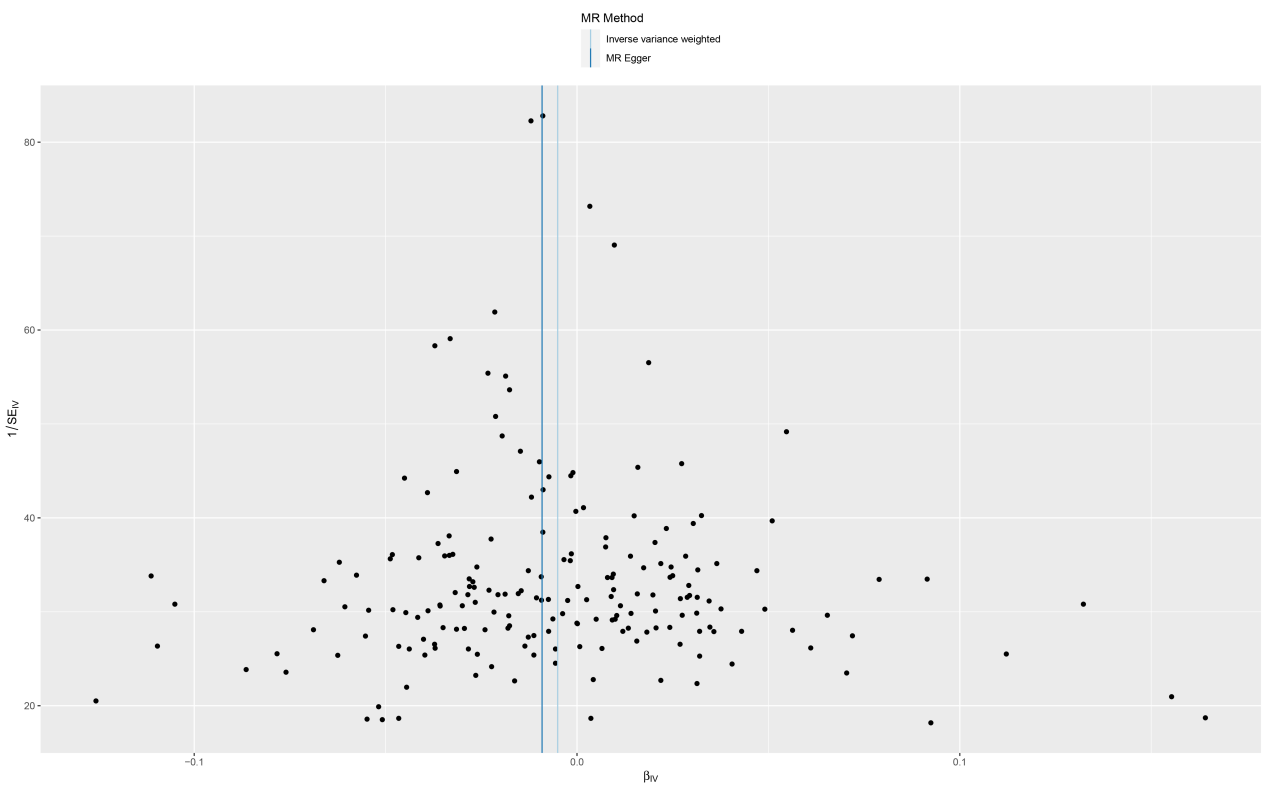


b.


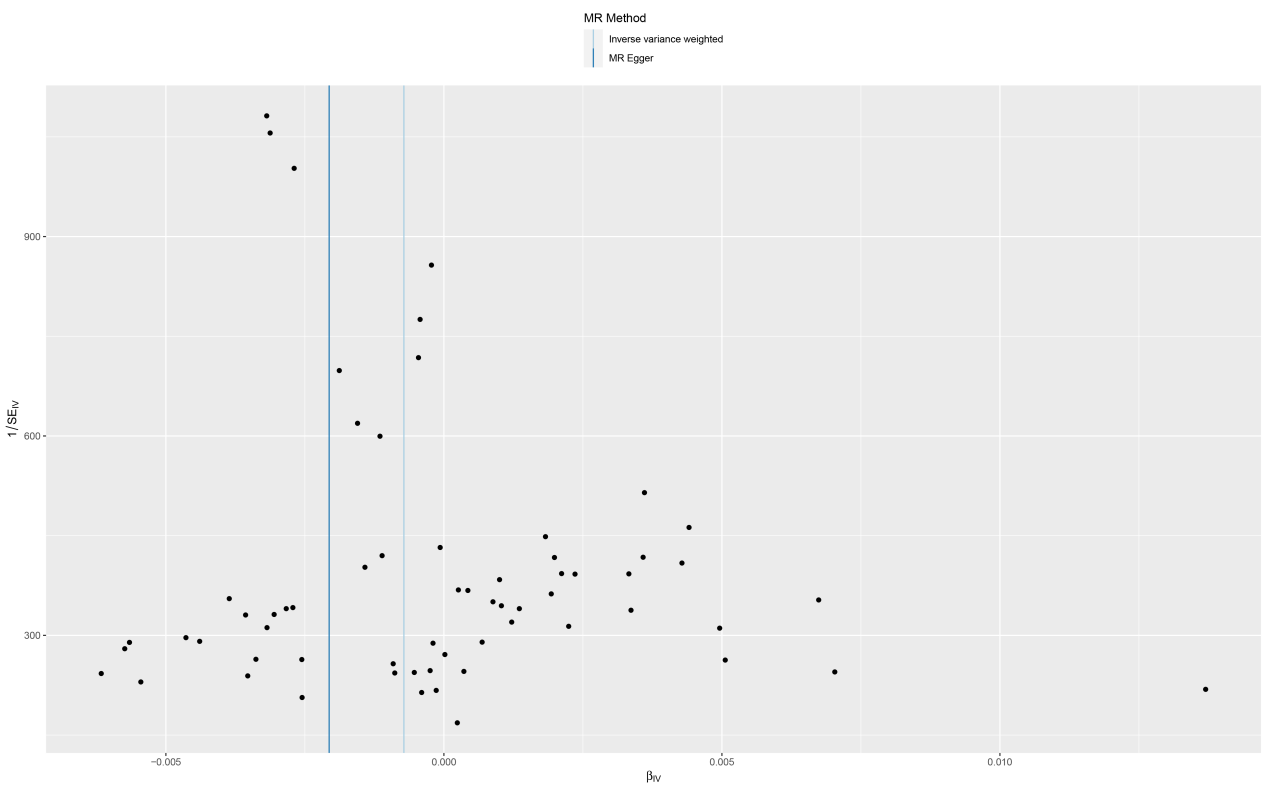


**Supplementary Figure S3. Scatter plots** **of MR estimate concerning relationship between exposures and prostate cancer.** **a. IGFBP-3 levels and prostate cancer in PRACTICAL consortium, b. BMI and prostate cancer in UK Biobank, c. SLE and prostate cancer in UK Biobank.** SNP effects on the outcome are plotted against SNP effects on the exposure, and the slope of the line represents the causal association.

a.


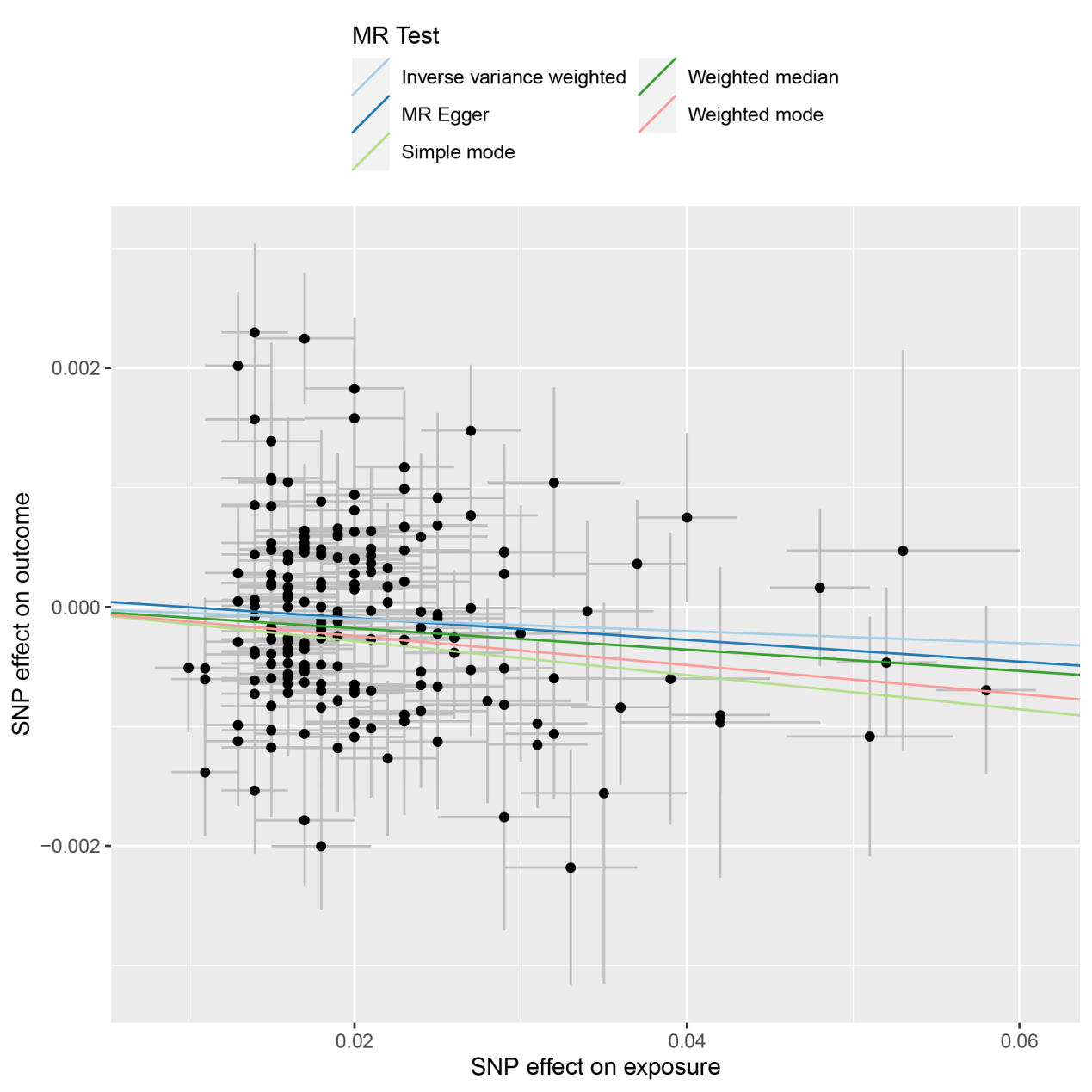


b.


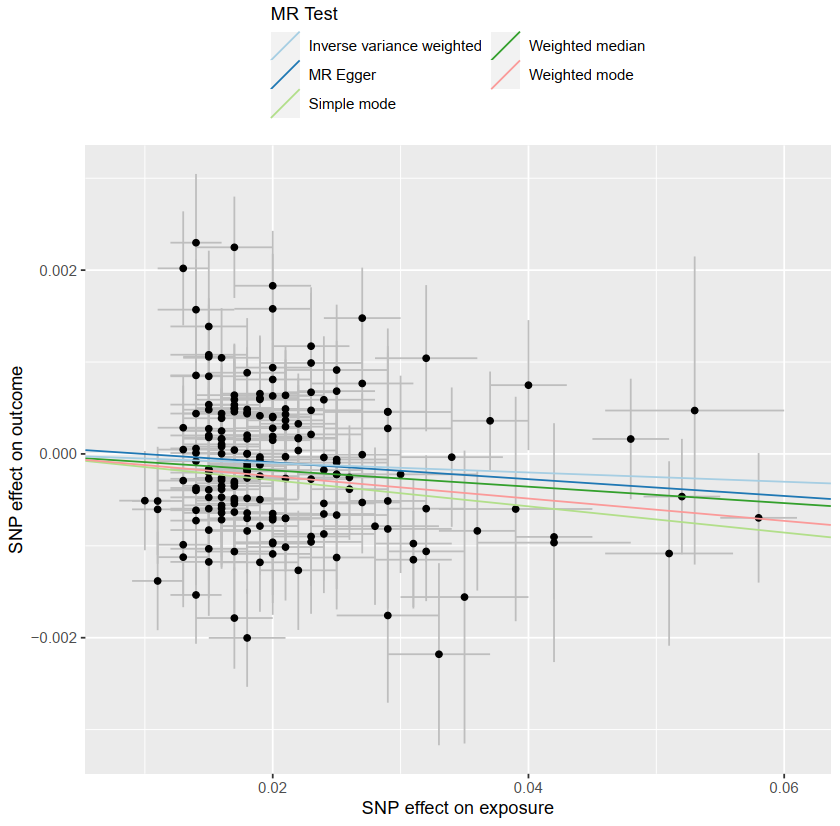


c.


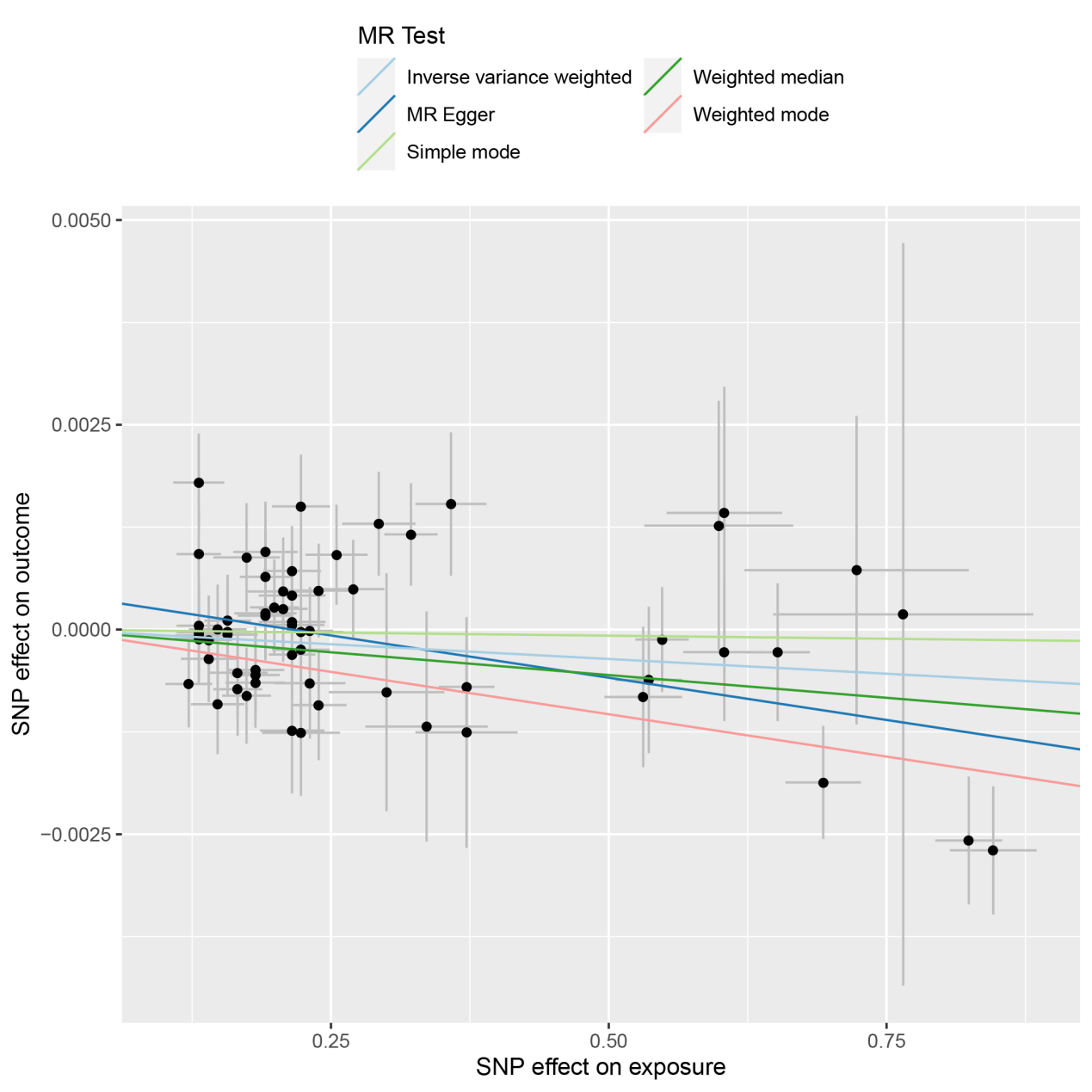


**Supplementary Figure S4.** **Leave-one-out plots of MR estimate concerning relationship between exposures and prostate cancer.** **a. IGFBP-3 levels and prostate cancer in PRACTICAL consortium, b. BMI and prostate cancer in UK Biobank, c. SLE and prostate cancer in UK Biobank.** Leave-one-out sensitivity analysis is performed to ascertain if an association is being disproportionately influenced by a single SNP. Each black point in the forest plot represents the MR analysis (using IVW) excluding that particular SNP.

a.


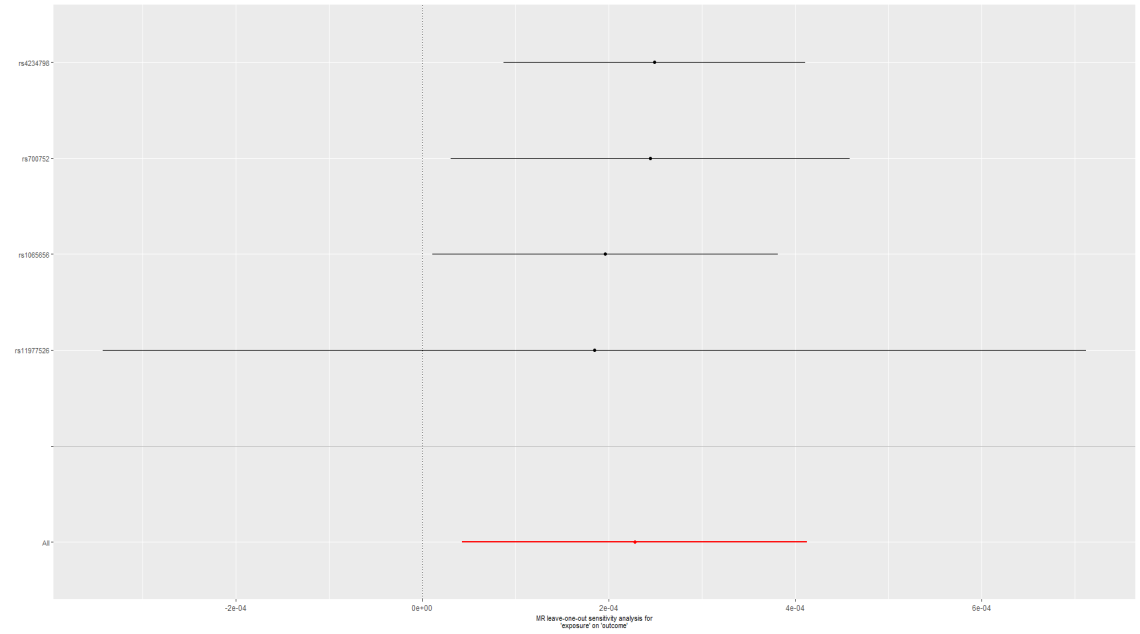


b.


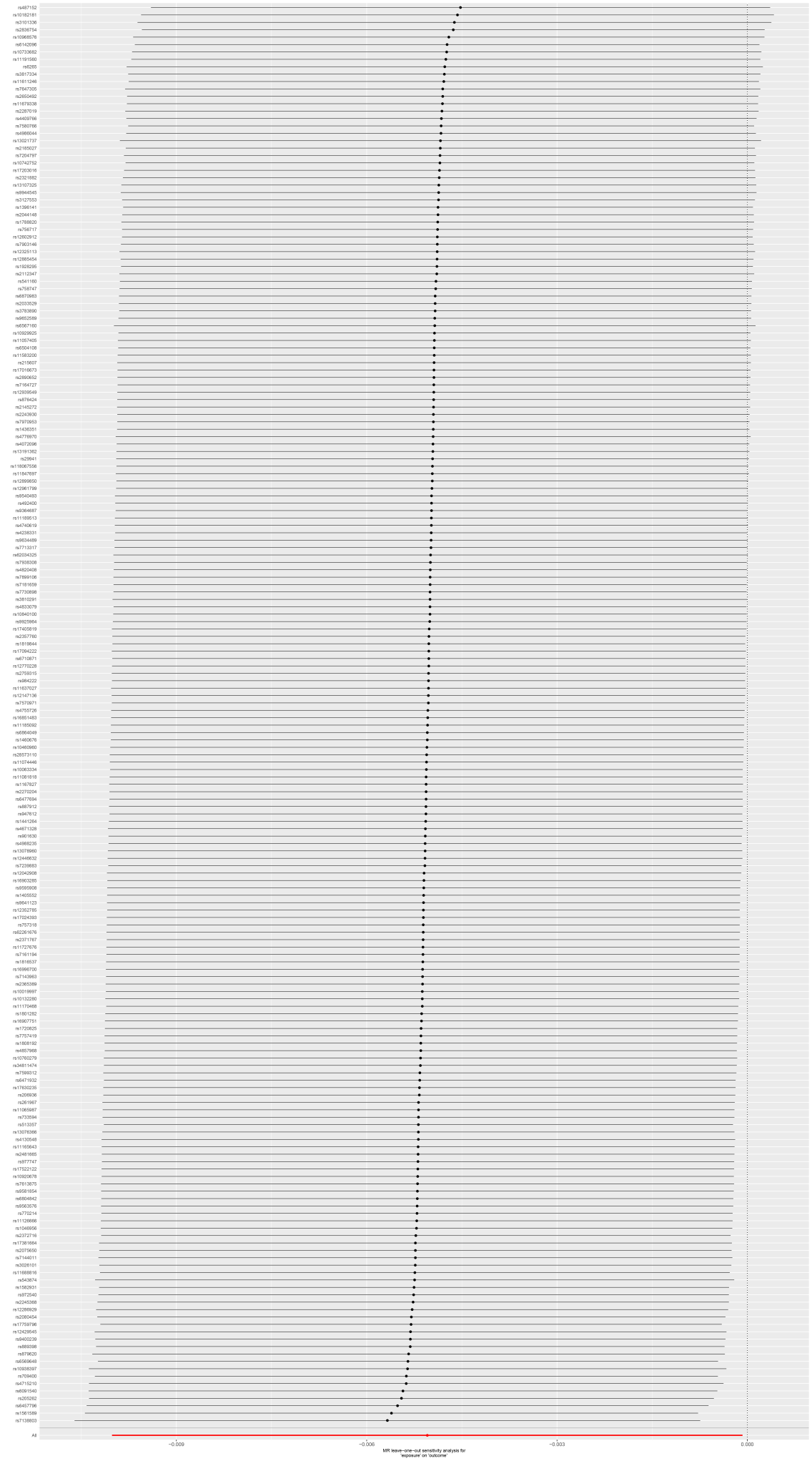


c.


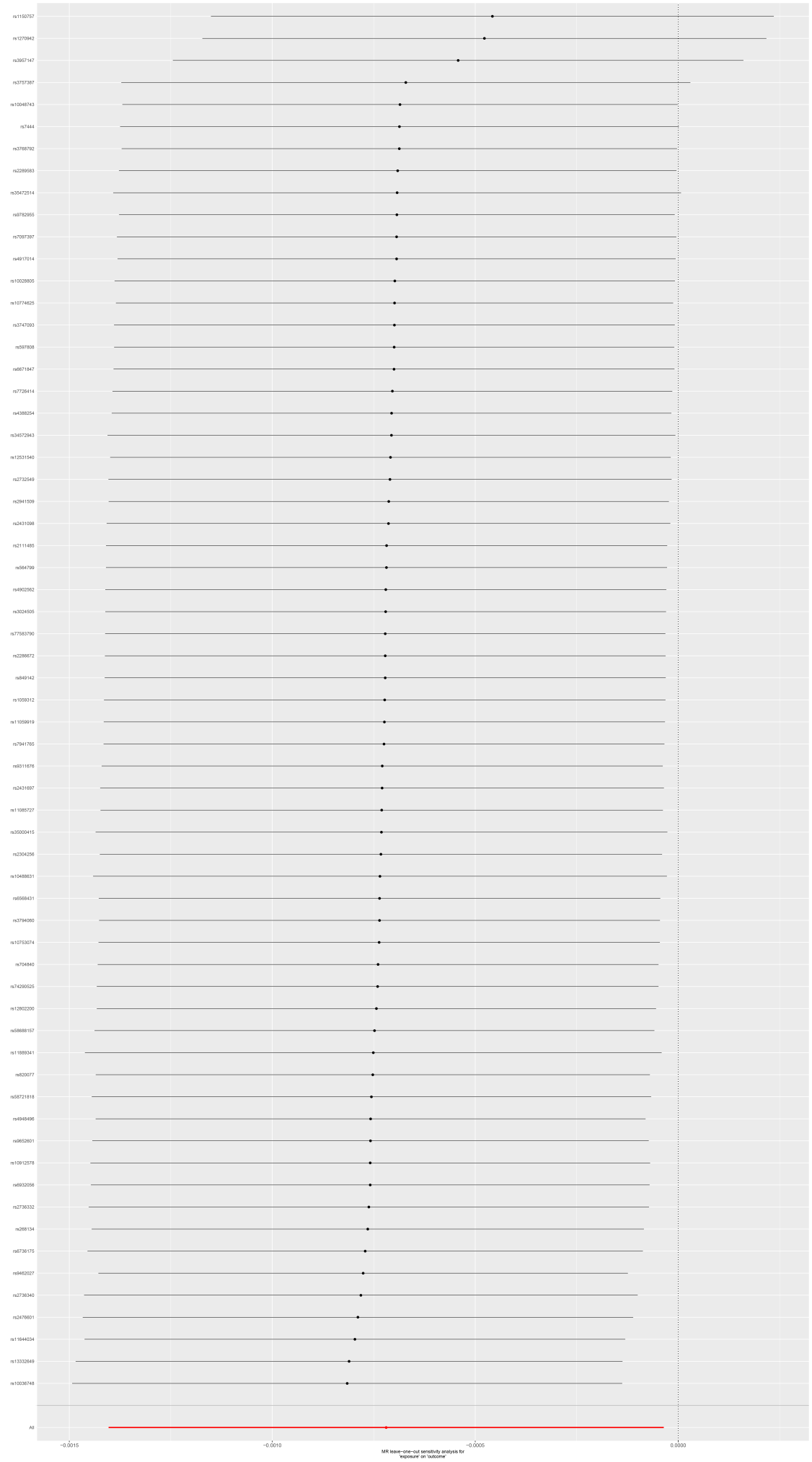


**Supplementary References:**

Ahola-Olli, A.V., Wurtz, P., Havulinna, A.S., Aalto, K., Pitkanen, N., Lehtimaki, T., et al. (2017). Genome-wide Association Study Identifies 27 Loci Influencing Concentrations of Circulating Cytokines and Growth Factors. *Am J Hum Genet* 100(1)**,** 40-50. doi: 10.1016/j.ajhg.2016.11.007.

Alberti, K.G., Eckel, R.H., Grundy, S.M., Zimmet, P.Z., Cleeman, J.I., Donato, K.A., et al. (2009). Harmonizing the metabolic syndrome: a joint interim statement of the International Diabetes Federation Task Force on Epidemiology and Prevention; National Heart, Lung, and Blood Institute; American Heart Association; World Heart Federation; International Atherosclerosis Society; and International Association for the Study of Obesity. *Circulation* 120(16)**,** 1640-1645. doi: 10.1161/CIRCULATIONAHA.109.192644.

Andrew, T., Hart, D.J., Snieder, H., de Lange, M., Spector, T.D., and MacGregor, A.J. (2001). Are twins and singletons comparable? A study of disease-related and lifestyle characteristics in adult women. *Twin Res* 4(6)**,** 464-477.

Au Yeung, S.L., and Schooling, C.M. (2019). Impact of glycemic traits, type 2 diabetes and metformin use on breast and prostate cancer risk: a Mendelian randomization study. *BMJ Open Diabetes Res Care* 7(1)**,** e000872. doi: 10.1136/bmjdrc-2019-000872.

Banda, Y., Kvale, M.N., Hoffmann, T.J., Hesselson, S.E., Ranatunga, D., Tang, H., et al. (2015). Characterizing Race/Ethnicity and Genetic Ancestry for 100,000 Subjects in the Genetic Epidemiology Research on Adult Health and Aging (GERA) Cohort. *Genetics* 200(4)**,** 1285-1295. doi: 10.1534/genetics.115.178616.

Benn, M., Tybjaerg-Hansen, A., Smith, G.D., and Nordestgaard, B.G. (2016). High body mass index and cancer risk-a Mendelian randomisation study. *Eur J Epidemiol* 31(9)**,** 879-892. doi: 10.1007/s10654-016-0147-5.

Bentham, J., Morris, D.L., Graham, D.S.C., Pinder, C.L., Tombleson, P., Behrens, T.W., et al. (2015). Genetic association analyses implicate aberrant regulation of innate and adaptive immunity genes in the pathogenesis of systemic lupus erythematosus. *Nat Genet* 47(12)**,** 1457-1464. doi: 10.1038/ng.3434.

Beutner, F., Teupser, D., Gielen, S., Holdt, L.M., Scholz, M., Boudriot, E., et al. (2011). Rationale and design of the Leipzig (LIFE) Heart Study: phenotyping and cardiovascular characteristics of patients with coronary artery disease. *PLoS One* 6(12)**,** e29070. doi: 10.1371/journal.pone.0029070.

Beynon, R.A., Richmond, R.C., Santos Ferreira, D.L., Ness, A.R., May, M., Smith, G.D., et al. (2019). Investigating the effects of lycopene and green tea on the metabolome of men at risk of prostate cancer: The ProDiet randomised controlled trial. *Int J Cancer* 144(8)**,** 1918-1928. doi: 10.1002/ijc.31929.

Bierut, L.J., Agrawal, A., Bucholz, K.K., Doheny, K.F., Laurie, C., Pugh, E., et al. (2010). A genome-wide association study of alcohol dependence. *Proc Natl Acad Sci U S A* 107(11)**,** 5082-5087. doi: 10.1073/pnas.0911109107.

Bild, D.E., Bluemke, D.A., Burke, G.L., Detrano, R., Diez Roux, A.V., Folsom, A.R., et al. (2002). Multi-ethnic study of atherosclerosis: objectives and design. *Am J Epidemiol* 156(9)**,** 871-881.

Boker, L.K., van Noord, P.A., van der Schouw, Y.T., Koot, N.V., Bueno de Mesquita, H.B., Riboli, E., et al. (2001). Prospect-EPIC Utrecht: study design and characteristics of the cohort population. European Prospective Investigation into Cancer and Nutrition. *Eur J Epidemiol* 17(11)**,** 1047-1053.

Bonilla, C., Lewis, S.J., Martin, R.M., Donovan, J.L., Hamdy, F.C., Neal, D.E., et al. (2016a). Pubertal development and prostate cancer risk: Mendelian randomization study in a population-based cohort. *BMC Med* 14**,** 66. doi: 10.1186/s12916-016-0602-x.

Bonilla, C., Lewis, S.J., Rowlands, M.A., Gaunt, T.R., Davey Smith, G., Gunnell, D., et al. (2016b). Assessing the role of insulin-like growth factors and binding proteins in prostate cancer using Mendelian randomization: Genetic variants as instruments for circulating levels. *Int J Cancer* 139(7)**,** 1520-1533. doi: 10.1002/ijc.30206.

Brunner, C., Davies, N.M., Martin, R.M., Eeles, R., Easton, D., Kote-Jarai, Z., et al. (2017). Alcohol consumption and prostate cancer incidence and progression: A Mendelian randomisation study. *Int J Cancer* 140(1)**,** 75-85. doi: 10.1002/ijc.30436.

Bull, C.J., Bonilla, C., Holly, J.M., Perks, C.M., Davies, N., Haycock, P., et al. (2016). Blood lipids and prostate cancer: a Mendelian randomization analysis. *Cancer Med* 5(6)**,** 1125-1136. doi: 10.1002/cam4.695.

Colditz, G.A., and Hankinson, S.E. (2005). The Nurses' Health Study: lifestyle and health among women. *Nat Rev Cancer* 5(5)**,** 388-396. doi: nrc1608 [pii]

10.1038/nrc1608.

Collin, S.M., Metcalfe, C., Palmer, T.M., Refsum, H., Lewis, S.J., Smith, G.D., et al. (2011). The causal roles of vitamin B(12) and transcobalamin in prostate cancer: can Mendelian randomization analysis provide definitive answers? *Int J Mol Epidemiol Genet* 2(4)**,** 316-327.

Coviello, A.D., Haring, R., Wellons, M., Vaidya, D., Lehtimaki, T., Keildson, S., et al. (2012). A genome-wide association meta-analysis of circulating sex hormone-binding globulin reveals multiple Loci implicated in sex steroid hormone regulation. *PLoS Genet* 8(7)**,** e1002805. doi: 10.1371/journal.pgen.1002805.

Davies, N.M., Gaunt, T.R., Lewis, S.J., Holly, J., Donovan, J.L., Hamdy, F.C., et al. (2015). The effects of height and BMI on prostate cancer incidence and mortality: a Mendelian randomization study in 20,848 cases and 20,214 controls from the PRACTICAL consortium. *Cancer Causes Control* 26(11)**,** 1603-1616. doi: 10.1007/s10552-015-0654-9.

Dawber, T.R., Meadors, G.F., and Moore, F.E., Jr. (1951). Epidemiological approaches to heart disease: the Framingham Study. *Am J Public Health Nations Health* 41(3)**,** 279-281. doi: 10.2105/ajph.41.3.279.

Dimitrakopoulou, V.I., Tsilidis, K.K., Haycock, P.C., Dimou, N.L., Al-Dabhani, K., Martin, R.M., et al. (2017). Circulating vitamin D concentration and risk of seven cancers: Mendelian randomisation study. *BMJ* 359**,** j4761. doi: 10.1136/bmj.j4761.

Dupuis, J., Langenberg, C., Prokopenko, I., Saxena, R., Soranzo, N., Jackson, A.U., et al. (2010). New genetic loci implicated in fasting glucose homeostasis and their impact on type 2 diabetes risk. *Nat Genet* 42(2)**,** 105-116. doi: 10.1038/ng.520.

Ferrucci, L., Bandinelli, S., Benvenuti, E., Di Iorio, A., Macchi, C., Harris, T.B., et al. (2000). Subsystems contributing to the decline in ability to walk: bridging the gap between epidemiology and geriatric practice in the InCHIANTI study. *J Am Geriatr Soc* 48(12)**,** 1618-1625.

Friedman, G.D., Cutter, G.R., Donahue, R.P., Hughes, G.H., Hulley, S.B., Jacobs, D.R., Jr., et al. (1988). CARDIA: study design, recruitment, and some characteristics of the examined subjects. *J Clin Epidemiol* 41(11)**,** 1105-1116.

Galante, J., Adamska, L., Young, A., Young, H., Littlejohns, T.J., Gallacher, J., et al. (2016). The acceptability of repeat Internet-based hybrid diet assessment of previous 24-h dietary intake: administration of the Oxford WebQ in UK Biobank. *Br J Nutr* 115(4)**,** 681-686. doi: 10.1017/S0007114515004821.

Gaziano, J.M., Concato, J., Brophy, M., Fiore, L., Pyarajan, S., Breeling, J., et al. (2016). Million Veteran Program: A mega-biobank to study genetic influences on health and disease. *J Clin Epidemiol* 70**,** 214-223. doi: 10.1016/j.jclinepi.2015.09.016.

German, C.A., Sinsheimer, J.S., Klimentidis, Y.C., Zhou, H., and Zhou, J.J. (2020). Ordered multinomial regression for genetic association analysis of ordinal phenotypes at Biobank scale. *Genet Epidemiol* 44(3)**,** 248-260. doi: 10.1002/gepi.22276.

Gomez-Acebo, I., Dierssen-Sotos, T., Palazuelos, C., Fernandez-Navarro, P., Castano-Vinyals, G., Alonso-Molero, J., et al. (2018). Pigmentation phototype and prostate and breast cancer in a select Spanish population-A Mendelian randomization analysis in the MCC-Spain study. *PLoS One* 13(8)**,** e0201750. doi: 10.1371/journal.pone.0201750.

Gottesman, O., Kuivaniemi, H., Tromp, G., Faucett, W.A., Li, R., Manolio, T.A., et al. (2013). The Electronic Medical Records and Genomics (eMERGE) Network: past, present, and future. *Genet Med* 15(10)**,** 761-771. doi: 10.1038/gim.2013.72.

Hellwege, J.N., Stallings, S., Torstenson, E.S., Carroll, R., Borthwick, K.M., Brilliant, M.H., et al. (2019). Heritability and genome-wide association study of benign prostatic hyperplasia (BPH) in the eMERGE network. *Sci Rep* 9(1)**,** 6077. doi: 10.1038/s41598-019-42427-z.

Hoffmann, T.J., Choquet, H., Yin, J., Banda, Y., Kvale, M.N., Glymour, M., et al. (2018). A Large Multiethnic Genome-Wide Association Study of Adult Body Mass Index Identifies Novel Loci. *Genetics* 210(2)**,** 499-515. doi: 10.1534/genetics.118.301479.

Hofman, A., van Duijn, C.M., Franco, O.H., Ikram, M.A., Janssen, H.L., Klaver, C.C., et al. (2011). The Rotterdam Study: 2012 objectives and design update. *Eur J Epidemiol* 26(8)**,** 657-686. doi: 10.1007/s10654-011-9610-5.

International Multiple Sclerosis Genetics, C. (2019). Multiple sclerosis genomic map implicates peripheral immune cells and microglia in susceptibility. *Science* 365(6460). doi: 10.1126/science.aav7188.

International Multiple Sclerosis Genetics, C., Wellcome Trust Case Control, C., Sawcer, S., Hellenthal, G., Pirinen, M., Spencer, C.C., et al. (2011). Genetic risk and a primary role for cell-mediated immune mechanisms in multiple sclerosis. *Nature* 476(7359)**,** 214-219. doi: 10.1038/nature10251.

Jarvelin, M.R., Sovio, U., King, V., Lauren, L., Xu, B., McCarthy, M.I., et al. (2004). Early life factors and blood pressure at age 31 years in the 1966 northern Finland birth cohort. *Hypertension* 44(6)**,** 838-846. doi: 01.HYP.0000148304.33869.ee [pii]

10.1161/01.HYP.0000148304.33869.ee.

Jiang, X., Dimou, N.L., Al-Dabhani, K., Lewis, S.J., Martin, R.M., Haycock, P.C., et al. (2019). Circulating vitamin D concentrations and risk of breast and prostate cancer: a Mendelian randomization study. *Int J Epidemiol* 48(5)**,** 1416-1424. doi: 10.1093/ije/dyy284.

Justice, A.E., Winkler, T.W., Feitosa, M.F., Graff, M., Fisher, V.A., Young, K., et al. (2017). Genome-wide meta-analysis of 241,258 adults accounting for smoking behaviour identifies novel loci for obesity traits. *Nat Commun* 8**,** 14977. doi: 10.1038/ncomms14977.

Kaplan, R.C., Petersen, A.K., Chen, M.H., Teumer, A., Glazer, N.L., Doring, A., et al. (2011). A genome-wide association study identifies novel loci associated with circulating IGF-I and IGFBP-3. *Hum Mol Genet* 20(6)**,** 1241-1251. doi: 10.1093/hmg/ddq560.

Kapoor, M., Wang, J.C., Wetherill, L., Le, N., Bertelsen, S., Hinrichs, A.L., et al. (2013). A meta-analysis of two genome-wide association studies to identify novel loci for maximum number of alcoholic drinks. *Hum Genet* 132(10)**,** 1141-1151. doi: 10.1007/s00439-013-1318-z.

Karlsson Linner, R., Biroli, P., Kong, E., Meddens, S.F.W., Wedow, R., Fontana, M.A., et al. (2019). Genome-wide association analyses of risk tolerance and risky behaviors in over 1 million individuals identify hundreds of loci and shared genetic influences. *Nat Genet* 51(2)**,** 245-257. doi: 10.1038/s41588-018-0309-3.

Khankari, N.K., Murff, H.J., Zeng, C., Wen, W., Eeles, R.A., Easton, D.F., et al. (2016a). Polyunsaturated fatty acids and prostate cancer risk: a Mendelian randomisation analysis from the PRACTICAL consortium. *Br J Cancer* 115(5)**,** 624-631. doi: 10.1038/bjc.2016.228.

Khankari, N.K., Shu, X.O., Wen, W., Kraft, P., Lindstrom, S., Peters, U., et al. (2016b). Association between Adult Height and Risk of Colorectal, Lung, and Prostate Cancer: Results from Meta-analyses of Prospective Studies and Mendelian Randomization Analyses. *PLoS Med* 13(9)**,** e1002118. doi: 10.1371/journal.pmed.1002118.

Klarin, D., Damrauer, S.M., Cho, K., Sun, Y.V., Teslovich, T.M., Honerlaw, J., et al. (2018). Genetics of blood lipids among ~300,000 multi-ethnic participants of the Million Veteran Program. *Nat Genet* 50(11)**,** 1514-1523. doi: 10.1038/s41588-018-0222-9.

Lemaitre, R.N., Tanaka, T., Tang, W., Manichaikul, A., Foy, M., Kabagambe, E.K., et al. (2011). Genetic loci associated with plasma phospholipid n-3 fatty acids: a meta-analysis of genome-wide association studies from the CHARGE Consortium. *PLoS Genet* 7(7)**,** e1002193. doi: 10.1371/journal.pgen.1002193.

Li, M., Kwok, M.K., Fong, S.S.M., and Schooling, C.M. (2019). Indoleamine 2,3-dioxygenase and ischemic heart disease: a Mendelian Randomization study. *Sci Rep* 9(1)**,** 8491. doi: 10.1038/s41598-019-44819-7.

Lind, L. (2019). Genome-Wide Association Study of the Metabolic Syndrome in UK Biobank. *Metab Syndr Relat Disord* 17(10)**,** 505-511. doi: 10.1089/met.2019.0070.

Loeffler, M., Engel, C., Ahnert, P., Alfermann, D., Arelin, K., Baber, R., et al. (2015). The LIFE-Adult-Study: objectives and design of a population-based cohort study with 10,000 deeply phenotyped adults in Germany. *BMC Public Health* 15**,** 691. doi: 10.1186/s12889-015-1983-z.

Lorentzon, M., Swanson, C., Andersson, N., Mellstrom, D., and Ohlsson, C. (2005). Free testosterone is a positive, whereas free estradiol is a negative, predictor of cortical bone size in young Swedish men: the GOOD study. *J Bone Miner Res* 20(8)**,** 1334-1341.

Manousaki, D., Mitchell, R., Dudding, T., Haworth, S., Harroud, A., Forgetta, V., et al. (2020). Genome-wide Association Study for Vitamin D Levels Reveals 69 Independent Loci. *Am J Hum Genet* 106(3)**,** 327-337. doi: 10.1016/j.ajhg.2020.01.017.

McDonald, W.I., Compston, A., Edan, G., Goodkin, D., Hartung, H.P., Lublin, F.D., et al. (2001). Recommended diagnostic criteria for multiple sclerosis: guidelines from the International Panel on the diagnosis of multiple sclerosis. *Ann Neurol* 50(1)**,** 121-127. doi: 10.1002/ana.1032.

Melzer, D., Perry, J.R., Hernandez, D., Corsi, A.M., Stevens, K., Rafferty, I., et al. (2008). A genome-wide association study identifies protein quantitative trait loci (pQTLs). *PLoS Genet* 4(5)**,** e1000072. doi: 10.1371/journal.pgen.1000072.

Nalls, M.A., Blauwendraat, C., Vallerga, C.L., Heilbron, K., Bandres-Ciga, S., Chang, D., et al. (2019). Identification of novel risk loci, causal insights, and heritable risk for Parkinson's disease: a meta-analysis of genome-wide association studies. *Lancet Neurol* 18(12)**,** 1091-1102. doi: 10.1016/S1474-4422(19)30320-5.

Ong, J.S., Law, M.H., An, J., Han, X., Gharahkhani, P., Whiteman, D.C., et al. (2019). Association between coffee consumption and overall risk of being diagnosed with or dying from cancer among >300 000 UK Biobank participants in a large-scale Mendelian randomization study. *Int J Epidemiol* 48(5)**,** 1447-1456. doi: 10.1093/ije/dyz144.

Orho-Melander, M., Hindy, G., Borgquist, S., Schulz, C.A., Manjer, J., Melander, O., et al. (2018). Blood lipid genetic scores, the HMGCR gene and cancer risk: a Mendelian randomization study. *Int J Epidemiol* 47(2)**,** 495-505. doi: 10.1093/ije/dyx237.

Pardinas, A.F., Holmans, P., Pocklington, A.J., Escott-Price, V., Ripke, S., Carrera, N., et al. (2018). Common schizophrenia alleles are enriched in mutation-intolerant genes and in regions under strong background selection. *Nat Genet* 50(3)**,** 381-389. doi: 10.1038/s41588-018-0059-2.

Polman, C.H., Reingold, S.C., Edan, G., Filippi, M., Hartung, H.P., Kappos, L., et al. (2005). Diagnostic criteria for multiple sclerosis: 2005 revisions to the "McDonald Criteria". *Ann Neurol* 58(6)**,** 840-846. doi: 10.1002/ana.20703.

Poser, C.M., Paty, D.W., Scheinberg, L., McDonald, W.I., Davis, F.A., Ebers, G.C., et al. (1983). New diagnostic criteria for multiple sclerosis: guidelines for research protocols. *Ann Neurol* 13(3)**,** 227-231. doi: 10.1002/ana.410130302.

Psaty, B.M., O'Donnell, C.J., Gudnason, V., Lunetta, K.L., Folsom, A.R., Rotter, J.I., et al. (2009). Cohorts for Heart and Aging Research in Genomic Epidemiology (CHARGE) Consortium: Design of prospective meta-analyses of genome-wide association studies from 5 cohorts. *Circ Cardiovasc Genet* 2(1)**,** 73-80. doi: 10.1161/CIRCGENETICS.108.829747.

Raitakari, O.T., Juonala, M., Ronnemaa, T., Keltikangas-Jarvinen, L., Rasanen, L., Pietikainen, M., et al. (2008). Cohort profile: the cardiovascular risk in Young Finns Study. *Int J Epidemiol* 37(6)**,** 1220-1226.

Schizophrenia Working Group of the Psychiatric Genomics, C. (2014). Biological insights from 108 schizophrenia-associated genetic loci. *Nature* 511(7510)**,** 421-427. doi: 10.1038/nature13595.

Smith Byrne, K., Appleby, P.N., Key, T.J., Holmes, M.V., Fensom, G.K., Agudo, A., et al. (2019). The role of plasma microseminoprotein-beta in prostate cancer: an observational nested case-control and Mendelian randomization study in the European prospective investigation into cancer and nutrition. *Ann Oncol* 30(6)**,** 983-989. doi: 10.1093/annonc/mdz121.

Spector, T.D., and Williams, F.M. (2006). The UK Adult Twin Registry (TwinsUK). *Twin Res Hum Genet* 9(6)**,** 899-906.

Stefanick, M.L., Cochrane, B.B., Hsia, J., Barad, D.H., Liu, J.H., and Johnson, S.R. (2003). The Women's Health Initiative postmenopausal hormone trials: overview and baseline characteristics of participants. *Ann Epidemiol* 13(9 Suppl)**,** S78-86.

Sudlow, C., Gallacher, J., Allen, N., Beral, V., Burton, P., Danesh, J., et al. (2015). UK biobank: an open access resource for identifying the causes of a wide range of complex diseases of middle and old age. *PLoS Med* 12(3)**,** e1001779. doi: 10.1371/journal.pmed.1001779.

Tan, V.Y., Biernacka, K.M., Dudding, T., Bonilla, C., Gilbert, R., Kaplan, R.C., et al. (2018). Reassessing the Association between Circulating Vitamin D and IGFBP-3: Observational and Mendelian Randomization Estimates from Independent Sources. *Cancer Epidemiol Biomarkers Prev* 27(12)**,** 1462-1471. doi: 10.1158/1055-9965.EPI-18-0113.

Tanaka, T., Scheet, P., Giusti, B., Bandinelli, S., Piras, M.G., Usala, G., et al. (2009). Genome-wide association study of vitamin B6, vitamin B12, folate, and homocysteine blood concentrations. *Am J Hum Genet* 84(4)**,** 477-482. doi: 10.1016/j.ajhg.2009.02.011.

Taylor, A.E., Martin, R.M., Geybels, M.S., Stanford, J.L., Shui, I., Eeles, R., et al. (2017). Investigating the possible causal role of coffee consumption with prostate cancer risk and progression using Mendelian randomization analysis. *Int J Cancer* 140(2)**,** 322-328. doi: 10.1002/ijc.30462.

Tobacco, and Genetics, C. (2010). Genome-wide meta-analyses identify multiple loci associated with smoking behavior. *Nat Genet* 42(5)**,** 441-447. doi: 10.1038/ng.571.

Völzke, H., Alte, D., Schmidt, C.O., Radke, D., Lorbeer, R., Friedrich, N., et al. (2011). Cohort profile: the study of health in pomerania. *Int J Epidemiol* 40(2)**,** 294-307.

Wang, J.C., Foroud, T., Hinrichs, A.L., Le, N.X., Bertelsen, S., Budde, J.P., et al. (2013). A genome-wide association study of alcohol-dependence symptom counts in extended pedigrees identifies C15orf53. *Mol Psychiatry* 18(11)**,** 1218-1224. doi: 10.1038/mp.2012.143.

Wichmann, H.E., Gieger, C., and Illig, T. (2005). KORA-gen--resource for population genetics, controls and a broad spectrum of disease phenotypes. *Gesundheitswesen* 67 Suppl 1**,** S26-30.

Wood, A.R., Esko, T., Yang, J., Vedantam, S., Pers, T.H., Gustafsson, S., et al. (2014). Defining the role of common variation in the genomic and biological architecture of adult human height. *Nat Genet* 46(11)**,** 1173-1186. doi: 10.1038/ng.3097.

Wu, Y., Byrne, E.M., Zheng, Z., Kemper, K.E., Yengo, L., Mallett, A.J., et al. (2019). Genome-wide association study of medication-use and associated disease in the UK Biobank. *Nat Commun* 10(1)**,** 1891. doi: 10.1038/s41467-019-09572-5.

Xue, A., Wu, Y., Zhu, Z., Zhang, F., Kemper, K.E., Zheng, Z., et al. (2018). Genome-wide association analyses identify 143 risk variants and putative regulatory mechanisms for type 2 diabetes. *Nat Commun* 9(1)**,** 2941. doi: 10.1038/s41467-018-04951-w.

Yarmolinsky, J., Berryman, K., Langdon, R., Bonilla, C., consortium, P., Davey Smith, G., et al. (2018a). Mendelian randomization does not support serum calcium in prostate cancer risk. *Cancer Causes Control* 29(11)**,** 1073-1080. doi: 10.1007/s10552-018-1081-5.

Yarmolinsky, J., Bonilla, C., Haycock, P.C., Langdon, R.J.Q., Lotta, L.A., Langenberg, C., et al. (2018b). Circulating Selenium and Prostate Cancer Risk: A Mendelian Randomization Analysis. *J Natl Cancer Inst* 110(9)**,** 1035-1038. doi: 10.1093/jnci/djy081.

Zhang, C., Doherty, J.A., Burgess, S., Hung, R.J., Lindstrom, S., Kraft, P., et al. (2015). Genetic determinants of telomere length and risk of common cancers: a Mendelian randomization study. *Hum Mol Genet* 24(18)**,** 5356-5366. doi: 10.1093/hmg/ddv252.

Zhang, H., Qin, J., Berndt, S.I., Albanes, D., Deng, L., Gail, M.H., et al. (2020). On Mendelian randomization analysis of case-control study. *Biometrics* 76(2)**,** 380-391. doi: 10.1111/biom.13166.

Zhong, V.W., Kuang, A., Danning, R.D., Kraft, P., van Dam, R.M., Chasman, D.I., et al. (2019). A genome-wide association study of bitter and sweet beverage consumption. *Hum Mol Genet* 28(14)**,** 2449-2457. doi: 10.1093/hmg/ddz061.
